# Structural Dependence of Catenand Effect: Thermodynamic and Kinetic Modulation of Catenane Coordination Properties via Ring‐size and Exocyclic Substituent Variation

**DOI:** 10.1002/anie.202514599

**Published:** 2025-10-06

**Authors:** Yulin Deng, Zi‐Gang Lu, Samuel Kin‐Man Lai, Man Pang Tang, Xiaoyong Mo, Shan He, David Lee Phillips, Edmund Chun Ming Tse, Ho Yu Au‐Yeung

**Affiliations:** ^1^ Department of Chemistry The University of Hong Kong Pokfulam Road Hong Kong China; ^2^ CAS‐HKU Joint Laboratory on New Materials The University of Hong Kong Pokfulam Road Hong Kong China; ^3^ Laboratory for Synthetic Chemistry and Chemical Biology Limited Unites 1503–1511, Building 17 W, Hong Kong Science Park, New Territories Hong Kong China; ^4^ State Key Laboratory of Synthetic Chemistry The University of Hong Kong Pokfulam Road Hong Kong China

**Keywords:** Catenane, Coordination ligand, Copper, Mechanical interlocking, Switchable catalyst

## Abstract

Although the effects of ligand interlocking on specific features of coordinated metal were first reported in 1980s’, strategies to precisely control these features through structural modifications of interlocked ligands remains underdeveloped. This limitation has hindered the broader exploitation of this unique class of coordination compounds across various fields of transition metal chemistry. Through a systematic comparison and detail analysis of a series of Cu^I^ catenane complexes, we show in this work that the size of the interlocked rings and exocyclic substituents are important structural parameters that regulate the exposure of the metal coordination sphere, which also influences the coordination geometry, electronic structures, spectroscopic, photophysical and electrochemical properties, as well as thermodynamic stability, ligand exchange kinetics, and chemical reactivity of the coordinated metal. Relationship between the structural features of the catenane and the extent of these effects is also revealed. These insights not only faciliate the rational design of a new type of switchable catenane catalyst wherein the interlocked structure is preserved, but also establish new principles for leveraging the unique effects of mechanical interlocking for diverse applications involving coordination complexes.

## Graphical abstract

By modifying ring size and exocyclic substituents, effects of ligand interlocking on metal coordination features is explored. Through systematic analysis of Cu^I^ catenanes, relationship between structural parameters of the interlocked ligands and coordination properties like geometry, stability, and reactivity is established. Insights from this study enable the design of switchable catalysts and establish principles for leveraging mechanical interlocking in coordination chemistry.

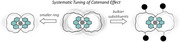

## Introduction

Coordination ligands are central to the structures, properties, and functions of transition metal complexes. While tuning of ligand stereoelectronic properties via new covalent design is routine, harnessing mechanical bond and ligand interlocking for new transition metal chemistry and applications is yet to reach its full potential.^[^ ^1^, ^2^, ^3^, ^4^ ^]^ Although a wealth of molecular switches and machines derived from mechanically interlocked molecules (MIMs) have been obtained from metal‐templated synthesis,^[^ ^5^, ^6^, ^7^, ^8^, ^9^, ^10^, ^11^ ^]^ with metal coordination/de‐coordination being a major mechanism underlying their operations,^[^ ^12^, ^13^, ^14^, ^15^, ^16^, ^17^, ^18^, ^19^, ^20^, ^21^, ^22^, ^23^ ^]^ little is known about the effects of ligand interlocking on the chemistry of the coordinated metal ions,^[^ ^24^, ^25^, ^26^, ^27^ ^]^ and progress on developing metal complexes supported by interlocked ligands for catalysis,^[^ ^28^, ^29^, ^30^, ^31^, ^32^ ^]^ materials,^[^ ^33^, ^34^, ^35^, ^36^, ^37^, ^38^ ^]^ biological applications,^[^ ^39^, ^40^ ^]^ and other areas is therefore slow.

In this regard, understanding effects of ligand interlocking on the fundamental thermodynamic, kinetic, electronic, and chemical properties of the coordinated metals, as well as relating these effects to the structure of the interlocked ligands will be necessary to realize their rational design for specific functions. Indeed, early works by Sauvage have shown that ligand interlocking would impede ligand exchange and alter redox properties of transition metals (which is known as “catenand effect”),^[^ ^24^, ^25^, ^26^ ^]^ but studies extending to other coordination features remain scarce and scattered.^[^ ^27^, ^41^, ^42^ ^]^ In particular, obtaining MIMs for systematic structure‐property relationship studies is not trivial, because templated synthesis could be sensitive to subtle structural variation in the building blocks.^[^ ^43^, ^44^, ^45^, ^46^ ^]^ Hence, strategies to control the extent of catenand effect remain elusive, and sometimes inconclusive or conflicting views on the roles of ligand interlocking may also arise. For example, while previous works have shown Cu^I^ catenane (and rotaxane) complexes are less active in mediating click reactions when compared to the non‐interlocked analogues,^[^ ^47^, ^48^ ^]^ our studies on Cu^I^ catenanes in catalyzing cross‐coupling reactions have shown that the interlocking is essential in maintaining the catalytic activity and selectivity.^[^ ^49^, ^50^, ^51^, ^52^, ^53^ ^]^ Although the exact interlocked structures, reaction types and conditions in these examples are not fully comparable, it is obvious that a systematic and in‐depth understanding of the relationship between the catenane structure, coordination features, and metal reactivity is necessary for fully unleashing the potential of this unique class of coordination compounds.

**Scheme 1:**
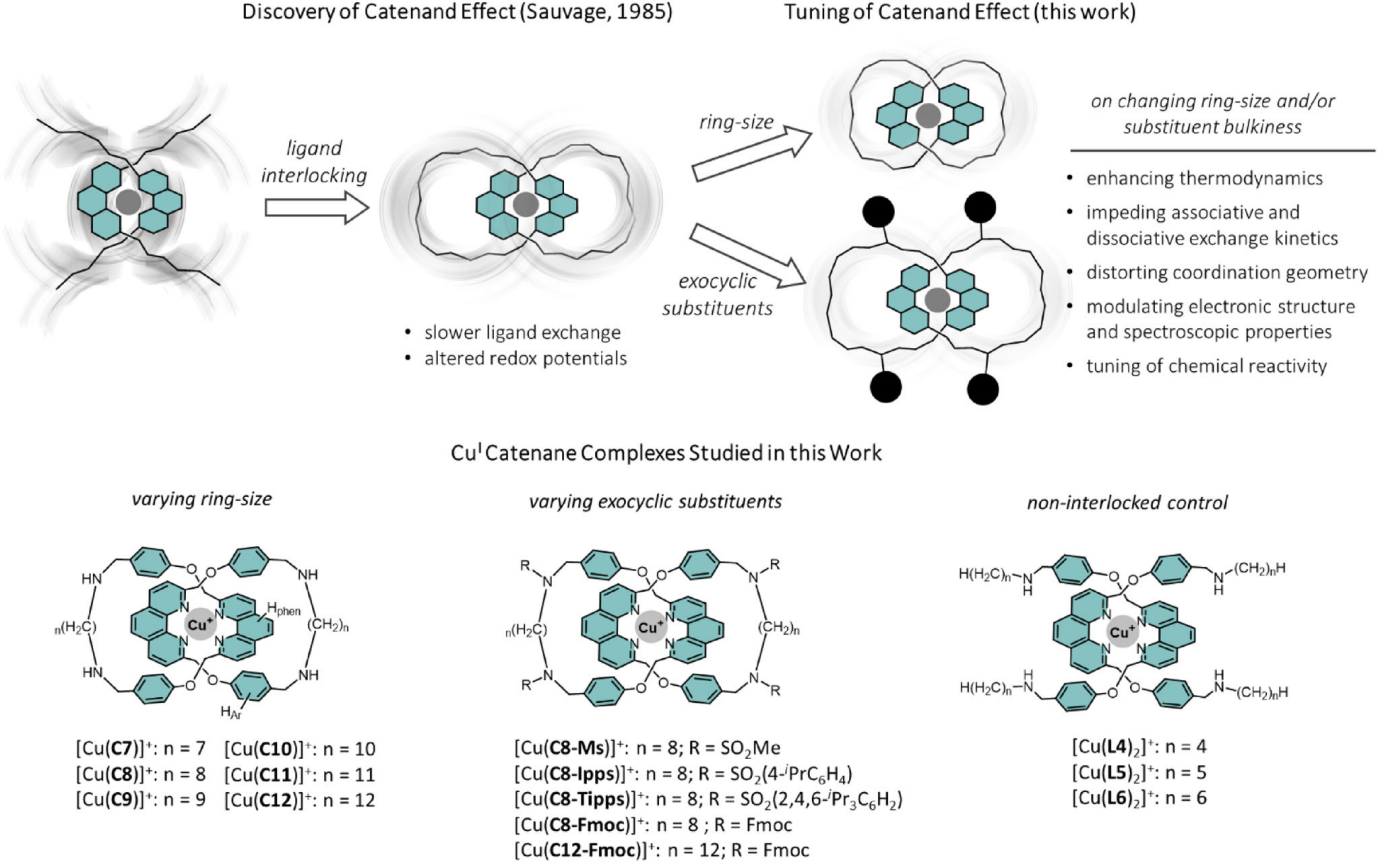
Proposed tuning of catenand effect via varying the ring‐size and the steric hindrance of the exocyclic substituents on the catenane ligand, which would directly influence the relative motions and (co)conformational flexibility, and hence the chemical properties of the coordinated metal. Structures of the Cu^I^ catenane complexes studied in this work are also shown.

In fact, the main characteristics of a metal complex coordinated by a catenane is their dynamic yet restricted ligand motions. As such, we propose that the extent of catenand effect will be related to, and hence controllable by the ability of the interlocked rings to undergo (co)conformational changes around the metal. In particular, changing the size of the interlocked rings and tightness of the interlocking, and/or tuning the steric bulkiness of the exocyclic substituents are anticipated to be efficient ligand modification strategies for controlling the (co)conformational motions, and thus the influences on the structure, properties, and reactivity of the coordinated metal (Scheme 1).

## Results and Discussion

In this work, a series of [2]catenanes composed of two interlocked, phenanthroline‐containing macrocycles for Cu^I^ coordination was studied (Scheme 1). In addition to the good efficiency and high yields of Cu^I^‐templated catenane synthesis,^[^ ^54^, ^55^, ^56^, ^57^, ^58^, ^59^, ^60^, ^61^, ^62^ ^]^ the tetrahedral‐planar geometry change during Cu^II/I^ conversion, which is often involved in the chemical reactivity of the transition metal, will also be sensitive to the tightness of the interlocking. Reductive amination of the Cu^+^‐templated precursor by *α*,*ω*‐alkyldiamine of different length gave [Cu(**Cn**)](PF_6_) (*n* = 7–12) with varying ring size, and the control non‐interlocked complexes [Cu(**Ln**)_2_](PF_6_) (*n* = 4–6) were also obtained similarly using *n*‐butyl, *n*‐pentyl and *n*‐hexylamine (Scheme 1). The secondary amines from the reductive amination can also be easily functionalized via substitution or condensation, and the medium‐sized [Cu(**C8**)](PF_6_) in the series was further reacted with an appropriate sulfonyl chloride to give [Cu(**C8‐Ms**)](PF_6_), [Cu(**C8‐Ipps**)](PF_6_), and [Cu(**C8‐Tipps**)](PF_6_) for studying the role of exocyclic substituents on the extent of the catenand effect.

### Coordination Structure

**Figure 1:**
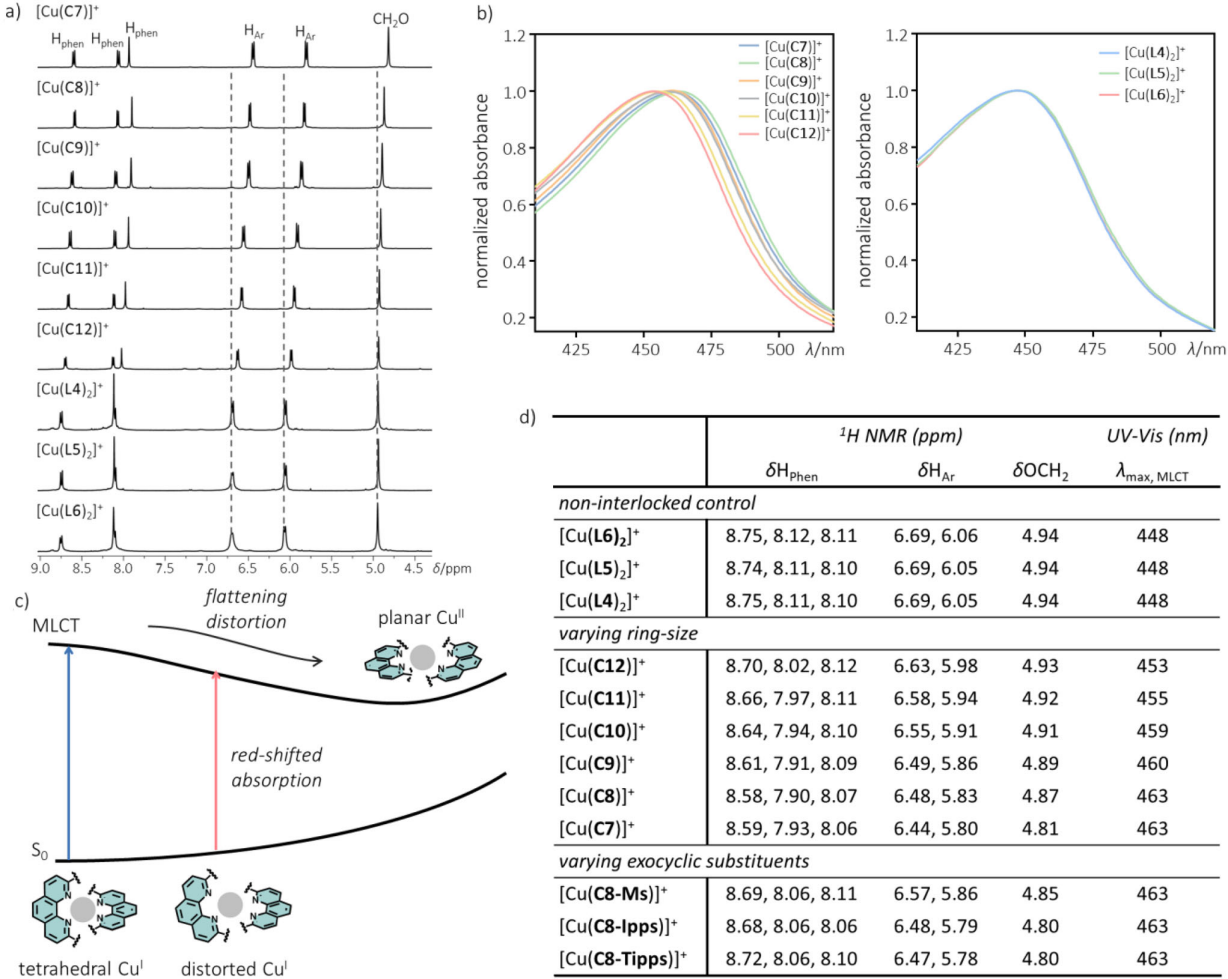
a) Partial ^1^H NMR spectra (500 MHz, DMSO‐*d*_6_, 298 K) and b) normalized UV–Vis spectra of the Cu^I^ catenanes of varying ring‐size and their non‐interlocked controls (as PF_6_^−^ salt); c) a simplified energy diagram showing the flattening distortion in the excited state and red‐shifted MLCT absorption of Cu(I) bis(phenanthroline) complexes; and d) summary of selected spectroscopic data.

Effects of the catenane ligands on the Cu^I^ coordination structure were first investigated by ^1^H NMR spectroscopy. Comparing the ^1^H NMR spectra of [Cu(**L4**)_2_]^+^, [Cu(**L5**)_2_]^+^, and [Cu(**L6**)_2_]^+^ (as PF_6_^–^ salt), no observable difference was found in the chemical shifts of proton signals from the phenanthroline (H_phen_), phenyl linkers (H_Ar_), and the methylene protons next to the ether oxygen (H_1_), suggesting the coordination environment in these non‐interlocked complexes is highly similar (Figure 1a and Table S1). In contrast, small but observable chemical shift changes were found for H_phen_, H_Ar_, H_1_, and the methylene protons in the aliphatic linkers in [Cu(**C12**)]^+^, showing that both the primary and secondary coordination environment are affected upon ligand interlocking. When the size of the catenane reduces from [Cu(**C12**)]^+^ to [Cu(**C7**)]^+^, the chemical shift changes become more obvious with a larger magnitude, indicating a more significant structural distortion. Yet, these structural changes are overall subtle, and their IR and resonance Raman spectra are nearly indistinguishable (Figures S28 and S29; Tables S2 and S3). On the other hand, ^1^H NMR spectra of [Cu(**C8‐Ms**)]^+^, [Cu(**C8‐Ipps**)]^+^, and [Cu(**C8‐Tipps**)]^+^, which are all derived from [Cu(**C8**)]^+^ with the same macrocycle size, showed similar chemical shifts for H_phen_, indicating that the exocyclic substituents have only a minimal effect on the primary coordination structure in these Cu^I^ catenanes.

**Figure 2:**
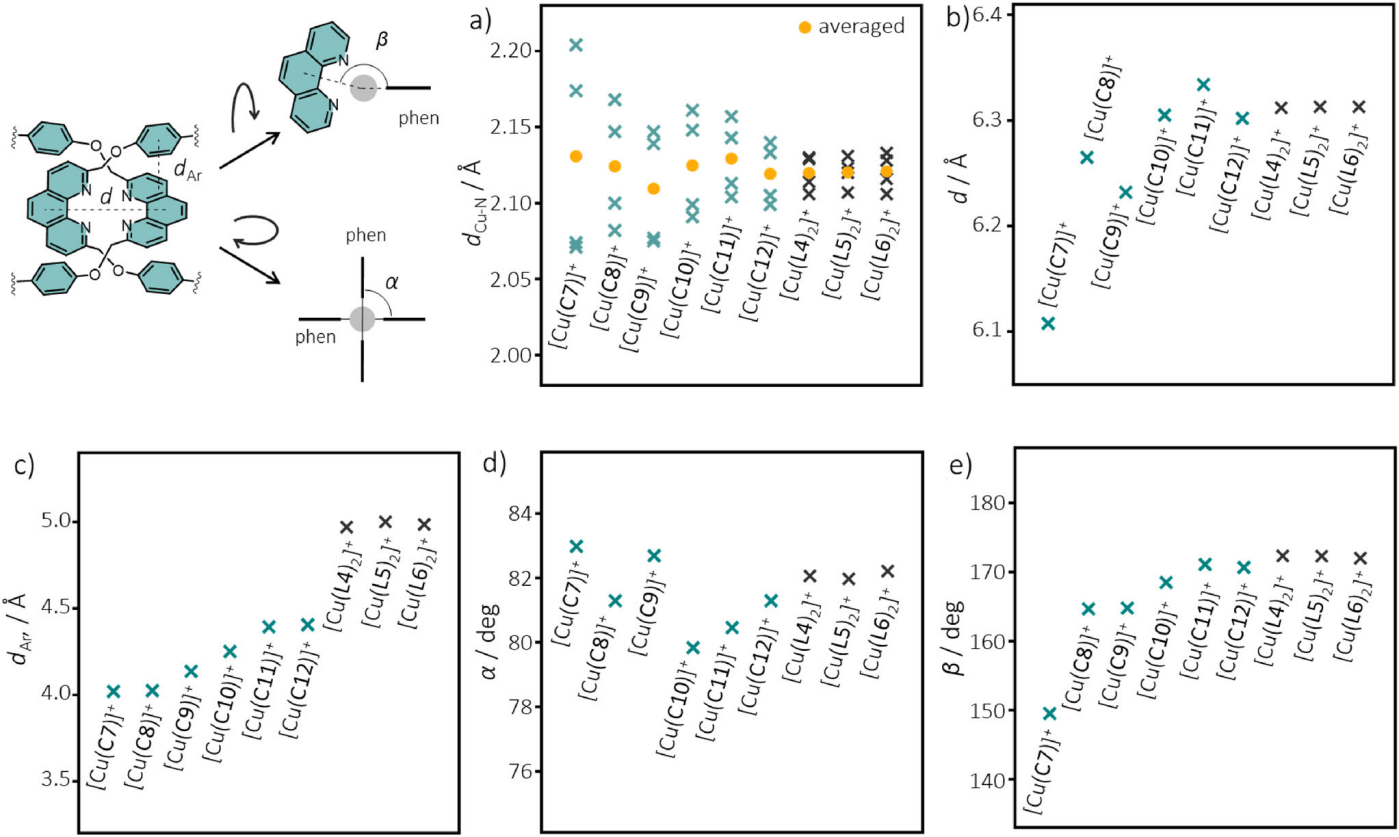
Selected structural parameters of the catenane and non‐interlocked complexes obtained from DFT simulations.

Although X‐ray crystal structures of these Cu^I^ catenanes may allow a direct comparison of the coordination structures, effects from crystal packing, crystal forms, solvents of re‐crystallization and other factors may mask the subtle structural differences in these catenane complexes.^[^ ^51^ ^]^ We thus turned our attention to DFT simulations and obtained energy‐minimized structures of [Cu(**Cn**)]^+^ (*n* = 7–12) and [Cu(**Ln**)_2_]^+^ (*n* = 4–6) using the B3LYP functional (Figures S30 and S31). To analyse the coordination structure, the Cu─N distances (*d*_Cu─N_), the distance between the two phenanthroline centroids (*d*), the distance between the phenyl and phenanthroline planes (*d*_Ar_), the dihedral angle between the two phenanthrolines (*α*), and the rotation angle (*β*) are compared (Figure 2a and Table S4). For a perfect tetrahedral geometry, *α* and *β* would be 90° and 180°, respectively.

Consistent to the observations in ^1^H NMR, no significant difference was found in the simulated structures of the non‐interlocked complexes. The Cu^I^ coordination is very close to an ideal tetrahedral geometry, with an almost orthogonal orientation of the phenanthroline planes (e.g., *α* = 82.1° for [Cu(**L4**)_2_]^+^) and only a slight rotation of the phenanthroline macrocycle (e.g., *β* = 172.4° for [Cu(**L4**)_2_]^+^). An averaged *d*_Cu─N_ of 2.120–2.121 Å was found, which is consistent to the Cu─N bond length observed in other reported Cu^I^ bis(phenanthroline) complexes.^[^ ^63^, ^64^, ^65^, ^66^ ^]^ Structures of these three non‐interlocked complexes are relatively symmetrical, and the four Cu─N distances are comparable with each other with a Δ*d*_Cu─N_ of 0.024–0.027 Å. The distance between the two phenanthrolines is also almost the same (i.e., *d* = 6.31 Å) in these three complexes. In contrast, the two phenanthrolines become closer in the catenane complexes of a reducing ring size (e.g., *d* = 6.30 Å for [Cu(**C12**)]^+^ versus *d* = 6.11 Å for [Cu(**C7**)]^+^). A gradual decrease in the interplanar distance between the phenanthroline and phenylene linker was also observed (e.g., *d*_Ar_ = 4.41 Å for [Cu(**C12**)]^+^ v.s *d*_Ar_ = 4.02 Å for [Cu(**C7**)]^+^), which are consistent to a tightening of the overall structure. Nevertheless, the averaged *d*_Cu─N_ (2.110–2.131 Å) and *α* (79.8°–83.0°) are very similar in these complexes, and as a result, the tetrahedral coordination is compromised with a significant decrease in *β* (e.g., 170.7° for [Cu(**C12**)]^+^ versus 149.5° for [Cu(**C7**)]^+^) and a larger Δ*d*_Cu─N_ (e.g., 0.041 Å for [Cu(**C12**)]^+^ versus 0.133 Å for [Cu(**C7**)]^+^). In short, these data suggest that while the non‐interlocked phenanthrolines could freely arrange around the Cu^I^ and optimize to the preferred tetrahedral coordination, restriction imposed by the ligand interlocking will compromise the preferred geometry and lead to a structural distortion in an increasing extent when the interlocked rings reduce in size.

### Photophysical and Electrochemical Properties

As a result of the structural distortion, the electronic structure, and hence photophysical and electrochemical properties of the Cu^I^ catenanes will also be modulated. In particular, the tetrahedral‐planar geometry reorganization during Cu^I/II^ conversion, which is fundamental to the redox and chemical reactivity of the metal, are expected to be sensitive to the tightness of the ligand interlocking. For example, the Cu^I^‐to‐phenanthroline MLCT absorption (S_0_→S_2,3_ transitions, ca. 455 nm) would be followed by a flattening distortion due to pseudo‐Jahn–Teller effects of the *d* ^9^ Cu^II^.^[^ ^67^, ^68^, ^69^, ^70^, ^71^, ^72^ ^]^ Indeed, comparing the steady state UV–Vis absorption spectra of the Cu^I^ catenanes, a gradual red‐shift of the MLCT absorption was observed (e.g., 450 nm for [Cu(**C12**)]^+^ versus 460 nm for [Cu(**C7**)]^+^), but that of the non‐interlocked [Cu(**Ln**)_2_]^+^ (*n* = 4–6) was all found to be of a slightly higher energy at 447 nm (Figure 1b). As the tetrahedral geometry of the *d* ^10^ Cu^I^ is distorted in an increasing extent when the catenane ring‐size decreases, energy gap between the ground (i.e., Cu^I^) and Franck–Condon excited state (i.e., Cu^II^) would also become smaller and resulting in a red shift of the MLCT absorption. On the other hand, similar *λ*_max_ at 463 nm was found for the MLCT absorption of [Cu(**C8**)]^+^, [Cu(**C8‐Ms**)]^+^, [Cu(**C8‐Ipps**)]^+^, and [Cu(**C8‐Tipps**)]^+^, further supporting that the energy of the MLCT transition is ring‐size dependent. Further time‐dependent density functional theory (TDDFT) studies also showed a red‐shift of the low energy absorption (e.g., 428 nm for [Cu(**C12**)]^+^ versus 451 nm for [Cu(**C7**)]^+^, Figure S32), which is fully consistent to the experimental absorption spectra. Excited state dynamics of the catenane complexes was probed by femtosecond transient absorption (fs‐TA) spectroscopy, and a 3‐component decay kinetics composed of a S_n_ to S_1_ internal conversion and an S_1_ pseudo Jahn‐Teller distortion of sub‐picosecond, an intersystem crossing to T_1_ with a time range of 5–10 ps, and a decay of T_1_ to S_0_ with a timescale of several hundred picoseconds was observed (Figures S37–S45).^[^ ^73^, ^74^, ^75^ ^]^ Except for the significantly longer τ_3_ of [Cu(**C7**)]^+^, which could be explained by the tightest interlocking and hence least favorable structural reorganization in the series, no other trend in these time constants was found, and more detail studies on the effects of ligand interlocking on the excited state dynamics is warranted.

Ring‐size of the catenane ligands is also found to impact on the Cu^II/I^ redox shuttling. Cyclic voltammetry (CV) study on [Cu(**Cn**)](PF_6_) (*n* = 7 to 10) in MeCN under N_2_ showed a quasi‐reversible wave at *E*_1/2_ of ∼0.7 V (versus NHE), whereas no redox wave was observed for the larger [Cu(**C11**)](PF_6_) and [Cu(**C12**)](PF_6_) (Figure S50). Due to the low solubility in MeCN, attempts to obtain cyclic voltammograms of the sulfonamide derivatives were unsuccessful. While the similar *E*_1/2_ values may suggest similar thermodynamics of the Cu^II/I^ redox cycling, the peak‐to‐peak separation (Δ*E*) between the oxidation and reduction waves of these catenane complexes was found to be different. In particular, the smallest Δ*E* of 500 mV of [Cu(**C9**)]^+^, when compared to the 570–600 mV of [Cu(**C7**)]^+^, [Cu(**C8**)]^+^ and [Cu(**C10**)]^+^, suggests the medium‐sized [Cu(**C9**)]^+^ has the fastest electron transfer kinetics among the series.^[^ ^51^ ^]^ While the tetrahedral‐to‐planar geometry reorganization would be impeded by a tight interlocking, the reorganization of the very large catenanes may also be less efficient because the movement will involve more atoms.

### Thermodynamics of Cu^I^ Coordination

In addition to the mechanical chelation, the structural distortion and reinforced secondary interactions due to the catenane ligands of varying ring‐size should also lead to a different thermodynamic stability of the Cu^I^ coordination. Stability constant (i.e., *β*) of the Cu^I^ catenanes was measured by competitive cyanide exchange using UV–Vis spectroscopy, and the data is shown in Table 1. A log *β* of ∼16 was found for these Cu^I^ catenane complexes, which are higher than those of the non‐interlocked counterparts by ∼4 order of magnitude, which can be ascribed to the mechanical chelation.^[^ ^76^, ^77^, ^78^, ^79^ ^]^ Of note, the high stability of the Cu^I^ catenanes is comparable to that of copper binding proteins in nature.^[^ ^80^, ^81^, ^82^, ^83^, ^84^, ^85^, ^86^ ^]^ An increase in the thermodynamic stability was found when the catenane ring‐size decreases from [Cu(**C12**)]^+^ (*β* = 6.1 × 10^15^ M^−1^) to [Cu(**C7**)]^+^ (*β* = 2.6 × 10^16^ M^−1^). Thermodynamic parameters were obtained from van't Hoff analysis by variable temperature UV‐Vis studies (Figure S51), and the increase in the stability of Cu^I^ catenanes of a smaller ring‐size is found to be enthalpy driven, which is consistent to the reinforced inter‐ring interactions observed in the ^1^H NMR structural analysis. On the other hand, the Cu^I^ coordination to a smaller catenane has a less favourable entropy, indicating a greater extent of the structural tightening extending to the periphery of the complex, and that the larger catenane complexes could retain a higher degree of flexibility after the coordination. Varying the steric bulkiness of the exocyclic substituents on the catenane was found to have no obvious effect on the thermodynamics of the Cu^I^ coordination, and a comparable stability was found for [Cu(**C8‐Ms**)]^+^ (*β* = 7.8 × 10^15^ M^−1^), [Cu(**C8‐Ipps**)]^+^ (*β* = 8.4 × 10^15^ M^−1^) and [Cu(**C8‐Tipps**)]^+^ (*β* = 8.5 × 10^15^ M^−1^).

**Table 1:** Thermodynamic parameters of the Cu^I^ catenane complexes at 298 K.

	*β* (M^−1^)	Δ*G* (kJ mol^−1^)	Δ*H* (kJ mol^−1^)	Δ*S* (J mol^−1^ K^−1^)
Varying ring‐size				
[Cu(**C12**)]^+^	6.1 (±1.3) × 10^15^	−90.0 (±0.6)	−146.5 (±9.2)	−187.8 (±29.0)
[Cu(**C11**)]^+^	1.0 (±0.2) × 10^16^	−91.2 (±0.4)	−148.3 (±7.7)	−188.2 (±24.4)
[Cu(**C10**)]^+^	1.2 (±0.4) × 10^16^	−91.5 (±0.8)	−148.9 (±8.0)	−191.6 (±25.5)
[Cu(**C9**)]^+^	1.7 (±0.3) × 10^16^	−92.5 (±0.5)	−150.3 (±7.8)	−194.1 (±24.7)
[Cu(**C8**)]^+^	1.9 (±0.5) × 10^16^	−92.8 (±0.7)	−153.2 (±8.4)	−202.1 (±26.6)
[Cu(**C7**)]^+^	2.6 (±0.4) × 10^16^	−93.6 (±0.4)	−158.3 (±7.5)	−213.9 (±23.9)

Varying exocyclic substituents				
[Cu(**C8‐Ms**)]^+^	7.8 (±0.7) × 10^15^	−90.7 (±0.2)	n.d.^a)^	n.d.^a)^
[Cu(**C8‐Ipps**)]^+^	8.4 (±0.9) × 10^15^	−90.8 (±0.3)	n.d.^a)^	n.d.^a)^
[Cu(**C8‐Tipps**)]^+^	8.5 (±0.5) × 10^15^	−90.9 (±0.1)	n.d.^a)^	n.d.^a)^

^a)^ n.d.: not determined.

### Kinetics of Cu^I^ Coordination

**Figure 3:**
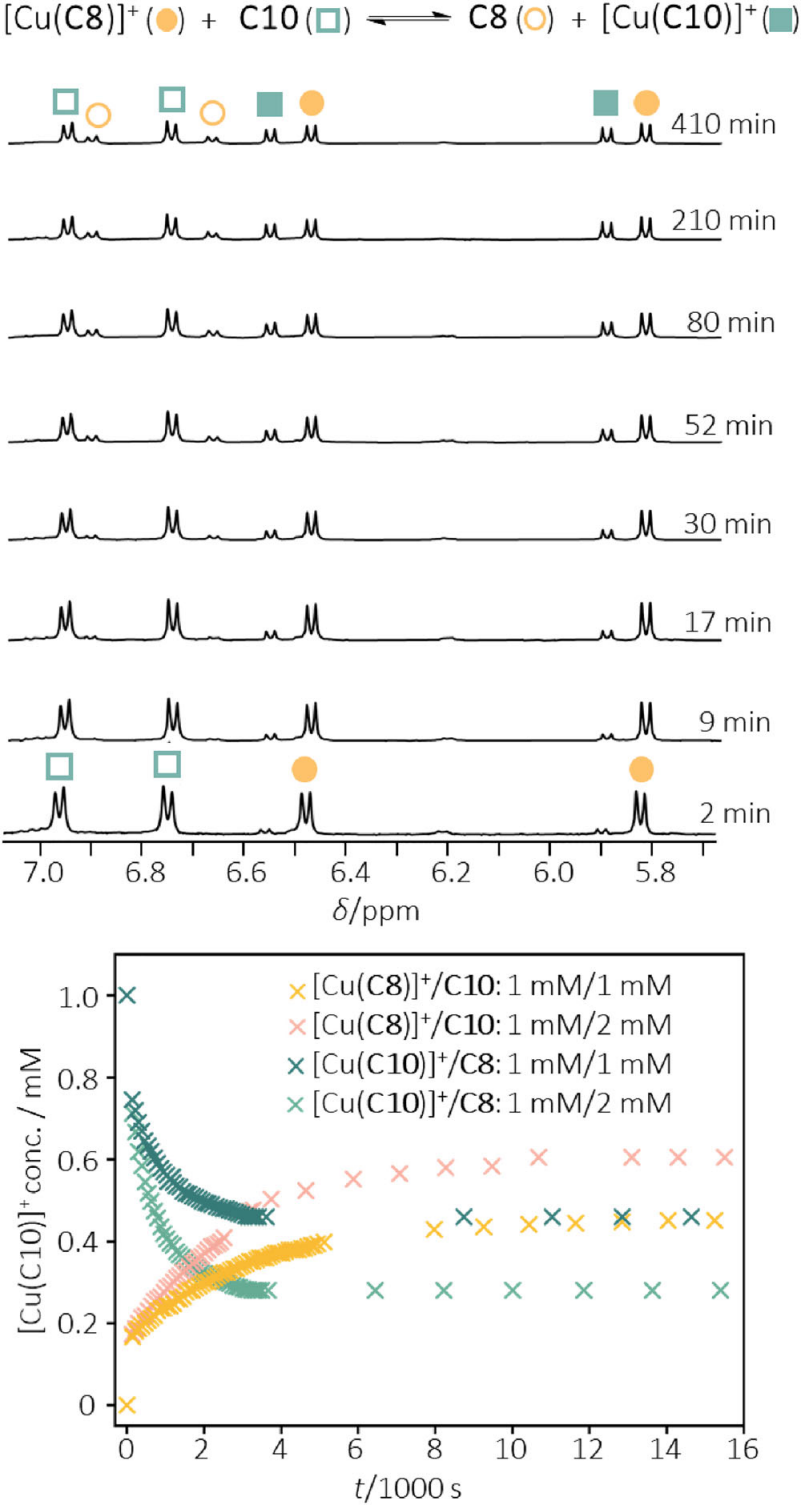
Kinetics of Cu^I^ ion scrambling between **C8** and **C10**, and the partial ^1^H NMR spectra at different time using an initial mixture containing an equimolar amount of [Cu(**C8**)]^+^ and **C10** (1 mM each).

**Figure 4:**
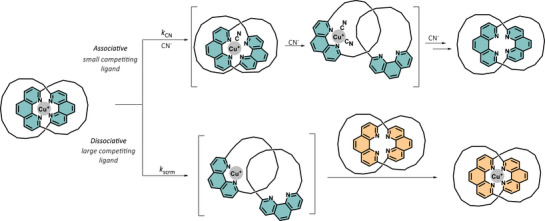
Proposed pathways for the associative and dissociative exchange of the Cu^I^ ion from the catenane complex to a small (e.g., cyanide) and a large (e.g., a catenane) competitive ligand, respectively. For both pathways, an initial, rate‐limiting ligand re‐organization is required to open the Cu^I^ coordination sphere, and thus hindering the catenane co‐conformational change, by either reducing the ring‐size or increasing the steric bulkiness of the exocyclic substituents, will result in a slower kinetics.

While the Cu^I^ binding is very strong and well‐shielded within the catenane coordination, the coordinatively labile ion was found to be able to hop between different catenanes. For example, ^1^H NMR study on an equimolar mixture of [Cu(**C8**)]^+^ and **C10** (1 mM each) in 1:1 (*v*/*v*) CDCl_3_/CD_3_CN showed the slow emergence of proton signals from free **C8** and [Cu(**C10**)]^+^ until equilibrium was reached after 4 h (Figure 3). Consistent to the *β*_C10_/*β*_C8_ ratio, a [Cu(**C10**)]^+^/[Cu(**C8**)]^+^ concentration ratio of ∼0.85 was found at equilibrium, and the same equilibrium mixture can also be attained by initiating the scrambling with [Cu(**C10**)]^+^ and **C8** in ∼1 h (Figure S61). For the hopping of Cu^I^ ion from **C8** to **C10**, since the parent complex is already coordinatively saturated, it would be improbable for the bidentate phenanthroline in the incoming **C10** to squeeze into the primary coordination sphere in [Cu(**C8**)]^+^. As a result, the scrambling would likely proceed via a dissociative mechanism involving a phenanthroline dissociation and co‐conformation change that reveals an empty coordination site at the Cu^I^ ion, with a space sufficiently large for accommodating the incoming **C10** (Figure 4). Such a co‐conformational change will therefore contribute to the activation barrier of the exchange, and hence the rate of the ligand scrambling (*k*_scrm_) would in turn indicate the ease of the catenane in revealing the Cu^I^ coordination sphere for a dissociative exchange. To obtain the kinetic data, changes in the concentration of the interlocked species over time were fitted to a kinetic model developed by Biedermann for describing host‐guest exchange with a rate‐limiting guest dissociation (Figure S54),^[^ ^87^ ^]^ and the obtained rate constants are summarized in Table 2. Consistent to the tighter interlocking with less facile co‐conformational movements, a smaller *k*_scrm_ was found for catenane complexes of a smaller ring‐size, and a difference of ∼30‐fold in *k*_scrm_ was found between the largest [Cu(**C12**)]^+^ and the smallest [Cu(**C7**)]^+^ in the series.

**Table 2:** Kinetic parameters of the Cu(I) catenane complexes at 298 K.

	*k*_scrm_ (10^−4^ s^−1^)	*k*_CN_ (M^−1^ s^−1^)
Varying ring‐size		
[Cu(**C12**)]^+^	22.7 (±0.4) (C′ = **C8**)^a)^	2700 (±60)
[Cu(**C11**)]^+^	14.2 (±0.2) (C′ = **C8**)	2280 (±45)
[Cu(**C10**)]^+^	12.2 (±0.2) (C′ = **C8**)	1950 (±30)
[Cu(**C9**)]^+^	5.70 (±0.03) (C′ = **C12**)	1680 (±20)
[Cu(**C8**)]^+^	3.30 (±0.04) (C′ = **C10**)	1420 (±10)
[Cu(**C7**)]^+^	0.80 (±0.1) (C′ = **C10**)	1080 (±40)

Varying exocyclic substituents		
[Cu(**C8‐Ms**)]^+^	3.56 (±0.04) (C′ = **C10**)	n.d.^b)^
[Cu(**C8‐Ipps**)]^+^	2.00 (±0.01) (C′ = **C10**)	n.d.^b)^
[Cu(**C8‐Tipps**)]^+^	1.82 (±0.01) (C′ = **C10**)	n.d.^b)^
[Cu(**C10**)]^+^	12.2 (±0.2) (C′ = **C8**)	
[Cu(**C10**)]^+^	12.5 (±0.4) (C′ = **C8‐Ms**)	
[Cu(**C10**)]^+^	12.3 (±0.1) (C′ = **C8‐Tipps**)	

^a)^ C′: free catenane used in the initial mixture.

^b)^ n.d.: not determined.

In addition to ring‐size and interlocking tightness, energy barrier of the co‐conformational change could also be controlled by varying the steric bulkiness of the exocyclic substituents as they would slip through each other during the move. In line with this, a decreasing trend in *k*_scrm_ of 3.56 × 10^−4^ s^−1^, 2.00 × 10^−4^ s^−1^, and 1.82 × 10^−4^ s^−1^ was found for [Cu(**C8‐Ms**)]^+^, [Cu(**C8‐Ipps**)]^+^ and [Cu(**C8‐Tipps**)]^+^ (Figure S68), respectively, with an increasing bulkiness of the exocyclic sulfonamides. On the other hand, for the scrambling initiated with [Cu(**C10**)]^+^ and free **C8‐Ms** or **C8‐Tipps**, a *k*_scrm_ of 1.25 × 10^−3^ s^−1^ and 1.23 × 10^−3^ s^−1^ was found, respectively (Figure S67), which is similar to that obtained from using the unsubstituted **C8** (*k*_scrm_ = 1.22 × 10^−3^ s^−1^) as the receiving ligand. The insignificant effect of the exocyclic substituents in the receiving catenane on *k*_scrm_ affirms that the initial dissociative opening of the coordination sphere of the parent complex is rate limiting. To put these kinetic effects into further perspective, [Cu(**C8‐Fmoc**)]^+^ was designed with the base‐labile, bulky Fmoc for a stimuli‐triggered switching of the Cu^I^ exchange dynamics. While the stability of [Cu(**C8‐Fmoc**)]^+^ (*β* = 3.3 × 10^16^ M^−1^) is comparable to the parent [Cu(**C8**)]^+^ and other sulfonamide analogues, scrambling experiment showed a *k*_scrm_ of 4.16 × 10^−5^ s^−1^ for [Cu(**C8‐Fmoc**)]^+^, which is an order of magnitude smaller than that of the parent [Cu(**C8**)]^+^, consistent to the slower opening of the Cu^I^ coordination sphere due to the bulky Fmoc moieties (Figure S74). After piperidine treatment that removed the Fmoc, a significant increase in the scrambling rate was resulted with an apparent *k*_scrm_ of 3.80 × 10^−4^ s^−1^, supporting that the Cu^I^ exchange dynamics has been successfully switched back to a faster rate upon stimulation by the base (Table 3).

**Table 3:** Comparison of *β* and *k*_scrm_ of [Cu(**C8‐Fmoc**)]^+^ and the parent [Cu(**C8**)]^+^ at 298 K.

	*β* (10^16^ M^−1^)	*k*_scrm_ (10^−4^ s^−1^)^a)^
[Cu(**C8**)]^+^	1.9 (±0.5)	3.30 (±0.04)
[Cu(**C8‐Fmoc**)]^+^	3.3 (±0.4)	0.42 (±0.1)
[Cu(**C8‐Fmoc**)]^+^ b)	/	3.80 (±0.08)

^a)^ free **C10** was used in the initial mixture.

^b)^ after piperidine treatment.

While a relatively large extent of the co‐conformational change is required for the Cu^I^ ion to exchange in a dissociative fashion, the required co‐conformational change would be of a smaller amplitude if the competing ligand is small enough to squeeze in and coordinate to the Cu^I^ in the parent complex. Indeed, the observed rate for the cyanide‐assisted Cu^I^ extraction from the catenane complexes is much faster, with the apparent rate constant (*k*_obs_) in the order of 10^3^ s^−1^ (Figure S52). The overall reaction rate is found to be first order with respect to cyanide (with a rate constant *k*_CN_), and contribution from self‐dissociation is insignificant.^[^ ^24^ ^]^ An associative exchange mechanism featuring an initial cyanide coordination as the rate‐limiting step is hence proposed (Figure 4). Consistent to our proposal, a decreasing trend in *k*_CN_ was observed for catenane complexes with a smaller ring‐size, with a ∼60% decrease from 2700 M^−1^s^−1^ for [Cu(**C12**)]^+^ to 1100 M^−1^s^−1^ for [Cu(**C7**)]^+^ (Table 2). Overall, these kinetic studies show that the Cu^I^ ion in the interlocked ligand remains labile and accessible to external ligands (or substrate in the case of catalysis) despite the coordination pocket is well‐shielded. Uniquely, co‐conformational flexibility of the catenane would allow the coordination sphere to open in an adjustable and dynamic fashion, depending on the steric need of the incoming ligand/substrate. As such, modifying the catenane structures to tune the ease of the co‐conformational change will be a novel way to control the chemical reactivity of the coordinated Cu^I^, which is fundamentally different to that of simple steric control with no adaptability in common ligand designs. In fact, stereoelectronic properties of the primary sphere in these catenane complexes are highly similar, and effect of the length of the peripheral aliphatic linkers on the steric shielding of the metal would be insignificant.^[^ ^24^ ^]^

### Chemical Reactivity

**Figure 5:**
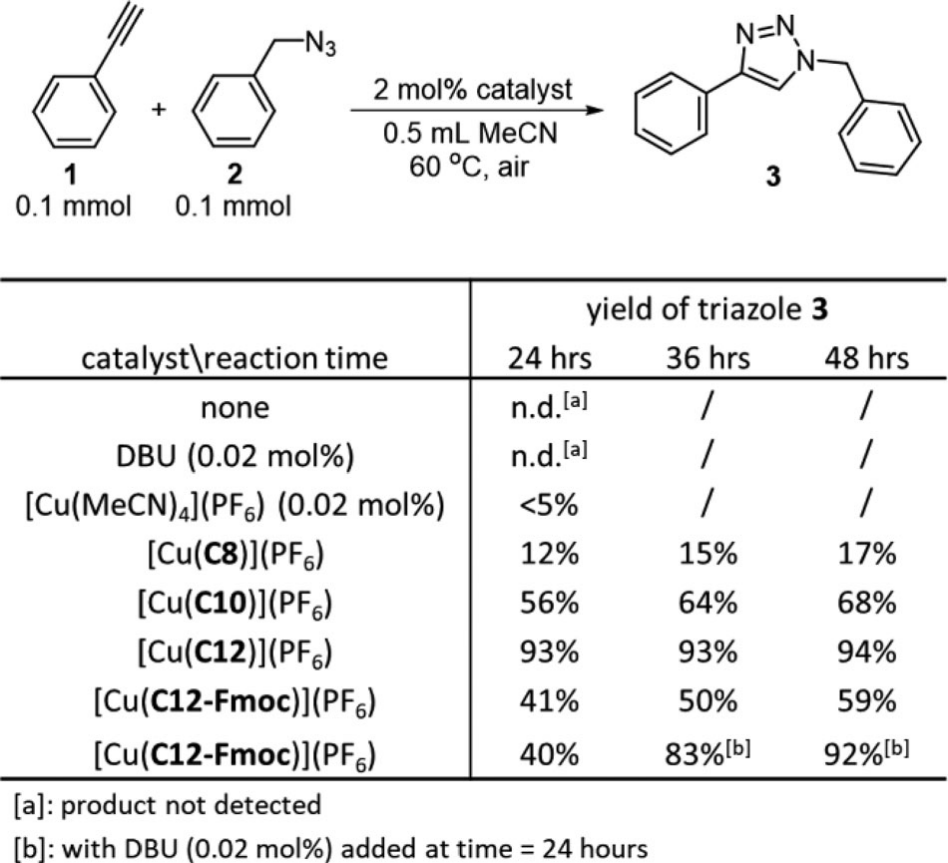
Activity of the Cu^I^ catenanes in the click reaction between phenylacetylene and benzylazide.

To put these understandings on the dynamic opening of the metal coordination sphere in the catenane complexes in further perspectives, activity of the Cu^I^ catenanes in mediating the cycloaddition of azide and alkyne (i.e., click reaction) was investigated. As both the azide and alkyne will need to coordinate to the Cu^I^,^[^ ^88^ ^]^ which will be dependent on the accessibility to the metal coordination sphere, the click reaction will be a good model reaction to correlate the Cu^I^ reactivity to the catenane co‐conformational flexibility. The click reaction between phenylacetylene and benzylazide (0.1 mmol each) in the presence of 2 mol% of a Cu^I^ catenane was tested (Figures 5 and S79). After 24 h of reaction, the click product was formed in only a low yield of 12% when [Cu(**C8**)](PF_6_) was used as the catalyst, suggesting that the coordination sphere is hardly accessible. This result is similar to Papot's work that the click activity of Cu^I^ is completely inhibited upon coordination to their catenanes.^[^ ^48^ ^]^ Loosening the interlocking can indeed boost the click activity by rendering a more facile access to the Cu^I^, and the reaction catalyzed by [Cu(**C10**)](PF_6_) and [Cu(**C12**)](PF_6_) resulted in a significant increase in the yield of the triazole product at 56% and 93%, respectively. Due to the good stability from the interlocked ligand, [Cu(**C12**)](PF_6_) can also be recovered with no observable decomposition, and no significant decrease in the catalytic efficiency was found after five rounds of catalysis (Figures S80).

On the other hand, the click reaction catalyzed by [Cu(**C12‐Fmoc**)](PF_6_) gave the triazole product in a much lower yield of 41% under the same condition, and this reduced activity is likely a combined result of the hindered opening of the coordination sphere and substrate blocking by the bulky Fmoc. Upon an in situ removal of the Fmoc by adding 0.02 mol% of the sterically hindered 1,8‐diazabicyclo[5.4.0]undec‐7‐ene (DBU), the click activity was efficiently restored and the triazole product was obtained in 92% yield after a further 24‐h reaction (Figure S85). Control experiments using 0.02 mol% of DBU or [Cu(MeCN)_4_](PF_6_) gave no click product, further confirming that the increase in the yield of **3** was due to the removal of the Fmoc that restored the ability of the catenane ligand in revealing the Cu^I^ coordination sphere. Of note, compare to the irreversible cleaving of the interlocked components to eliminate the catenand effect for switching on the catalytic activity,^[^ ^47^, ^48^ ^]^ our demonstration of modulating the co‐conformational dynamics of the catenane ligand as a control of the catalytic activity, while preserving the mechanical interlocking for further cycles of on–off switching, is unprecedented.

## Conclusion

While mechanical interlocking represents a different dimension of ligand design strategy that is independent from, and complementary to covalent modifications, exploiting ligand interlocking for new features and applications of transition metal complexes is severely impeded by our ability in relating the unique effects from ligand interlocking to specific structural elements of the interlocked ligands for rational design and control. Through a systematic analysis of the structures, spectroscopic, photophysical and redox properties, thermodynamics, kinetics, as well as chemical reactivity of a series of 4‐coordinate Cu^I^ catenane complexes, we showed for the first time that the degree of these ligand interlocking effects is directly associated with the co‐conformational flexibility of the interlocked ligand, and hence can be controlled by varying the catenane size and exocyclic substituents in a reliable and predictable fashion.

With these understandings, Cu^I^ complexes with removable groups on the catenane skeleton were designed as a new type of switchable MIMs with a tunable degree of catenand effect. In addition to the ligand exchange rate, an on–off switching of the activity of the Cu^I^ in a model click reaction while fully preserving the mechanical bond, was successfully achieved. Considering the diverse applications of transition metal complexes, and that the coordination chemistry of mechanically interlocked ligands is yet to be fully uncovered, our discovery and demonstration of controlling effects from ligand interlocking via rational MIM‐design will thus exhibit significant potential across multiple disciplines.

## Supporting Information

The authors have cited additional references within the Supporting Information.^[^ ^89^, ^90^, ^91^, ^92^, ^93^, ^94^, ^95^, ^96^, ^97^, ^98^, ^99^, ^100^, ^101^, ^102^, ^103^, ^104^, ^105^, ^106^, ^107^ ^]^

## Conflict of Interests

The authors declare no conflict of interest.

## Supporting information



Supporting Information

## Data Availability

The data that support the findings of this study are available in the Supporting Information of this article.
