# Supplementary material for: Structural Dependence of Catenand Effect: Thermodynamic and Kinetic Modulation of Catenane Coordination Properties via Ring‐size and Exocyclic Substituent Variation
Source: Angew Chem Int Ed Engl. 2025 Oct 6;64(48):e202514599. doi: 10.1002/anie.202514599 (PMC12643341; doi:10.1002/anie.202514599)
Supplement: Supplementary file 1 — Supporting Information [file ANIE-64-e202514599-s001.pdf]

Electronic Supplementary Information for

Structural Dependence of Catenand Effect: Thermodynamic and Kinetic Modulation of Catenane Coordination Properties via Ring-size and Exocyclic Substituent Variation

Yulin Deng,[1] Zi-Gang Lu,[1] Samuel Kin-Man Lai,[1] Man Pang Tang,[1] Xiaoyong Mo,[1,2,3] Shan He,[1] David Lee Phillips,[1] Edmund Chun Ming Tse[1,2,3] and Ho Yu Au-Yeung[1,2,4]*

[1]Department of Chemistry, The University of Hong Kong, Hong Kong, China
[2]CAS-HKU Joint Laboratory on New Materials, The University of Hong Kong, Hong Kong, China
[3]Laboratory for Synthetic Chemistry and Chemical Biology Limited, Units 1503-1511, Building 17W, Hong Kong Science Park, New Territories, Hong Kong, China
[4]State Key Laboratory of Synthetic Chemistry, The University of Hong Kong, Hong Kong, China

*Correspondence: hoyuay@hku.hk

1. Synthesis

General. All reagents were purchased from commercial suppliers (J&K, Sigma-Aldrich, TCI, Energy, Macklin and Bidepharm) and used without further purification unless otherwise noted. **Phen-CHO**, [Cu(**C7**)](PF_6), [Cu(**C8**)](PF_6), [Cu(**C9**)](PF_6), [Cu(**C10**)](PF_6), [Cu(**C11**)](PF_6), [Cu(**C12**)](PF_6), **C8**, **C10**, **C12** were synthesized according to literature procedures.[1-2] ESI-MS were carried out using a Waters-Acquity UPLC H-Class system coupled with a QDa MS detector. HR-ESI-MS were obtained from a Bruker Impact II Ultra-High Resolution QTOF mass spectrometer. NMR spectra were recorded on Bruker DPX spectrometers with working frequencies of 400 MHz, 500 MHz or 600 MHz for ^{1}H, and 101 MHz, 126 MHz or 150 MHz for ^{13}C, respectively. Chemical shifts are reported in ppm and referenced to solvent residues (For ^{1}H: DMSO-d_6: δ = 2.50 ppm; CD_3CN: δ = 1.94 ppm; $CDCl_3$: δ = 7.26 ppm; for ^{13}C: DMSO-d_6: δ = 39.52 ppm; CD_3CN: δ = 1.32 ppm; $CDCl_3$: δ = 77.16 ppm).

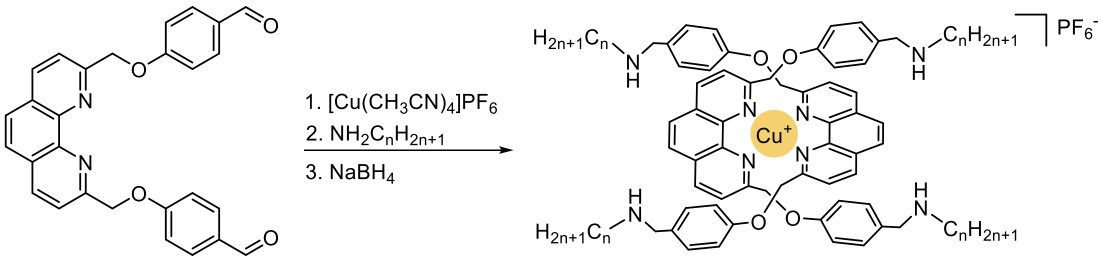

Phen-CHO

[Cu(**L4**)$_2$](PF_6): n = 4
[Cu(**L5**)$_2$](PF_6): n = 5
[Cu(**L6**)$_2$](PF_6): n = 6

General procedures for the synthesis of non-interlocked complexes. A mixture of **Phen-CHO** (0.45 g, 1.0 mmol) and [Cu(CH_3CN)$_4$](PF_6) (0.19 g, 0.5 mmol) in 100 mL of 5:3:2 $CHCl_3$/CH_3CN/CH_3OH (50 mL/30 mL/20 mL) was stirred under argon until a clear red solution was obtained, followed by addition of the alkyl amine (3.0 mmol) and piperidine (0.5 mL), and the mixture was heated to 60 °C for overnight. The reaction mixture was cooled in an ice bath, $NaBH_4$ (0.10 g, 2.5 mmol) was added, and the mixture was stirred in the ice bath for 1 h. After warming the reaction mixture to room temperature, solvents were removed by a rotary evaporator. The dark red residue was re-dissolved in 50 mL CH_2Cl_2, washed with water (2 × 50 mL) and brine (50 mL), dried over anhydrous Na_2SO_4 and filtered. Solvents were removed from the filtrate by a rotary evaporator to afford a red solid, which was washed with diethyl ether (3 × 20 mL) and dried under vacuum.

*Synthesis of [Cu(**L4**)$_2$](PF$_6$)*. Red powder. Yield: 0.61 g, 85%. ^{1}H NMR (400 MHz, DMSO-d_6, 298 K) δ 8.75 (d, J = 8.2 Hz, 4H), 8.10 (d, J = 8.1 Hz, 8H), 6.69 (d, J = 8.1 Hz, 8H), 6.05 (d, J = 8.3 Hz, 8H), 4.94 (s, 8H), 3.40 (s, 8H), 2.35 (br, 8H), 1.38–1.33 (m, 8H), 1.30–1.24 (m, 8H), 0.84 (t, J = 7.2 Hz, 12H). ^{13}C NMR (101 MHz, DMSO-d_6, 298 K) δ 155.8, 155.2, 142.4, 138.2, 132.7, 128.5, 128.4, 126.6, 124.5, 112.9, 70.3, 52.2, 48.2, 31.7, 20.0, 14.0. HRMS (ESI+): m/z calcd. for $C_{72}H_{84}N_8O_4CuPF_6$ [M-PF$_6$]$^+$: 1187.5906, found: 1187.5848.

*Synthesis of [Cu(**L5**)$_2$](PF$_6$)*. Red powder. Yield: 0.59 g, 79%. ^{1}H NMR (400 MHz, DMSO-d_6, 298 K) δ 8.74 (d, J = 8.2 Hz, 4H), 8.10 (d, J = 8.0 Hz, 8H), 6.69 (d, J = 7.4 Hz, 8H), 6.05 (d, J = 7.9 Hz, 8H), 4.94 (s, 8H), 2.36 (br, 8H), 1.38 (br, 8H), 1.24 (br, 16H), 0.85 (t, J = 6.6 Hz, 12H). ^{13}C{^{1}H} NMR (101 MHz, DMSO-d_6, 298 K) δ 156.5, 155.9, 155.1, 142.4, 138.2, 129.7, 128.5, 126.6, 124.6, 115.0, 112.9, 70.3, 29.0, 22.1, 22.0, 14.0, 13.9. HRMS (ESI+): m/z calcd. for $C_{76}H_{92}N_8O_4Cu$ PF$_6$ [M-PF$_6$]$^+$: 1243.6538, found: 1243.6564.

*Synthesis of [Cu(**L6**)$_2$](PF$_6$)*. Red powder. Yield: 0.63 g, 82%. ^{1}H NMR (400 MHz, DMSO-d_6, 298 K) δ 8.74 (d, J = 8.1 Hz, 4H), 8.10 (d, J = 8.3 Hz, 8H), 6.68 (d, J = 7.5 Hz, 8H), 6.06 (d, J = 7.5 Hz, 8H), 4.94 (s, 8H), 3.39 (s, 8H), 2.35 (br, 8H), 1.36 (br, 8H), 1.28–1.19 (m, 24H), 0.84 (t, J = 6.7 Hz, 12H). ^{13}C NMR (101 MHz, DMSO-d_6, 298 K) δ 155.8, 155.2, 142.4, 138.8, 138.2, 128.4, 127.9, 124.5, 124.3, 112.9, 70.3, 31.3, 26.5, 22.1, 14.0. HRMS (ESI+): m/z calcd. for $C_{80}H_{100}N_8O_4CuPF_6$ [M-PF$_6$]$^+$: 1299.7158, found: 1299.7083.

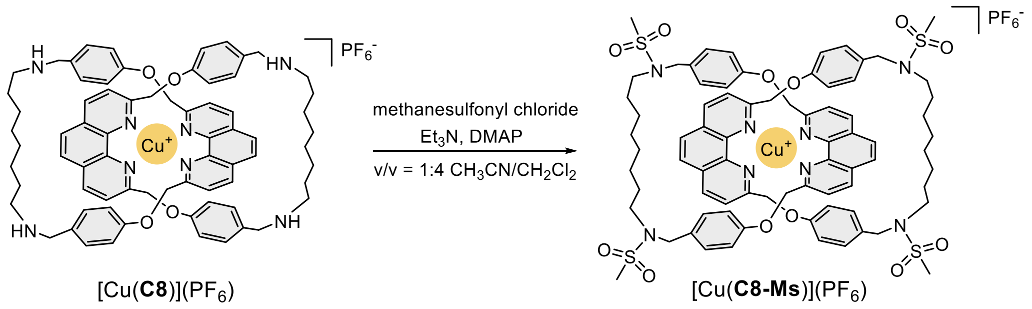

*Synthesis of [Cu(**C8-Ms**)](PF$_6$)*. To a solution of [Cu(**C8**)](PF$_6$) (35 μmol, 47 mg), triethylamine (0.35 mmol, 50 μL) and 4-dimethylaminopyridine (0.35 mmol, 43 mg) in 5 mL of 1:4 CH_3CN/CH_2Cl_2 (1 mL/4 mL) at 0 °C, a solution of methanesulfonyl chloride (0.35 mmol, 28 μL) in CH_2Cl_2 (3 mL) was added dropwise. The mixture was recovered to room temperature and stirred overnight. The obtained precipitation was collected by centrifugation at 4000 rpm for 3 minutes, washed with CH_3CN (2 × 2 mL) and CH_2Cl_2 (2

mL), and dried under vacuum. [Cu(**C8-Ms**)](PF_6) was obtained as an orange solid. Yield: 37 mg, 65%. ^{1}H NMR (500 MHz, DMSO-d_6, 298 K) δ 8.69 (d, J = 8.1 Hz, 4H), 8.11 (d, J = 8.2 Hz, 4H), 8.06 (s, 4H), 6.57 (d, J = 8.7 Hz, 8H), 5.86 (d, J = 8.6 Hz, 8H), 4.85 (s, 8H), 4.04 (s, 8H), 2.99 (t, J = 8.0 Hz, 8H), 2.86 (s, 12H), 1.57 (br, 8H), 1.34 (br, 8H), 1.25 (br, 8H). ^{13}C NMR (126 MHz, DMSO-d_6, 298 K) δ 156.2, 154.4, 142.5, 137.7, 129.6, 128.4, 127.6, 126.5, 125.3, 113.1, 70.5, 49.3, 47.6, 38.2, 28.2, 27.5, 26.2. HRMS (ESI+): m/z calcd. for $C_{76}H_{88}N_8O_{12}S_4CuPF_6$ [M-PF_6]$^+$: 1497.4704, found: 1497.4694.

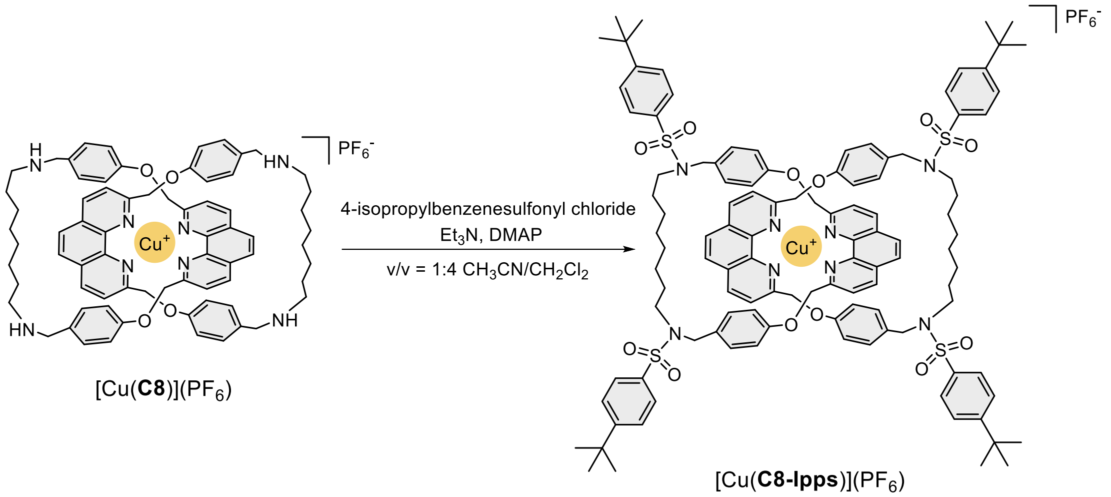

*Synthesis of [Cu(**C8-Ipps**)](PF_6).* To a solution of [Cu(**C8**)](PF_6) (35 μmol, 47 mg), triethylamine (0.35 mmol, 50 μL) and 4-dimethylaminopyridine (0.35 mmol, 43 mg) in 5 mL of 1:4 CH_3CN/CH_2Cl_2 (1 mL/4 mL) at 0 °C, a solution of 4-isopropylbenzenesulfonyl chloride (0.35 mmol, 77 mg) in CH_2Cl_2 (3 mL) was added dropwise. The mixture was recovered to room temperature and stirred overnight. The reaction mixture was diluted with CH_2Cl_2 (20 mL), washed with $NaHCO_3$ (aq., sat., 3 × 10 mL), dried over anhydrous Na_2SO_4 and filtered. Solvents were removed by a rotary evaporator, the afforded crude residue was purified by column chromatography using CH_2Cl_2/MeOH (v/v = 50/1) as the eluent. Yield: 57 mg, 79%. ^{1}H NMR (500 MHz, DMSO-d_6, 298 K) δ 8.68 (d, J = 8.2 Hz, 4H), 8.06 (t, J = 4.1 Hz, 8H), 7.68 (d, J = 8.3 Hz, 8H), 7.43 (d, J = 8.3 Hz, 8H), 6.48 (d, J = 8.4 Hz, 8H), 5.79 (d, J = 8.5 Hz, 8H), 4.80 (s, 8H), 4.02 (s, 8H), 3.01–2.92 (m, 12H), 1.35 (br, 8H), 1.25–1.22 (m, 8H), 1.20 (d, J = 6.9 Hz, 24H), 1.13 (br, 8H). ^{13}C NMR (126 MHz, DMSO-d_6, 298 K) δ 156.2, 154.4, 153.6, 142.5, 137.7, 137.0, 129.3, 128.4, 127.6, 127.3, 127.0, 126.5, 125.4, 113.1, 70.4, 49.7, 48.0, 33.3, 28.1, 27.4, 26.0, 23.8, 23.5. HRMS (ESI+): m/z calcd. for $C_{108}H_{120}N_8O_{12}S_4CuPF_6$ [M-PF_6]$^+$: 1913.7223, found: 1913.7349.

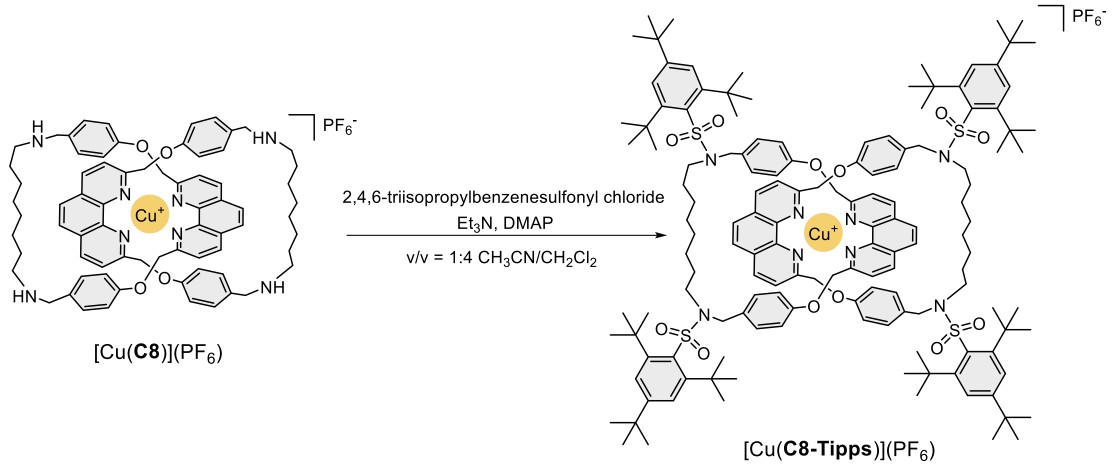

*Synthesis of [Cu(**C8-Tipps**)](PF$_6$)*. To a solution of [Cu(**C8**)](PF$_6$) (35 µmol, 47 mg), triethylamine (0.35 mmol, 50 µL) and 4-dimethylaminopyridine (0.35 mmol, 43 mg) in 5 mL of 1:4 CH_3CN/CH_2Cl_2 (1 mL/4 mL) at 0 °C, a solution of 2,4,6-triisopropylbenzenesulfonyl chloride (0.35 mmol, 109 mg) in CH_2Cl_2 (3 mL) was added dropwise. The mixture was recovered to room temperature and stirred overnight. The reaction mixture was diluted with CH_2Cl_2 (10 mL), washed with $NaHCO_3$ (aq., sat., 3 × 10 mL), dried over anhydrous Na_2SO_4 and filtered. Solvents were removed by a rotary evaporator, the afforded crude residue was purified by column chromatography using CH_2Cl_2/MeOH (v/v = 50/1) as the eluent. Yield: 77 mg, 92%. ^{1}H NMR (500 MHz, DMSO-d_6, 298 K) δ 8.72 (d, J = 8.2 Hz, 4H), 8.10 (d, J = 8.2 Hz, 4H), 8.06 (s, 4H), 7.20 (s, 8H), 6.47 (d, J = 8.3 Hz, 8H), 5.78 (d, J = 8.3 Hz, 8H), 4.80 (s, 8H), 4.11 (s, 8H), 3.94 (septet, J = 6.5 Hz, 8H), 2.93–2.87 (m, 4H), 2.85 (br, 8H), 1.38 (br, 8H), 1.23 (br, 8H), 1.17 (d, J = 7.0 Hz, 24H), 1.11 (d, J = 6.7 Hz, 48H), 1.01 (br, 8H). ^{13}C NMR (126 MHz, DMSO-d_6, 298 K) δ 156.2, 154.6, 152.9, 150.4, 142.5, 137.9, 131.9, 128.5, 128.5, 128.1, 126.5, 125.1, 123.8, 113.3, 79.2, 70.4, 47.3, 45.3, 33.3, 28.8, 28.1, 26.5, 26.1, 24.5, 23.3. HRMS (ESI+): m/z calcd. for $C_{132}H_{168}N_8O_{12}S_4CuPF_6$ [M+H-PF$_6$]$^{2+}$: 1125.5530, found: 1125.5517.

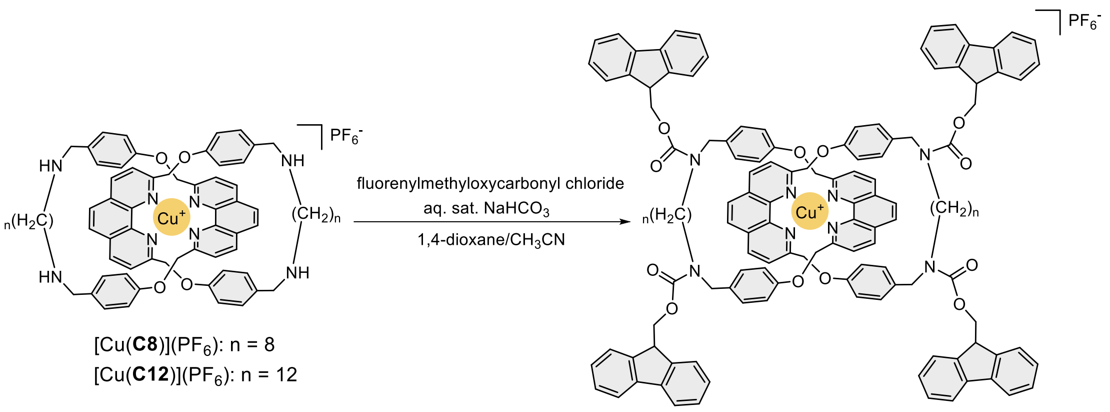

[Cu(**C8**)](PF$_6$): n = 8
[Cu(**C12**)](PF$_6$): n = 12

[Cu(**C8-Fmoc**)](PF$_6$): n = 8
[Cu(**C12-Fmoc**)](PF$_6$): n = 12

*Synthesis of [Cu(**C8-Fmoc**)](PF$_6$)*. To a solution of [Cu(**C8**)](PF$_6$) (75 µmol, 100 mg) in 13 mL of 10:3 1,4-dioxane/MeCN (10 mL/3 mL) at 0 °C, fluorenylmethyloxycarbonyl chloride (0.6 mmol, 155 mg) was added in three batches. An aqueous solution of $NaHCO_3$ (sat., 1.5 mL) was then added to the reaction mixture. The mixture was recovered to room temperature and stirred for 24 hours. Solvents were removed by a rotary evaporator, the red reaction residue was re-dissolved in 10 mL CH_2Cl_2, washed with water (2 × 5 mL) and brine (5 mL), dried over anhydrous Na_2SO_4 and filtered. The organic layer was collected, and the solvents were removed by a rotary evaporator, the afforded crude residue was purified by flash column chromatography using CH_2Cl_2/MeOH (v/v = 100/2) as the eluent. Yield: 128 mg, 77%. ^{1}H NMR (500 MHz, CD_3CN, 298 K) δ 8.35 (br, 4H), 7.90 (br, 8H), 7.73–7.55 (m, 12H), 7.45–7.27 (m, 16H), 7.13 (br, 4H), 6.19 (br, 8H), 5.78 (br, 8H), 4.79 (br, 8H), 4.65 (br, 4H), 4.41–4.06 (m, 8H), 4.02–3.85 (m, 8H), 3.07 (br, 4H), 2.58 (br, 4H), 1.54 (br, 4H), 1.37 (br, 8H), 1.23–1.03 (m, 12H). ^{13}C NMR (126 MHz, CD_3CN, 298 K) δ 156.4, 155.9, 154.5, 144.5, 144.2 143.0, 141.5, 141.2, 137.4, 130.4, 128.5, 127.6, 127.1, 126.4, 125.4, 124.7, 112.0, 113.1, 72.4, 71.0, 66.3, 65.6, 48.8, 47.6, 47.2, 46.8, 28.5, 28.3, 27.2, 27.1, 26.5. HRMS (ESI+): m/z calcd. for $C_{132}H_{120}N_8O_{12}CuPF_6$ [M-PF$_6$]$^+$: 2073.8357, found: 2073.8352.

*Synthesis of [Cu(**C12-Fmoc**)](PF$_6$)*. To a solution of [Cu(**C12**)](PF$_6$) (55 µmol, 80 mg) in 10 mL of 3:1 1,4-dioxane/MeCN (7.5 mL/2.5 mL) at 0 °C, fluorenylmethyloxycarbonyl chloride (0.44 mmol, 114 mg) was added in three batches. An aqueous solution of $NaHCO_3$ (sat., 1.2 mL) was then added to the reaction mixture. The mixture was recovered to room temperature and stirred for 24 hours. Solvents were removed by a rotary evaporator, the red reaction residue was re-dissolved in 10 mL CH_2Cl_2, washed with water (2 × 5 mL) and brine (5 mL), dried over anhydrous Na_2SO_4 and filtered. The organic layer was collected, and the solvents were removed by a rotary evaporator,

the afforded crude residue was purified by flash column chromatography using CH_2Cl_2/MeOH (v/v = 50/1) as the eluent. Yield: 83 mg, 64%. ^1H NMR (500 MHz, CD_3CN, 298 K) δ 8.35 (br, 4H), 7.94–7.79 (m, 8H), 7.76–7.57 (m, 12H), 7.44–7.27 (m, 16H), 7.18 (br, 4H), 6.39 (br, 4H), 6.14 (br, 4H), 5.97–5.80 (br, 8H), 4.86 (s, 8H), 4.63 (br, 4H), 4.50 (br, 4H), 4.27–4.12 (br, 4H), 4.00 (br, 4H), 3.78 (br, 4H), 3.03 (br, 4H), 2.61 (br, 4H), 1.45–1.30 (m, 24H), 1.21–0.99 (m, 16H). ^{13}C NMR (126 MHz, CD_3CN, 298 K) δ 157.5, 156.9, 156.6, 155.9, 145.5, 145.3, 143.9, 142.5, 142.3, 138.7, 129.6, 129.0, 128.6, 128.1, 127.4, 126.1, 125.7, 120.9, 72.0, 67.1, 66.8, 50.1, 49.4, 48.5, 48.3, 47.5, 30.2, 29.5, 28.7, 28.0, 27.5, 27.3. HRMS (ESI+): m/z calcd. for $C_{140}H_{136}N_8O_{12}CuPF_6$ $[M-PF_6+H]^{2+}$: 1093.4842, found: 1093.4839.

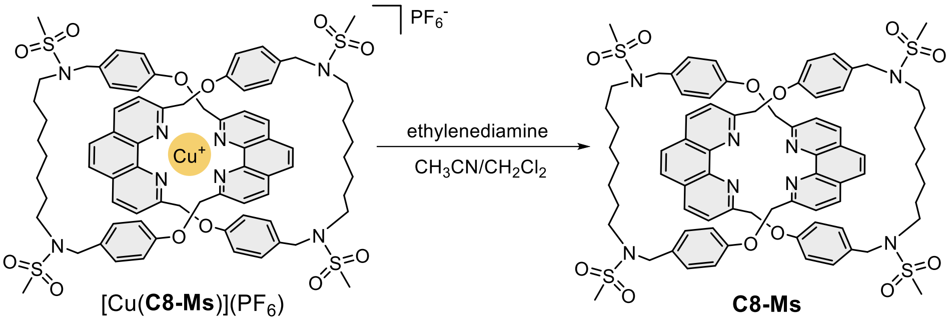

Synthesis of ***C8-Ms***. To a solution of [Cu(**C8-Ms**)](PF_6) (20 μmol, 33 mg) in 20 mL of 1: MeCN/CH_2Cl_2 (10 mL/10mL), ethylenediamine (0.5 mL) was added. The solution was stirred at room temperature for 15 minutes, and washed with H_2O (3 × 20 mL). Solvents were removed by a rotary evaporator. **C8-Ms** was obtained as a white solid. Yield: 27 mg, 94%. ^1H NMR (500 MHz, $CDCl_3$, 298 K) δ 8.12 (d, J = 8.1 Hz, 4H), 7.79 (d, J = 8.2 Hz, 4H), 7.63 (s, 4H), 6.95 (d, J = 8.5 Hz, 8H), 6.71 (d, J = 8.7 Hz, 8H), 5.27 (s, 8H), 4.09 (s, 8H), 2.81 (t, J = 8.3 Hz, 8H), 2.70 (s, 12H), 1.33 (br, 8H), 0.97 (br, 8H), 0.83 (br, 8H). ^{13}C NMR (126 MHz, $CDCl_3$, 298 K) δ 158.3, 157.4, 145.4, 137.1, 129.7, 128.7, 128.4, 126.6, 122.5, 115.4, 72.7, 50.5, 47.2, 39.0, 29.5, 28.3, 27.0. HRMS (ESI+): m/z calcd. for $C_{76}H_{88}N_8O_{12}S_4$ $[M+H]^+$: 1433.5477, found: 1433.5470.

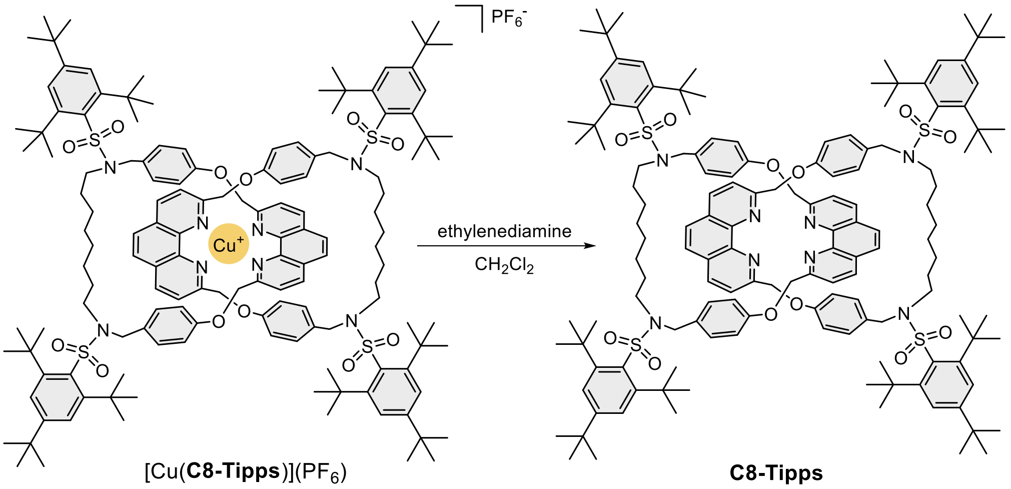

*Synthesis of **C8-Tipps***. To a solution of [Cu(**C8-Tipps**)](PF_6) (20 μmol, 48 mg) in CH_2Cl_2 (5 mL), ethylenediamine (0.5 mL) was added. The solution was stirred at room temperature for 5 minutes, and washed with H_2O (3 × 10 mL). Solvents were removed by a rotary evaporator. The crude mixture was re-dissolved in ethyl acetate (10 mL), and the products were recrystallized by slow evaporation of the solvent. The solvent residue was decanted, the crystals were collected, washed with Et_2O (2 × 5 mL), and dried under vacuum. **C8-Tipps** was obtained as a white crystal. Yield: 25 mg, 56%. ^{1}H NMR (600 MHz, $CDCl_3$, 298 K) δ 7.79 (d, *J* = 7.5 Hz, 4H), 7.23 (s, 8H), 7.17 (d, *J* = 8.7 Hz, 8H), 7.14 (s, 8H), 7.01 (d, *J* = 8.4 Hz, 8H), 5.13 (s, 8H), 4.33 (s, 8H), 4.13 (septet, *J* = 6.7 Hz, 8H), 2.91–2.88 (m, 4H), 2.84 (t, *J* = 7.7 Hz, 8H), 1.29 (br, 8H), 1.25 (d, *J* = 6.9 Hz, 24H), 1.22 (br, 48H), 0.91 (br, 8H), 0.78 (br, 8H). ^{13}C NMR (126 MHz, $CDCl_3$, 298 K) δ 158.9, 157.5, 153.0, 151.3, 144.9, 136.4, 132.3, 130.6, 128.4, 127.5, 125.7, 124.0, 121.0, 116.2, 72.8, 48.6, 44.8, 34.3, 30.0, 29.9, 29.5, 27.3, 27.2, 25.0, 23.7. HRMS (ESI+): m/z calcd. for $C_{132}H_{168}N_8O_{12}S_4$ $[M+2H]^{2+}$: 1094.0921, found: 1094.0920.

2. NMR Spectra

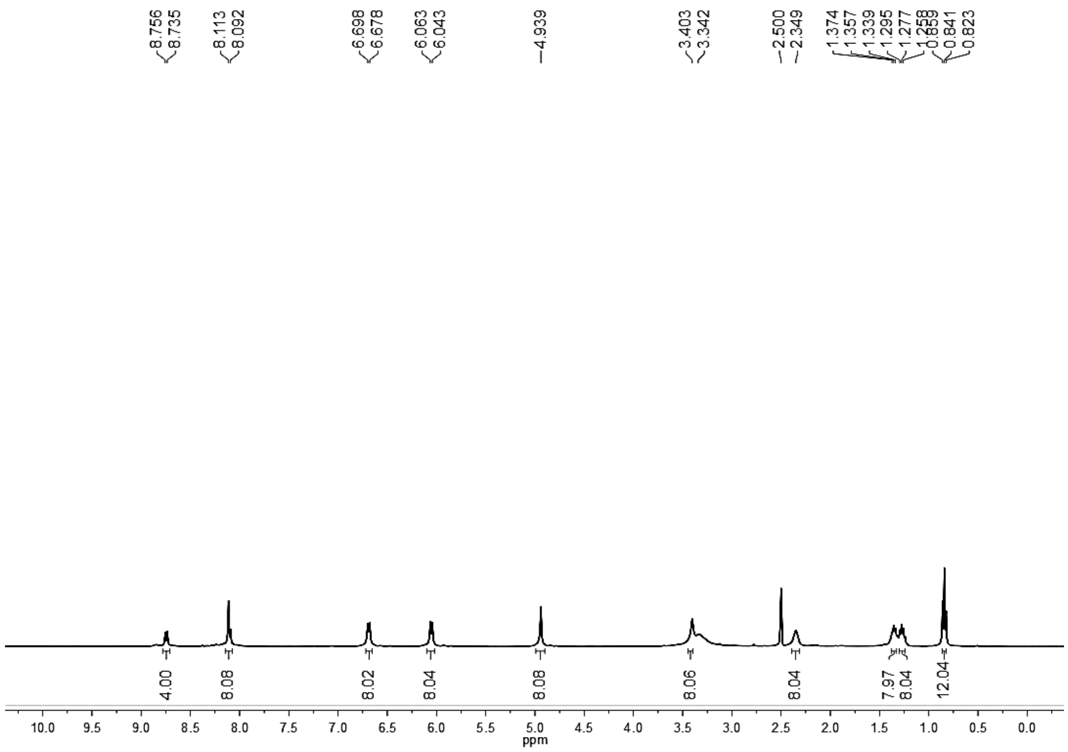

Figure S1. ^{1}H NMR (400 MHz, DMSO-d_6, 298 K) of [Cu(**L4**)$_2$](PF$_6$).

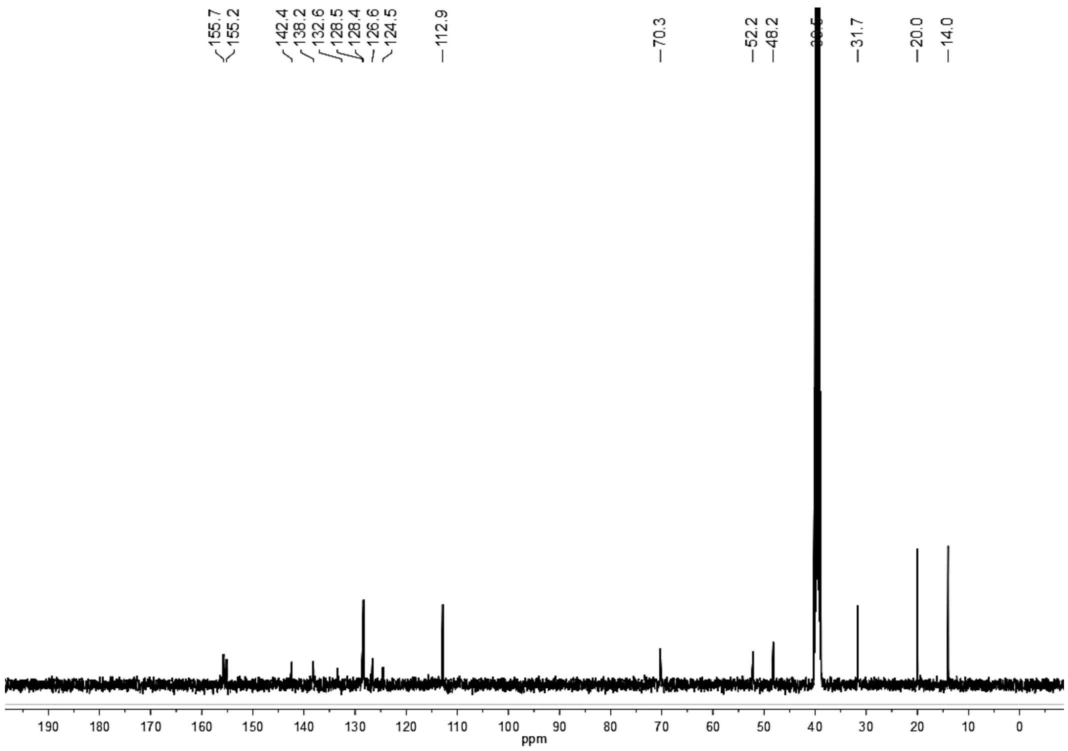

Figure S2. $^{13}C\{^{1}H\}$ NMR (101 MHz, DMSO-d_6, 298 K) of [Cu(**L4**)$_2$](PF$_6$).

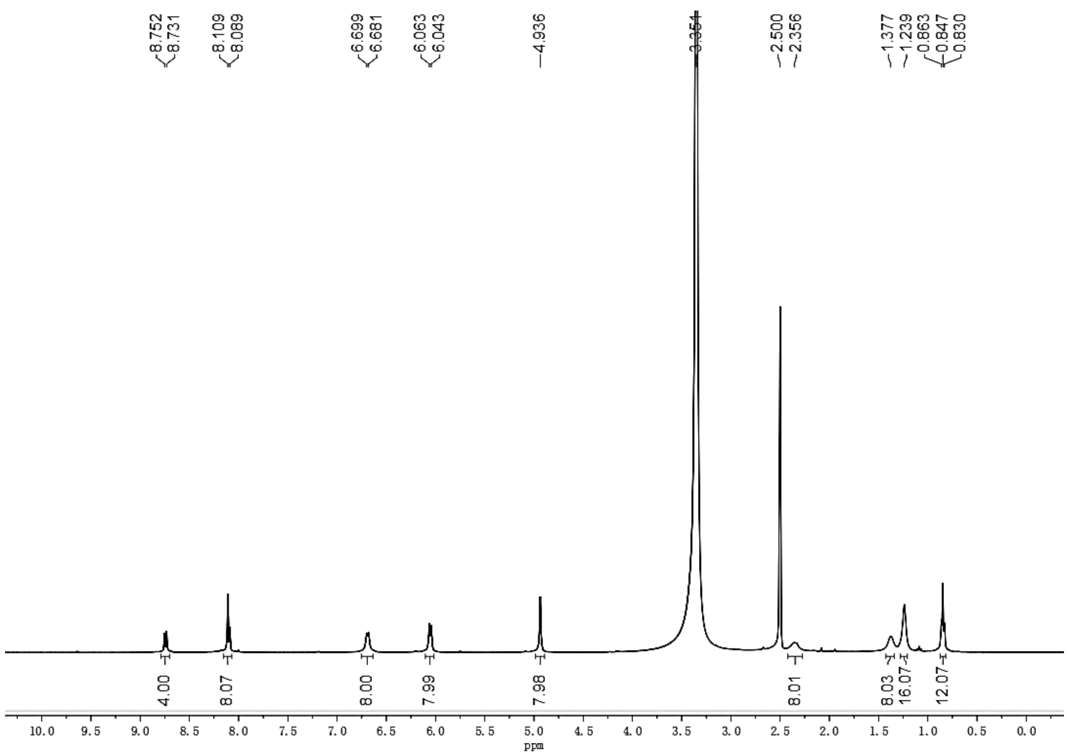

Figure S3. ^{1}H NMR (400 MHz, DMSO-d_6, 298 K) of [Cu(**L5**)$_2$](PF$_6$).

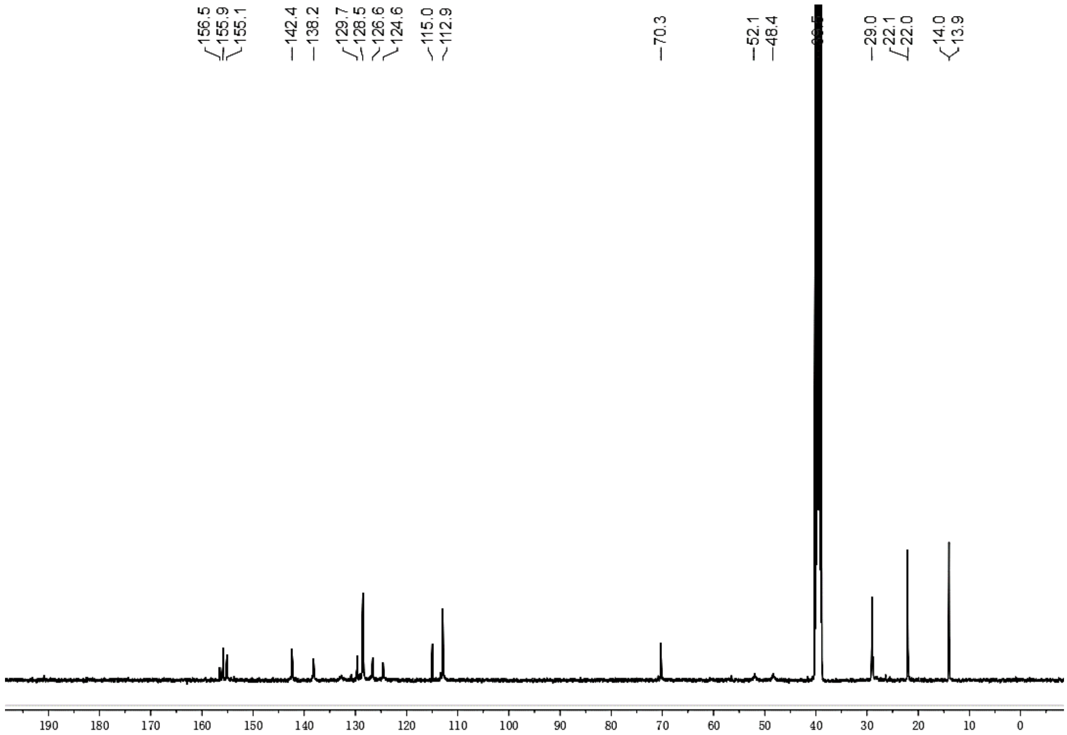

Figure S4. $^{13}C\{^{1}H\}$ NMR (101 MHz, DMSO-d_6, 298 K) of [Cu(**L5**)$_2$](PF$_6$).

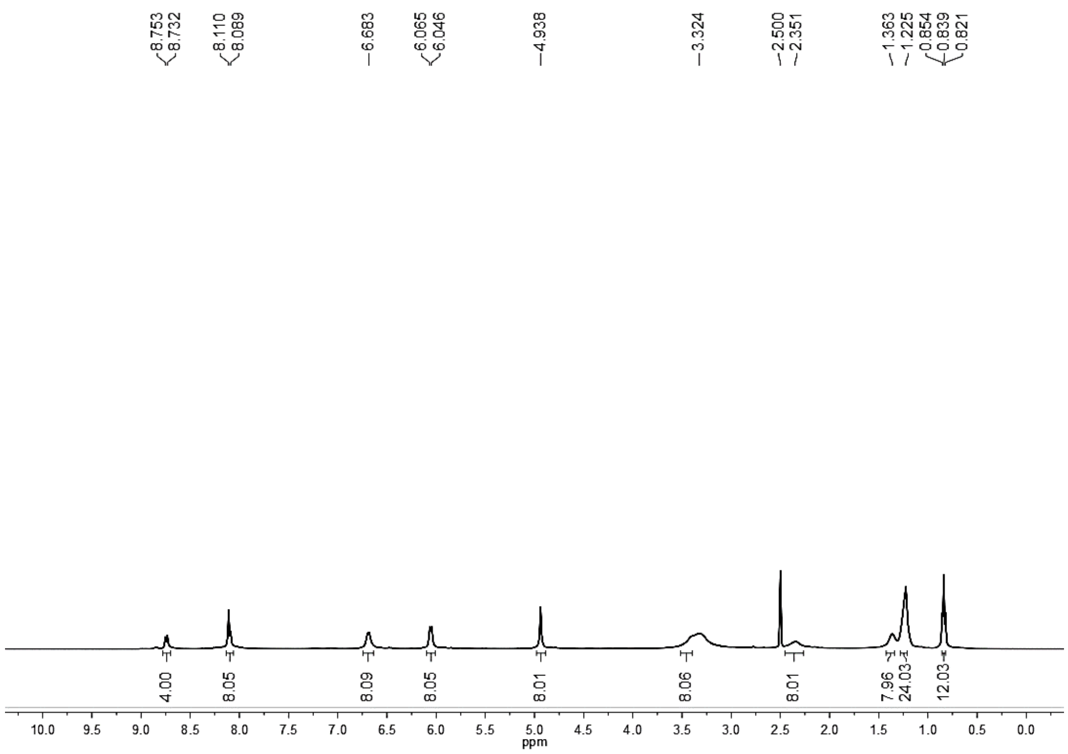

Figure S5. ^{1}H NMR (400 MHz, DMSO-d_6, 298 K) of $[Cu(\mathbf{L6})_2](PF_6)$.

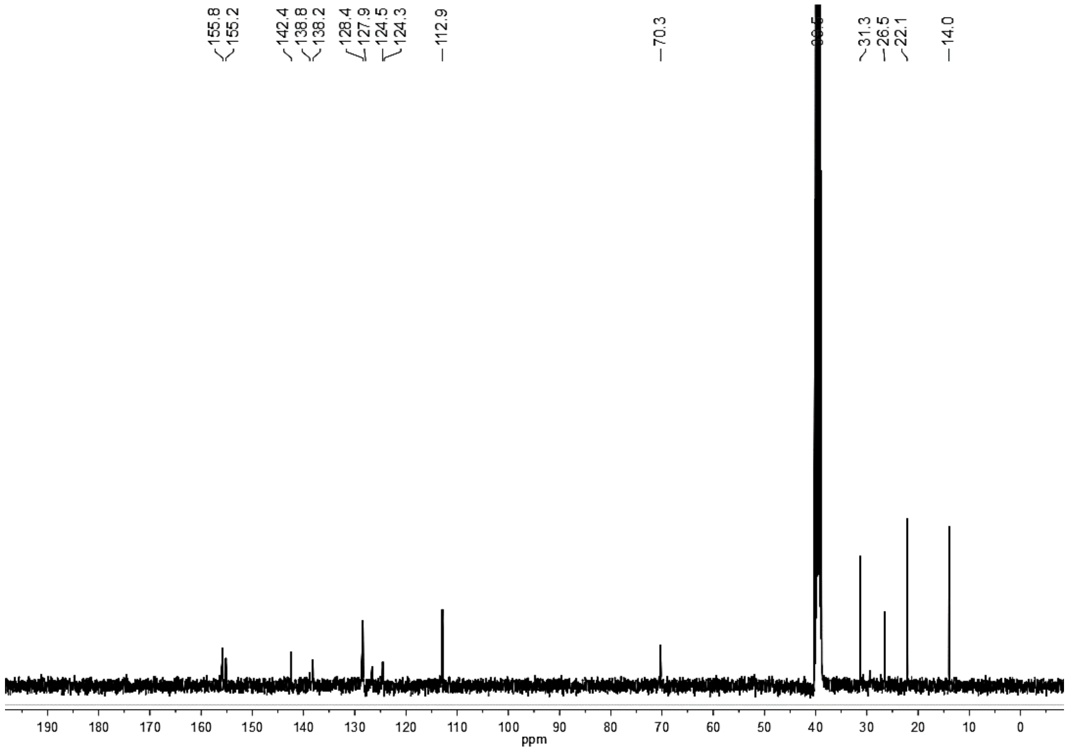

Figure S6. $^{13}C\{^{1}H\}$ NMR (101 MHz, DMSO-d_6, 298 K) of $[Cu(\mathbf{L6})_2](PF_6)$.

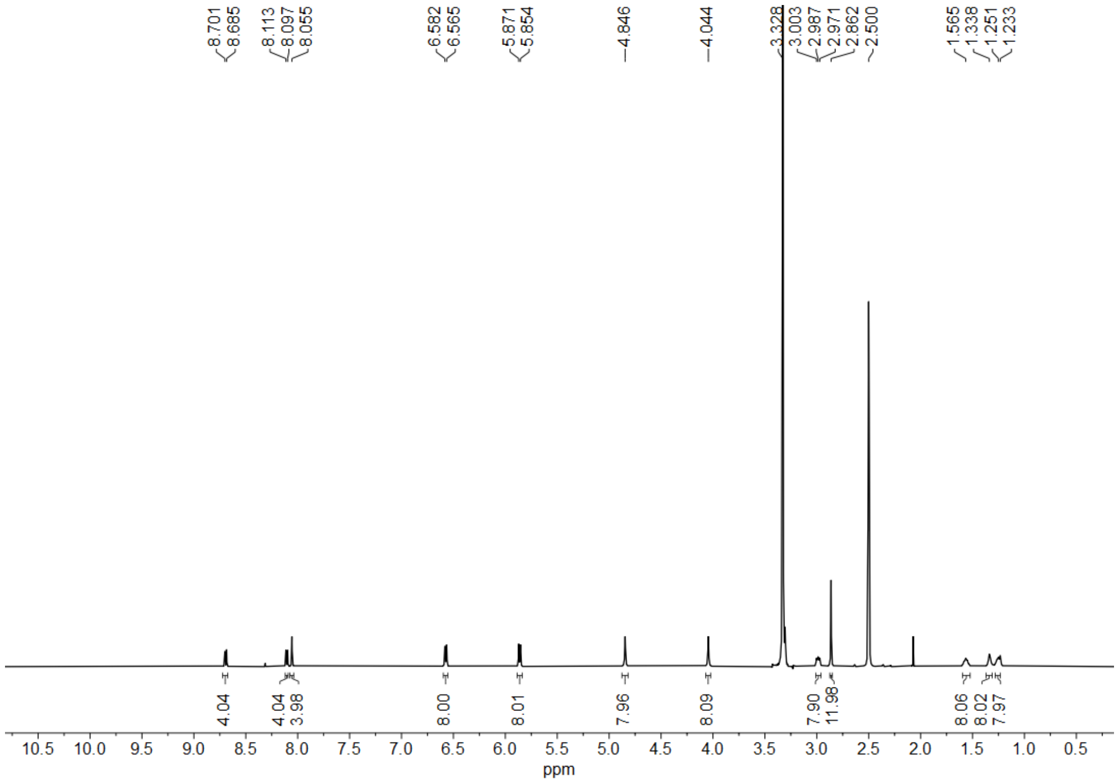

Figure S7. ^{1}H NMR (500 MHz, DMSO-d_6, 298 K) of [Cu(**C8-Ms**)](PF_6).

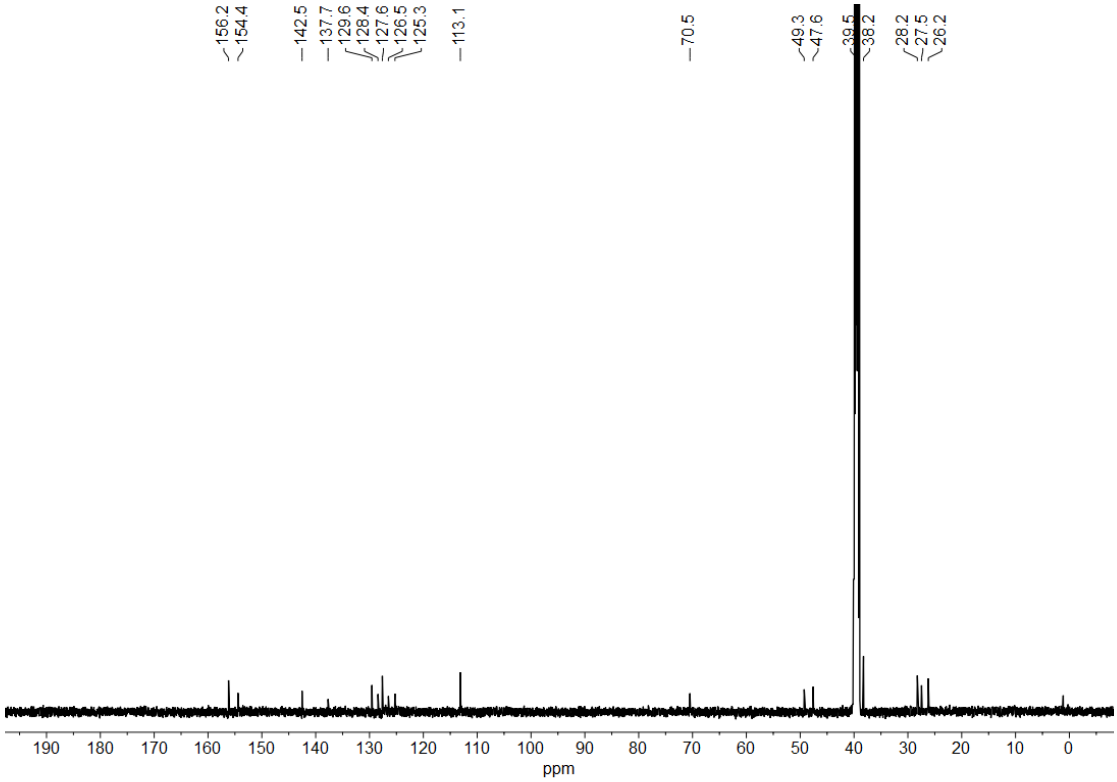

Figure S8. $^{13}C\{^{1}H\}$ NMR (126 MHz, DMSO-d_6, 298 K) of [Cu(**C8-Ms**)](PF_6).

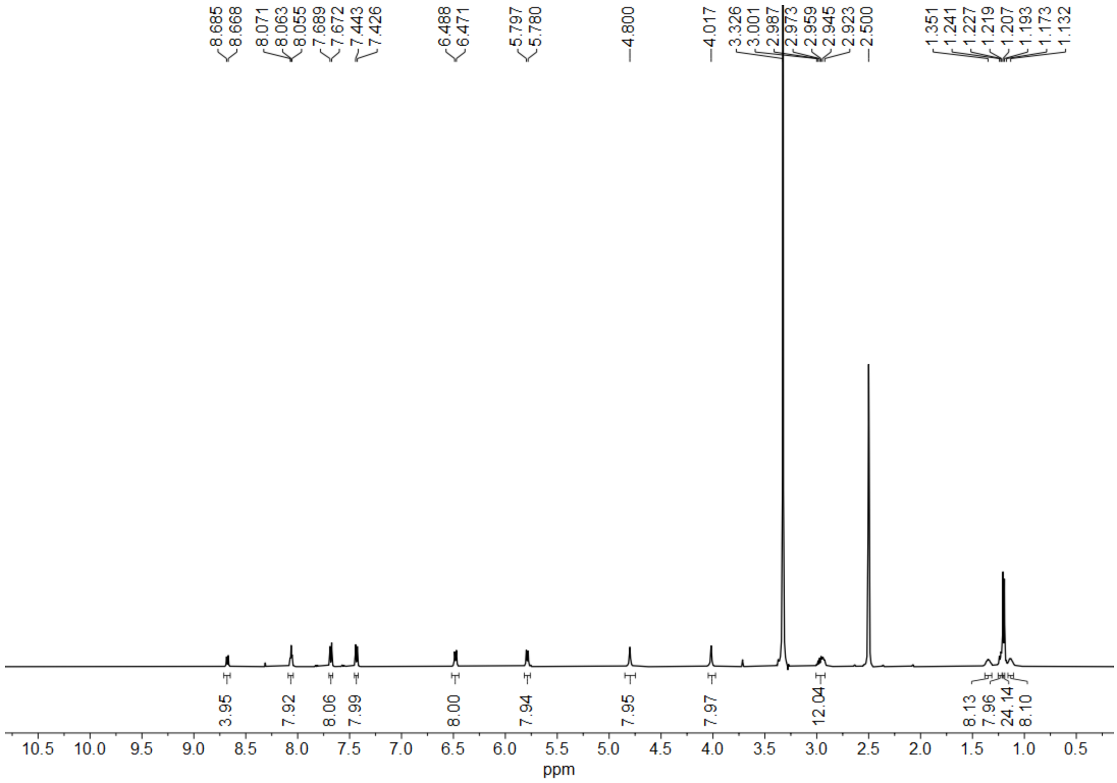

Figure S9. ^{1}H NMR (500 MHz, DMSO-d_6, 298 K) of [Cu(**C8-Ipps**)](PF_6).

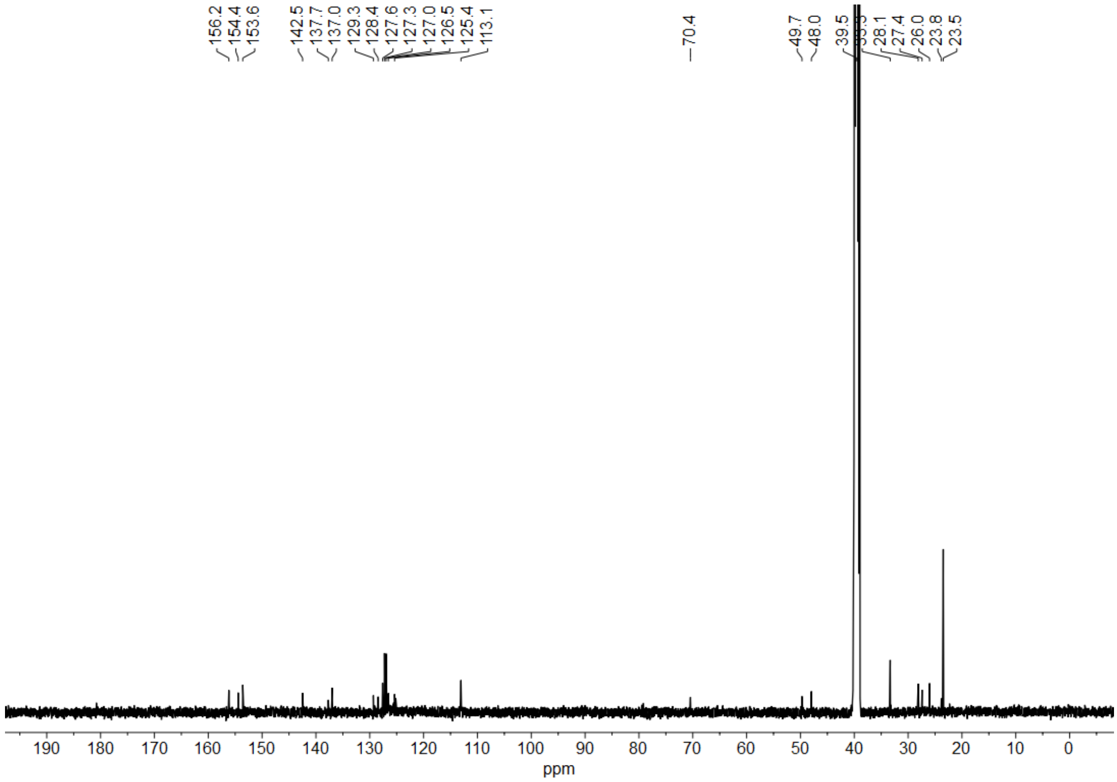

Figure S10. $^{13}C\{^{1}H\}$ NMR (126 MHz, DMSO-d_6, 298 K) of [Cu(**C8-Ipps**)](PF_6).

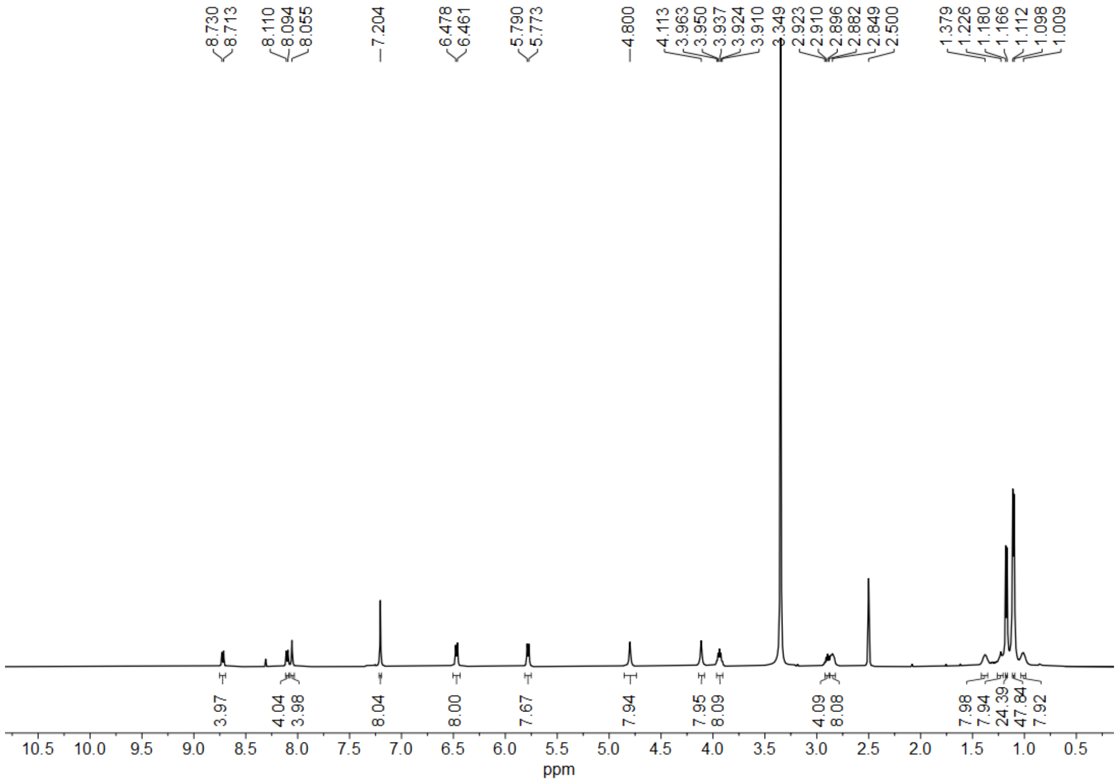

Figure S11. ^{1}H NMR (500 MHz, DMSO-d_6, 298 K) of [Cu(**C8-Tipps**)](PF_6).

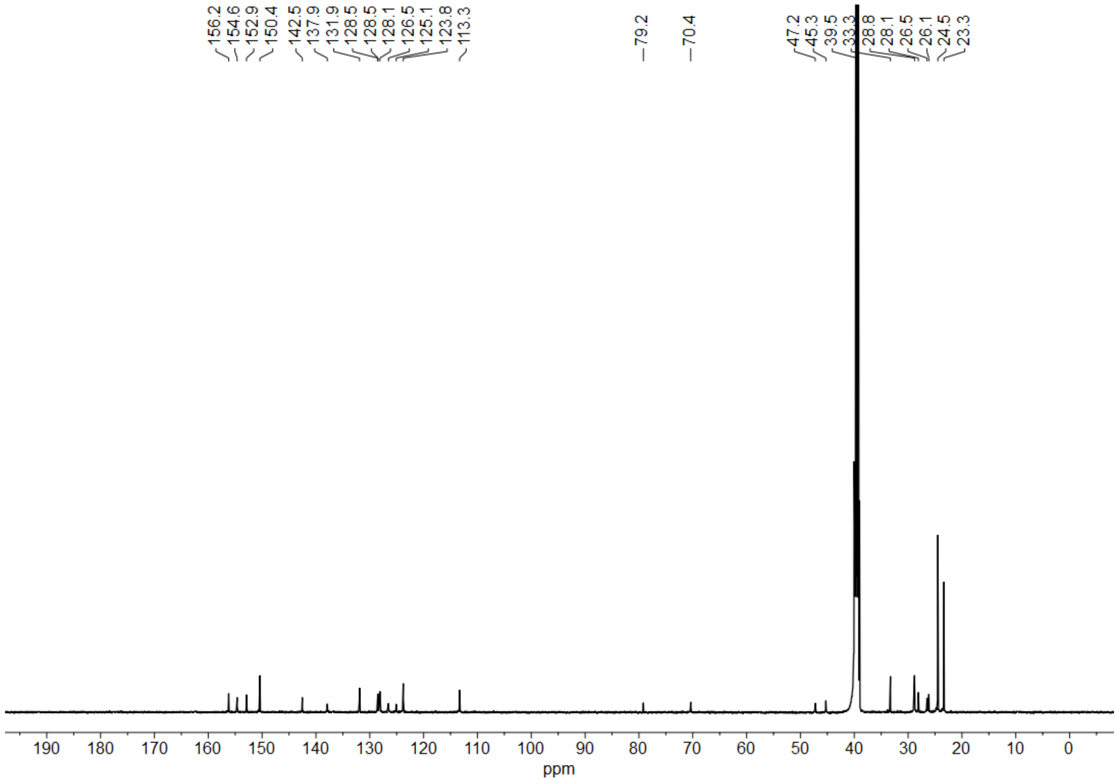

Figure S12. $^{13}C\{^{1}H\}$ NMR (126 MHz, DMSO-d_6, 298 K) of [Cu(**C8-Tipps**)](PF_6).

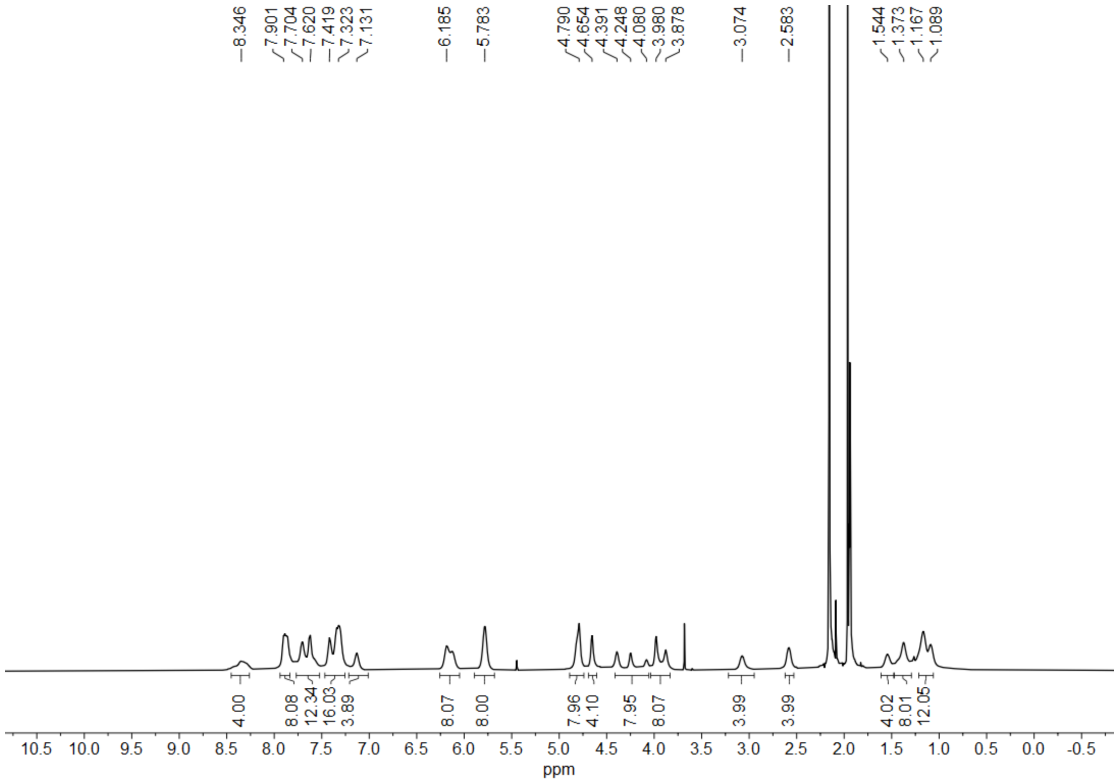

Figure S13. ^{1}H NMR (500 MHz, CD_3CN, 298 K) of [Cu(**C8-Fmoc**)](PF_6).

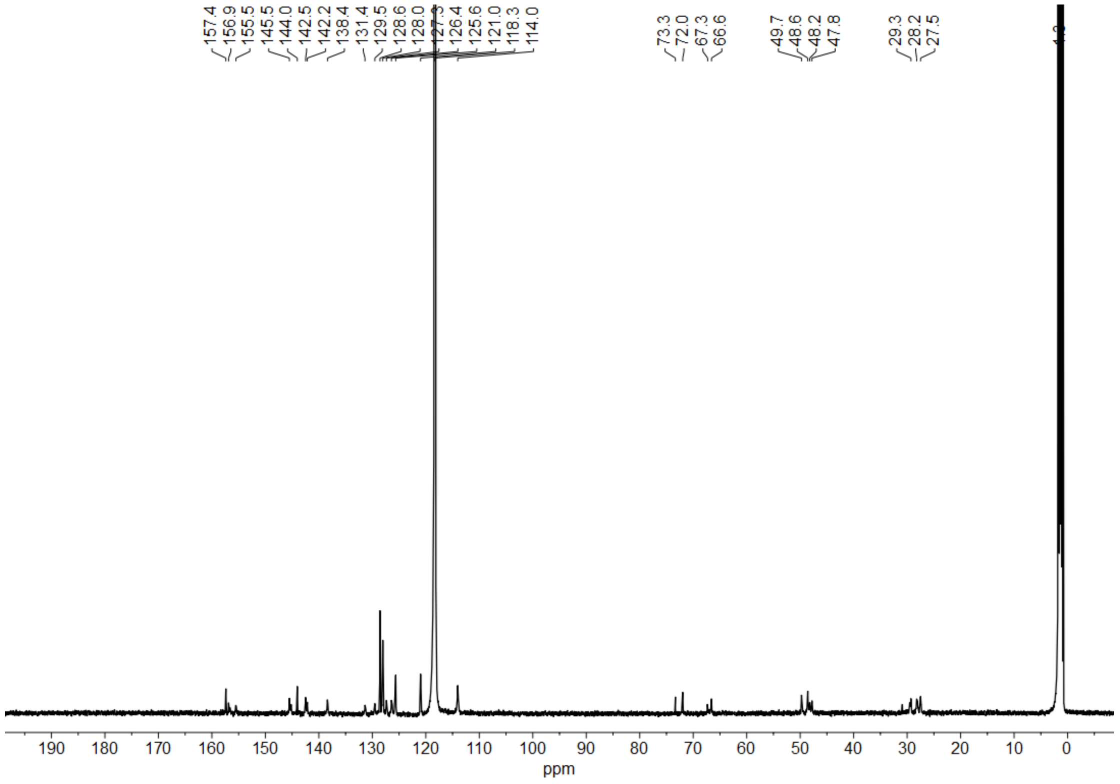

Figure S14. $^{13}C\{^{1}H\}$ NMR (126 MHz, CD_3CN, 298 K) of [Cu(**C8-Fmoc**)](PF_6).

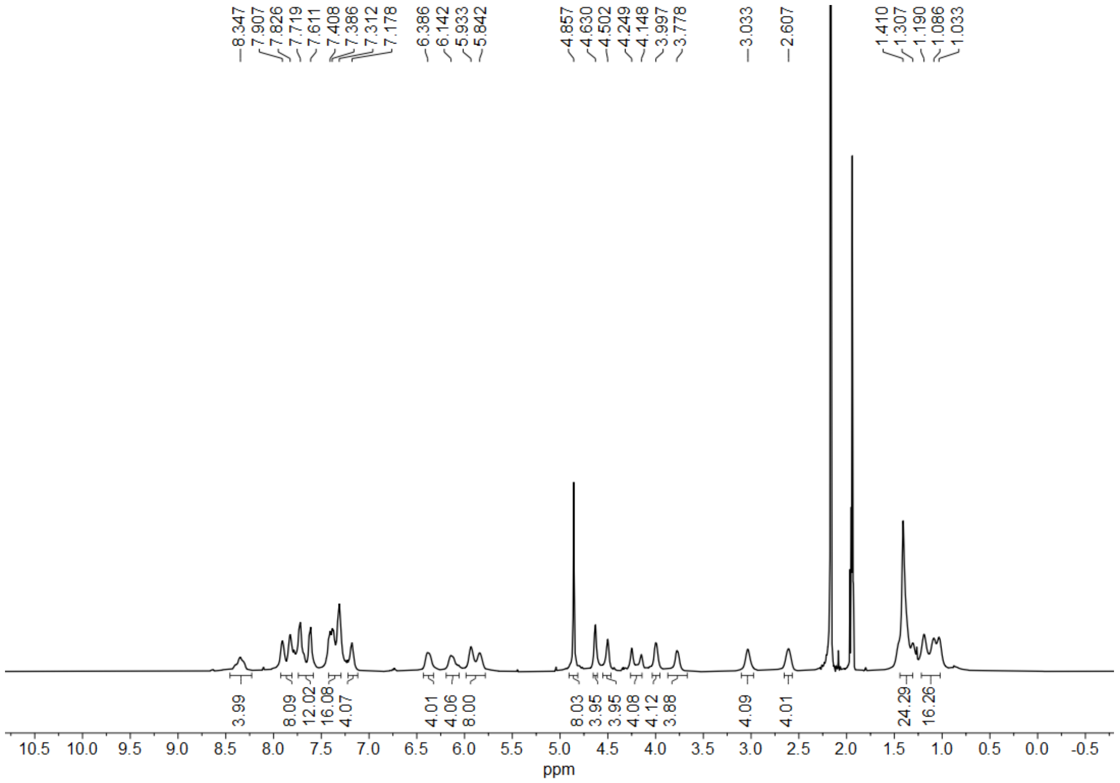

Figure S15. ^{1}H NMR (500 MHz, CD_3CN, 298 K) of [Cu(**C12-Fmoc**)](PF_6).

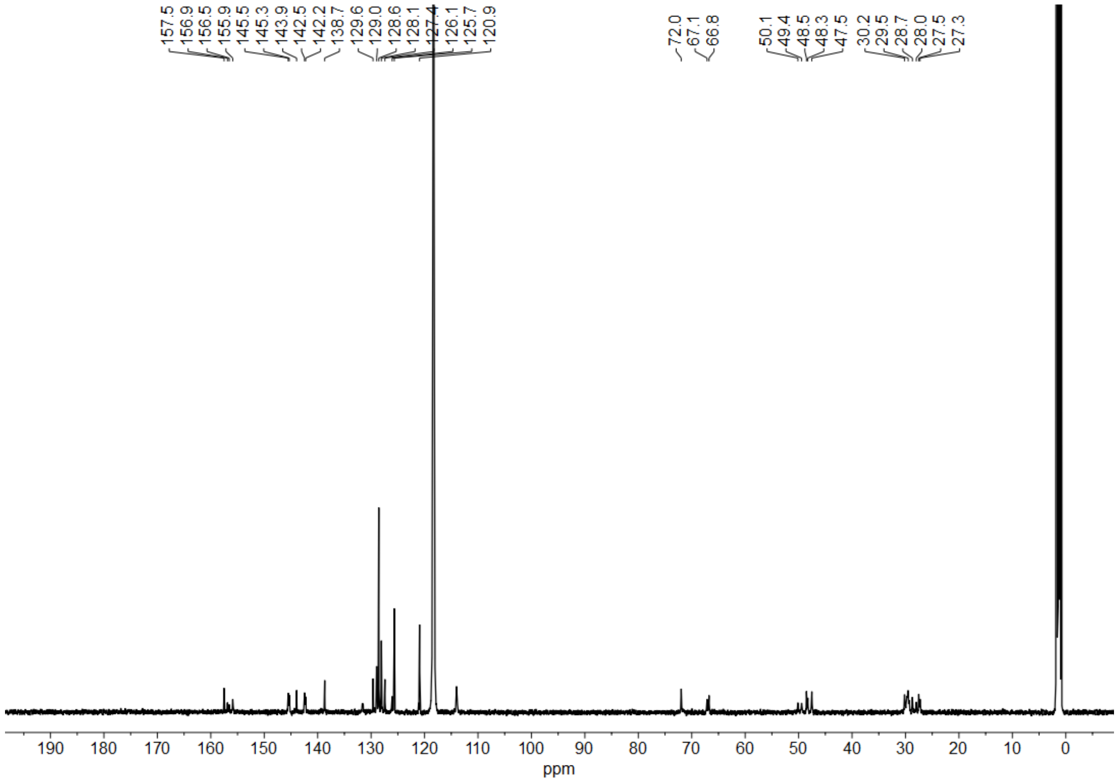

Figure S16. $^{13}C\{^{1}H\}$ NMR (126 MHz, CD_3CN, 298 K) of [Cu(**C12-Fmoc**)](PF_6).

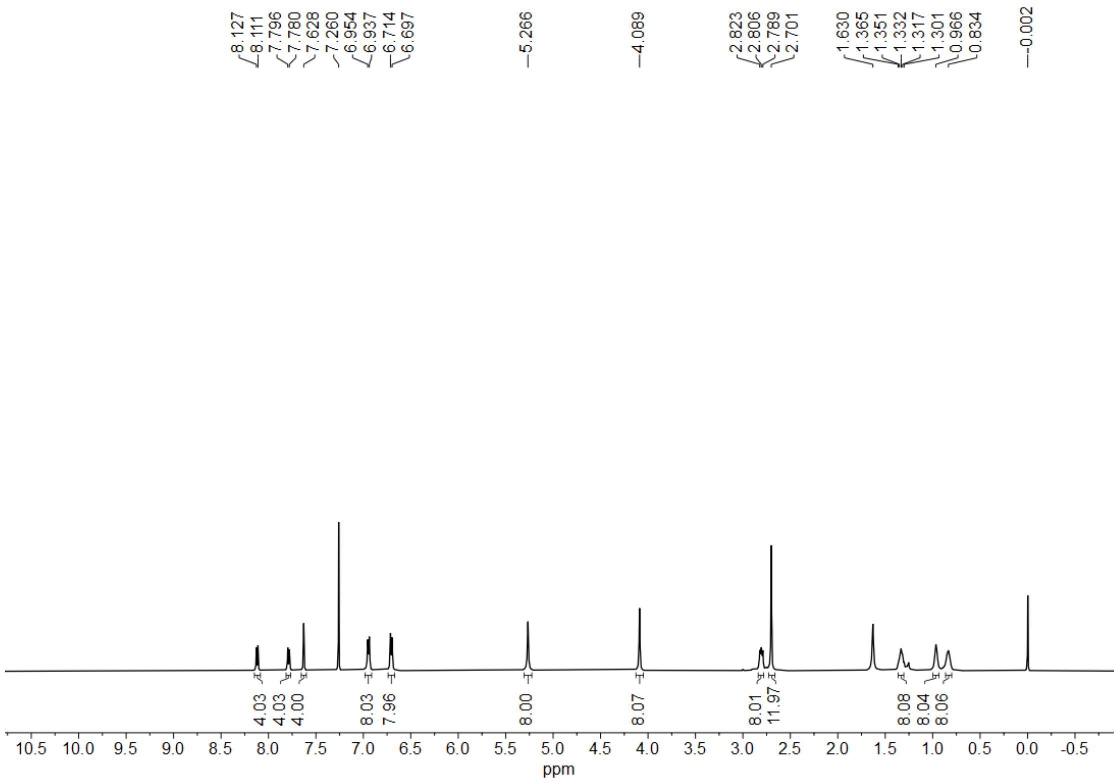

Figure S17. ^{1}H NMR (500 MHz, $CDCl_3$, 298 K) of **C8-Ms**.

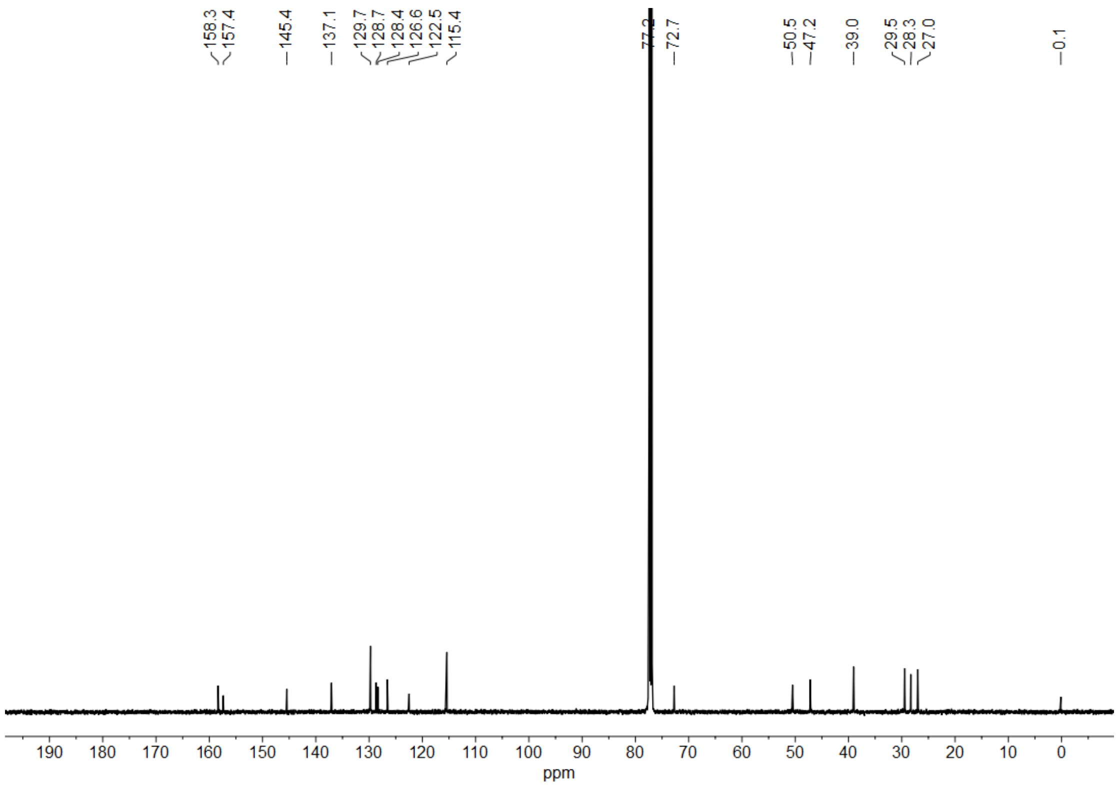

Figure S18. ^{13}C{^{1}H} NMR (126 MHz, $CDCl_3$, 298 K) of **C8-Ms**.

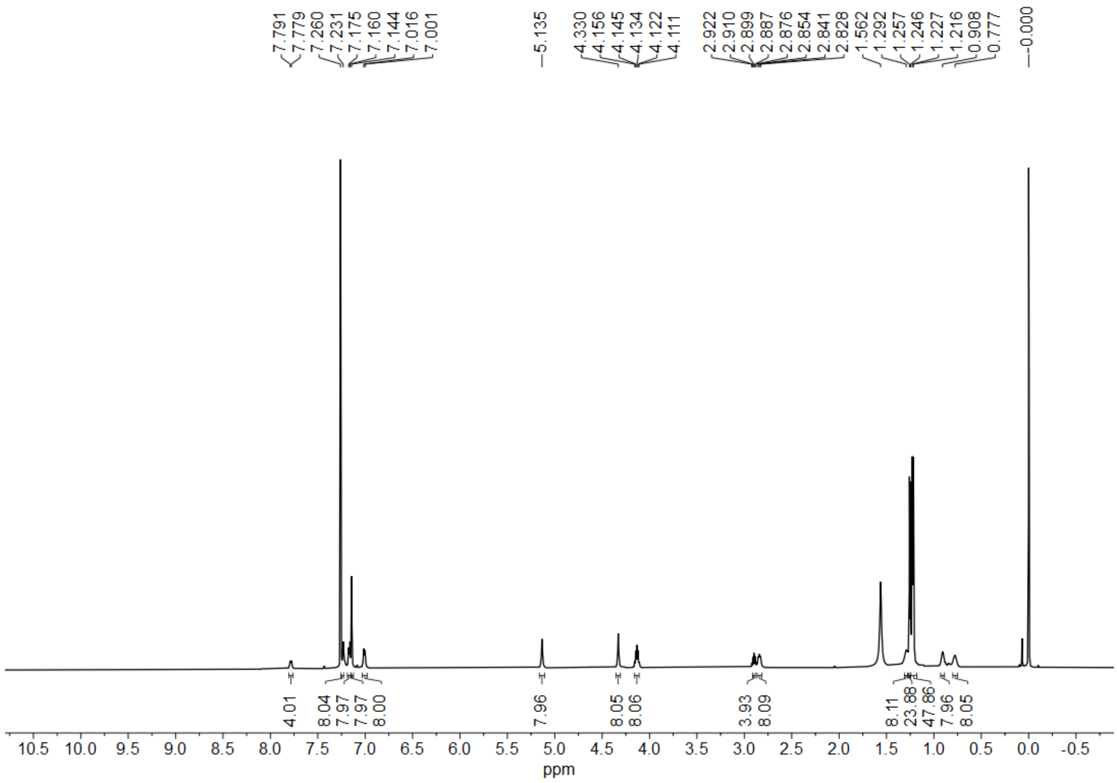

Figure S19. ^{1}H NMR (600 MHz, $CDCl_3$, 298 K) of **C8-Tipps**.

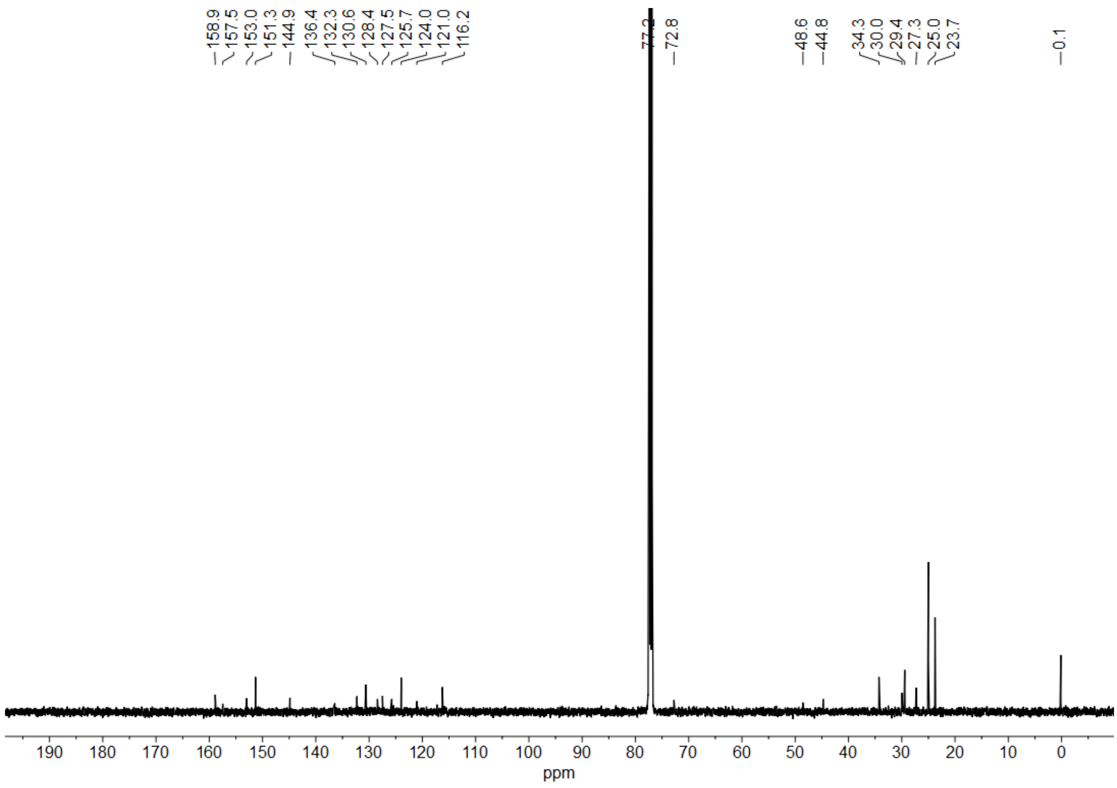

Figure S20. $^{13}C\{^{1}H\}$ NMR (126 MHz, $CDCl_3$, 298 K) of **C8-Tipps**.

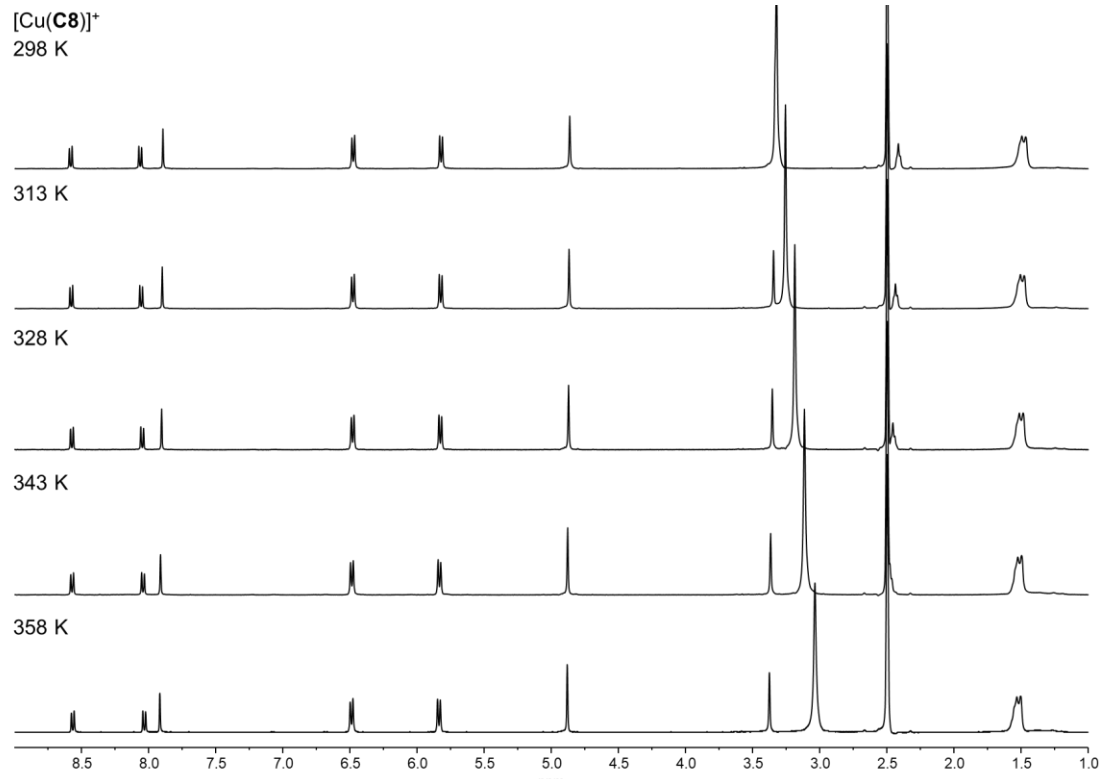

Figure S21. Variable temperature ^{1}H NMR (400 MHz, DMSO-d_6) spectra of [Cu(**C8**)](PF_6).

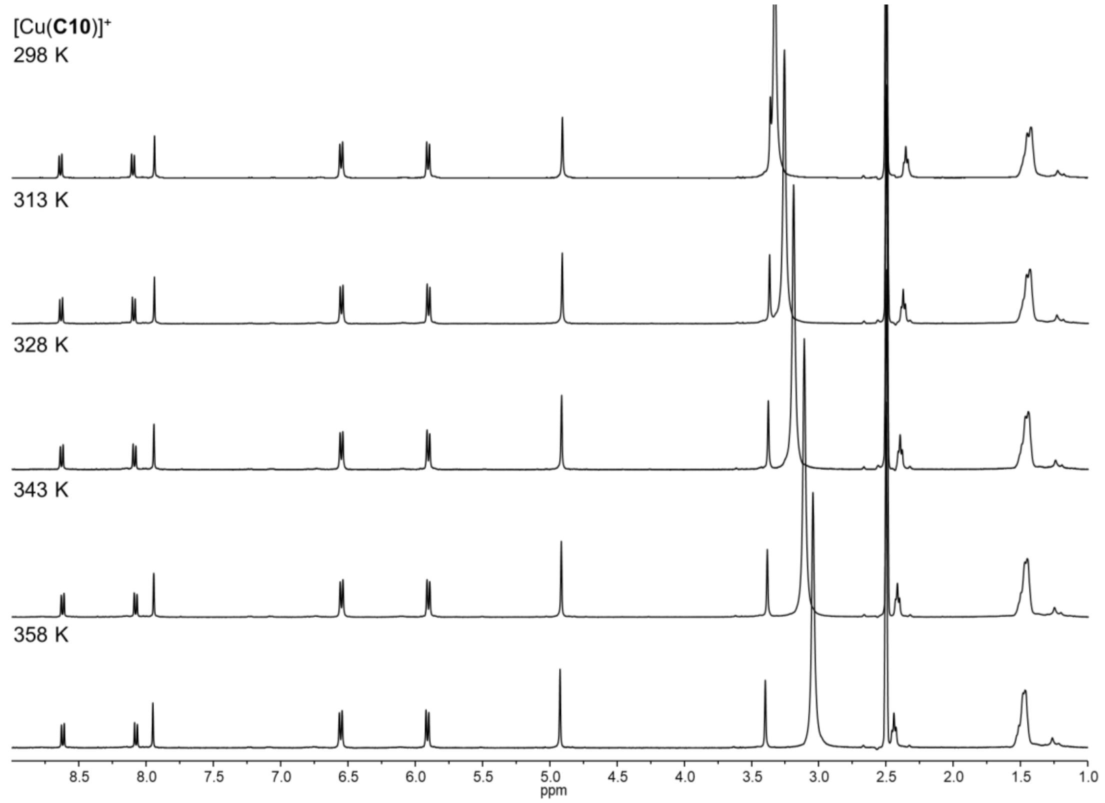

Figure S22. Variable temperature ^{1}H NMR (400 MHz, DMSO-d_6) spectra of [Cu(**C10**)](PF_6).

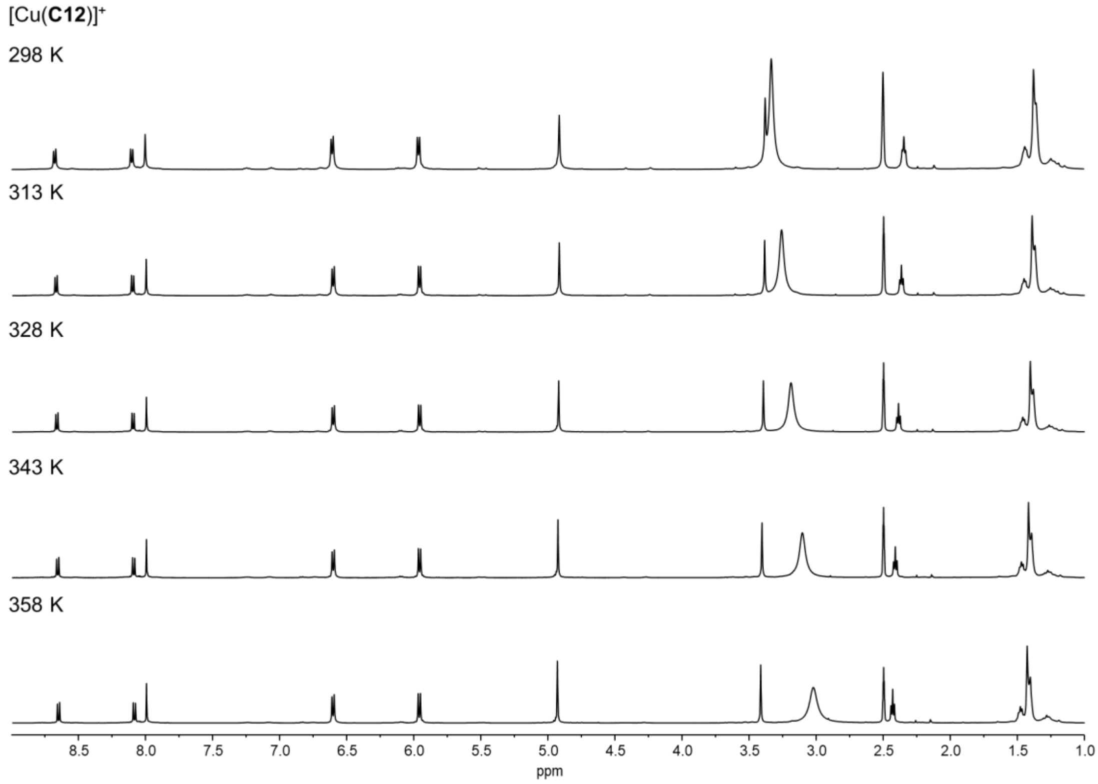

Figure S23. Variable temperature ^{1}H NMR (400 MHz, DMSO-d_6) spectra of [Cu(**C12**)](PF_6).

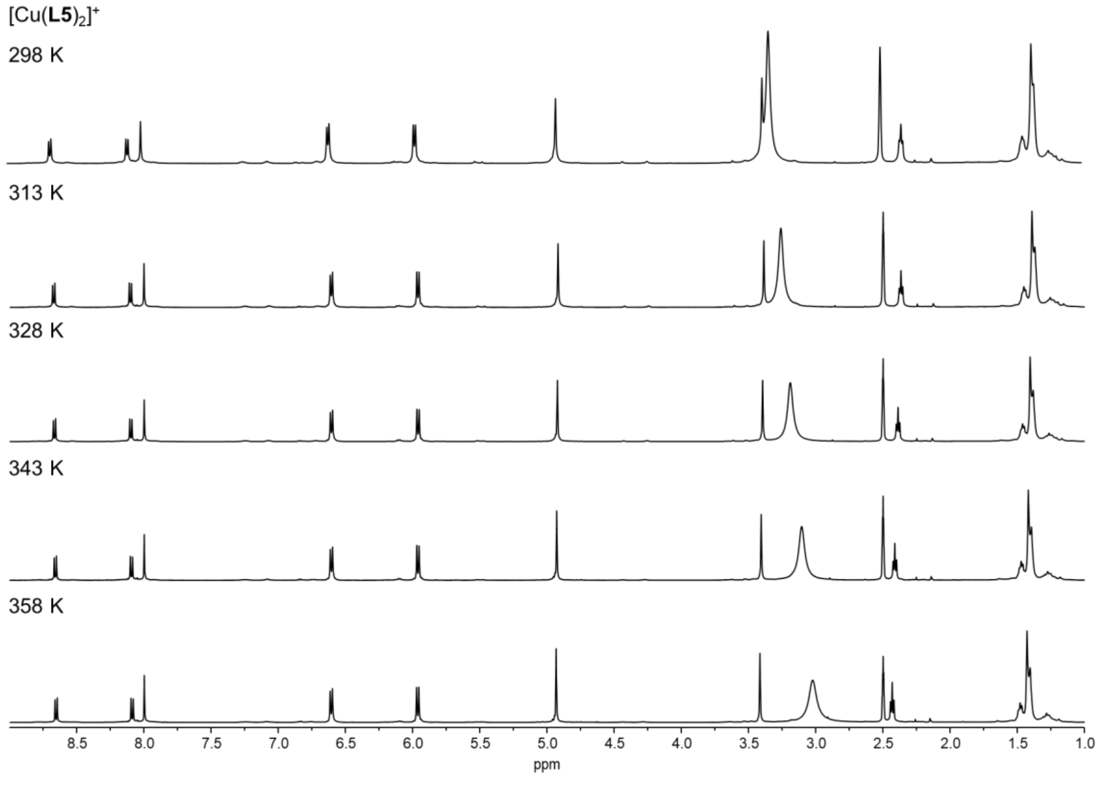

Figure S24. Variable temperature ^{1}H NMR (400 MHz, DMSO-d_6) spectra of [Cu(**L5**)$_2$](PF_6).

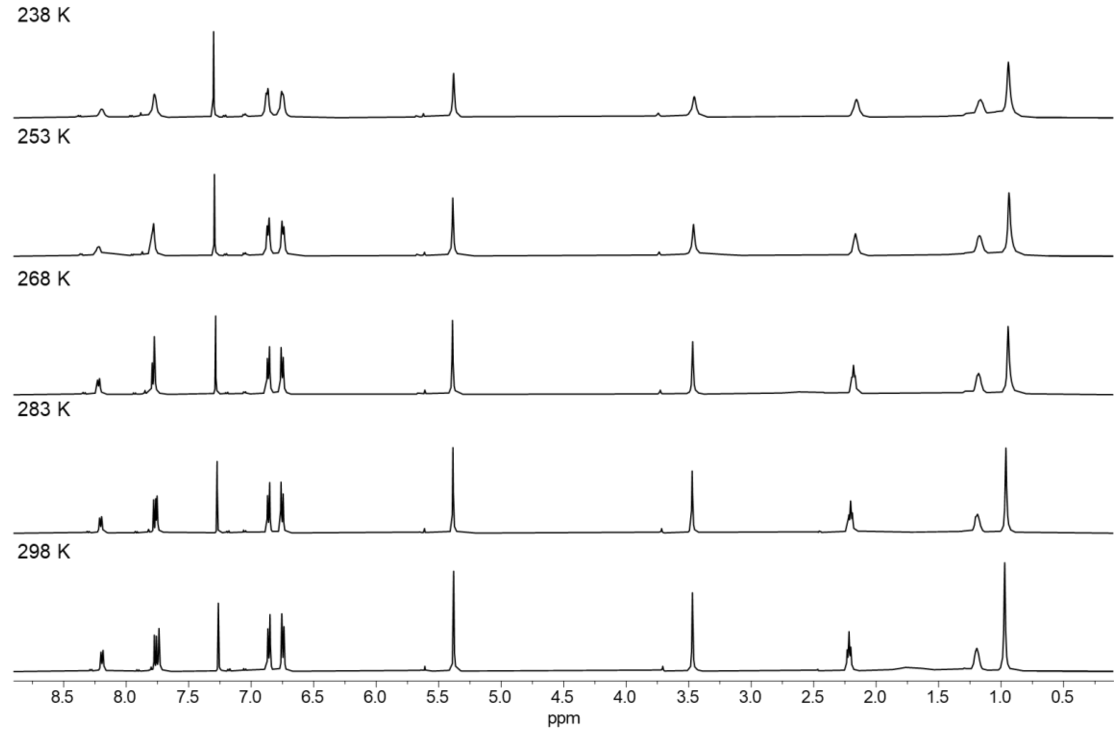

Figure S25. Variable temperature ^{1}H NMR (500 MHz, $CDCl_3$) spectra of **C8**.

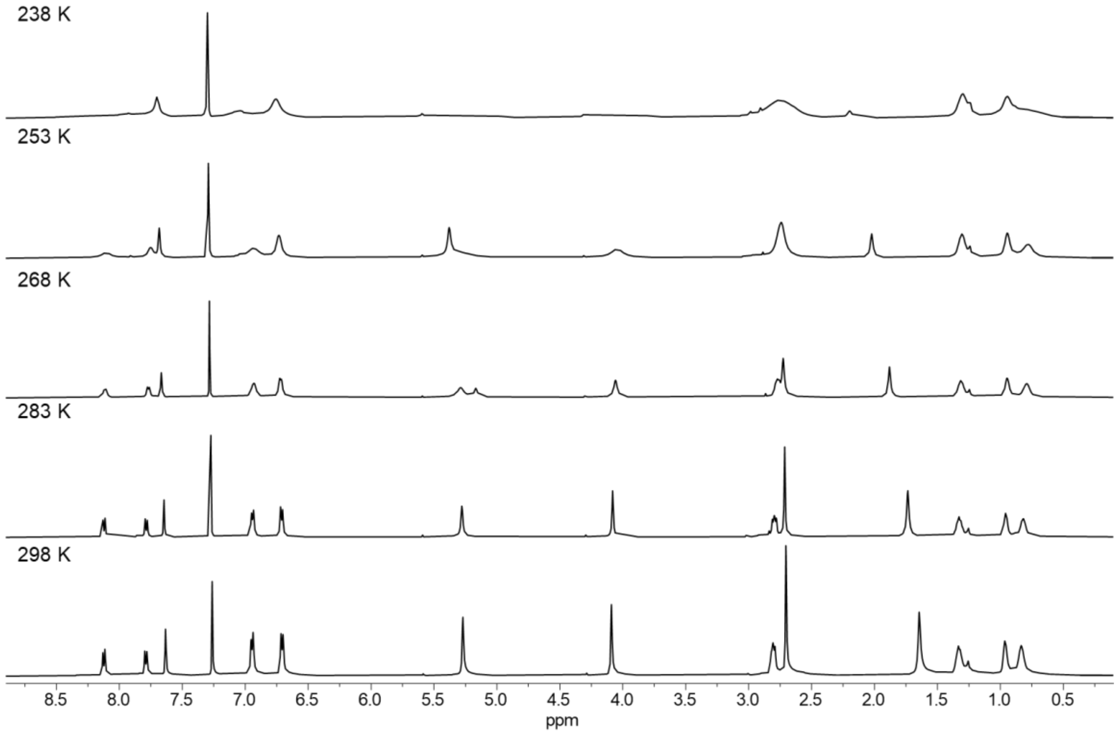

Figure S26. Variable temperature ^{1}H NMR (500 MHz, $CDCl_3$) spectra of **C8-Ms**.

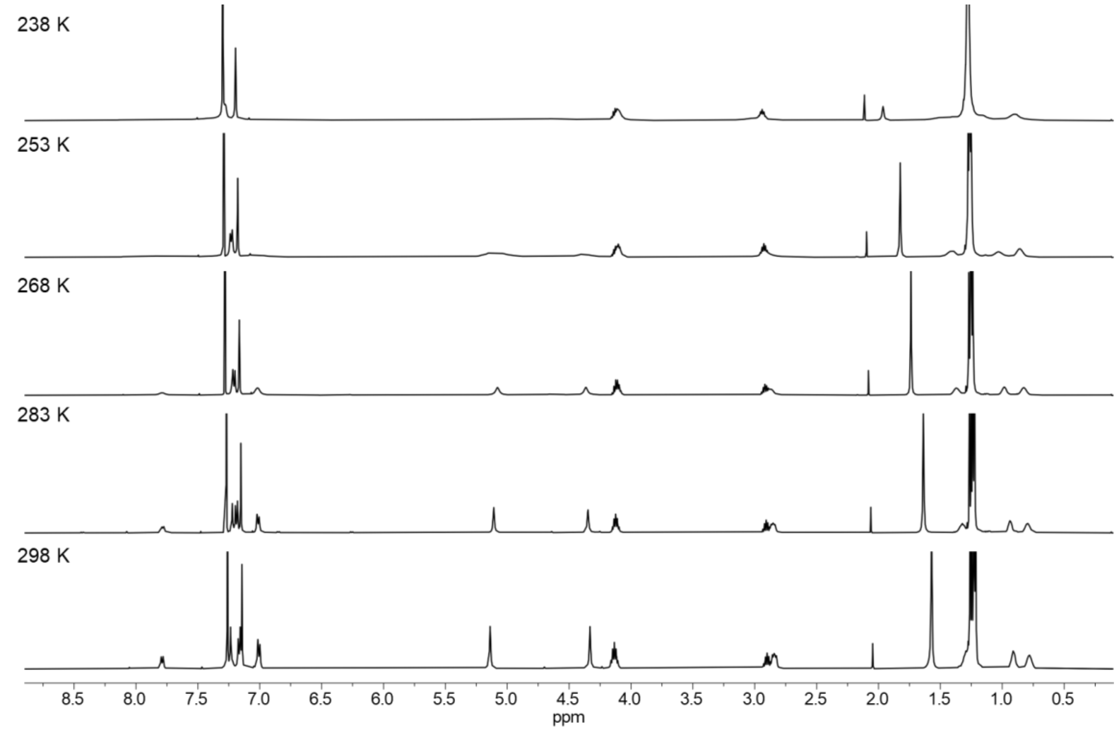

Figure S27. Variable temperature ^{1}H NMR (500 MHz, $CDCl_3$) spectra of **C8-Tipps**.

Table S1. Summary of ^{1}H NMR data.

	H_{Phen}	H_{Phen}	H_{Phen}	^{i}Ps(Ar)	H_{Ar}	H_{Ar}	OCH_2	$ArCH_2N$	NCH_2	^{i}Pr	Me	$(CH_2)_n$
[Cu(**C7**)]$^+$	8.59, d	7.93, s	8.06, d	/	6.44, d	5.80, d	4.81, s	3.32, s	2.52, br	/	/	1.52, m
[Cu(**C8**)]$^+$	8.58, d	7.90, s	8.07, d	/	6.48, d	5.83, d	4.87, s	3.34, s	2.42, t	/	/	1.47, m
[Cu(**C9**)]$^+$	8.61, d	7.91, s	8.09, d	/	6.49, d	5.86, d	4.89, s	-	2.40, t	/	/	1.44, m
[Cu(**C10**)]$^+$	8.64, d	7.94, s	8.10, d	/	6.55, d	5.91, d	4.91, s	3.37, s	2.36, t	/	/	1.42, m
[Cu(**C11**)]$^+$	8.66, d	7.97, s	8.11, d	/	6.58, d	5.94, d	4.92, s	3.39, s	2.37, t	/	/	1.41, m
[Cu(**C12**)]$^+$	8.70, d	8.02, s	8.12, d	/	6.63, d	5.98, d	4.93, s	3.40, s	2.36, t	/	/	1.40, m
[Cu(**L4**)$_2$]$^+$	8.75, d	8.11, s	8.10, d	/	6.69, d	6.05, d	4.94, s	3.40, s	2.36, br	/	/	1.36, m<br>1.28, m
[Cu(**L5**)$_2$]$^+$	8.74, d	8.11, s	8.10, d	/	6.69, d	6.05, d	4.94, s	-	2.36, br	/	/	1.38, br<br>1.24, br
[Cu(**L6)$_2$**]$^+$	8.75, d	8.12, s	8.11, d	/	6.69, d	6.06, d	4.94, s	3.41, br	2.36, br	/	/	1.37, br<br>1.23, m
[Cu(**C8-Ms**)]$^+$	8.69, d	8.06, s	8.11, d	/	6.57, d	5.86, d	4.85, s	4.04, s	2.99, t	/	2.86, s	1.57, m<br>1.34, m<br>1.25, m
[Cu(**C8-Ipps**)]$^+$	8.68, d	8.06, s	8.06, d	7.68, d<br>7.43, d	6.48, d	5.79, d	4.80, s	4.02, s	2.95, t	2.97, septet<br>1.20, d	/	1.35, m<br>1.22, m<br>1.13, m
[Cu(**C8-Tipps**)]$^+$	8.72, d	8.06, s	8.10, d	7.20, s	6.47, d	5.78, d	4.80, s	4.11, s	2.85, t	3.94, septet<br>2.90, septet<br>1.17, d<br>1.11, d	/	1.38, m<br>1.23, m<br>1.01, m

3. *IR and Raman Spectra*

FT-IR spectra were recorded on a PerkinElmer Spectrum Two FT-IR spectrometer employing powder sample. Raman spectra were recorded using a WITec Alpha 300R confocal Raman microscopy system with a Zeiss EC Epiplan-Neofluar Dic 100x/0.9 NA objective, and a 300 grooves/mm grating. Solid samples were fixed on a glass slide using a carbon tape (2 mm × 2 mm). All the spectra were recorded upon excitation by a 785 nm laser. Laser powers for excitation are 20–40 mW.

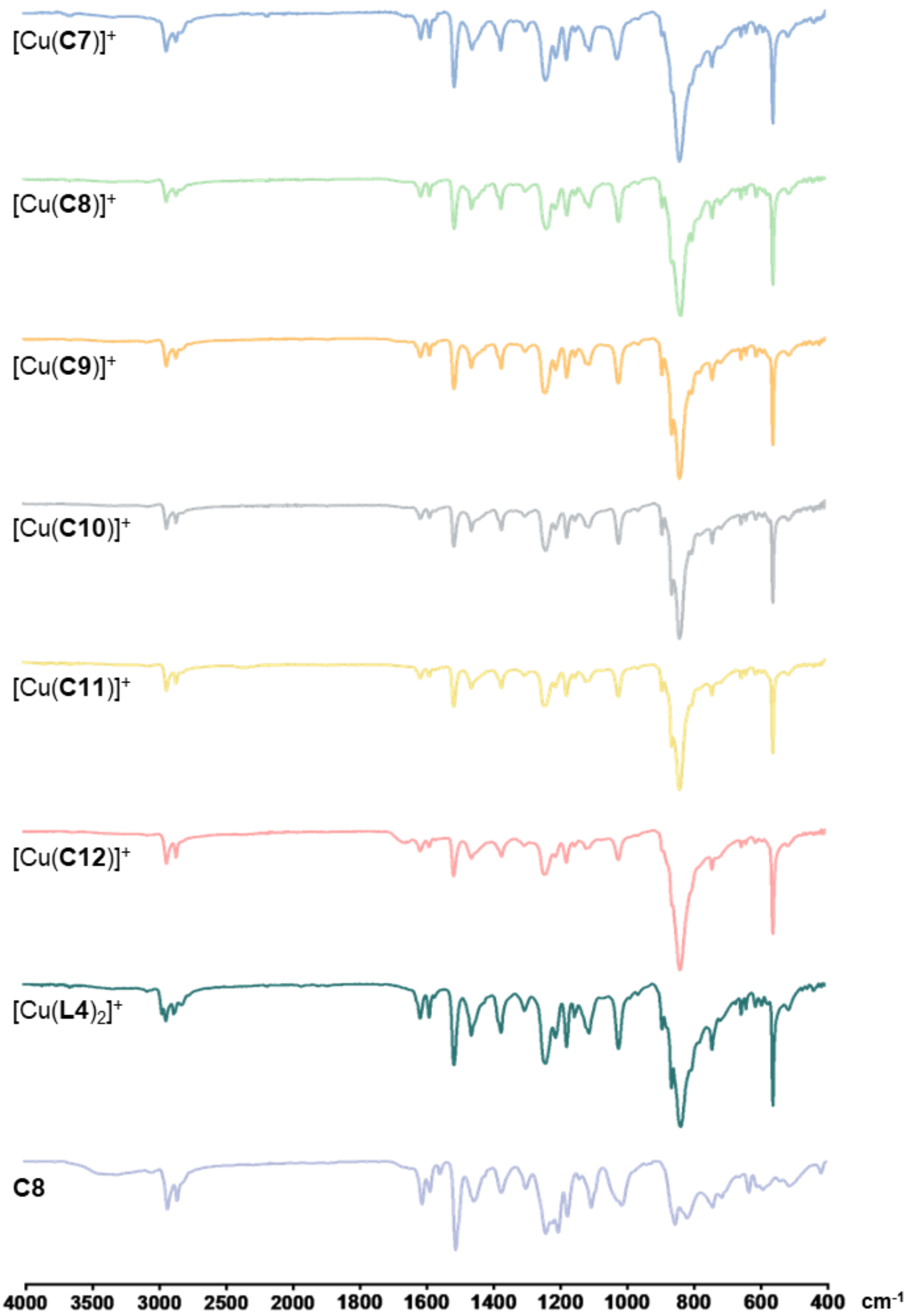

Figure S28. FT-IR spectra of [Cu(**Cn**)]$^+$ (n = 7–12), [Cu(**L4**)$_2$]$^+$, and metal-free **C8**.

Table S2. Summary of FT-IR data.

[Cu(**C7**)]$^+$	[Cu(**C8**)]$^+$	[Cu(**C9**)]$^+$	[Cu(**C10**)]$^+$	[Cu(**C11**)]$^+$	[Cu(**C12**)]$^+$	[Cu(**L4**)$_2$]$^+$	**C8**	
						2955	2924	
2926	2926	2925	2924	2924	2924	2927		
2854	2852	2854	2853	2853	2852	2809	2852	C–H stretching
						2759		
2815	2813	2812	2810	2809	2808	2866	2808	
1609	1610	1610	1610	1610	1611	1611	1609	
1583	1583	1583	1583	1584	1585	1584	1585	
1509	1509	1509	1510	1510	1511	1510	1508	
1456	1458	1457	1458	1458	1457	1458	1455	
1443	1442	1441	1440	1440	1440	1443	1423	
1380	1380	1378	1376	1378	1378	1377	1372	
1370	1369	1369	1368	1368	1368	1370		
1297	1298	1298	1297	1300	1300	1299	1299	
1236	1234	1238	1236	1238	1238	1238	1239	*ν*C–C, *ν*C–N, δC–H
							1220	
1206	1207	1206	1206	1207	1207	1207	1202	
1174	1173	1173	1173	1174	1174	1173	1175	
1149	1148	1147	1149	1149	1149	1149	1140	
1116	1117	1117	1117	1117	1117	1115	1103	
1105	1105	1106	1107	1106	1105	1108	1092	
							1027	
1023	1019	1019	1019	1019	1019	1018	1013	
888	888	889	888	888	887	888		PF_6^-
858	858	859	859	859	858	859	853	
836	833	836	835	835	835	834	816	
801	799	800	800	800	802	801		
776	778	777	776	776	777	777		
739	738	738	738	739	739	738	741	
715	714	713	712	712	721	714	713	ring breathing and deforming, ρC–H
							702	
652	652	652	652	652	651	652		
637	637	637	637	637	637	637	633	
605	606	606	608	608	609	609	607	
585	589	588	588	589	590	591	590	
556	557	557	556	557	557	573		PF_6^-
							540	
511	509	509	510	511	511	567	512	
							417	

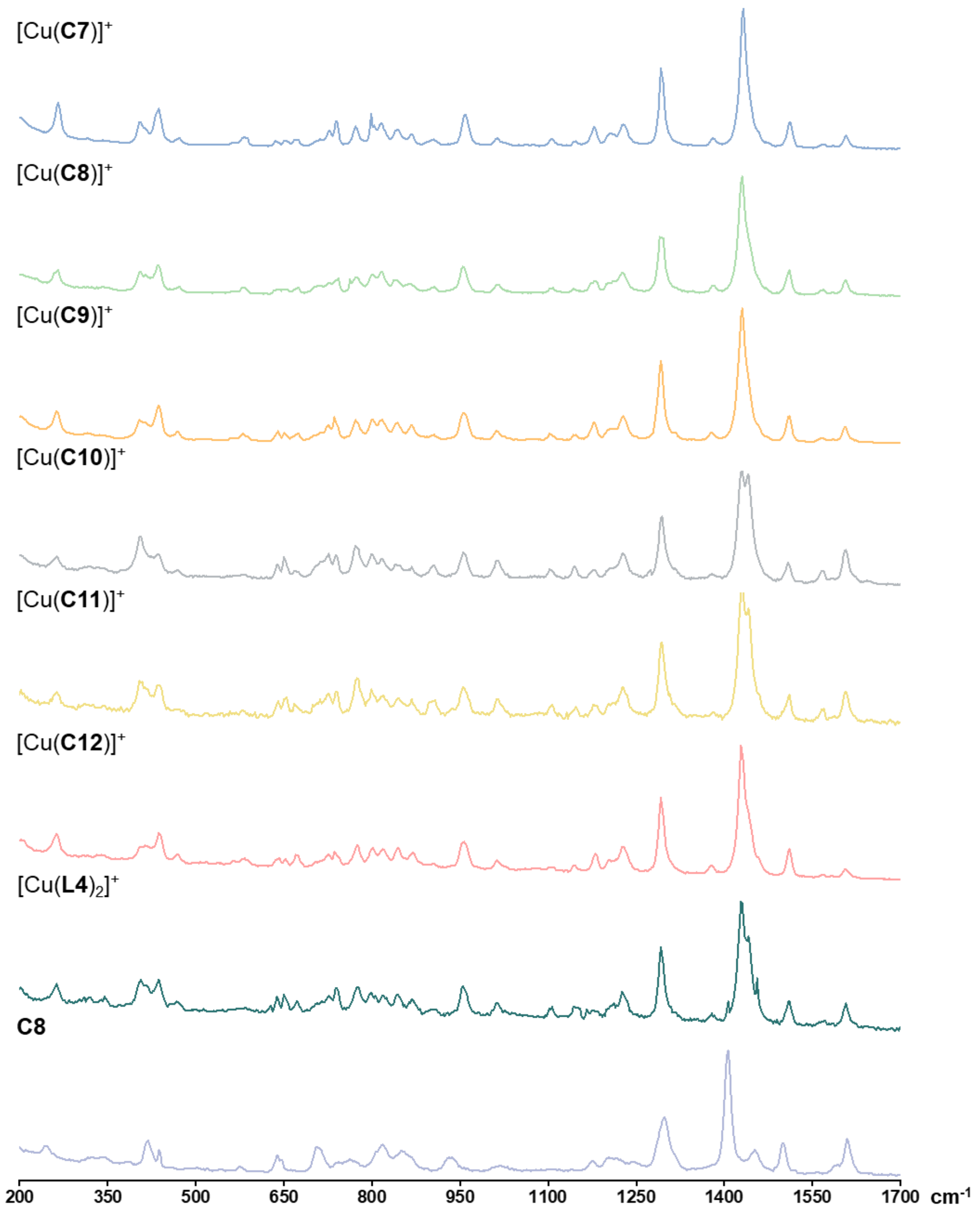

Figure S29. Raman spectra of the [2]catenane complexes [Cu(**Cn**)]$^{+}$ (n = 7–12), the non-interlocked complex [Cu(**L4**)$_2$]$^{+}$, and the metal-free [2]catenane **C8**.

Table S3. Summary of Raman shifts.

$[Cu(\mathbf{C7})]^+$	$[Cu(\mathbf{C8})]^+$	$[Cu(\mathbf{C9})]^+$	$[Cu(\mathbf{C10})]^+$	$[Cu(\mathbf{C11})]^+$	$[Cu(\mathbf{C12})]^+$	$[Cu(\mathbf{L4})_2]^+$	**C8**
266	266	263	263	263	263	263	245
404	404	404	406	404	406	406	
414	414	414			414	417	419
437	437	437	434	437	437	437	437
473	473	470	470	470	470	468	
581	581	581	581	581	581	581	573
						628	
636	641	641	641	641	643	638	645
655	655	650	650	655	653	650	
675	675	675	668	668	670	673	
							707
726	726	726	726	726	726	726	
741	741	736	738	738	738	738	743
772	775	772	772	775	775	775	763
799	801	801	801	799	801	799	
816	818	816	818	816	818	818	818
844	844	844	844	844	844	844	851
868	868	868	868	868	870	870	
906	906	906	906	906	906	906	
960	955	955	955	955	955	955	934
1013	1013	1013	1013	1013	1013	1013	1018
1105	1105	1102	1102	1105	1105	1107	
1145	1143	1145	1145	1145	1145	1145	
1179	1179	1179	1179	1179	1179	1179	1177
1208	1208	1208	1208	1208	1208	1208	1201
1228	1228	1228	1228	1228	1228	1228	1219
1292	1292	1292	1294	1294	1292	1292	1299
1381	1381	1381	1381	1381	1379	1379	
						1407	1407
1433	1431	1431	1430	1430	1428	1428	1452
			1441	1441	1441	1441	
						1456	
1509	1509	1509	1509	1509	1509	1509	1501
1568	1568	1568	1568	1568	1568	1572	1589
1607	1607	1607	1607	1607	1607	1607	1609

4. DFT Computation

All calculations were performed using the Gaussian 16 software package.[3] The ground-state geometries were optimized through DFT calculations with a density matrix convergence criterion of 10^{-8}, employing a 6-31 + G**/LANL2DZ(Cu) mixed basis set [4,5] and the B3LYP exchange-correlation functional.[6-8] To account for solvation effects, acetonitrile (ε = 35.688) was considered using the conductor-like polarizable continuum model (CPCM).[9] The optimized singlet ground state (S_0) geometries served as the basis for calculating vibrational frequencies, ensuring that all stationary points represented minima on the potential energy surface (NIMAG = 0).

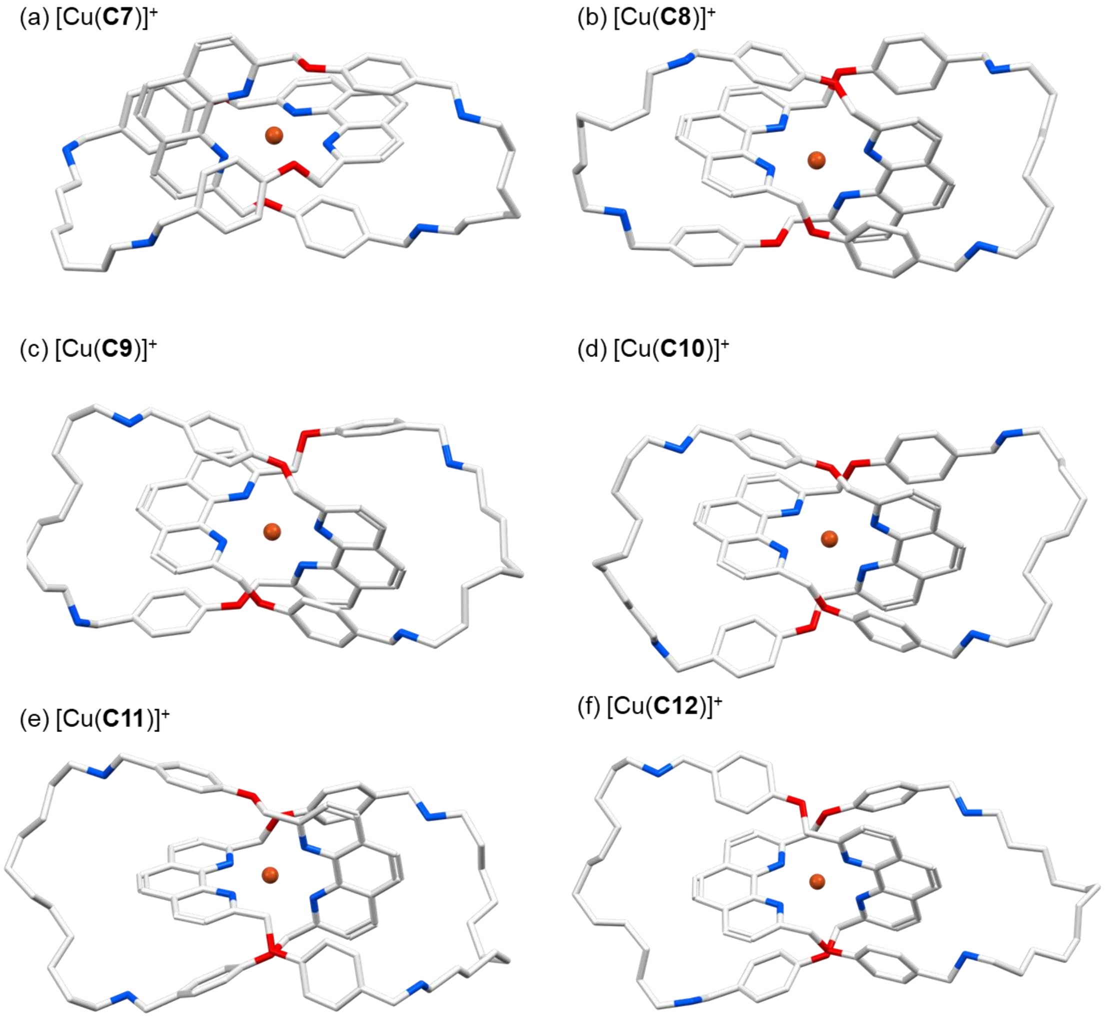

Figure S30. Representative structures of geometrically optimized S_0 state of (a) [Cu(**C7**)]$^+$; (b) [Cu(**C8**)]$^+$; (c) [Cu(**C9**)]$^+$; (d) [Cu(**C10**)]$^+$; (e) [Cu(**C11**)]$^+$; (f) [Cu(**C12**)]$^+$. Cu: orange, O: red, N: blue, C: grey. Protons are omitted for clarity.

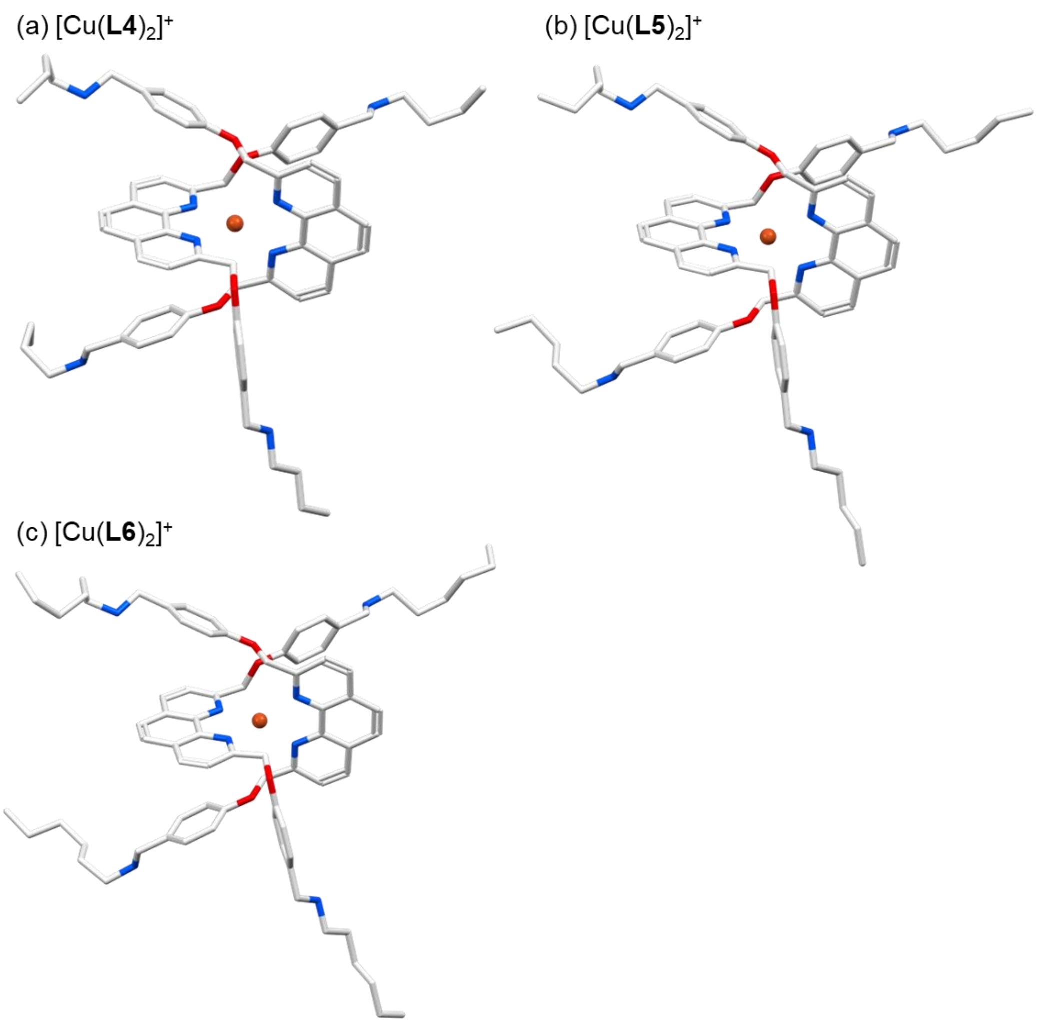

Figure S31. Representative structures of geometrically optimized S_0 state of (a) [Cu(**L4**)$_2$]$^+$; (b) [Cu(**L5**)$_2$]$^+$; (c) [Cu(**L6**)$_2$]$^+$. Cu: orange, O: red, N: blue, C: grey. Protons are omitted for clarity.

Table S4. Structural parameters of DFT-simulated [Cu(**Cn**)]$^+$ (n = 7–12) and [Cu(**Ln**)$_2$]$^+$ (n = 4–6) in the ground state.

	α (°)	*d* (Å)	β (°)	$d_{\text{Cu-N}}$ (Å)					
				Cu-N1	Cu-N2	Cu-N3	Cu-N1	avg.	$\Delta d_{\text{Cu-N}}$
[Cu(**C7**)]$^+$	82.98	6.108	149.53	2.071	2.204	2.074	2.174	2.131	0.133
[Cu(**C8**)]$^+$	81.29	6.265	164.68	2.082	2.168	2.100	2.147	2.124	0.086
[Cu(**C9**)]$^+$	82.69	6.232	164.80	2.077	2.139	2.075	2.147	2.110	0.072
[Cu(**C10**)]$^+$	79.84	6.305	168.51	2.099	2.148	2.091	2.161	2.125	0.070
[Cu(**C11**)]$^+$	80.46	6.334	171.11	2.113	2.143	2.104	2.157	2.129	0.053
[Cu(**C12**)]$^+$	81.29	6.302	170.68	2.099	2.133	2.105	2.140	2.119	0.041
[Cu(**L4**)$_2$]$^+$	82.06	6.312	172.36	2.106	2.130	2.114	2.129	2.120	0.024
[Cu(**L5**)$_2$]$^+$	81.97	6.313	172.31	2.107	2.131	2.120	2.123	2.120	0.024
[Cu(**L6**)$_2$]$^+$	82.21	6.313	172.03	2.106	2.133	2.116	2.128	2.121	0.027

Table S5. Cartesian coordinates of the complex [Cu(**C7**)]$^+$ at its optimized S_0 geometry.

atom		x	y	z	atom		x	y	z
1	C	-4.86321	-0.59772	2.480889	47	C	0.183878	-3.17367	0.750279
2	C	-4.26397	-0.34441	3.676697	48	C	-4.57668	3.733133	-1.55388
3	C	-2.86794	-0.01977	3.743094	49	C	-5.93565	3.452783	-1.71743
4	C	-2.09433	0.04232	2.553971	50	C	-6.83313	3.553046	-0.64415
5	C	-2.72508	-0.25961	1.282515	51	C	-6.32959	3.959089	0.60195
6	C	-4.1106	-0.56535	1.259425	52	C	-4.97456	4.242152	0.780805
7	N	-1.96244	-0.26273	0.155521	53	C	-0.66974	-3.23923	1.858873
8	C	-2.53042	-0.56171	-1.01804	54	C	-1.86168	-3.95359	1.777667
9	C	-3.90799	-0.83284	-1.1291	55	C	-2.22847	-4.63556	0.607355
10	C	-4.69435	-0.83703	0.005654	56	C	-1.32623	-4.62309	-0.4628
11	C	-2.21085	0.25691	4.961279	57	C	-0.12398	-3.91013	-0.40164
12	C	-0.87112	0.584693	4.951434	58	C	-8.31187	3.219667	-0.80127
13	C	-0.18146	0.645621	3.722535	59	C	-3.58763	-5.29374	0.481527
14	N	-0.77937	0.376973	2.560252	60	N	-8.72463	1.864477	-0.39684
15	C	-1.63234	-0.71039	-2.22507	61	N	-4.3064	-4.76761	-0.69107
16	O	-1.0077	0.533675	-2.53773	62	C	3.395567	-0.12234	3.629537
17	C	1.282409	1.015836	3.691754	63	C	4.111286	1.063987	3.431209
18	O	2.043349	-0.18416	3.865931	64	C	5.472273	1.001345	3.11935
19	C	0.170498	0.497071	-3.24699	65	C	6.152735	-0.21651	3.01987
20	C	0.677727	-0.64148	-3.8849	66	C	5.430388	-1.39079	3.282923
21	C	1.942769	-0.58826	-4.48264	67	C	4.069624	-1.3504	3.577974
22	C	2.716934	0.576025	-4.4626	68	C	9.450807	-1.86223	-2.41025
23	C	2.151026	1.726297	-3.89047	69	C	-5.75258	-5.01019	-0.65018
24	C	0.891126	1.698011	-3.30144	70	C	-8.2218	0.783331	-1.24944
25	C	4.159285	0.555848	-4.92593	71	C	6.439255	-0.00991	-4.13864
26	C	7.619008	-0.2368	2.623074	72	C	7.167329	-0.91011	-3.13223
27	N	5.001439	-0.00317	-3.85729	73	C	8.690534	-0.71527	-3.10181
28	N	8.031712	-1.52694	2.066228	74	C	-8.65373	-0.58521	-0.71
29	C	3.670129	4.217528	-0.19296	75	C	-8.21602	-1.75726	-1.60241
30	C	4.479209	3.121752	-0.23554	76	C	-8.46004	-3.1347	-0.96368
31	C	3.933119	1.795164	-0.17298	77	C	-7.96203	-4.32697	-1.80644
32	C	2.534205	1.612111	-0.03519	78	C	-6.43036	-4.46113	-1.91466
33	C	1.676506	2.779856	0.019627	79	C	-1.95265	3.291845	0.167955
34	C	2.246448	4.07433	-0.07994	80	Cu	-0.05653	0.478442	0.481127
35	N	0.340436	2.585689	0.12389	81	C	9.102887	-2.14447	-0.93603
36	C	-0.48247	3.632538	0.096	82	C	9.585532	-1.08868	0.073518
37	C	-0.00682	4.95561	-0.00291	83	C	9.40574	-1.53937	1.539599
38	C	1.355571	5.171398	-0.07914	84	H	-5.92117	-0.83704	2.429309
39	C	4.732001	0.636415	-0.24995	85	H	-4.83238	-0.37825	4.601267
40	C	4.133794	-0.60688	-0.22664	86	H	-4.33278	-1.04647	-2.10397
41	C	2.734043	-0.7037	-0.11729	87	H	-5.75487	-1.05755	-0.05363
42	N	1.958903	0.379049	0.001696	88	H	-2.76761	0.214936	5.892812
43	O	-2.7532	4.43131	-0.13942	89	H	-0.34318	0.805776	5.872992
44	C	2.05322	-2.04677	-0.26829	90	H	-2.21317	-1.06894	-3.08256
45	O	1.275358	-2.34984	0.887619	91	H	-0.87343	-1.4643	-1.98454
46	C	-4.09874	4.125201	-0.30139	92	H	1.509147	1.726484	4.49638

93	H	1.521237	1.481377	2.731219	127	H	6.005859	1.930428	2.930656
94	H	0.112862	-1.56603	-3.92218	128	H	5.938388	-2.3487	3.230223
95	H	2.332847	-1.48668	-4.9566	129	H	3.508599	-2.26435	3.749755
96	H	2.717572	2.65441	-3.88195	130	H	10.53029	-1.66779	-2.48442
97	H	0.469032	2.585194	-2.84122	131	H	9.268354	-2.78174	-2.98465
98	H	4.503673	1.57778	-5.12659	132	H	-5.91898	-6.09402	-0.58386
99	H	4.254066	-0.00443	-5.87487	133	H	-6.21393	-4.57349	0.255703
100	H	7.802911	0.614755	1.941926	134	H	-7.12196	0.796919	-1.36139
101	H	8.248745	-0.05004	3.506163	135	H	-8.63575	0.933302	-2.2566
102	H	4.696564	-0.96113	-3.6861	136	H	6.674533	-0.34859	-5.16615
103	H	7.38003	-1.79664	1.330411	137	H	6.808269	1.02314	-4.06073
104	H	4.08787	5.218339	-0.25197	138	H	6.936156	-1.95953	-3.36981
105	H	5.555571	3.231954	-0.32837	139	H	6.746777	-0.72029	-2.13711
106	H	-0.71362	5.775967	-0.02178	140	H	9.065244	-0.63756	-4.13243
107	H	1.751671	6.180338	-0.15204	141	H	8.924177	0.245172	-2.62179
108	H	5.809122	0.733309	-0.3391	142	H	-9.74435	-0.59418	-0.58449
109	H	4.727556	-1.51096	-0.30282	143	H	-8.2292	-0.71743	0.297735
110	H	1.409883	-1.98951	-1.15423	144	H	-7.15023	-1.64432	-1.84405
111	H	2.799479	-2.8315	-0.43697	145	H	-8.74622	-1.70227	-2.56398
112	H	-3.8876	3.662703	-2.39072	146	H	-9.53782	-3.25457	-0.78649
113	H	-6.30378	3.164423	-2.69916	147	H	-7.98878	-3.15984	0.029258
114	H	-7.00858	4.064215	1.445625	148	H	-8.37275	-5.25674	-1.3881
115	H	-4.59222	4.565489	1.744701	149	H	-8.38267	-4.24018	-2.81772
116	H	-0.40725	-2.68874	2.756498	150	H	-6.17527	-5.11383	-2.75872
117	H	-2.53322	-3.95803	2.632991	151	H	-5.99396	-3.48214	-2.15474
118	H	-1.57216	-5.16089	-1.3742	152	H	-2.19665	2.920012	1.174463
119	H	0.547958	-3.93427	-1.25222	153	H	-2.15254	2.481688	-0.54331
120	H	-8.61197	3.355148	-1.84739	154	H	9.56421	-3.10442	-0.66278
121	H	-8.90009	3.927733	-0.20454	155	H	8.018796	-2.29696	-0.83583
122	H	-4.14622	-5.13382	1.421642	156	H	9.075236	-0.12854	-0.08625
123	H	-3.49318	-6.38283	0.360218	157	H	10.65543	-0.90282	-0.09959
124	H	-8.41106	1.701553	0.56037	158	H	9.805578	-2.55975	1.634722
125	H	-4.16197	-3.75825	-0.7121	159	H	10.01295	-0.90823	2.1994
126	H	3.624617	2.031021	3.487817					

Table S6. Cartesian coordinates of the complex [Cu(**C8**)]$^+$ at its optimized S_0 geometry.

atom		x	y	z	atom		x	y	z
1	C	-5.21834	0.523858	-0.64918	10	C	-4.42396	-0.86076	1.274603
2	C	-4.93465	1.266409	-1.75654	11	C	-3.23485	2.212303	-3.32948
3	C	-3.57958	1.444598	-2.19482	12	C	-1.90856	2.339435	-3.69126
4	C	-2.5208	0.836755	-1.47275	13	C	-0.91608	1.697453	-2.92174
5	C	-2.82079	0.05277	-0.28805	14	N	-1.22301	0.969504	-1.84519
6	C	-4.17224	-0.09641	0.115289	15	C	-0.86529	-1.63776	2.358249
7	N	-1.79031	-0.4885	0.413016	16	O	-0.28258	-2.81307	1.784622
8	C	-2.04621	-1.17279	1.534342	17	C	0.54668	1.815075	-3.28427
9	C	-3.36356	-1.38771	1.985668	18	O	1.026493	3.0818	-2.80426

19	C	1.038393	-3.07531	2.049931	67	C	2.780854	4.384211	-1.93153
20	C	1.826377	-2.38263	2.975413	68	C	9.129741	2.563377	-0.12142
21	C	3.189038	-2.69268	3.102506	69	C	-6.9553	1.831433	3.161209
22	C	3.785936	-3.7044	2.347604	70	C	-8.84258	-2.2365	-1.0068
23	C	2.962602	-4.42428	1.461982	71	C	7.925922	-0.48249	2.137027
24	C	1.614621	-4.1201	1.308042	72	C	8.507332	0.905014	1.791611
25	C	5.261037	-4.06197	2.4426	73	C	8.722636	1.134736	0.284811
26	C	6.566963	3.892717	-1.70495	74	C	-9.2669	-0.79995	-0.6738
27	N	6.0642	-3.10624	3.208964	75	C	-9.11178	-0.39098	0.80023
28	N	6.768191	3.432572	-0.31813	76	C	-9.29183	1.122243	1.009756
29	C	4.919047	-1.93419	-1.84913	77	C	-9.47261	1.568961	2.472136
30	C	5.283793	-0.95669	-0.97386	78	C	-8.3334	1.207126	3.443629
31	C	4.295767	-0.17762	-0.28134	79	C	-0.64181	-2.66788	-2.91669
32	C	2.917269	-0.41775	-0.50949	80	Cu	0.044705	-0.19917	-0.56528
33	C	2.528065	-1.44014	-1.4644	81	C	8.115199	3.668754	0.223076
34	C	3.534808	-2.19884	-2.11826	82	C	-6.87491	3.351258	3.371592
35	N	1.207069	-1.62073	-1.71709	83	C	8.912786	-1.65232	1.997657
36	C	0.831232	-2.54289	-2.60786	84	C	8.284489	-3.05961	1.990868
37	C	1.766537	-3.35799	-3.27727	85	C	7.493474	-3.4721	3.24967
38	C	3.11418	-3.18704	-3.03337	86	H	-6.24331	0.38858	-0.31934
39	C	4.635592	0.844893	0.629317	87	H	-5.7287	1.738542	-2.32806
40	C	3.635223	1.530532	1.288187	88	H	-3.52889	-1.95929	2.893072
41	C	2.288897	1.210504	1.034179	89	H	-5.44751	-1.01973	1.600057
42	N	1.944859	0.273821	0.141437	90	H	-4.01911	2.693145	-3.90746
43	O	-1.25163	-3.52204	-1.93929	91	H	-1.6186	2.920596	-4.56081
44	C	1.186416	1.865287	1.834418	92	H	-1.18508	-1.84692	3.385865
45	O	0.551259	2.896982	1.069885	93	H	-0.12863	-0.83151	2.378788
46	C	-2.61978	-3.57974	-1.91641	94	H	0.679035	1.756622	-4.37199
47	C	-0.73905	3.224574	1.404727	95	H	1.093839	0.996288	-2.81023
48	C	-3.19488	-4.20506	-0.79917	96	H	1.407909	-1.59723	3.594785
49	C	-4.5803	-4.26704	-0.67002	97	H	3.785626	-2.11303	3.800549
50	C	-5.43032	-3.72743	-1.64838	98	H	3.391752	-5.22787	0.86665
51	C	-4.83849	-3.14673	-2.77643	99	H	0.996972	-4.66521	0.599896
52	C	-3.44986	-3.0652	-2.92183	100	H	5.667377	-4.109	1.424717
53	C	-1.43752	4.018062	0.480671	101	H	5.355455	-5.09049	2.843997
54	C	-2.77763	4.326579	0.699121	102	H	7.216634	3.306082	-2.36445
55	C	-3.46409	3.854026	1.83091	103	H	6.860456	4.952207	-1.82597
56	C	-2.73489	3.124339	2.772253	104	H	5.722229	-3.10944	4.169038
57	C	-1.38239	2.817526	2.57967	105	H	5.667935	-2.52542	-2.3685
58	C	-6.93423	-3.70168	-1.47441	106	H	6.330848	-0.74814	-0.78009
59	C	-4.96912	4.054909	1.953439	107	H	1.415396	-4.10017	-3.98674
60	N	-7.3826	-2.39243	-0.96454	108	H	3.856242	-3.79369	-3.54486
61	N	-5.48533	3.832839	3.305579	109	H	5.676843	1.091292	0.796295
62	C	2.377968	3.247028	-2.65062	110	H	3.871201	2.309113	2.006192
63	C	3.350677	2.373757	-3.15374	111	H	1.598742	2.288404	2.758039
64	C	4.702502	2.608556	-2.87241	112	H	0.453742	1.098619	2.096542
65	C	5.119055	3.708644	-2.11615	113	H	-2.54397	-4.61311	-0.03158
66	C	4.13072	4.605813	-1.67426	114	H	-5.00905	-4.73439	0.213581

115	H	-5.47059	-2.73873	-3.5627	141	H	7.797253	0.877529	-0.24534
116	H	-3.03274	-2.60399	-3.80997	142	H	-10.3167	-0.67054	-0.97041
117	H	-0.92483	4.359124	-0.414	143	H	-8.68776	-0.1141	-1.3115
118	H	-3.3084	4.922717	-0.04089	144	H	-8.12696	-0.70722	1.165911
119	H	-3.23839	2.779376	3.669802	145	H	-9.85385	-0.93786	1.401138
120	H	-0.85667	2.252894	3.341949	146	H	-10.1777	1.452703	0.447553
121	H	-7.24514	-4.47345	-0.75916	147	H	-8.43891	1.648952	0.55781
122	H	-7.41999	-3.94596	-2.43765	148	H	-10.3991	1.119298	2.858291
123	H	-5.45576	3.33041	1.287672	149	H	-9.63719	2.655219	2.487359
124	H	-5.23904	5.048141	1.55521	150	H	-8.64421	1.493032	4.45927
125	H	-6.98048	-1.67677	-1.57127	151	H	-8.21749	0.115179	3.464205
126	H	3.073216	1.509614	-3.74729	152	H	-0.77289	-3.09108	-3.91938
127	H	5.442619	1.904621	-3.24627	153	H	-1.10435	-1.67618	-2.88553
128	H	4.420795	5.485006	-1.10232	154	H	9.655329	-1.59311	2.80785
129	H	2.023205	5.071896	-1.56688	155	H	9.478938	-1.54203	1.062326
130	H	9.321956	2.574188	-1.20351	156	H	9.096699	-3.78587	1.85042
131	H	10.08298	2.831666	0.357223	157	H	7.636448	-3.16145	1.109481
132	H	-6.63306	1.58071	2.141821	158	H	7.930595	-2.98562	4.130353
133	H	-6.22057	1.371368	3.835576	159	H	7.61238	-4.56044	3.403108
134	H	-9.28661	-2.93949	-0.28743	160	H	-5.4049	4.697587	3.835503
135	H	-9.24842	-2.5097	-1.99954	161	H	-7.2491	3.587985	4.375473
136	H	7.05585	-0.66736	1.494029	162	H	-7.53369	3.875379	2.656946
137	H	7.536767	-0.46234	3.163474	163	H	6.098199	3.928512	0.266914
138	H	9.455419	1.058559	2.329757	164	H	8.526801	4.6384	-0.1137
139	H	7.815382	1.666055	2.175503	165	H	8.012479	3.737328	1.31178
140	H	9.490444	0.442181	-0.08586					

Table S7. Cartesian coordinates of the complex [Cu(**C9**)]$^+$ at its optimized S_0 geometry.

atom		x	y	z	atom		x	y	z
1	C	5.160492	-0.98463	-1.04082	18	O	-1.34407	-2.95704	-2.5096
2	C	4.780019	-1.70471	-2.13223	19	C	-0.83931	2.964304	1.689106
3	C	3.39842	-1.79412	-2.50931	20	C	-1.53333	2.448429	2.785608
4	C	2.412594	-1.12229	-1.74126	21	C	-2.84923	2.866685	3.032642
5	C	2.819421	-0.34662	-0.58458	22	C	-3.47902	3.816821	2.228262
6	C	4.194146	-0.28582	-0.24053	23	C	-2.74403	4.355434	1.156766
7	N	1.860681	0.277873	0.147823	24	C	-1.44829	3.937711	0.881374
8	C	2.21335	0.980953	1.228799	25	C	-4.90982	4.277118	2.448833
9	C	3.557589	1.102748	1.628461	26	C	-6.92295	-4.1749	-2.37384
10	C	4.544663	0.470083	0.897866	27	N	-5.55242	3.669319	3.612741
11	C	2.956384	-2.53923	-3.6229	28	N	-7.51569	-4.18626	-1.02842
12	C	1.607825	-2.59336	-3.91436	29	C	-5.06026	1.403546	-1.58912
13	C	0.69302	-1.89833	-3.09758	30	C	-5.30805	0.404916	-0.69569
14	N	1.092189	-1.18149	-2.0446	31	C	-4.23491	-0.29816	-0.05136
15	C	1.101602	1.581994	2.059811	32	C	-2.89215	0.028784	-0.36904
16	O	0.424966	2.58127	1.300864	33	C	-2.63042	1.07708	-1.33558
17	C	-0.79106	-1.96434	-3.37757	34	C	-3.71504	1.771835	-1.92486

35	N	-1.33727	1.367849	-1.63015
36	C	-1.06421	2.349418	-2.48964
37	C	-2.08421	3.103925	-3.10852
38	C	-3.40308	2.811802	-2.83069
39	C	-4.44295	-1.31137	0.907858
40	C	-3.35824	-1.9137	1.513673
41	C	-2.05536	-1.51896	1.15532
42	N	-1.83546	-0.58461	0.225136
43	O	0.679645	4.011366	-2.6158
44	C	-0.85249	-2.07609	1.884055
45	O	-0.09228	-2.91344	1.014827
46	C	2.025468	4.252298	-2.35592
47	C	1.212208	-3.18861	1.356359
48	C	2.502645	4.106055	-1.04846
49	C	3.856224	4.293198	-0.78981
50	C	4.753912	4.632572	-1.8138
51	C	4.248703	4.823669	-3.1041
52	C	2.888482	4.632127	-3.38299
53	C	1.924547	-3.98363	0.444535
54	C	3.265448	-4.27304	0.670464
55	C	3.945544	-3.77371	1.795161
56	C	3.21373	-3.01013	2.706632
57	C	1.854822	-2.72832	2.509239
58	C	6.239799	4.654834	-1.50783
59	C	5.430418	-4.05122	1.964854
60	N	6.702627	3.271557	-1.34342
61	N	5.925802	-3.70894	3.297039
62	C	-2.70072	-3.16613	-2.52802
63	C	-3.59991	-2.52479	-3.38523
64	C	-4.96533	-2.83516	-3.30593
65	C	-5.457	-3.77148	-2.39405
66	C	-4.5347	-4.3978	-1.53784
67	C	-3.17817	-4.1061	-1.59965
68	C	-9.6537	-1.84459	1.159954
69	C	8.275003	-1.70969	3.216983
70	C	7.985372	3.082904	-0.661
71	C	-7.48168	1.180807	2.552577
72	C	-8.08748	-0.23469	2.460413
73	C	-9.03491	-0.43795	1.266108
74	C	8.305522	1.581918	-0.61805
75	C	9.247439	1.123617	0.502668
76	C	9.432498	-0.40317	0.4887
77	C	10.25325	-0.98335	1.65278
78	C	9.591954	-0.92534	3.043908
79	C	0.391525	2.612537	-2.78956
80	Cu	-0.03607	-0.01284	-0.64108
81	C	-8.70372	-2.99811	0.786366
82	C	-8.10718	-2.90572	-0.62554
83	C	8.372144	-3.20301	2.858751
84	C	7.322612	-4.09784	3.537451
85	C	-8.47017	2.255242	3.034116
86	C	-7.99638	3.714257	2.899106
87	C	-6.90357	4.194863	3.869063
88	H	6.206227	-0.92781	-0.75668
89	H	5.516221	-2.22958	-2.73364
90	H	3.804482	1.684869	2.509657
91	H	5.58611	0.544553	1.195215
92	H	3.679715	-3.06621	-4.23824
93	H	1.24373	-3.16358	-4.76229
94	H	1.502763	2.01428	2.983913
95	H	0.404876	0.78121	2.329215
96	H	-0.96701	-2.23562	-4.42545
97	H	-1.25023	-0.98918	-3.18212
98	H	-1.08234	1.722569	3.452205
99	H	-3.387	2.424171	3.864829
100	H	-3.20214	5.107042	0.517153
101	H	-0.8999	4.336633	0.033425
102	H	-5.49781	4.014338	1.56021
103	H	-4.92482	5.38452	2.495423
104	H	-7.48494	-3.52998	-3.07248
105	H	-7.00526	-5.19674	-2.76678
106	H	-4.98563	3.884083	4.43231
107	H	-5.87858	1.937494	-2.06298
108	H	-6.32841	0.130922	-0.44919
109	H	-1.81692	3.899624	-3.79508
110	H	-4.2067	3.370807	-3.30128
111	H	-5.45361	-1.60443	1.171947
112	H	-3.49749	-2.68216	2.266234
113	H	-1.17202	-2.64138	2.767564
114	H	-0.24144	-1.23169	2.218505
115	H	1.816269	3.823985	-0.25624
116	H	4.232769	4.148039	0.219566
117	H	4.923878	5.099372	-3.91109
118	H	2.501607	4.75768	-4.39008
119	H	1.413104	-4.35727	-0.43744
120	H	3.799645	-4.88919	-0.04962
121	H	3.707815	-2.61384	3.587829
122	H	1.328345	-2.14766	3.257741
123	H	6.414736	5.173296	-0.55675
124	H	6.7895	5.216069	-2.28325
125	H	5.988919	-3.45399	1.230233
126	H	5.628968	-5.1083	1.699798
127	H	6.734449	2.823991	-2.25863
128	H	-3.26208	-1.79262	-4.11018
129	H	-5.65396	-2.33298	-3.98207
130	H	-4.89643	-5.12058	-0.81182

131	H	-2.46801	-4.59154	-0.93671	152	H	10.31809	-1.3109	3.77421
132	H	-10.462	-1.81442	0.415601	153	H	9.414406	0.121992	3.324353
133	H	-10.1349	-2.08775	2.117773	154	H	0.627798	2.319804	-3.82356
134	H	7.471984	-1.24327	2.632247	155	H	1.004794	2.010959	-2.11326
135	H	7.966637	-1.61473	4.267689	156	H	-9.27788	-3.93399	0.850849
136	H	7.874162	3.471597	0.360937	157	H	-7.89238	-3.09381	1.519475
137	H	8.821262	3.642187	-1.12145	158	H	9.354256	-3.59005	3.167199
138	H	-7.09311	1.46805	1.564185	159	H	8.332282	-3.33352	1.769247
139	H	-6.61461	1.171322	3.2212	160	H	-8.73544	2.056813	4.083687
140	H	-8.6271	-0.46443	3.391738	161	H	-9.4061	2.162927	2.466176
141	H	-7.26963	-0.96547	2.404164	162	H	-8.86322	4.367376	3.070127
142	H	-9.857	0.28679	1.331806	163	H	-7.68824	3.903205	1.861465
143	H	-8.50299	-0.19539	0.334224	164	H	-7.17857	3.891736	4.887216
144	H	8.705413	1.263443	-1.59174	165	H	-6.90481	5.301852	3.857568
145	H	7.346984	1.061997	-0.49687	166	H	7.477942	-4.04779	4.622385
146	H	8.832072	1.444372	1.468086	167	H	7.51152	-5.14618	3.236391
147	H	10.22717	1.61403	0.408657	168	H	5.349406	-4.19857	3.980088
148	H	9.916098	-0.68641	-0.45684	169	H	-8.25284	-4.88858	-1.01183
149	H	8.442073	-0.88109	0.471395	170	H	-7.31729	-2.14591	-0.66478
150	H	11.21738	-0.45723	1.705602	171	H	-8.88954	-2.57329	-1.33575
151	H	10.49649	-2.02853	1.418988					

Table S8. Cartesian coordinates of the complex [Cu(**C10**)]$^+$ at its optimized S_0 geometry.

atom		x	y	z	atom		x	y	z
1	C	5.302654	-0.9033	-0.92898	23	C	-2.65675	4.437116	1.112056
2	C	4.968226	-1.63803	-2.02554	24	C	-1.40631	3.927996	0.77652
3	C	3.59891	-1.76201	-2.43687	25	C	-4.62704	4.711187	2.689002
4	C	2.576304	-1.11225	-1.6974	26	C	-6.85221	-3.29495	-2.27961
5	C	2.931777	-0.32862	-0.53044	27	N	-5.36339	3.834178	3.601212
6	C	4.296781	-0.23089	-0.15591	28	N	-7.28558	-3.96581	-1.05393
7	N	1.93955	0.277139	0.173734	29	C	-5.03593	1.363029	-1.35161
8	C	2.253508	0.999502	1.254135	30	C	-5.24197	0.408886	-0.4015
9	C	3.584913	1.154584	1.685144	31	C	-4.14068	-0.29634	0.190208
10	C	4.602585	0.537982	0.985594	32	C	-2.81519	-0.01903	-0.23349
11	C	3.20632	-2.51416	-3.56419	33	C	-2.59815	0.986039	-1.25518
12	C	1.870382	-2.58154	-3.90616	34	C	-3.70964	1.679371	-1.79869
13	C	0.919418	-1.89652	-3.12357	35	N	-1.32511	1.225344	-1.66012
14	N	1.268178	-1.18809	-2.04659	36	C	-1.09973	2.147143	-2.59821
15	C	1.120805	1.613093	2.043733	37	C	-2.14619	2.893389	-3.17859
16	O	0.477702	2.598354	1.240231	38	C	-3.44663	2.659077	-2.78132
17	C	-0.54684	-1.93255	-3.48114	39	C	-4.31066	-1.27168	1.194432
18	O	-1.16386	-2.97953	-2.72663	40	C	-3.20284	-1.9009	1.726338
19	C	-0.72732	3.094466	1.677013	41	C	-1.91934	-1.56402	1.25465
20	C	-1.2948	2.815559	2.924757	42	N	-1.73509	-0.65133	0.295365
21	C	-2.54976	3.345326	3.246036	43	O	0.803356	3.549201	-2.35206
22	C	-3.25773	4.150017	2.348622	44	C	-0.68988	-2.19787	1.862732

45	O	-0.0648	-3.02574	0.885052	93	H	5.635162	0.637431	1.304542
46	C	2.144517	3.835755	-2.43387	94	H	3.958087	-3.02974	-4.15457
47	C	1.20753	-3.47649	1.143147	95	H	1.543963	-3.14899	-4.7711
48	C	2.582722	4.938548	-1.68884	96	H	1.506191	2.061033	2.967171
49	C	3.929667	5.294726	-1.7002	97	H	0.403054	0.828742	2.315307
50	C	4.870318	4.572702	-2.44851	98	H	-0.67532	-2.11623	-4.55506
51	C	4.41292	3.477937	-3.18975	99	H	-0.99673	-0.96776	-3.22947
52	C	3.06664	3.104312	-3.1944	100	H	-0.78144	2.195721	3.651203
53	C	1.837077	-4.15359	0.089246	101	H	-2.99257	3.116193	4.210143
54	C	3.143609	-4.60712	0.237848	102	H	-3.1827	5.060716	0.392379
55	C	3.853669	-4.41509	1.43396	103	H	-0.95087	4.139794	-0.18575
56	C	3.199731	-3.76389	2.483766	104	H	-5.16147	4.921479	1.74476
57	C	1.887621	-3.29435	2.352317	105	H	-4.51992	5.684343	3.192451
58	C	6.333041	4.981639	-2.49221	106	H	-7.26265	-2.2754	-2.39744
59	C	5.271932	-4.93615	1.575883	107	H	-7.26158	-3.87589	-3.11993
60	N	7.243597	3.851658	-2.27036	108	H	-5.40357	2.901295	3.196099
61	N	6.081864	-4.13125	2.492387	109	H	-6.89926	-3.47919	-0.2456
62	C	-2.53652	-3.02077	-2.69049	110	H	-5.87264	1.897669	-1.79127
63	C	-3.37662	-2.16241	-3.41066	111	H	-6.2472	0.164674	-0.07254
64	C	-4.76158	-2.26338	-3.25108	112	H	-1.9176	3.636241	-3.93534
65	C	-5.3407	-3.21748	-2.40845	113	H	-4.27067	3.213895	-3.22009
66	C	-4.48285	-4.09257	-1.72487	114	H	-5.30942	-1.51583	1.544356
67	C	-3.09948	-3.99555	-1.85542	115	H	-3.30793	-2.65069	2.502968
68	C	-10.2369	-1.31178	1.495823	116	H	-0.96606	-2.78774	2.744557
69	C	7.938441	-1.42198	3.180999	117	H	0.001926	-1.4078	2.179048
70	C	7.380637	3.482145	-0.8582	118	H	1.860511	5.496205	-1.10005
71	C	-7.70301	1.550487	2.568436	119	H	4.255262	6.149085	-1.11084
72	C	-8.48052	0.220785	2.571034	120	H	5.126029	2.899307	-3.76985
73	C	-9.48381	0.031978	1.421257	121	H	2.755047	2.254423	-3.79074
74	C	8.282299	2.256988	-0.68341	122	H	1.296034	-4.2909	-0.842
75	C	8.540384	1.9056	0.788462	123	H	3.62543	-5.11129	-0.59689
76	C	9.347086	0.610831	0.973417	124	H	3.729021	-3.61119	3.419214
77	C	9.74629	0.314385	2.430865	125	H	1.417725	-2.79744	3.193598
78	C	8.592702	-0.04146	3.389021	126	H	6.495544	5.804981	-1.77254
79	C	0.331882	2.374393	-3.01848	127	H	6.564055	5.388376	-3.48556
80	Cu	0.050218	-0.07824	-0.64886	128	H	5.72123	-5.026	0.56881
81	C	-9.43899	-2.54987	1.033812	129	H	5.250029	-5.95895	1.9819
82	C	-9.50788	-2.80893	-0.48066	130	H	8.16341	4.108941	-2.62529
83	C	-8.74449	-4.07735	-0.92049	131	H	6.119823	-3.17674	2.138605
84	C	8.901646	-2.60237	3.411408	132	H	-2.97539	-1.41257	-4.08249
85	C	8.255108	-3.95938	3.747661	133	H	-5.40034	-1.57395	-3.79892
86	C	7.443555	-4.6561	2.645079	134	H	-4.90987	-4.84826	-1.0731
87	C	-8.56138	2.794386	2.848418	135	H	-2.43866	-4.6607	-1.30732
88	C	-7.81044	4.140248	2.840051	136	H	-11.1562	-1.2486	0.897174
89	C	-6.7199	4.296096	3.928401	137	H	-10.5659	-1.45991	2.533812
90	H	6.339175	-0.81161	-0.61878	138	H	7.503164	-1.4812	2.172995
91	H	5.732063	-2.14384	-2.60854	139	H	7.096482	-1.50517	3.882263
92	H	3.797018	1.752602	2.564599	140	H	6.378521	3.255776	-0.47188

141	H	7.767099	4.320724	-0.24669	160	H	-9.83278	-3.44009	1.543835
142	H	-7.18412	1.674147	1.606442	161	H	-8.39128	-2.46077	1.356717
143	H	-6.91966	1.472135	3.334618	162	H	-9.13906	-1.93827	-1.04089
144	H	-9.01823	0.127439	3.526527	163	H	-10.5646	-2.92582	-0.76051
145	H	-7.75447	-0.60397	2.5545	164	H	-8.96056	-4.88033	-0.20122
146	H	-10.2187	0.846769	1.442861	165	H	-9.12637	-4.4203	-1.88999
147	H	-8.96273	0.118715	0.455693	166	H	9.564329	-2.34328	4.250019
148	H	9.245551	2.43934	-1.18322	167	H	9.557758	-2.72667	2.538784
149	H	7.82294	1.404547	-1.20212	168	H	7.611903	-3.85455	4.632365
150	H	7.57897	1.828282	1.314889	169	H	9.065028	-4.64438	4.033026
151	H	9.078861	2.735952	1.26855	170	H	7.338699	-5.71486	2.916474
152	H	10.26443	0.682819	0.371947	171	H	8.013532	-4.6393	1.695729
153	H	8.782265	-0.23557	0.556265	172	H	-9.05233	2.671112	3.825762
154	H	10.26797	1.196088	2.829842	173	H	-9.37016	2.851637	2.108177
155	H	10.48488	-0.49801	2.439451	174	H	-8.55397	4.936762	2.980003
156	H	8.977803	-0.005	4.41813	175	H	-7.36736	4.314356	1.849222
157	H	7.817338	0.734471	3.33433	176	H	-7.04742	3.768806	4.835705
158	H	0.394621	2.511266	-4.10599	177	H	-6.63055	5.353982	4.205268
159	H	0.930871	1.504184	-2.73447					

Table S9. Cartesian coordinates of the complex [Cu(**C11**)]$^+$ at its optimized S_0 geometry.

atom		x	y	z	atom		x	y	z
1	C	-5.38598	-0.8177	0.494283	25	C	4.550803	5.181117	-2.38893
2	C	-5.1406	-1.6375	1.55311	26	C	6.513346	-4.36403	2.402931
3	C	-3.79747	-1.89619	1.987515	27	N	5.429904	4.425238	-3.28279
4	C	-2.70425	-1.30126	1.304546	28	N	7.22502	-3.71707	1.292258
5	C	-2.96605	-0.43146	0.174824	29	C	5.048676	0.949087	1.015318
6	C	-4.30859	-0.19126	-0.21833	30	C	5.23079	0.040168	0.0169
7	N	-1.90943	0.132825	-0.46537	31	C	4.11486	-0.64435	-0.57118
8	C	-2.13422	0.955803	-1.49407	32	C	2.799365	-0.38882	-0.10227
9	C	-3.43592	1.249119	-1.9442	33	C	2.609465	0.571605	0.966038
10	C	-4.52001	0.674154	-1.31181	34	C	3.734164	1.242064	1.509963
11	C	-3.50057	-2.72373	3.090156	35	N	1.344844	0.803801	1.40363
12	C	-2.18511	-2.90868	3.465781	36	C	1.142946	1.696957	2.372242
13	C	-1.15929	-2.26993	2.740799	37	C	2.204965	2.414931	2.962256
14	N	-1.4157	-1.49496	1.683761	38	C	3.495308	2.185908	2.534353
15	C	-0.93158	1.572652	-2.1659	39	C	4.262153	-1.57446	-1.62074
16	O	-0.39475	2.554295	-1.27955	40	C	3.141949	-2.17963	-2.15282
17	C	0.283376	-2.43791	3.15355	41	C	1.869181	-1.86342	-1.63792
18	O	0.918129	-3.28689	2.197915	42	N	1.705118	-0.99604	-0.63428
19	C	0.791365	3.153055	-1.62726	43	O	-0.6383	3.265582	2.519551
20	C	1.440618	2.976738	-2.85391	44	C	0.632176	-2.47781	-2.25023
21	C	2.653731	3.632558	-3.09233	45	O	0.019346	-3.33182	-1.28698
22	C	3.241005	4.459576	-2.13039	46	C	-1.93486	3.647783	2.770454
23	C	2.568523	4.625121	-0.90878	47	C	-1.23498	-3.81556	-1.56678
24	C	1.35706	3.990497	-0.65511	48	C	-2.27758	4.949708	2.382102

49	C	-3.57511	5.415388	2.58098	97	H	-0.17898	0.799739	-2.36565
50	C	-4.55844	4.609198	3.172828	98	H	0.336383	-2.88162	4.154639
51	C	-4.19608	3.314966	3.558755	99	H	0.780148	-1.46055	3.175526
52	C	-2.90114	2.826306	3.365114	100	H	1.020686	2.343336	-3.62726
53	C	-1.88912	-4.46133	-0.50754	101	H	3.158395	3.489479	-4.0427
54	C	-3.17984	-4.94807	-0.68403	102	H	3.005227	5.256754	-0.1382
55	C	-3.84636	-4.82324	-1.91342	103	H	0.845725	4.109502	0.295205
56	C	-3.16216	-4.21202	-2.96836	104	H	5.010456	5.426901	-1.4146
57	C	-1.86736	-3.70493	-2.81015	105	H	4.353555	6.148669	-2.87576
58	C	-5.96179	5.135245	3.426287	106	H	6.927783	-4.08902	3.390292
59	C	-5.28586	-5.26183	-2.07332	107	H	6.669878	-5.44656	2.29549
60	N	-7.00164	4.147212	3.120322	108	H	5.582124	3.498977	-2.88849
61	N	-6.19688	-4.12832	-1.85064	109	H	5.895478	1.468635	1.453814
62	C	2.267961	-3.49759	2.320141	110	H	6.225873	-0.17913	-0.35857
63	C	3.048963	-3.04713	3.388464	111	H	1.992748	3.134752	3.745369
64	C	4.420797	-3.33219	3.403113	112	H	4.331194	2.720336	2.976218
65	C	5.031545	-4.0577	2.378948	113	H	5.252768	-1.80345	-2.00201
66	C	4.226299	-4.49804	1.313667	114	H	3.22808	-2.8951	-2.96341
67	C	2.864278	-4.22838	1.27935	115	H	0.904098	-3.04402	-3.1484
68	C	10.86379	0.239734	-0.58204	116	H	-0.06735	-1.6837	-2.54137
69	C	-8.39693	-0.04521	-2.82779	117	H	-1.52263	5.576319	1.91655
70	C	-7.26972	4.006772	1.685872	118	H	-3.82818	6.424922	2.264508
71	C	8.011907	2.493572	-2.16026	119	H	-4.94671	2.671395	4.007779
72	C	9.001206	1.320534	-2.01224	120	H	-2.66473	1.816422	3.679901
73	C	9.710682	1.257639	-0.64992	121	H	-1.37709	-4.55351	0.445066
74	C	-8.33028	2.936889	1.414196	122	H	-3.68426	-5.4267	0.151937
75	C	-8.67163	2.798268	-0.07577	123	H	-3.64344	-4.12027	-3.93983
76	C	-9.67431	1.670866	-0.36594	124	H	-1.3722	-3.23907	-3.65449
77	C	-10.156	1.60242	-1.82767	125	H	-6.09414	6.079391	2.866055
78	C	-9.06146	1.343235	-2.88076	126	H	-6.06518	5.390641	4.489505
79	C	-0.27711	1.918775	2.83189	127	H	-5.526	-6.03251	-1.33011
80	Cu	-0.06608	-0.43782	0.374365	128	H	-5.43226	-5.72531	-3.06694
81	C	10.48997	-1.23895	-0.78896	129	H	-7.86236	4.43605	3.582688
82	C	9.603096	-1.84287	0.310857	130	H	2.614046	-2.48599	4.207613
83	C	9.41624	-3.35959	0.146892	131	H	5.019185	-2.97929	4.240006
84	C	-9.27014	-1.18908	-3.36931	132	H	4.680755	-5.05635	0.49944
85	C	-8.56688	-2.55889	-3.36952	133	H	2.242106	-4.56309	0.45502
86	C	-8.50932	-3.24686	-1.99394	134	H	11.36454	0.339699	0.391829
87	C	8.694539	3.858602	-2.34932	135	H	11.61281	0.517325	-1.3377
88	C	7.760392	5.08183	-2.39807	136	H	-8.08348	-0.26911	-1.79893
89	C	6.721966	5.074703	-3.54601	137	H	-7.47353	-0.01353	-3.4239
90	H	-6.40376	-0.61858	0.17284	138	H	-6.3299	3.721607	1.195993
91	H	-5.95774	-2.1076	2.091958	139	H	-7.58119	4.965403	1.227009
92	H	-3.57354	1.924158	-2.78188	140	H	7.34918	2.524633	-1.28201
93	H	-5.53217	0.883095	-1.64498	141	H	7.368581	2.290097	-3.02726
94	H	-4.30806	-3.20289	3.635797	142	H	9.75407	1.387077	-2.81239
95	H	-1.9308	-3.53591	4.313317	143	H	8.461765	0.379493	-2.18284
96	H	-1.22443	2.03176	-3.11722	144	H	10.12369	2.245556	-0.4065

145	H	8.968418	1.04264	0.13229	165	H	-9.08334	-3.23348	-4.06596
146	H	-9.24529	3.180717	1.975003	166	H	9.281486	3.826498	-3.27967
147	H	-7.97205	1.976176	1.808762	167	H	9.41992	4.017962	-1.54048
148	H	-7.74356	2.62695	-0.63859	168	H	8.388665	5.976889	-2.50348
149	H	-9.08046	3.750724	-0.44486	169	H	7.241205	5.192131	-1.43572
150	H	-10.5546	1.806328	0.278627	170	H	7.168697	4.594712	-4.42817
151	H	-9.23224	0.709507	-0.06809	171	H	6.496989	6.10758	-3.84023
152	H	-10.6523	2.552198	-2.07352	172	C	8.654396	-4.04505	1.286262
153	H	-10.932	0.828871	-1.9074	173	H	8.903396	-3.57298	-0.80109
154	H	-9.49721	1.483023	-3.88049	174	H	10.40867	-3.82647	0.078691
155	H	-8.28479	2.113266	-2.786	175	H	9.14579	-3.80395	2.249668
156	H	-0.35403	1.75431	3.915699	176	H	8.73431	-5.13385	1.16555
157	H	-0.93778	1.211782	2.321731	177	H	7.112458	-2.70856	1.392234
158	H	11.42183	-1.82037	-0.83842	178	C	-7.61355	-4.49324	-1.96499
159	H	10.00252	-1.37252	-1.76476	179	H	-9.52916	-3.5312	-1.70323
160	H	8.626064	-1.33999	0.316649	180	H	-8.14752	-2.551	-1.22554
161	H	10.05904	-1.64244	1.292278	181	H	-7.81946	-5.11302	-2.85995
162	H	-9.56178	-0.93814	-4.39885	182	H	-7.86557	-5.11446	-1.09489
163	H	-10.2057	-1.2614	-2.79659	183	H	-5.98085	-3.41579	-2.54752
164	H	-7.552	-2.42958	-3.77487					

Table S10. Cartesian coordinates of the complex [Cu(**C12**)]$^{+}$ at its optimized S_0 geometry.

atom		x	y	z	atom		x	y	z
1	C	-4.83309	-1.21513	1.14902	24	C	2.654649	-5.08492	1.287565
2	C	-5.07166	-0.33837	0.133297	25	C	6.472062	-5.05686	1.367903
3	C	-3.98983	0.272502	-0.58573	26	C	4.911038	5.545465	-2.42924
4	C	-2.65015	-0.02531	-0.22803	27	N	7.433323	-4.31034	2.179947
5	C	-2.39726	-0.95303	0.855677	28	N	5.489779	5.715887	-1.08976
6	C	-3.49113	-1.54829	1.533633	29	C	5.540511	0.207901	-0.30485
7	N	-1.10774	-1.2154	1.188342	30	C	5.379538	1.096666	0.713936
8	C	-0.84655	-2.06353	2.185137	31	C	4.071099	1.422214	1.204938
9	C	-1.8754	-2.70887	2.902129	32	C	2.925973	0.811951	0.629317
10	C	-3.19219	-2.45159	2.577884	33	C	3.097204	-0.12589	-0.46376
11	C	-4.19237	1.171309	-1.655	34	C	4.407776	-0.42077	-0.92132
12	C	-3.1017	1.717403	-2.30092	35	N	1.991264	-0.69297	-1.0132
13	C	-1.80143	1.373984	-1.87958	36	C	2.137386	-1.55747	-2.02299
14	N	-1.58653	0.52568	-0.87001	37	C	3.403881	-1.90613	-2.53069
15	C	0.606255	-2.30744	2.517868	38	C	4.536727	-1.34074	-1.98191
16	O	0.986697	-3.56947	1.962349	39	C	3.86057	2.325489	2.267477
17	C	-0.58795	1.966246	-2.55539	40	C	2.576887	2.555614	2.719692
18	O	-0.25343	3.178488	-1.87272	41	C	1.495832	1.884237	2.114554
19	C	2.328332	-3.86147	1.893249	42	N	1.670139	1.048277	1.086831
20	C	3.352147	-3.03725	2.370359	43	O	0.314426	-3.06239	-1.70893
21	C	4.689014	-3.42412	2.20453	44	C	0.098546	2.044431	2.665859
22	C	5.031831	-4.63134	1.592663	45	O	-0.68241	2.822103	1.759832
23	C	3.985803	-5.45966	1.14944	46	C	-0.94676	-3.5326	-1.98081

47	C	-2.0152	2.982197	2.054416	95	H	-6.08856	-0.08872	-0.15442
48	C	-1.58717	-4.20913	-0.93243	96	H	-1.62046	-3.39312	3.704368
49	C	-2.89142	-4.66481	-1.09691	97	H	-4.00161	-2.93056	3.120887
50	C	-3.58181	-4.47947	-2.30494	98	H	-5.20352	1.42173	-1.96208
51	C	-2.90566	-3.85197	-3.35578	99	H	-3.23042	2.406853	-3.12841
52	C	-1.59893	-3.37507	-3.20877	100	H	0.757919	-2.31929	3.605405
53	C	-2.77538	3.684641	1.109558	101	H	1.203843	-1.50063	2.085186
54	C	-4.13945	3.878361	1.320249	102	H	-0.79765	2.173844	-3.61193
55	C	-4.7793	3.387881	2.467943	103	H	0.242436	1.255412	-2.48926
56	C	-4.00165	2.696451	3.402611	104	H	3.135582	-2.09843	2.866865
57	C	-2.63316	2.490854	3.211929	105	H	5.475785	-2.76565	2.558509
58	C	-5.05249	-4.8114	-2.43657	106	H	4.218941	-6.41354	0.680977
59	C	-6.26134	3.617292	2.714298	107	H	1.852851	-5.72546	0.932474
60	N	-5.83791	-3.60589	-2.13321	108	H	6.728419	-4.89981	0.31101
61	N	-6.91169	2.409606	3.227004	109	H	6.549286	-6.14995	1.52963
62	C	0.985285	3.725454	-2.09916	110	H	5.521528	4.914205	-3.09701
63	C	1.884255	3.281412	-3.07429	111	H	4.873792	6.539731	-2.90012
64	C	3.143153	3.886151	-3.17435	112	H	7.163443	-4.41355	3.157299
65	C	3.523522	4.939893	-2.33956	113	H	6.530868	-0.04507	-0.67021
66	C	2.589914	5.39467	-1.3918	114	H	6.238677	1.570418	1.178666
67	C	1.33987	4.798796	-1.26715	115	H	3.475285	-2.61524	-3.34818
68	C	10.95897	1.424806	-0.72771	116	H	5.523641	-1.59482	-2.35692
69	C	-11.4469	-0.05101	0.171695	117	H	4.709873	2.821658	2.727805
70	C	-7.28231	-3.73361	-2.3471	118	H	2.390606	3.235579	3.543896
71	C	9.151886	-1.75674	0.837619	119	H	0.143441	2.535202	3.645228
72	C	9.793126	-0.35734	0.762563	120	H	-0.35298	1.053349	2.793115
73	C	10.40819	-0.01084	-0.60369	121	H	-1.05774	-4.33883	0.006272
74	C	-7.9167	-2.34047	-2.32175	122	H	-3.38999	-5.15636	-0.26492
75	C	-9.44951	-2.30344	-2.3289	123	H	-3.40777	-3.71391	-4.31116
76	C	-9.96502	-0.86556	-2.49967	124	H	-1.11324	-2.88836	-4.04681
77	C	-11.4847	-0.67822	-2.3638	125	H	-2.28786	4.048081	0.210135
78	C	-12.0676	-0.90072	-0.95443	126	H	-4.71802	4.415717	0.571886
79	C	0.888225	-2.14217	-2.63704	127	H	-4.48081	2.298391	4.291804
80	Cu	0.234906	-0.08591	-0.02416	128	H	-2.07026	1.953223	3.9661
81	C	9.948348	2.562102	-0.47973	129	H	-5.33558	-5.59604	-1.72361
82	C	8.710498	2.525964	-1.38868	130	H	-5.26451	-5.20642	-3.44794
83	C	7.645034	3.584964	-1.04868	131	H	-6.72055	4.022278	1.795784
84	C	-11.4494	1.463542	-0.09207	132	H	-6.38877	4.394285	3.482408
85	C	-11.1035	2.327275	1.133356	133	H	-5.49326	-2.8585	-2.73598
86	C	-9.74298	2.033126	1.786121	134	H	1.630738	2.470623	-3.74776
87	C	10.1532	-2.92028	0.798334	135	H	3.841991	3.514219	-3.92013
88	C	9.538486	-4.33183	0.761698	136	H	2.862293	6.209922	-0.7283
89	C	8.813752	-4.80217	2.037061	137	H	0.630178	5.137252	-0.5181
90	C	-9.39606	3.037264	2.896686	138	H	11.38805	1.542592	-1.73324
91	C	-8.23948	2.598672	3.816219	139	H	11.79599	1.546616	-0.02521
92	C	7.902352	4.989367	-1.61341	140	H	-10.4193	-0.38744	0.362125
93	C	6.919337	6.048602	-1.06105	141	H	-12.0037	-0.25403	1.0983
94	H	-5.65576	-1.67783	1.685833	142	H	-7.69981	-4.36078	-1.54638

143	H	-7.52823	-4.23702	-3.3021	167	H	6.674016	3.23303	-1.42028
144	H	8.439583	-1.86784	0.005376	168	H	-12.4431	1.762726	-0.45664
145	H	8.553758	-1.83365	1.752412	169	H	-10.7463	1.697436	-0.90365
146	H	10.56129	-0.26074	1.545149	170	H	-11.8885	2.203672	1.894321
147	H	9.02014	0.383245	1.007512	171	H	-11.1345	3.385288	0.835267
148	H	11.22904	-0.70574	-0.82474	172	H	-8.95613	2.037187	1.017204
149	H	9.654591	-0.18474	-1.38479	173	H	-9.75293	1.018874	2.20991
150	H	-7.54064	-1.77501	-3.18831	174	H	10.82365	-2.84608	1.668475
151	H	-7.54915	-1.81387	-1.43041	175	H	10.79632	-2.81587	-0.08609
152	H	-9.82699	-2.739	-1.39368	176	H	10.35144	-5.04636	0.573766
153	H	-9.84114	-2.93028	-3.14333	177	H	8.864226	-4.41717	-0.10172
154	H	-9.65636	-0.50608	-3.49161	178	H	9.37586	-4.45553	2.913191
155	H	-9.45133	-0.21637	-1.77669	179	H	8.839701	-5.90799	2.064628
156	H	-11.9958	-1.3551	-3.06356	180	H	-10.28	3.181599	3.535574
157	H	-11.7364	0.338257	-2.69449	181	H	-9.17906	4.019254	2.453376
158	H	-13.1463	-0.69244	-0.99947	182	H	7.849728	4.949776	-2.71012
159	H	-11.9781	-1.9607	-0.67988	183	H	8.920909	5.320972	-1.36901
160	H	1.139144	-2.64933	-3.57566	184	H	-6.94386	1.701895	2.495145
161	H	0.172225	-1.33988	-2.85446	185	H	-8.51816	1.648043	4.292266
162	H	10.46961	3.521805	-0.60042	186	H	-8.13054	3.333015	4.625895
163	H	9.614785	2.53305	0.566991	187	H	5.339692	4.857063	-0.56308
164	H	8.244179	1.536586	-1.30738	188	H	7.05698	6.994603	-1.60145
165	H	9.015954	2.62818	-2.44073	189	H	7.174839	6.246264	-0.01129
166	H	7.543891	3.644992	0.046324					

Table S11. Cartesian coordinates of the complex $[Cu(\mathbf{L4})_2]^+$ at its optimized S_0 geometry.

atom		x	y	z	atom		x	y	z
1	C	-2.09201	-5.1547	-1.64635	20	C	3.218081	-0.20063	2.797123
2	C	-2.96596	-4.5383	-2.48925	21	C	4.189039	0.640079	3.349748
3	C	-2.99959	-3.1073	-2.59843	22	C	5.298495	1.065712	2.611893
4	C	-2.11871	-2.31609	-1.81545	23	C	5.413831	0.616875	1.28785
5	C	-1.1983	-2.97242	-0.90791	24	C	4.455574	-0.21586	0.716292
6	C	-1.18663	-4.38978	-0.83673	25	C	6.330671	2.001834	3.209124
7	N	-0.37904	-2.19997	-0.14746	26	C	-3.47824	7.358671	-0.78593
8	C	0.46949	-2.78709	0.701794	27	N	6.686365	1.625952	4.58069
9	C	0.557138	-4.18885	0.816815	28	N	-2.13905	7.945328	-0.92069
10	C	-0.26715	-4.98791	0.050638	29	C	2.135974	4.389654	-0.18767
11	C	-3.87649	-2.43035	-3.47171	30	C	1.393731	4.532328	0.945659
12	C	-3.83376	-1.05267	-3.54426	31	C	0.443978	3.529189	1.334574
13	C	-2.91702	-0.34159	-2.74435	32	C	0.264592	2.373344	0.529799
14	N	-2.09018	-0.96045	-1.89675	33	C	1.048411	2.228661	-0.67963
15	C	1.318686	-1.89591	1.575709	34	C	1.983055	3.236005	-1.02712
16	O	2.456845	-1.45859	0.829049	35	N	0.858712	1.120293	-1.44315
17	C	-2.82896	1.163149	-2.84353	36	C	1.566958	0.969034	-2.56214
18	O	-3.46552	1.725944	-1.69696	37	C	2.522953	1.918224	-2.97838
19	C	3.349007	-0.62989	1.470342	38	C	2.730331	3.045325	-2.21109

39	C	-0.3353	3.631469	2.506065	87	H	-0.22208	-6.07006	0.128802
40	C	-1.2158	2.618555	2.827704	88	H	-4.57122	-2.99808	-4.08349
41	C	-1.32605	1.496937	1.981756	89	H	-4.4922	-0.50994	-4.21378
42	N	-0.60864	1.384454	0.85986	90	H	1.643028	-2.45058	2.463837
43	O	2.250531	-0.56503	-4.35746	91	H	0.728729	-1.02997	1.896759
44	C	-2.25645	0.362188	2.341602	92	H	-3.32309	1.502523	-3.76137
45	O	-3.37599	0.402874	1.458934	93	H	-1.77711	1.472157	-2.87558
46	C	3.29432	-1.37484	-3.96946	94	H	2.378386	-0.50847	3.409267
47	C	-4.33038	-0.57688	1.582482	95	H	4.084226	0.962442	4.381158
48	C	3.489376	-1.86638	-2.67336	96	H	6.270258	0.92226	0.691006
49	C	4.583178	-2.69804	-2.41338	97	H	4.556715	-0.55963	-0.30801
50	C	5.496114	-3.04946	-3.41406	98	H	5.925943	3.022827	3.23542
51	C	5.279982	-2.54416	-4.70395	99	H	7.21136	2.042956	2.540704
52	C	4.196802	-1.71503	-4.98457	100	H	-4.13686	7.853871	-1.51151
53	C	-5.36077	-0.54499	0.63254	101	H	-3.92148	7.547308	0.209836
54	C	-6.38503	-1.4863	0.689462	102	H	7.011021	0.660651	4.56665
55	C	-6.40875	-2.48293	1.676753	103	H	2.856422	5.148566	-0.47778
56	C	-5.37044	-2.49934	2.614277	104	H	1.509892	5.406828	1.578896
57	C	-4.33565	-1.55978	2.57999	105	H	3.073705	1.74943	-3.89589
58	C	6.694022	-3.9265	-3.10865	106	H	3.45872	3.793531	-2.51041
59	C	-7.50998	-3.52393	1.710235	107	H	-0.23191	4.502669	3.146084
60	N	6.334647	-5.07651	-2.27505	108	H	-1.82208	2.672188	3.725373
61	N	-8.02405	-3.7307	3.068895	109	H	-2.5842	0.468505	3.382265
62	C	-3.42743	3.090049	-1.54615	110	H	-1.73284	-0.59657	2.236761
63	C	-2.92923	3.980638	-2.50622	111	H	2.818252	-1.61427	-1.86016
64	C	-2.95028	5.353651	-2.24639	112	H	4.72394	-3.08746	-1.40952
65	C	-3.46017	5.867152	-1.04851	113	H	5.96739	-2.80573	-5.50513
66	C	-3.94826	4.956332	-0.10013	114	H	4.028224	-1.33235	-5.98668
67	C	-3.93746	3.584234	-0.33809	115	H	-5.33799	0.219523	-0.13775
68	C	-2.60743	10.29035	-1.68766	116	H	-7.18198	-1.44556	-0.04968
69	C	-8.64268	-6.15144	3.101741	117	H	-5.37636	-3.25385	3.395127
70	C	7.477915	-5.94202	-1.97496	118	H	-3.55459	-1.60429	3.330566
71	C	-0.39738	10.67406	-2.96201	119	H	7.447008	-3.34829	-2.55252
72	C	-1.85228	10.19269	-3.02311	120	H	7.179297	-4.21951	-4.05834
73	C	7.056773	-7.15236	-1.13883	121	H	-7.11671	-4.48479	1.351743
74	C	8.230712	-8.06859	-0.77035	122	H	-8.30401	-3.23565	0.996059
75	C	-10.7911	-7.26881	2.193194	123	H	5.63179	-5.6243	-2.77211
76	C	-9.75545	-7.19025	3.322942	124	H	-2.53207	3.625681	-3.45049
77	C	1.234948	-0.23868	-3.42172	125	H	-2.55571	6.037939	-2.99228
78	Cu	-0.62361	-0.14672	-0.58595	126	H	-4.34113	5.326118	0.84427
79	C	-2.08031	9.371919	-0.57339	127	H	-4.29767	2.879594	0.404543
80	C	-9.13027	-4.69696	3.143401	128	H	-3.67108	10.07842	-1.8609
81	C	6.136182	3.76939	6.741006	129	H	-2.56415	11.32419	-1.31576
82	C	7.227045	3.829722	5.659432	130	H	-8.14966	-6.35688	2.141237
83	C	7.738454	2.464867	5.170582	131	H	-7.8746	-6.26881	3.877353
84	H	-2.06529	-6.23751	-1.56946	132	H	8.211309	-5.34478	-1.41503
85	H	-3.65081	-5.11942	-3.09954	133	H	7.995035	-6.28798	-2.89119
86	H	1.2647	-4.62498	1.513603	134	H	-0.33759	11.71082	-2.60651

135	H	0.208531	10.0569	-2.28859	153	H	8.572227	2.635045	4.463311
136	H	-2.39292	10.78631	-3.77259	154	H	-8.35883	-2.83161	3.412064
137	H	-1.87833	9.152857	-3.37099	155	H	-9.64347	-4.52808	4.098593
138	H	6.305716	-7.73263	-1.69614	156	H	-9.87686	-4.5242	2.347023
139	H	6.559692	-6.79669	-0.226	157	H	-1.51234	7.434989	-0.3008
140	H	8.981166	-7.48951	-0.21386	158	H	-2.63844	9.591219	0.3566
141	H	8.727605	-8.4106	-1.6891	159	H	-1.03045	9.61183	-0.36992
142	H	-11.5186	-8.06652	2.383596	160	H	-10.3074	-7.48161	1.231316
143	H	-11.3505	-6.33304	2.082983	161	C	7.804639	-9.28331	0.061225
144	H	-10.2656	-6.97688	4.273401	162	H	0.07143	10.63435	-3.95231
145	H	-9.28884	-8.17682	3.444059	163	H	5.285935	3.19159	6.358466
146	H	0.356429	-0.00172	-4.03211	164	H	7.334799	-8.97301	1.002805
147	H	0.973051	-1.09341	-2.78957	165	H	8.663335	-9.91767	0.309593
148	C	6.60177	3.170808	8.074081	166	H	7.079468	-9.90074	-0.4834
149	H	5.766206	4.78839	6.917756	167	H	7.460951	3.722106	8.477433
150	H	8.091959	4.390793	6.041656	168	H	6.900909	2.121688	7.967845
151	H	6.851182	4.405877	4.803231	169	H	5.801343	3.20833	8.822375
152	H	8.157477	1.908726	6.016767					

Table S12. Cartesian coordinates of the complex $[Cu(\mathbf{L5})_2]^+$ at its optimized S_0 geometry.

atom		x	y	z	atom		x	y	z
1	C	-3.6853	-4.27052	-1.30101	26	C	-1.01853	8.067822	-1.32666
2	C	-4.32186	-3.46592	-2.19642	27	N	6.935225	-0.49242	4.556814
3	C	-3.89459	-2.11131	-2.40388	28	N	0.445066	8.18196	-1.3812
4	C	-2.80149	-1.59166	-1.66231	29	C	3.429992	3.430231	-0.251
5	C	-2.13231	-2.445	-0.70049	30	C	2.769446	3.859127	0.860429
6	C	-2.57456	-3.78303	-0.53369	31	C	1.53191	3.252705	1.261004
7	N	-1.09885	-1.92691	0.013347	32	C	0.977675	2.197319	0.489743
8	C	-0.47203	-2.69693	0.907207	33	C	1.676983	1.749975	-0.69719
9	C	-0.83781	-4.04149	1.119247	34	C	2.902199	2.365859	-1.0558
10	C	-1.88513	-4.58318	0.402283	35	N	1.128818	0.744346	-1.42928
11	C	-4.51248	-1.25269	-3.33697	36	C	1.753332	0.321722	-2.52831
12	C	-4.02904	0.029195	-3.50443	37	C	2.98032	0.871815	-2.9523
13	C	-2.92848	0.463639	-2.73893	38	C	3.552242	1.887856	-2.21579
14	N	-2.34058	-0.32518	-1.83477	39	C	0.824017	3.655578	2.412882
15	C	0.641621	-2.0744	1.713798	40	C	-0.35032	3.013054	2.748752
16	O	1.855849	-2.16443	0.963461	41	C	-0.82768	1.964806	1.936853
17	C	-2.362	1.849642	-2.94022	42	N	-0.18234	1.575858	0.832915
18	O	-2.76593	2.6611	-1.83839	43	O	1.882589	-1.40765	-4.2707
19	C	2.999049	-1.67436	1.551855	44	C	-2.08838	1.223269	2.316077
20	C	3.047511	-1.08544	2.821451	45	O	-3.12071	1.595053	1.404815
21	C	4.269467	-0.62635	3.323669	46	C	2.559192	-2.53196	-3.85389
22	C	5.454776	-0.74283	2.590403	47	C	-4.34434	0.985042	1.535865
23	C	5.383822	-1.34165	1.323595	48	C	2.568493	-3.02217	-2.54239
24	C	4.177136	-1.79954	0.802672	49	C	3.279824	-4.19071	-2.25372
25	C	6.772577	-0.21861	3.126435	50	C	3.994444	-4.88171	-3.23916

51	C	3.973982	-4.36844	-4.54339	99	H	7.599597	-0.63467	2.52038
52	C	3.268799	-3.208	-4.85366	100	H	-1.43383	8.675694	-2.14071
53	C	-5.28683	1.292031	0.544874	101	H	-1.43646	8.480033	-0.38914
54	C	-6.56202	0.736295	0.60595	102	H	6.836732	-1.49644	4.696939
55	C	-6.93	-0.13971	1.638237	103	H	4.368185	3.888206	-0.54981
56	C	-5.97447	-0.43503	2.616755	104	H	3.169337	4.666102	1.467006
57	C	-4.69055	0.116806	2.578798	105	H	3.449176	0.491817	-3.85199
58	C	4.777687	-6.13403	-2.90137	106	H	4.495504	2.331282	-2.52168
59	C	-8.31026	-0.76456	1.678321	107	H	1.21172	4.462705	3.02743
60	N	3.977524	-7.08562	-2.12459	108	H	-0.90904	3.301247	3.632539
61	N	-8.90624	-0.68486	3.017034	109	H	-2.37296	1.47901	3.343248
62	C	-2.30124	3.951818	-1.78289	110	H	-1.91471	0.14095	2.261554
63	C	-1.5643	4.574485	-2.79891	111	H	2.045273	-2.5152	-1.73943
64	C	-1.15677	5.90139	-2.6368	112	H	3.271164	-4.57617	-1.23835
65	C	-1.46847	6.630661	-1.48358	113	H	4.51405	-4.88644	-5.33253
66	C	-2.20054	5.983937	-0.4772	114	H	3.247168	-2.81894	-5.86717
67	C	-2.61637	4.662188	-0.61692	115	H	-4.99924	1.959813	-0.26104
68	C	0.795612	10.48382	-2.32466	116	H	-7.28558	0.987659	-0.16629
69	C	-10.2905	-2.76007	3.17049	117	H	-6.24116	-1.10054	3.432327
70	C	4.710848	-8.31353	-1.80154	118	H	-3.98284	-0.1333	3.36113
71	C	3.066656	9.95548	-3.49097	119	H	5.65307	-5.8736	-2.28823
72	C	1.533029	10.03231	-3.5972	120	H	5.179719	-6.57227	-3.83373
73	C	3.820242	-9.2992	-1.03923	121	H	-8.24038	-1.82638	1.406356
74	C	4.542931	-10.5665	-0.55336	122	H	-8.94445	-0.29371	0.903899
75	C	-12.6603	-3.20095	2.214344	123	H	3.154641	-7.33303	-2.67515
76	C	-11.6897	-3.35816	3.394977	124	H	-1.30977	4.048935	-3.71231
77	C	1.034262	-0.72595	-3.36029	125	H	-0.58226	6.375879	-3.42772
78	Cu	-0.695	0.075062	-0.55434	126	H	-2.44914	6.52312	0.434074
79	C	0.940542	9.540502	-1.11972	127	H	-3.1685	4.157558	0.16944
80	C	-10.2712	-1.22739	3.090583	128	H	-0.2692	10.60964	-2.56248
81	C	7.352074	1.910357	6.472765	129	H	1.146168	11.47682	-2.01342
82	C	8.321797	1.435807	5.382177	130	H	-9.86079	-3.19099	2.255381
83	C	8.237257	-0.07086	5.092917	131	H	-9.62982	-3.0598	3.994498
84	H	-4.00682	-5.2967	-1.15056	132	H	5.569123	-8.03498	-1.17337
85	H	-5.16195	-3.83683	-2.77588	133	H	5.129816	-8.7946	-2.70455
86	H	-0.29751	-4.63303	1.850451	134	C	3.752449	11.2991	-3.21331
87	H	-2.18908	-5.6146	0.554433	135	H	3.35094	9.232468	-2.71445
88	H	-5.35627	-1.60977	-3.91991	136	H	1.272168	10.72163	-4.41323
89	H	-4.48145	0.705211	-4.22182	137	H	1.151022	9.046169	-3.88823
90	H	0.749794	-2.60584	2.66654	138	H	2.97196	-9.58837	-1.6782
91	H	0.404607	-1.02459	1.918757	139	H	3.392283	-8.77138	-0.17704
92	H	-2.73535	2.268433	-3.88199	140	H	5.403112	-10.2781	0.06927
93	H	-1.26702	1.801062	-2.98722	141	C	5.01816	-11.5111	-1.66773
94	H	2.155401	-0.97915	3.42778	142	H	-12.1375	-2.90919	4.29455
95	H	4.301228	-0.17819	4.312103	143	H	-11.5792	-4.4295	3.614741
96	H	6.290028	-1.45553	0.732955	144	H	0.29294	-0.22046	-3.98953
97	H	4.134199	-2.26382	-0.17723	145	H	0.491951	-1.42043	-2.71062
98	H	6.817596	0.870207	2.985931	146	H	7.590816	1.393733	7.415271

147	C	7.35659	3.428883	6.714283	165	H	5.982368	-13.4544	-1.93715
148	H	9.357526	1.656743	5.671564	166	H	4.944766	-13.3568	-0.50428
149	H	8.139872	2.003669	4.458324	167	H	3.464435	11.71265	-2.23988
150	H	8.394202	-0.61926	6.030162	168	H	3.489801	12.04078	-3.97859
151	H	9.066368	-0.35309	4.416856	169	H	4.843566	11.19192	-3.21404
152	H	-8.93631	0.298266	3.282931	170	C	8.67465	3.980752	7.272222
153	H	-10.731	-0.82449	4.0019	171	H	6.545057	3.675256	7.41269
154	H	-10.8923	-0.88406	2.243332	172	H	7.114657	3.944456	5.773397
155	H	0.831426	7.555981	-0.67649	173	H	8.940446	3.485247	8.214866
156	H	0.43446	10.00168	-0.25024	174	H	8.598785	5.056193	7.471499
157	H	1.99968	9.451432	-0.85174	175	H	9.507055	3.833584	6.574598
158	H	3.862954	-11.1214	0.108346	176	C	-14.0092	-3.88629	2.45987
159	H	3.454717	9.547234	-4.4342	177	H	-12.1957	-3.61972	1.309876
160	H	6.338987	1.593128	6.197581	178	H	-12.8298	-2.13649	2.005857
161	H	5.740855	-10.9962	-2.31412	179	H	-13.8802	-4.96203	2.633705
162	H	4.162898	-11.7687	-2.30914	180	H	-14.6823	-3.76448	1.603244
163	C	5.654643	-12.7954	-1.12476	181	H	-14.5112	-3.46695	3.340831
164	H	6.530767	-12.5705	-0.50363					

Table S13. Cartesian coordinates of the complex [Cu(**L6**)$_2$]$^+$ at its optimized S_0 geometry.

atom		x	y	z	atom		x	y	z
1	C	5.409059	1.334656	-1.7126	26	C	-3.74743	-7.33953	-0.9283
2	C	5.442611	0.256254	-2.5432	27	N	-5.26989	4.733538	4.273201
3	C	4.312271	-0.62089	-2.6581	28	N	-5.0371	-6.69097	-1.1906
4	C	3.142182	-0.37304	-1.8935	29	C	-4.80574	-0.82758	-0.3103
5	C	3.112134	0.76776	-0.9997	30	C	-4.49427	-1.4978	0.834001
6	C	4.244972	1.619109	-0.9226	31	C	-3.12728	-1.68416	1.229001
7	N	1.993043	0.97861	-0.2584	32	C	-2.07935	-1.17401	0.418001
8	C	1.95019	2.020412	0.577301	33	C	-2.41302	-0.47229	-0.8046
9	C	3.025131	2.923864	0.697401	34	C	-3.77501	-0.29753	-1.1562
10	C	4.168422	2.724912	-0.0494	35	N	-1.3982	0.001163	-1.5753
11	C	4.299821	-1.73739	-3.5201	36	C	-1.68347	0.648776	-2.7048
12	C	3.166785	-2.52134	-3.5996	37	C	-3.01026	0.875736	-3.1244
13	C	2.0398	-2.19789	-2.8174	38	C	-4.05038	0.405483	-2.3507
14	N	2.035747	-1.15679	-1.9801	39	C	-2.76171	-2.35748	2.413401
15	C	0.713398	2.191368	1.426001	40	C	-1.42691	-2.47864	2.742701
16	O	-0.28157	2.877713	0.661901	41	C	-0.44689	-1.93008	1.891501
17	C	0.780162	-3.02534	-2.9218	42	N	-0.76846	-1.30387	0.755401
18	O	0.704724	-3.87043	-1.7743	43	O	-0.82161	2.075237	-4.5124
19	C	-1.48166	3.142267	1.281201	44	C	1.015108	-2.01155	2.265201
20	C	-1.78358	2.795181	2.604501	45	O	1.653166	-2.93627	1.387401
21	C	-3.03666	3.117737	3.133501	46	C	-0.72405	3.395733	-4.1351
22	C	-4.00563	3.782781	2.374401	47	C	3.005658	-3.13064	1.525001
23	C	-3.67932	4.126366	1.054401	48	C	-0.44143	3.83862	-2.8376
24	C	-2.43843	3.81161	0.506501	49	C	-0.35057	5.211116	-2.5868
25	C	-5.36932	4.106643	2.951001	50	C	-0.54192	6.159525	-3.5982

51	C	-0.82704	5.691438	-4.8886	99	H	-5.92879	4.731068	2.229301
52	C	-0.92081	4.328642	-5.1604	100	H	-3.66677	-8.20713	-1.5968
53	C	3.59842	-3.97006	0.571501	101	H	-3.67935	-7.74063	0.100301
54	C	4.962107	-4.24172	0.640301	102	H	-4.69175	5.566912	4.181601
55	C	5.769633	-3.68506	1.643601	103	H	-5.84133	-0.68424	-0.6041
56	C	5.15957	-2.84873	2.584901	104	H	-5.27579	-1.89996	1.471701
57	C	3.790783	-2.56707	2.538401	105	H	-3.18994	1.409844	-4.0498
58	C	-0.46616	7.644221	-3.303	106	H	-5.08158	0.56393	-2.6531
59	C	7.25902	-3.96213	1.689901	107	H	-3.53283	-2.76894	3.058001
60	N	0.70136	7.981769	-2.4842	108	H	-1.12204	-2.98635	3.651301
61	N	7.714006	-4.27415	3.049501	109	H	1.112393	-2.34055	3.306301
62	C	-0.40071	-4.67358	-1.6388	110	H	1.479853	-1.02197	2.167701
63	C	-1.39782	-4.82234	-2.6114	111	H	-0.29886	3.145614	-2.0163
64	C	-2.47386	-5.68069	-2.3678	112	H	-0.11795	5.549406	-1.5814
65	C	-2.58399	-6.39958	-1.1723	113	H	-0.97391	6.403344	-5.6976
66	C	-1.57758	-6.22913	-0.2102	114	H	-1.13252	3.970051	-6.1632
67	C	-0.49564	-5.38138	-0.433	115	H	2.976101	-4.39273	-0.211
68	C	-6.51409	-8.53361	-2.0418	116	H	5.407878	-4.90004	-0.1021
69	C	10.04545	-3.37515	3.100401	117	H	5.76289	-2.41806	3.378501
70	C	0.801524	9.416764	-2.2004	118	H	3.357112	-1.91945	3.292001
71	C	-8.37323	-7.26442	-3.3667	119	H	-1.35694	7.958362	-2.7388
72	C	-6.94556	-7.85949	-3.3581	120	H	-0.49853	8.204823	-4.2558
73	C	2.061838	9.727507	-1.3873	121	H	7.80826	-3.07415	1.348901
74	C	2.190305	11.1939	-0.9401	122	H	7.501184	-4.76454	0.967801
75	C	12.2179	-4.47905	2.225801	123	H	1.539246	7.689831	-2.9884
76	C	11.53363	-3.67912	3.346501	124	H	-1.3489	-4.28824	-3.5536
77	C	-0.50685	1.058323	-3.5737	125	H	-3.24676	-5.78655	-3.1235
78	Cu	0.493079	-0.44762	-0.6976	126	H	-1.64231	-6.76653	0.733201
79	C	-6.19774	-7.54642	-0.9055	127	H	0.272862	-5.23761	0.319701
80	C	9.141991	-4.61541	3.132301	128	H	-5.63052	-9.15855	-2.2294
81	C	-6.60475	3.387899	6.733101	129	H	-7.29482	-9.21687	-1.6858
82	C	-7.31882	3.954131	5.498001	130	H	9.94117	-2.85715	2.136801
83	C	-6.56987	5.122497	4.838601	131	H	9.683978	-2.67914	3.868601
84	H	6.263789	1.999417	-1.6316	132	H	-0.08716	9.705804	-1.6211
85	H	6.324601	0.042615	-3.1394	133	H	0.780652	10.02287	-3.1247
86	H	2.945669	3.760667	1.382901	134	C	-9.46897	-8.18767	-3.9311
87	H	5.011953	3.403974	0.033201	135	H	-8.65522	-6.95071	-2.3509
88	H	5.17731	-1.96553	-4.1177	136	H	-6.85699	-8.58249	-4.1812
89	H	3.129546	-3.37994	-4.2612	137	H	-6.21902	-7.06652	-3.5713
90	H	0.960524	2.768357	2.324701	138	H	2.949426	9.449167	-1.9758
91	H	0.337654	1.207685	1.729301	139	H	2.061109	9.077007	-0.5029
92	H	0.808335	-3.62604	-3.8383	140	H	1.287918	11.48244	-0.3801
93	H	-0.09641	-2.3671	-2.9594	141	C	2.41765	12.19639	-2.0828
94	H	-1.0637	2.281648	3.231001	142	H	11.63601	-4.20963	4.303501
95	H	-3.25897	2.853147	4.163001	143	H	12.06698	-2.72545	3.468701
96	H	-4.40819	4.651999	0.441701	144	H	-0.20629	0.19761	-4.1815
97	H	-2.19502	4.084799	-0.5151	145	H	0.348759	1.332785	-2.9483
98	H	-5.95036	3.181069	3.062501	146	H	-6.53801	4.173996	7.501001

147	C	-7.2672	2.141229	7.341901	171	H	1.681529	13.94722	-1.0437
148	H	-8.32071	4.312776	5.767601	172	H	2.575945	14.30348	-2.5121
149	H	-7.47346	3.150338	4.763601	173	H	3.840603	13.38123	0.143401
150	H	-6.37814	5.891788	5.597101	174	H	3.908277	15.00462	-0.5505
151	H	-7.22345	5.585027	4.075501	175	H	4.735816	13.66279	-1.3575
152	H	7.17357	-5.07222	3.379801	176	C	14.14805	-5.49204	3.611601
153	H	9.295567	-5.13492	4.086701	177	H	14.19903	-3.64654	2.482301
154	H	9.439459	-5.32093	2.335201	178	H	14.14977	-5.09684	1.492201
155	H	-5.09297	-5.85887	-0.6063	179	H	13.83077	-5.03832	4.557401
156	H	-6.06507	-8.11453	0.034301	180	H	15.23655	-5.61499	3.654501
157	H	-7.05791	-6.88458	-0.7495	181	H	13.70151	-6.49322	3.557501
158	H	3.023108	11.25986	-0.2284	182	C	-9.24805	1.124018	8.599801
159	H	-8.37279	-6.34652	-3.9696	183	H	-9.34848	2.732223	7.163301
160	H	-5.57236	3.145952	6.452801	184	H	-8.59846	3.186089	8.686001
161	H	1.579447	12.14133	-2.7897	185	H	-9.35019	0.307823	7.873401
162	H	3.314637	11.90065	-2.6483	186	H	-10.2404	1.319763	9.022501
163	C	2.569716	13.65748	-1.6238	187	H	-8.60377	0.764389	9.412001
164	C	-8.66069	2.378091	7.942501	188	C	-9.71193	-9.48316	-3.1474
165	H	-6.61062	1.741899	8.128701	189	H	-9.21638	-8.43928	-4.9714
166	H	-7.33414	1.354532	6.574901	190	H	-10.409	-7.62043	-3.9766
167	C	13.73739	-4.64152	2.402801	191	H	-8.83326	-10.1379	-3.1604
168	H	12.02632	-3.97314	1.268301	192	H	-10.5478	-10.0481	-3.5772
169	H	11.76195	-5.47543	2.141401	193	H	-9.95622	-9.27335	-2.0984
170	C	3.832229	13.93973	-0.7994					

The singlet excited geometry and absorption spectra were computed using TD-DFT calculations.[10-12] The influence of solvation on vertical excitation energies and oscillator strengths (f) was considered by employing CPCM at the same level of theory as the optimization step. The first twenty singlet excited states and their transition oscillator strengths (*f*) were calculated, and those with *f* > 0.01 were used to generate simulated UV-vis spectra using Multiwfn.[13] Natural transition orbitals (NTOs)[14] associated with the calculated TD-DFT spectra were visualized using the Avogadro software.[15]

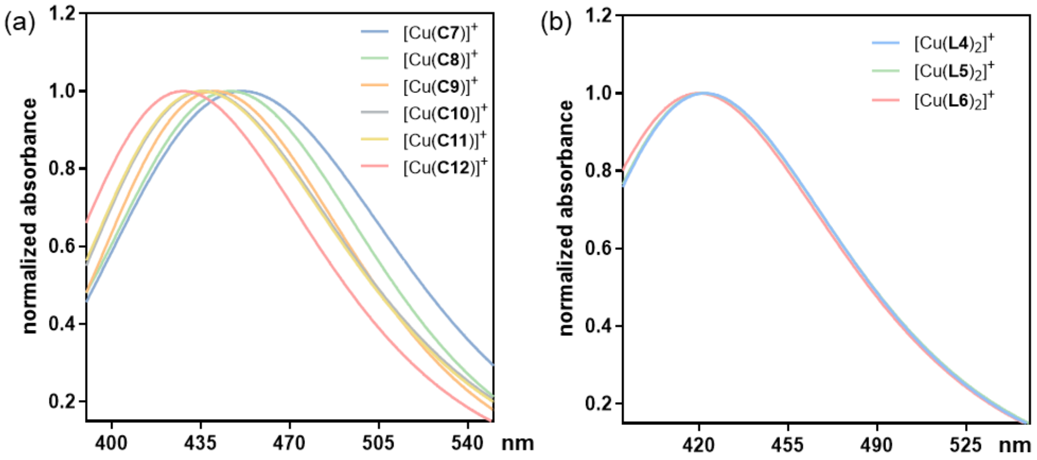

Figure S32. TDDFT simulated steady-state UV-Vis absorption spectra.

Table S14. Major $S_0 \rightarrow S_n$ transitions of [Cu(**Cn**)]$^+$ (n = 7–12) and [Cu(**Ln**)$_2$]$^+$ (n = 4–6) at the optimized S_0 geometry in acetonitrile solution.

	n	λ (nm)	*f*	major contributions
[Cu(**C7**)]$^+$	1	490.83	0.0125	HOMO → LUMO (54%), H-1 → LUMO (34%)
	2	451.20	0.0631	H-1 → LUMO (49%), HOMO → LUMO (35%)
	3	419.43	0.0171	H-1 → L+2 (49%), HOMO → L+2 (24%)
	4	397.57	0.0114	H-1 → L+4 (64%)
	5	322.02	0.0107	H-11 → LUMO (63%)
[Cu(**C8**)]$^+$	1	472.52	0.0184	HOMO → LUMO (45%), HOMO → L+1 (43%)
	2	446.52	0.1038	H-3 → LUMO (46%), H-3 → L+1 (40%)
	3	373.1	0.0103	H-6 → L+1 (45%), H-4 → L+3 (29%)
	4	329.15	0.0238	H-11 → L+3 (48%), H-11 → L+4 (39%)
[Cu(**C9**)]$^+$	1	484.32	0.0141	HOMO → LUMO (58%)
	2	440.06	0.1069	H-5 → LUMO (49%), H-1 → L+1 (33%)
	3	413.06	0.0108	H-1 → L+2 (61%)
	4	330.36	0.0172	H-11 → L+3 (39%), H-8 → L+3 (32%)
[Cu(**C10**)]$^+$	1	489.46	0.0237	HOMO → LUMO (65%)
	2	436.19	0.1010	H-5 → LUMO (53%), H-4 → L+1 (25%)
	3	331.97	0.0173	H-11 → L+3 (39%), H-9 → L+3 (39%)
[Cu(**C11**)]$^+$	1	488.59	0.0230	HOMO → LUMO (65%)
	2	435.53	0.1003	H-5 → LUMO (42%), H-3 → L+1 (41%)
	3	331.77	0.0222	H-11 → L+2 (62%)
[Cu(**C12**)]$^+$	1	483.19	0.0193	HOMO → LUMO (59%)
	2	428.11	0.1015	H-5 → LUMO (48%), H-4 → L+1 (38%)
	3	401.78	0.0101	H-4 → L+2 (58%)
	4	329.43	0.0241	H-11 → L+2 (54%), H-11 → L+3 (30%)
[Cu(**L4**)$_2$]$^+$	1	482.99	0.0219	HOMO → LUMO (58%)
	2	423.41	0.094	H-5 → LUMO (43%), H-5 → L+1 (40%)
	3	398.86	0.0118	H-5 → L+2 (62%)
	4	384.95	0.0136	H-5 → L+3 (61%)
	5	328.21	0.0165	H-11 → L+2 (60%)
[Cu(**L5**)$_2$]$^+$	1	483.35	0.0228	HOMO → LUMO (58%)
	2	422.84	0.0936	H-5 → LUMO (43%), H-5 → L+1 (41%)
	3	397.96	0.0117	H-5 → L+2 (61%)
	4	384.3	0.0133	H-5 → L+3 (62%)
	5	327.95	0.019	H-11 → L+2 (62%)
[Cu(**L6**)$_2$]$^+$	1	422.47	0.1149	H-5 → LUMO (35%), H-5 → L+1 (28%)
	2	394.18	0.0231	H-2 → L+2 (45%), HOMO → L+3 (32%)
	3	373.69	0.0117	H-3 → L+1 (52%)
	4	320.17	0.0201	H-11 → L+2 (58%)

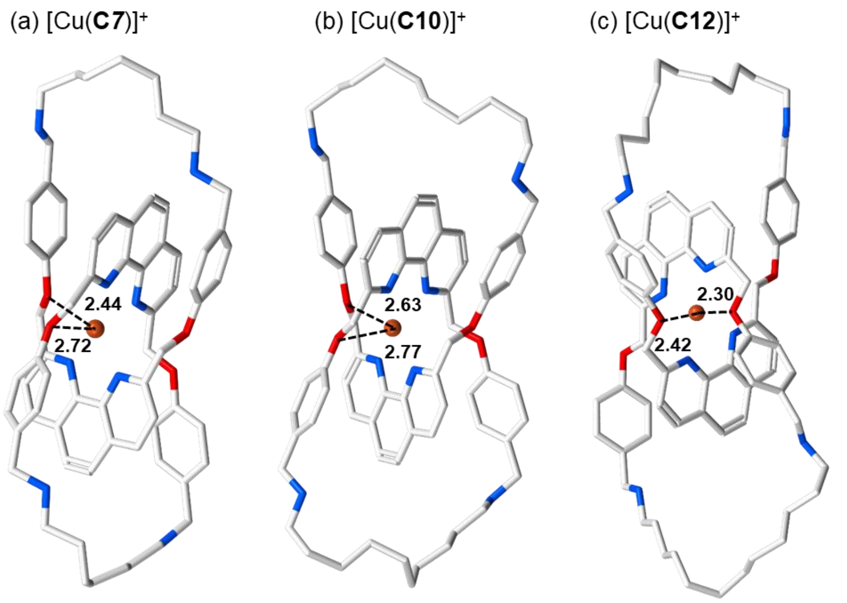

Figure S33. Geometrically optimized T_1 state structure of (a) $[Cu(\mathbf{C7})]^+$; (b) $[Cu(\mathbf{C10})]^+$; (c) $[Cu(\mathbf{C12})]^+$ showing a close contact between the Cu center and the ether O (Å). Protons are omitted for clarity. Cu: orange, O: red, N: blue, C: grey.

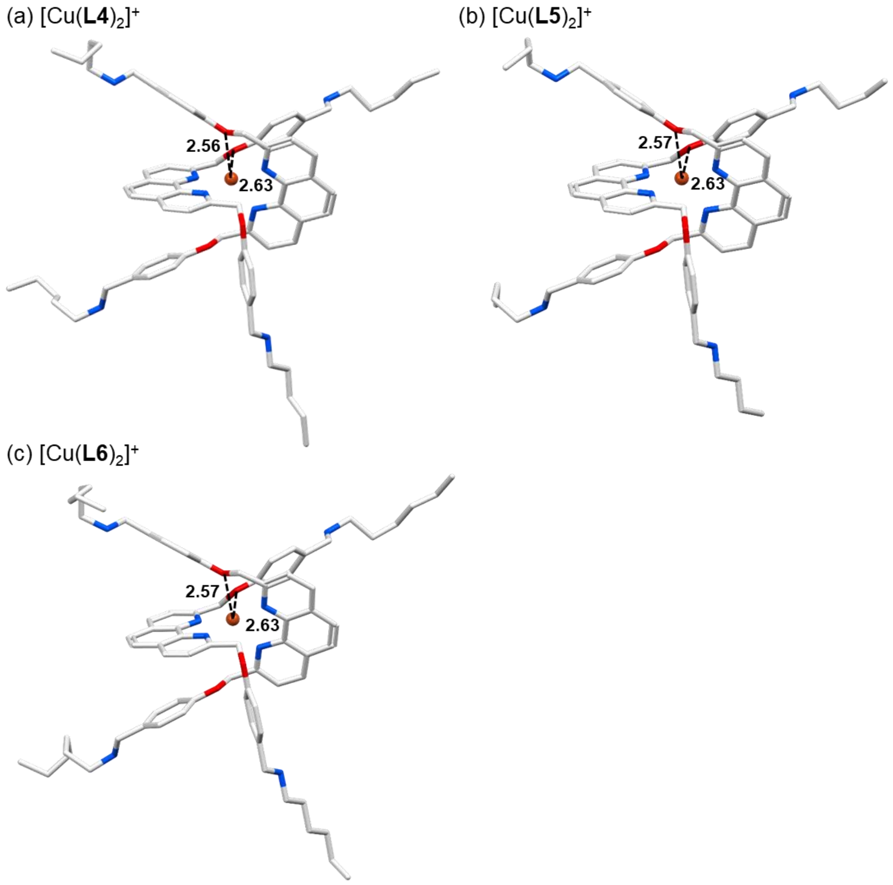

Figure S34. Geometrically optimized T_1 state structure of (a) $[Cu(\mathbf{L4})_2]^+$; (b) $[Cu(\mathbf{L5})_2)]^+$; (c) $[Cu(\mathbf{L6})_2]^+$ showing a close contact between the Cu center and the ether O (Å). Protons are omitted for clarity. Cu: orange, O: red, N: blue, C: grey.

Table S15. Structural parameters of selected, DFT-simulated catenane and non-interlocked complexes in the T_1 state.

	α (°)	β (°)	d / Å	$d_{Cu\text{-}N}$ / Å			
[Cu(**C7**)]$^+$	76.4	153.1	6.06	1.931	2.121	2.018	2.262
[Cu(**C10**)]$^+$	70.1	162.8	6.14	1.941	2.091	2.027	2.225
[Cu(**C12**)]$^+$	85.4	141.0	5.97	1.935	2.162	2.033	2.333
[Cu(**L4**)$_2$]$^+$	73.5	153.5	6.08	1.935	2.129	2.020	2.251
[Cu(**L5**)$_2$]$^+$	73.3	153.8	6.09	1.936	2.129	2.020	2.251
[Cu(**L6**)$_2$]$^+$	73.4	153.4	6.09	1.936	2.219	2.020	2.255

Table S16. Cartesian coordinates of optimized [Cu(**C7**)]$^+$ at T_1 state.

atom		x	y	z	atom		x	y	z
1	C	-5.25	-0.42893	0.378077	47	C	-0.98479	-3.47969	-0.56126
2	C	-5.10544	-0.78922	1.68422	48	C	-3.63892	3.994389	-0.04114
3	C	-3.80382	-0.88001	2.29384	49	C	-5.01184	4.228341	-0.05734
4	C	-2.67694	-0.57437	1.47289	50	C	-5.80811	4.002172	1.076706
5	C	-2.811	-0.22683	0.121834	51	C	-5.1692	3.534468	2.231655
6	C	-4.11527	-0.12658	-0.4581	52	C	-3.79316	3.289117	2.270084
7	N	-1.65239	-0.01076	-0.617	53	C	-1.79242	-3.71893	0.554889
8	C	-1.78644	0.366807	-1.91115	54	C	-3.09619	-4.17525	0.379788
9	C	-3.03286	0.51723	-2.51601	55	C	-3.61663	-4.42557	-0.89769
10	C	-4.21034	0.257591	-1.79375	56	C	-2.75932	-4.27082	-1.99331
11	C	-3.57474	-1.26799	3.614702	57	C	-1.44819	-3.80992	-1.8397
12	C	-2.24799	-1.35542	4.101256	58	C	-7.31373	4.239812	1.066254
13	C	-1.19255	-1.01992	3.270581	59	C	-5.08451	-4.74335	-1.09424
14	N	-1.39564	-0.62781	1.988684	60	N	-8.17145	3.043163	1.001598
15	C	-0.54641	0.573097	-2.74505	61	N	-5.70497	-3.71875	-1.95153
16	O	0.091414	1.808455	-2.40003	62	C	2.452741	-1.36566	2.830751
17	C	0.23585	-0.99721	3.747124	63	C	3.143839	-0.30568	3.427877
18	O	1.075107	-1.34201	2.624987	64	C	4.537358	-0.33115	3.453617
19	C	1.359409	2.011513	-2.88796	65	C	5.263513	-1.39751	2.901993
20	C	1.982543	1.199347	-3.84516	66	C	4.547575	-2.47133	2.3589
21	C	3.319476	1.433708	-4.18435	67	C	3.150834	-2.45933	2.319565
22	C	4.061514	2.458967	-3.58886	68	C	10.32541	-0.87633	-1.37651
23	C	3.390766	3.312324	-2.6998	69	C	-7.16775	-3.6716	-1.85616
24	C	2.057808	3.10455	-2.35603	70	C	-8.06643	2.283708	-0.24841
25	C	5.559841	2.573228	-3.80505	71	C	7.686776	1.601163	-2.96247
26	C	6.782651	-1.34586	2.907357	72	C	8.284896	0.302578	-2.40518
27	N	6.248829	1.427638	-3.18522	73	C	9.660417	0.463816	-1.74451
28	N	7.396248	-2.34745	2.037008	74	C	-8.86976	0.980811	-0.16686
29	C	4.51689	2.498215	0.681639	75	C	-8.79379	0.137325	-1.44916

30	C	4.928349	1.418995	-0.03849	76	C	-9.37021	-1.2788	-1.28398
31	C	4.008691	0.376829	-0.3926	77	C	-9.18614	-2.19	-2.51491
32	C	2.65707	0.455409	0.018361	78	C	-7.7318	-2.60658	-2.80937
33	C	2.219084	1.599181	0.790335	79	C	-0.98578	2.803679	2.192302
34	C	3.15158	2.623222	1.098688	80	Cu	-0.00579	-0.34657	0.677554
35	N	0.924356	1.650861	1.188204	81	C	9.562237	-1.78097	-0.3887
36	C	0.481893	2.727557	1.840318	82	C	9.530899	-1.29191	1.069521
37	C	1.338231	3.799863	2.167303	83	C	8.867909	-2.30775	2.027554
38	C	2.670433	3.740029	1.814165	84	H	-6.23981	-0.36601	-0.06194
39	C	4.395198	-0.75211	-1.14486	85	H	-5.97774	-1.01819	2.290621
40	C	3.471836	-1.73465	-1.43267	86	H	-3.08112	0.820869	-3.55603
41	C	2.144693	-1.59166	-0.98968	87	H	-5.18135	0.35681	-2.26967
42	N	1.75309	-0.51679	-0.29605	88	H	-4.41167	-1.51641	4.260205
43	O	-1.66511	3.3209	1.042598	89	H	-2.05071	-1.67378	5.11928
44	C	1.12107	-2.63505	-1.37819	90	H	-0.82046	0.578818	-3.80593
45	O	0.231658	-2.87312	-0.29484	91	H	0.155219	-0.2493	-2.57873
46	C	-3.02106	3.519119	1.124285	92	H	0.395525	-1.71314	4.559449
93	H	0.494473	0.00445	4.112012	127	H	5.069772	0.511168	3.889192
94	H	1.452912	0.381406	-4.31963	128	H	5.089111	-3.31932	1.952781
95	H	3.790762	0.776148	-4.91197	129	H	2.596335	-3.27959	1.874289
96	H	3.929998	4.136494	-2.23958	130	H	11.32606	-0.67593	-0.96789
97	H	1.556406	3.753318	-1.64427	131	H	10.48497	-1.44267	-2.30514
98	H	5.925018	3.483414	-3.31593	132	H	-7.56434	-4.65931	-2.1279
99	H	5.798745	2.675293	-4.87891	133	H	-7.50331	-3.48043	-0.81951
100	H	7.089239	-0.30903	2.674476	134	H	-7.02123	2.045085	-0.51564
101	H	7.145878	-1.53776	3.92756	135	H	-8.45626	2.914864	-1.05976
102	H	6.10179	0.605891	-3.77	136	H	8.240209	1.906797	-3.87065
103	H	7.044239	-2.23104	1.087298	137	H	7.813057	2.413741	-2.23303
104	H	5.211739	3.291182	0.941339	138	H	8.349166	-0.44074	-3.21389
105	H	5.951374	1.33504	-0.38402	139	H	7.578384	-0.10333	-1.67082
106	H	0.940123	4.656214	2.700763	140	H	10.33246	1.004269	-2.42596
107	H	3.351085	4.546283	2.071255	141	H	9.559328	1.096958	-0.85186
108	H	5.419652	-0.83656	-1.48793	142	H	-9.91561	1.211421	0.074987
109	H	3.753565	-2.61843	-1.99411	143	H	-8.48452	0.386471	0.676289
110	H	0.570584	-2.26848	-2.25329	144	H	-7.74417	0.071252	-1.76647
111	H	1.625962	-3.56256	-1.66984	145	H	-9.31941	0.654866	-2.2643
112	H	-3.02978	4.172949	-0.92223	146	H	-10.4428	-1.20147	-1.05831
113	H	-5.47227	4.602044	-0.96925	147	H	-8.91227	-1.75146	-0.40348
114	H	-5.75204	3.359785	3.134082	148	H	-9.79806	-3.0939	-2.38544
115	H	-3.34346	2.929721	3.188652	149	H	-9.59194	-1.67496	-3.39658
116	H	-1.4083	-3.49375	1.544054	150	H	-7.65637	-2.98266	-3.83719
117	H	-3.73009	-4.30542	1.253186	151	H	-7.08282	-1.72118	-2.77006
118	H	-3.12656	-4.48209	-2.99365	152	H	-1.12667	3.474386	3.047951
119	H	-0.82451	-3.70113	-2.71914	153	H	-1.37028	1.812321	2.446135
120	H	-7.57668	4.880308	0.215377	154	H	10.04302	-2.76955	-0.39868
121	H	-7.5962	4.786036	1.975183	155	H	8.538757	-1.94659	-0.75341
122	H	-5.56956	-4.81294	-0.10382	156	H	9.021821	-0.32143	1.149716
123	H	-5.22213	-5.72126	-1.57785	157	H	10.56541	-1.12491	1.402247

124	H	-7.92035	2.427482	1.775734	158	H	9.237318	-3.31204	1.774493
125	H	-5.34556	-2.81093	-1.65566	159	H	9.190169	-2.11194	3.057043
126	H	2.612943	0.5486	3.834282					

Table S17. Cartesian coordinates of the complex [Cu(**C10**)]$^{+}$ at its optimized T_1 geometry.

atom		x	y	z	atom		x	y	z
1	C	-5.29824	0.054992	0.123016	10	C	-4.11151	1.098914	-1.8208
2	C	-5.23237	-0.58291	1.32615	11	C	-3.82682	-1.59675	3.143259
3	C	-3.96776	-0.92604	1.927138	12	C	-2.53181	-1.92286	3.614465
4	C	-2.78671	-0.5714	1.21037	13	C	-1.41836	-1.54143	2.888137
5	C	-2.84651	0.064978	-0.03593	14	N	-1.53076	-0.85639	1.718006
6	C	-4.11001	0.418136	-0.60553	15	C	-0.37558	1.466933	-2.4517
7	N	-1.64774	0.334526	-0.68148	16	O	0.235201	2.420756	-1.57713
8	C	-1.6845	1.050829	-1.82838	17	C	-0.01522	-1.79662	3.362396
9	C	-2.8855	1.439249	-2.41887	18	O	0.74828	-2.24965	2.228691
19	C	1.480997	2.896123	-1.89782	67	C	2.650404	-3.44884	1.504857
20	C	2.189231	2.564953	-3.0585	68	C	10.53705	-1.68669	-0.31074
21	C	3.474136	3.084826	-3.25472	69	C	-8.53883	-1.13446	-2.60993
22	C	4.076483	3.927983	-2.31575	70	C	-7.12924	3.715571	1.222891
23	C	3.338935	4.262447	-1.16841	71	C	8.438209	1.264176	-1.93082
24	C	2.057153	3.765364	-0.95924	72	C	9.167639	-0.08218	-1.76877
25	C	5.477924	4.477157	-2.51458	73	C	9.849113	-0.31023	-0.40974
26	C	6.318454	-3.4631	2.648724	74	C	-8.16207	2.586028	1.182946
27	N	6.32376	3.552525	-3.27276	75	C	-8.61233	2.235164	-0.24234
28	N	6.868508	-4.0674	1.436354	76	C	-9.54291	1.013613	-0.30236
29	C	5.066247	0.799789	0.515544	77	C	-10.131	0.730561	-1.69716
30	C	5.086943	-0.09218	-0.51429	78	C	-9.12703	0.280719	-2.7765
31	C	3.877673	-0.70601	-0.98198	79	C	0.00916	2.074626	2.850817
32	C	2.648297	-0.3978	-0.35106	80	Cu	-0.10811	-0.5296	0.438393
33	C	2.626275	0.535406	0.750691	81	C	9.582884	-2.88482	-0.11051
34	C	3.834856	1.146342	1.165298	82	C	9.287858	-3.21316	1.362919
35	N	1.432651	0.800847	1.337309	83	C	8.300359	-4.38852	1.537467
36	C	1.370704	1.717733	2.303902	84	C	-9.59221	-2.25641	-2.6892
37	C	2.525753	2.380983	2.770759	85	C	-9.07401	-3.65732	-3.06663
38	C	3.75409	2.085047	2.216883	86	C	-8.183	-4.38631	-2.04982
39	C	3.844506	-1.62362	-2.05284	87	C	9.359017	2.488834	-2.04684
40	C	2.644649	-2.19059	-2.43468	88	C	8.639391	3.84585	-2.17604
41	C	1.462652	-1.83589	-1.75908	89	C	7.721267	3.988322	-3.41535
42	N	1.474028	-0.94971	-0.75696	90	H	-6.26109	0.306072	-0.31129
43	O	-0.46054	3.186608	2.082349	91	H	-6.14282	-0.84888	1.856493
44	C	0.133263	-2.41964	-2.1748	92	H	-2.8609	2.011906	-3.33944
45	O	-0.54861	-2.851	-1.00169	93	H	-5.0501	1.381932	-2.28825
46	C	-1.75381	3.607362	2.28368	94	H	-4.70583	-1.88272	3.712554
47	C	-1.83407	-3.34056	-1.14455	95	H	-2.40256	-2.46822	4.543236
48	C	-2.17824	4.677777	1.486053	96	H	-0.55871	1.913536	-3.43535
49	C	-3.478	5.164281	1.610016	97	H	0.293057	0.608779	-2.58372

50	C	-4.38302	4.605754	2.523458	98	H	0.007241	-2.55095	4.155085
51	C	-3.93943	3.538686	3.312299	99	H	0.420452	-0.86885	3.754656
52	C	-2.63958	3.037434	3.207584	100	H	1.762995	1.908724	-3.80904
53	C	-2.4199	-3.84889	0.019978	101	H	4.025013	2.815198	-4.15048
54	C	-3.73659	-4.29836	-0.00959	102	H	3.781006	4.914791	-0.41851
55	C	-4.4925	-4.26918	-1.19109	103	H	1.496125	4.013005	-0.06317
56	C	-3.87514	-3.79156	-2.35167	104	H	5.889404	4.744101	-1.52414
57	C	-2.55509	-3.32913	-2.34241	105	H	5.437089	5.419171	-3.08287
58	C	-5.78944	5.155967	2.687067	106	H	6.834546	-2.53498	2.956282
59	C	-5.9275	-4.7596	-1.20432	107	H	6.488073	-4.17832	3.467211
60	N	-6.81883	4.112319	2.59976	108	H	6.290662	2.639918	-2.82343
61	N	-6.78859	-3.92997	-2.05185	109	H	6.712986	-3.44134	0.646407
62	C	2.083892	-2.55916	2.422952	110	H	5.982745	1.266575	0.863202
63	C	2.876176	-1.99786	3.4304	111	H	6.019023	-0.35443	-1.00389
64	C	4.237857	-2.30094	3.475134	112	H	2.433578	3.113806	3.564873
65	C	4.831973	-3.16755	2.549087	113	H	4.656214	2.574259	2.571706
66	C	4.010978	-3.74827	1.57268	114	H	4.766076	-1.88719	-2.56287
115	H	2.601186	-2.90998	-3.24453	147	H	9.107592	-0.20281	0.396256
116	H	0.288758	-3.25821	-2.86201	148	H	-9.04224	2.875246	1.776743
117	H	-0.45551	-1.65406	-2.69769	149	H	-7.73547	1.700982	1.674067
118	H	-1.48392	5.109117	0.771215	150	H	-7.7261	2.063424	-0.86894
119	H	-3.7943	5.99321	0.980706	151	H	-9.12724	3.102054	-0.68202
120	H	-4.62697	3.083792	4.01976	152	H	-10.3758	1.17452	0.396909
121	H	-2.33618	2.21472	3.844911	153	H	-9.00789	0.127959	0.070318
122	H	-1.84596	-3.85652	0.940897	154	H	-10.6317	1.64342	-2.0504
123	H	-4.18888	-4.6664	0.908189	155	H	-10.9216	-0.02568	-1.60292
124	H	-4.44137	-3.76303	-3.27743	156	H	-9.6314	0.322155	-3.75257
125	H	-2.11733	-2.96413	-3.26442	157	H	-8.30325	1.004713	-2.83588
126	H	-5.94436	5.966058	1.950886	158	H	0.078465	2.349657	3.910573
127	H	-5.88337	5.615713	3.679541	159	H	-0.66873	1.22442	2.742069
128	H	-6.2898	-4.83215	-0.16186	160	H	10.02262	-3.78036	-0.57066
129	H	-5.96889	-5.78298	-1.60694	161	H	8.642462	-2.70505	-0.65141
130	H	-7.6711	4.469484	3.029222	162	H	8.904981	-2.32675	1.888129
131	H	-6.74238	-2.97024	-1.71384	163	H	10.23664	-3.47156	1.853942
132	H	2.459518	-1.31699	4.163525	164	H	8.532494	-5.15571	0.785185
133	H	4.849782	-1.84181	4.248425	165	H	8.45159	-4.86048	2.515734
134	H	4.445947	-4.43958	0.858257	166	H	-10.3366	-1.9722	-3.44728
135	H	2.018178	-3.89023	0.740382	167	H	-10.142	-2.3229	-1.74009
136	H	11.26297	-1.67485	0.514001	168	H	-8.53458	-3.60706	-4.02265
137	H	11.12633	-1.83953	-1.2255	169	H	-9.9519	-4.29457	-3.23952
138	H	-7.98842	-1.19884	-1.66053	170	H	-8.16182	-5.45144	-2.31633
139	H	-7.79458	-1.28589	-3.40406	171	H	-8.64475	-4.33214	-1.04462
140	H	-6.19776	3.374151	0.753288	172	H	10.015	2.35253	-2.91979
141	H	-7.48745	4.570994	0.617313	173	H	10.02168	2.532973	-1.17238
142	H	7.745965	1.41018	-1.08819	174	H	9.408211	4.629215	-2.21961
143	H	7.8181	1.196711	-2.83499	175	H	8.056897	4.047008	-1.26575
144	H	9.920946	-0.17693	-2.56516	176	H	8.166943	3.432963	-4.25287
145	H	8.444174	-0.89019	-1.94571	177	H	7.690342	5.039293	-3.72827

146	H	10.59901	0.473369	-0.24071					

Table S18. Cartesian coordinates of the complex [Cu(**C12**)]$^+$ at its optimized T_1 geometry.

atom		x	y	z	atom		x	y	z
1	C	-4.15616	2.970391	1.123933	12	C	-3.80487	-1.84575	0.698075
2	C	-4.76025	1.748561	1.142844	13	C	-2.42711	-1.68389	0.449081
3	C	-4.0038	0.552469	0.910563	14	N	-1.85675	-0.47804	0.443409
4	C	-2.61544	0.636375	0.649601	15	C	1.479154	3.118541	0.200803
5	C	-1.97114	1.929737	0.634224	16	O	2.044306	4.376922	0.528004
6	C	-2.74839	3.09034	0.878656	17	C	-1.55898	-2.90427	0.238142
7	N	-0.63445	1.97892	0.41281	18	O	-0.36869	-2.50876	-0.43563
8	C	-0.01329	3.15571	0.440316	19	C	3.41283	4.491746	0.474822
9	C	-0.70506	4.364582	0.669601	20	C	4.27511	3.47737	0.043741
10	C	-2.06734	4.32862	0.881734	21	C	5.655955	3.6961	0.051268
11	C	-4.58414	-0.73609	0.943656	22	C	6.204836	4.910615	0.473138
23	C	5.320342	5.922459	0.879018	71	C	9.680445	1.363772	-0.64962
24	C	3.942603	5.722089	0.886483	72	C	10.04452	-0.03244	-1.19053
25	C	7.706172	5.11825	0.544039	73	C	10.42335	-1.05263	-0.1051
26	C	4.101577	-5.96619	-1.19742	74	C	-8.79305	-1.39384	1.871699
27	N	8.423147	4.443515	-0.54017	75	C	-10.237	-1.28759	1.366271
28	N	4.766186	-5.57488	-2.44708	76	C	-10.3789	-1.88081	-0.04424
29	C	4.864581	-0.89654	2.347508	77	C	-11.7584	-1.71264	-0.70165
30	C	5.255534	-0.74714	1.04937	78	C	-12.1707	-0.26743	-1.04363
31	C	4.299416	-0.50214	-0.0026	79	C	-0.73666	-0.54202	3.468296
32	C	2.925924	-0.41344	0.375351	80	Cu	0.127101	-0.20314	0.094227
33	C	2.512671	-0.58529	1.702749	81	C	9.615447	-3.15666	-1.43184
34	C	3.478887	-0.82304	2.732999	82	C	8.269556	-3.25784	-0.69909
35	N	1.153393	-0.51169	1.971861	83	C	7.145238	-3.90709	-1.52706
36	C	0.751957	-0.64181	3.254098	84	C	-10.8678	-0.17284	-3.28077
37	C	1.639161	-0.86505	4.305135	85	C	-10.1571	0.726921	-4.30686
38	C	3.016446	-0.96255	4.040271	86	C	-8.81974	1.32828	-3.84538
39	C	4.621766	-0.35138	-1.3538	87	C	10.87715	2.179515	-0.13867
40	C	3.592297	-0.11657	-2.30044	88	C	10.53729	3.540661	0.496629
41	C	2.280034	-0.0086	-1.87144	89	C	9.885677	4.577363	-0.43999
42	N	1.945207	-0.1468	-0.56664	90	C	-8.10031	2.087288	-4.97123
43	O	-1.08794	-0.55367	4.852653	91	C	-6.91724	2.958411	-4.50534
44	C	1.152672	0.345576	-2.81072	92	C	7.188738	-5.44105	-1.59996
45	O	-0.09294	-0.02915	-2.18672	93	C	6.154568	-6.03188	-2.58617
46	C	-2.46849	-0.52313	5.009682	94	H	-4.72942	3.87463	1.304585
47	C	-1.26201	0.333672	-2.858	95	H	-5.82261	1.655166	1.344362
48	C	-3.17377	0.665794	4.799099	96	H	-0.15485	5.296568	0.680375
49	C	-4.56669	0.657028	4.850846	97	H	-2.62348	5.244322	1.060972
50	C	-5.27363	-0.52218	5.125979	98	H	-5.63858	-0.83746	1.180122
51	C	-4.54498	-1.68572	5.406305	99	H	-4.23258	-2.84251	0.703247
52	C	-3.14853	-1.69545	5.341843	100	H	1.674412	2.873558	-0.85272
53	C	-2.13026	-0.6819	-3.25181	101	H	1.918494	2.322967	0.813924

54	C	-3.34937	-0.34713	-3.84963	102	H	-2.10257	-3.64901	-0.35586
55	C	-3.70604	0.988304	-4.0662	103	H	-1.32249	-3.36056	1.209858
56	C	-2.80817	1.991537	-3.66875	104	H	3.898037	2.52009	-0.29569
57	C	-1.59237	1.676439	-3.06618	105	H	6.319584	2.904973	-0.28271
58	C	-6.78103	-0.56401	4.986822	106	H	5.715682	6.883489	1.200455
59	C	-5.0297	1.362285	-4.70881	107	H	3.263741	6.506586	1.207467
60	N	-7.12425	-0.89132	3.592699	108	H	8.080571	4.707629	1.492315
61	N	-5.77651	2.297658	-3.86312	109	H	7.922168	6.202731	0.584184
62	C	0.63546	-3.46782	-0.5778	110	H	4.740513	-5.85578	-0.30554
63	C	1.395964	-3.87691	0.517885	111	H	3.860412	-7.03803	-1.26698
64	C	2.495125	-4.71064	0.307648	112	H	8.115066	4.84782	-1.42271
65	C	2.843442	-5.14647	-0.97838	113	H	5.603896	-1.07634	3.123853
66	C	2.033375	-4.75844	-2.05566	114	H	6.307808	-0.80783	0.784362
67	C	0.933382	-3.92156	-1.86175	115	H	1.249355	-0.96134	5.310459
68	C	10.7348	-2.47056	-0.6243	116	H	3.719852	-1.1422	4.8487
69	C	-11.1979	0.515816	-1.94645	117	H	5.656024	-0.42061	-1.67704
70	C	-8.56247	-0.90664	3.304449	118	H	3.824501	-0.005	-3.35447
119	H	1.252294	-0.18065	-3.76638	155	H	-9.60908	-1.44023	-0.69343
120	H	1.149958	1.423515	-3.01551	156	H	-12.5258	-2.1467	-0.0446
121	H	-2.63174	1.579183	4.571442	157	H	-11.7768	-2.31422	-1.62024
122	H	-5.11398	1.574457	4.649625	158	H	-13.1535	-0.30479	-1.53521
123	H	-5.07306	-2.60579	5.646979	159	H	-12.321	0.302763	-0.11679
124	H	-2.5847	-2.60572	5.523125	160	H	-1.23269	-1.38389	2.966866
125	H	-1.85575	-1.71657	-3.07396	161	H	-1.10126	0.379414	2.998901
126	H	-4.0309	-1.14031	-4.14726	162	H	9.960599	-4.16081	-1.71413
127	H	-3.08131	3.03223	-3.81482	163	H	9.459546	-2.61731	-2.37668
128	H	-0.91709	2.464124	-2.74707	164	H	7.945373	-2.24798	-0.4174
129	H	-7.21042	0.419131	5.21471	165	H	8.398928	-3.80821	0.24498
130	H	-7.21493	-1.27961	5.708037	166	H	7.167673	-3.49021	-2.54613
131	H	-5.58212	0.44116	-4.96014	167	H	6.181306	-3.60296	-1.09782
132	H	-4.83945	1.876195	-5.66155	168	H	-11.8001	-0.5418	-3.73307
133	H	-6.74502	-1.8169	3.390439	169	H	-10.2492	-1.06197	-3.09472
134	H	1.163973	-3.50916	1.512259	170	H	-10.8307	1.549382	-4.59084
135	H	3.112196	-4.99622	1.15596	171	H	-9.98692	0.144395	-5.22386
136	H	2.288284	-5.08919	-3.05756	172	H	-8.17122	0.52792	-3.45906
137	H	0.323971	-3.59493	-2.69875	173	H	-8.99582	2.014166	-3.00455
138	H	10.99063	-3.10389	0.237385	174	H	11.57603	2.34366	-0.97344
139	H	11.63808	-2.4308	-1.25001	175	H	11.43097	1.59059	0.605356
140	H	-10.2684	0.718917	-1.39828	176	H	11.46986	3.974107	0.882838
141	H	-11.6437	1.499902	-2.15319	177	H	9.892236	3.387087	1.3729
142	H	-8.94166	0.118148	3.421068	178	H	10.29867	4.458991	-1.44941
143	H	-9.13378	-1.53677	4.01222	179	H	10.1685	5.592503	-0.10524
144	H	8.943476	1.252485	0.160083	180	H	-8.81915	2.755982	-5.46748
145	H	9.172544	1.933275	-1.43631	181	H	-7.76804	1.376456	-5.74081
146	H	10.86765	0.055656	-1.91628	182	H	7.037729	-5.85185	-0.59219
147	H	9.18422	-0.41447	-1.75594	183	H	8.18306	-5.78043	-1.92076
148	H	11.30202	-0.69457	0.44786	184	H	-6.08026	1.818593	-3.01692
149	H	9.608625	-1.10151	0.631252	185	H	-7.29346	3.707354	-3.79491

150	H	-8.45963	-2.44009	1.796334	186	H	-6.52451	3.514572	-5.36745
151	H	-8.14146	-0.81374	1.203591	187	H	4.749182	-4.55885	-2.51577
152	H	-10.5417	-0.23208	1.368428	188	H	6.159505	-7.12733	-2.51291
153	H	-10.9194	-1.80882	2.053439	189	H	6.467572	-5.78378	-3.60933
154	H	-10.1456	-2.95388	0.006622					

Table S19. Cartesian coordinates of the complex $[Cu(\mathbf{L4})_2]^+$ at its optimized T_1 geometry.

atom		x	y	z	atom		x	y	z
1	C	-2.72879	4.713421	1.482727	8	C	0.082078	2.583121	-0.84015
2	C	-3.49428	4.019883	2.37232	9	C	0.013055	3.969472	-0.98532
3	C	-3.36515	2.592017	2.523923	10	C	-0.92689	4.710856	-0.25204
4	C	-2.40709	1.928762	1.699834	11	C	-4.11708	1.823349	3.415111
5	C	-1.6296	2.630184	0.767424	12	C	-3.91709	0.42246	3.468605
6	C	-1.75897	4.051915	0.649865	13	C	-2.96157	-0.16789	2.662425
7	N	-0.73931	1.902257	-0.01173	14	N	-2.20598	0.562344	1.798479
15	C	1.125758	1.817168	-1.61234	63	C	-1.94819	-4.3611	2.147351
16	O	2.130821	1.378363	-0.69056	64	C	-1.82733	-5.71402	1.821795
17	C	-2.65634	-1.63861	2.719177	65	C	-2.06276	-6.17934	0.522258
18	O	-2.44807	-2.08747	1.368369	66	C	-2.42702	-5.24351	-0.456
19	C	3.266885	0.804128	-1.20481	67	C	-2.56269	-3.89102	-0.14995
20	C	3.463288	0.506345	-2.55979	68	C	-1.36575	-10.6142	1.238922
21	C	4.665216	-0.07764	-2.97007	69	C	-9.66762	4.252922	-3.3746
22	C	5.685672	-0.37579	-2.06006	70	C	5.43986	7.097261	2.041269
23	C	5.468835	-0.06633	-0.70922	71	C	0.377162	-11.0708	3.085549
24	C	4.278281	0.512977	-0.27891	72	C	-1.03323	-10.5774	2.739562
25	C	6.978721	-1.02229	-2.51403	73	C	4.802683	8.117756	1.095645
26	C	-1.95094	-7.65288	0.185972	74	C	5.750691	9.263073	0.717323
27	N	7.551452	-0.33599	-3.67761	75	C	-11.9905	4.879206	-2.42433
28	N	-0.71906	-8.24236	0.718905	76	C	-11.0243	4.937339	-3.61514
29	C	3.068076	-3.61656	0.166	77	C	0.598039	0.213762	3.631489
30	C	2.564612	-3.766	-1.09174	78	Cu	-1.02216	-0.15958	0.448833
31	C	1.440818	-2.98965	-1.52812	79	C	-0.53966	-9.65747	0.36408
32	C	0.835242	-2.06973	-0.63644	80	C	-9.75755	2.728501	-3.22577
33	C	1.353187	-1.92576	0.701584	81	C	8.00456	-2.13892	-6.14991
34	C	2.48245	-2.68742	1.088127	82	C	8.808134	-2.17617	-4.83994
35	N	0.749817	-1.03331	1.530774	83	C	8.871428	-0.8452	-4.07366
36	C	1.269071	-0.81723	2.73794	84	H	-2.83505	5.79093	1.389648
37	C	2.396037	-1.53081	3.204002	85	H	-4.2207	4.538241	2.992465
38	C	2.988347	-2.47103	2.390181	86	H	0.696395	4.46485	-1.66616
39	C	0.892915	-3.09148	-2.82522	87	H	-0.99458	5.78846	-0.37015
40	C	-0.19466	-2.31758	-3.17548	88	H	-4.85678	2.297022	4.05297
41	C	-0.73766	-1.42031	-2.23698	89	H	-4.50427	-0.19409	4.140888
42	N	-0.22035	-1.29635	-1.01131	90	H	1.572037	2.469912	-2.36993
43	O	1.488915	0.781051	4.580096	91	H	0.688873	0.950885	-2.12019
44	C	-1.90933	-0.54331	-2.60794	92	H	-3.4803	-2.19453	3.17728
45	O	-2.76949	-0.45715	-1.47706	93	H	-1.74698	-1.82229	3.306543

46	C	2.327851	1.799641	4.182585	94	H	2.699417	0.720144	-3.29867
47	C	-3.97458	0.207123	-1.63085	95	H	4.813828	-0.29648	-4.02348
48	C	2.47469	2.245948	2.863714	96	H	6.247617	-0.27695	0.02017
49	C	3.358467	3.294208	2.589471	97	H	4.121257	0.758782	0.766807
50	C	4.104899	3.910876	3.599633	98	H	6.788741	-2.06609	-2.79861
51	C	3.940073	3.446786	4.912575	99	H	7.681871	-1.06224	-1.66088
52	C	3.067206	2.402314	5.207273	100	H	-2.80027	-8.19197	0.626522
53	C	-4.89873	0.04044	-0.59465	101	H	-2.04265	-7.78256	-0.90857
54	C	-6.13497	0.677785	-0.66697	102	H	7.643981	0.650823	-3.44251
55	C	-6.47602	1.487973	-1.75974	103	H	3.926599	-4.19608	0.491148
56	C	-5.53801	1.632799	-2.78794	104	H	3.009396	-4.4691	-1.78876
57	C	-4.29231	1.000728	-2.73679	105	H	2.774689	-1.32515	4.19785
58	C	5.081097	5.026223	3.282324	106	H	3.848442	-3.03882	2.732639
59	C	-7.81261	2.200588	-1.81509	107	H	1.326235	-3.78764	-3.53697
60	N	4.513349	6.005936	2.352728	108	H	-0.64077	-2.3946	-4.16056
61	N	-8.43446	2.091155	-3.13919	109	H	-2.44107	-0.97297	-3.46369
62	C	-2.31592	-3.4475	1.153355	110	H	-1.55227	0.455148	-2.89694
111	H	1.929124	1.794402	2.043005	141	H	6.09139	9.770398	1.630827
112	H	3.463027	3.641996	1.566303	142	H	-12.9093	5.43767	-2.63827
113	H	4.500809	3.911468	5.720325	143	H	-12.2801	3.850977	-2.18
114	H	2.937423	2.049975	6.226128	144	H	-11.5036	4.489986	-4.49788
115	H	-4.63942	-0.59201	0.247482	145	H	-10.8401	5.989322	-3.87025
116	H	-6.84975	0.536293	0.140191	146	H	-0.17779	-0.27027	4.233756
117	H	-5.78899	2.242682	-3.65023	147	H	0.114888	0.983482	3.023457
118	H	-3.59412	1.136418	-3.55508	148	C	8.60523	-1.23454	-7.23312
119	H	5.979319	4.609464	2.802587	149	H	7.931249	-3.16282	-6.5408
120	H	5.428011	5.48268	4.228073	150	H	9.841596	-2.4855	-5.05249
121	H	-7.66426	3.267516	-1.60146	151	H	8.391323	-2.95378	-4.18578
122	H	-8.4626	1.816203	-1.00699	152	H	9.333921	-0.08105	-4.70871
123	H	3.673963	6.400747	2.777945	153	H	9.539541	-0.97538	-3.20105
124	H	-1.75794	-4.0393	3.164981	154	H	-8.54268	1.099734	-3.34859
125	H	-1.53494	-6.42126	2.591937	155	H	-10.2547	2.319807	-4.11461
126	H	-2.6159	-5.57672	-1.47367	156	H	-10.3923	2.469222	-2.35907
127	H	-2.86067	-3.17018	-0.90385	157	H	0.067376	-7.71316	0.346306
128	H	-2.43461	-10.4013	1.102842	158	H	-0.7871	-9.8477	-0.69728
129	H	-1.21145	-11.6327	0.85448	159	H	0.525634	-9.88649	0.480519
130	H	-9.20074	4.693055	-2.48245	160	H	-11.5352	5.317945	-1.52734
131	H	-8.99645	4.468617	-4.21625	161	C	5.10725	10.28672	-0.22437
132	H	6.326591	6.656528	1.564286	162	H	0.535188	-11.0783	4.170448
133	H	5.802238	7.613613	2.951503	163	H	6.979703	-1.81526	-5.93115
134	H	0.540711	-12.0921	2.718116	164	H	4.78665	9.815139	-1.1616
135	H	1.152654	-10.4323	2.646941	165	H	5.807564	11.09104	-0.47754
136	H	-1.7676	-11.196	3.272847	166	H	4.223018	10.74573	0.235166
137	H	-1.16276	-9.55283	3.108902	167	H	9.636871	-1.52786	-7.46655
138	H	3.901218	8.535506	1.569147	168	H	8.620791	-0.183	-6.92422
139	H	4.46476	7.597804	0.188916	169	H	8.024304	-1.29419	-8.16104
140	H	6.651215	8.846573	0.244277					

Table S20. Cartesian coordinates of the complex $[Cu(\mathbf{L5})_2]^+$ at its optimized T_1 geometry.

atom		x	y	z	atom		x	y	z
1	C	-3.7741	4.03046	1.383397	14	N	-2.34897	0.108701	1.817668
2	C	-4.38109	3.206514	2.283647	15	C	0.663882	1.982688	-1.60723
3	C	-3.9411	1.846991	2.475379	16	O	1.735466	1.800461	-0.6738
4	C	-2.84676	1.393432	1.679305	17	C	-2.31291	-2.11346	2.79464
5	C	-2.22984	2.226337	0.735595	18	O	-1.99394	-2.54013	1.458223
6	C	-2.66888	3.580476	0.578047	19	C	2.969989	1.463166	-1.172
7	N	-1.18865	1.69471	-0.01509	20	C	3.233169	1.18253	-2.51948
8	C	-0.52909	2.519404	-0.85813	21	C	4.531838	0.847556	-2.91265
9	C	-0.9011	3.851745	-1.04098	22	C	5.584783	0.783221	-1.99317
10	C	-1.99086	4.38518	-0.33431	23	C	5.30002	1.073861	-0.65099
11	C	-4.51766	0.952483	3.379492	24	C	4.013052	1.407778	-0.23736
12	C	-4.01227	-0.36683	3.474124	25	C	6.983779	0.399823	-2.43074
13	C	-2.93644	-0.74926	2.694114	26	C	-0.21547	-7.87202	0.441172
27	N	7.433739	1.196084	-3.57797	75	C	-12.7857	2.136399	-2.50224
28	N	1.103739	-8.1498	1.017327	76	C	-11.836	2.395422	-3.68188
29	C	3.724171	-2.87452	0.338294	77	C	0.449859	0.426733	3.680005
30	C	3.279042	-3.16219	-0.91727	78	Cu	-1.01149	-0.36316	0.500008
31	C	2.021469	-2.65447	-1.38302	79	C	1.621209	-9.4865	0.69379
32	C	1.224548	-1.85833	-0.52342	80	C	-10.1237	0.509071	-3.29306
33	C	1.684218	-1.57088	0.812957	81	C	8.2226	-0.21569	-6.23094
34	C	2.943729	-2.06566	1.229229	82	C	8.928574	-0.3511	-4.87503
35	N	0.894559	-0.80568	1.612253	83	C	8.813024	0.902213	-3.99337
36	C	1.338339	-0.45603	2.818051	84	H	-4.11459	5.055023	1.259674
37	C	2.585124	-0.9012	3.311992	85	H	-5.21414	3.565588	2.882001
38	C	3.374988	-1.71328	2.528556	86	H	-0.33665	4.468111	-1.73193
39	C	1.522275	-2.90893	-2.67864	87	H	-2.29375	5.417761	-0.48197
40	C	0.299829	-2.39335	-3.0585	88	H	-5.35404	1.265723	3.996608
41	C	-0.43085	-1.60317	-2.15169	89	H	-4.45769	-1.08184	4.15764
42	N	0.033733	-1.33756	-0.92739	90	H	0.959587	2.697414	-2.38247
43	O	1.186058	1.202656	4.613819	91	H	0.431694	1.028068	-2.09099
44	C	-1.75736	-1.00473	-2.55379	92	H	-2.99849	-2.82594	3.263796
45	O	-2.63286	-1.08849	-1.43394	93	H	-1.39269	-2.07399	3.392383
46	C	1.795584	2.362107	4.185609	94	H	2.447268	1.218554	-3.26511
47	C	-3.952	-0.70753	-1.61205	95	H	4.729228	0.639552	-3.96031
48	C	1.841347	2.797916	2.855713	96	H	6.099966	1.04228	0.085225
49	C	2.487307	3.999763	2.550676	97	H	3.801286	1.639694	0.801786
50	C	3.094485	4.781215	3.540209	98	H	6.999177	-0.65677	-2.73026
51	C	3.034525	4.324244	4.864345	99	H	7.66944	0.481266	-1.56652
52	C	2.397493	3.129122	5.190007	100	H	-0.93019	-8.58156	0.879275
53	C	-4.82744	-1.04623	-0.5752	101	H	-0.24549	-8.04493	-0.65091
54	C	-6.1719	-0.69652	-0.66854	102	H	7.374139	2.179004	-3.31827
55	C	-6.67177	-0.00749	-1.78339	103	H	4.681789	-3.25016	0.685383
56	C	-5.77902	0.312217	-2.81213	104	H	3.871045	-3.77376	-1.59085
57	C	-4.42554	-0.03107	-2.73984	105	H	2.897728	-0.59536	4.303094
58	C	3.812454	6.068388	3.18795	106	H	4.331315	-2.07624	2.893482
59	C	-8.13079	0.397462	-1.85653	107	H	2.100839	-3.51921	-3.3654

60	N	3.012919	6.915761	2.298632	108	H	-0.10881	-2.59257	-4.04267
61	N	-8.69509	0.174041	-3.1914	109	H	-2.17003	-1.5517	-3.40798
62	C	-1.55334	-3.84012	1.283745	110	H	-1.61923	0.042951	-2.8559
63	C	-1.00142	-4.61893	2.306547	111	H	1.398944	2.224144	2.049552
64	C	-0.56866	-5.91596	2.020976	112	H	2.512266	4.336337	1.518601
65	C	-0.67006	-6.45592	0.732934	113	H	3.490798	4.913421	5.656468
66	C	-1.22611	-5.65484	-0.27436	114	H	2.347917	2.781612	6.217537
67	C	-1.67389	-4.36255	-0.00804	115	H	-4.44396	-1.58606	0.283871
68	C	0.982708	-10.6084	1.52809	116	H	-6.8459	-0.97038	0.139679
69	C	-10.3663	2.018595	-3.42808	117	H	-6.14974	0.830302	-3.69109
70	C	3.685202	8.172945	1.956294	118	H	-3.76698	0.233338	-3.55943
71	C	2.590917	-10.476	3.576117	119	H	4.749518	5.840069	2.658698
72	C	1.144217	-10.464	3.051282	120	H	4.108979	6.584492	4.120047
73	C	2.790174	9.051639	1.077326	121	H	-8.222	1.467947	-1.6283
74	C	3.463892	10.33527	0.564669	122	H	-8.69341	-0.13019	-1.06373
123	H	2.134794	7.133518	2.770707	153	H	-10.5089	0.011581	-4.19206
124	H	-0.90502	-4.23572	3.316035	154	H	-10.6975	0.108216	-2.43785
125	H	-0.13391	-6.51647	2.814051	155	H	1.755325	-7.455	0.656568
126	H	-1.31855	-6.04872	-1.28363	156	H	1.490366	-9.7289	-0.37794
127	H	-2.11797	-3.74935	-0.78492	157	H	2.701405	-9.46536	0.877965
128	H	-0.0885	-10.6734	1.294492	158	H	2.798112	10.80575	-0.17258
129	H	1.415001	-11.559	1.188479	159	H	2.562736	-10.2982	4.659849
130	H	-10.0153	2.539573	-2.52646	160	H	7.183865	0.087409	-6.05187
131	H	-9.7482	2.384342	-4.25839	161	H	4.506497	10.94112	2.371788
132	H	4.603997	7.921978	1.407001	162	H	2.888016	11.6066	2.21528
133	H	4.003751	8.729989	2.856644	163	C	4.392817	12.66115	1.072528
134	C	3.353892	-11.7792	3.307355	164	H	5.32236	12.45977	0.525361
135	H	3.148104	-9.63078	3.150101	165	H	4.621785	13.38477	1.8635
136	H	0.587425	-11.2786	3.536744	166	H	3.694703	13.13902	0.373746
137	H	0.664668	-9.52933	3.366441	167	H	3.487197	-11.9621	2.234785
138	H	1.881982	9.318279	1.63906	168	H	2.819807	-12.6419	3.726068
139	H	2.459103	8.448508	0.221931	169	H	4.351261	-11.7525	3.761813
140	H	4.381691	10.07046	0.018553	170	C	9.627896	-1.93201	-7.55525
141	C	3.800448	11.37028	1.648894	171	H	7.600299	-1.32797	-7.97029
142	H	-12.2026	1.8552	-4.56788	172	H	7.763901	-2.31029	-6.52373
143	H	-11.8819	3.463323	-3.93818	173	H	10.12152	-1.13395	-8.12461
144	H	-0.20276	-0.20038	4.296083	174	H	9.566173	-2.81401	-8.20357
145	H	-0.18753	1.053457	3.050052	175	H	10.2792	-2.1887	-6.71197
146	H	8.68893	0.604703	-6.79815	176	C	-14.2183	2.60524	-2.78026
147	C	8.23446	-1.49207	-7.08826	177	H	-12.3985	2.650312	-1.61053
148	H	9.997326	-0.55511	-5.02146	178	H	-12.798	1.066344	-2.25701
149	H	8.526192	-1.22204	-4.33764	179	H	-14.248	3.681584	-2.99157
150	H	9.172881	1.768251	-4.56287	180	H	-14.8756	2.414471	-1.92392
151	H	9.486001	0.793481	-3.12215	181	H	-14.6428	2.086031	-3.64874
152	H	-8.58045	-0.8136	-3.41439					

Table S21. Cartesian coordinates of the complex $[Cu(\mathbf{L6})_2]^+$ at its optimized T_1 geometry.

atom		x	y	z	atom		x	y	z
1	C	-3.62417	-3.96262	-1.68169	14	N	-2.36745	0.039615	-1.77851
2	C	-4.30096	-3.07984	-2.46931	15	C	0.862023	-2.03713	1.317641
3	C	-3.92134	-1.69089	-2.54111	16	O	1.886722	-1.72758	0.365443
4	C	-2.80996	-1.27247	-1.74932	17	C	-2.4623	2.347734	-2.52974
5	C	-2.12089	-2.16831	-0.91977	18	O	-2.09617	2.655486	-1.17321
6	C	-2.50212	-3.54843	-0.88067	19	C	3.1261	-1.38317	0.847114
7	N	-1.06776	-1.67097	-0.16253	20	C	3.431605	-1.20732	2.20239
8	C	-0.34223	-2.54644	0.567553	21	C	4.732207	-0.85497	2.577013
9	C	-0.65696	-3.90429	0.632132	22	C	5.745835	-0.67523	1.630171
10	C	-1.7559	-4.41011	-0.0804	23	C	5.419272	-0.8632	0.278315
11	C	-4.57179	-0.73666	-3.32622	24	C	4.130124	-1.20846	-0.11611
12	C	-4.12165	0.605591	-3.30818	25	C	7.147302	-0.2654	2.038689
13	C	-3.02664	0.954352	-2.53921	26	C	-0.52608	7.946082	0.301558
27	N	7.589725	-0.95096	3.256008	75	C	-12.5102	-2.77522	2.815872
28	N	0.666509	8.417388	-0.40758	76	C	-11.4994	-3.0896	3.929368
29	C	3.667136	3.093288	-0.28285	77	C	0.343438	0.014946	-3.78473
30	C	3.275314	3.238239	1.014405	78	Cu	-0.98793	0.434208	-0.47851
31	C	2.059522	2.640201	1.484023	79	C	1.131074	9.740731	0.03066
32	C	1.247022	1.905004	0.585545	80	C	-9.87309	-1.11451	3.617053
33	C	1.64979	1.767962	-0.79241	81	C	8.322511	0.962354	5.552725
34	C	2.870724	2.347687	-1.21397	82	C	9.159472	0.723673	4.284048
35	N	0.847327	1.056012	-1.62742	83	C	8.979346	-0.65571	3.629886
36	C	1.243118	0.842398	-2.88071	84	H	-3.92018	-5.00769	-1.64791
37	C	2.448909	1.380347	-3.38401	85	H	-5.14588	-3.41249	-3.06627
38	C	3.249075	2.140785	-2.5603	86	H	-0.04122	-4.56154	1.236112
39	C	1.617817	2.744273	2.820778	87	H	-2.01413	-5.46363	-0.024
40	C	0.43263	2.148825	3.202009	88	H	-5.42281	-1.02237	-3.93663
41	C	-0.31725	1.428595	2.25361	89	H	-4.62455	1.365366	-3.89699
42	N	0.094329	1.303788	0.988633	90	H	1.215018	-2.80916	2.009578
43	O	1.058074	-0.63621	-4.82469	91	H	0.61704	-1.14187	1.898456
44	C	-1.60335	0.746089	2.652919	92	H	-3.1981	3.071254	-2.89421
45	O	-2.53187	0.901397	1.584619	93	H	-1.5714	2.409696	-3.16901
46	C	1.726105	-1.80826	-4.54287	94	H	2.677831	-1.33787	2.970331
47	C	-3.82857	0.458027	1.782998	95	H	4.963764	-0.72735	3.630056
48	C	1.857672	-2.36693	-3.26581	96	H	6.188865	-0.74177	-0.48069
49	C	2.55747	-3.56757	-3.11185	97	H	3.886869	-1.3609	-1.16307
50	C	3.134904	-4.2281	-4.20201	98	H	7.169922	0.815502	2.234874
51	C	2.988086	-3.64994	-5.47097	99	H	7.834244	-0.43173	1.187268
52	C	2.296587	-2.45367	-5.64634	100	H	-1.34908	8.636522	0.072266
53	C	-4.76313	0.863392	0.82488	101	H	-0.40429	7.974911	1.400702
54	C	-6.08993	0.457833	0.944075	102	H	7.503629	-1.95381	3.09946
55	C	-6.51323	-0.35289	2.007459	103	H	4.59361	3.537473	-0.63361
56	C	-5.56194	-0.73817	2.958416	104	H	3.879667	3.80195	1.717999
57	C	-4.22516	-0.34118	2.859062	105	H	2.722437	1.185161	-4.41385
58	C	3.915754	-5.5134	-4.01394	106	H	4.173879	2.573206	-2.93062
59	C	-7.95353	-0.8145	2.108958	107	H	2.210733	3.302204	3.539175

60	N	3.220995	-6.45405	-3.13088	108	H	0.067913	2.232106	4.219549
61	N	-8.46274	-0.71947	3.481083	109	H	-1.99441	1.194571	3.572191
62	C	-1.7065	3.952117	-0.88874	110	H	-1.41628	-0.31935	2.847672
63	C	-1.26752	4.863851	-1.85432	111	H	1.4406	-1.88969	-2.3866
64	C	-0.88219	6.14749	-1.45881	112	H	2.648711	-4.00059	-2.12038
65	C	-0.91891	6.542722	-0.11629	113	H	3.418738	-4.14401	-6.33887
66	C	-1.36028	5.609101	0.832524	114	H	2.179746	-2.01238	-6.63156
67	C	-1.76075	4.328932	0.457388	115	H	-4.43908	1.497507	0.006708
68	C	0.291131	10.90032	-0.52924	116	H	-6.81061	0.783903	0.197913
69	C	-10.0568	-2.63798	3.643879	117	H	-5.87301	-1.35164	3.798227
70	C	3.953871	-7.70965	-2.9489	118	H	-3.51937	-0.65976	3.617786
71	C	1.584386	11.30378	-2.72803	119	H	4.888472	-5.29376	-3.54908
72	C	0.235623	10.98841	-2.06318	120	H	4.145455	-5.94569	-5.0057
73	C	3.180772	-8.6833	-2.05669	121	H	-8.02046	-1.86576	1.798173
74	C	3.930299	-9.99927	-1.80994	122	H	-8.56856	-0.24517	1.38717
123	H	2.310513	-6.66372	-3.54104	159	H	2.167787	9.845218	-0.30835
124	H	-1.2228	4.594614	-2.90346	160	H	-12.145	-3.19749	1.867344
125	H	-0.53626	6.852578	-2.2081	161	C	3.148983	-10.9764	-0.91953
126	H	-1.3998	5.888657	1.882618	162	C	1.5003	11.54667	-4.24493
127	H	-2.11756	3.613302	1.190315	163	H	7.260511	0.887062	5.289702
128	H	-0.73262	10.83047	-0.13777	164	H	2.89561	-10.4683	0.021939
129	H	0.703969	11.83415	-0.12143	165	C	3.894295	-12.2812	-0.59109
130	H	-9.72847	-3.0747	2.690484	166	H	2.190562	-11.2198	-1.40248
131	H	-9.38959	-3.04173	4.416555	167	C	10.03439	0.004425	7.275426
132	H	4.918787	-7.46831	-2.48114	168	H	8.3356	-1.021	6.422919
133	H	4.191246	-8.2009	-3.91269	169	H	7.908553	0.256205	7.544538
134	H	2.013004	12.19761	-2.25118	170	H	4.85257	-12.0376	-0.10992
135	H	2.294583	10.48608	-2.54	171	C	4.147084	-13.1904	-1.80042
136	H	-0.48392	11.77259	-2.34021	172	H	3.312052	-12.838	0.155642
137	H	-0.16404	10.04619	-2.45569	173	H	-14.2772	-2.90551	4.054667
138	H	2.207093	-8.90184	-2.52083	174	H	-13.8546	-4.41065	3.252484
139	H	2.964598	-8.19029	-1.09924	175	C	-14.9239	-3.01349	1.993363
140	H	4.901302	-9.78172	-1.34021	176	H	4.793676	-12.7114	-2.54437
141	H	4.155717	-10.4709	-2.77607	177	H	3.204987	-13.4525	-2.29886
142	C	-13.9146	-3.32287	3.104316	178	H	4.63311	-14.1245	-1.49516
143	H	-12.5766	-1.68939	2.660957	179	H	-15.0314	-1.93144	1.846449
144	H	-11.8427	-2.6351	4.870968	180	H	-15.9153	-3.41804	2.228783
145	H	-11.4953	-4.17519	4.101115	181	H	-14.605	-3.44703	1.037187
146	H	-0.36068	0.673759	-4.30326	182	H	10.73723	-0.29964	6.487744
147	H	-0.23916	-0.69549	-3.19185	183	H	10.10593	-0.76766	8.053651
148	C	8.596829	0.002063	6.725151	184	C	10.48262	1.347383	7.865424
149	H	8.482501	1.997394	5.883154	185	H	10.51098	2.137775	7.106545
150	H	10.22838	0.845504	4.505071	186	H	11.48676	1.271699	8.298918
151	H	8.919931	1.508758	3.554276	187	H	9.800267	1.67566	8.66004
152	H	9.295029	-1.43554	4.332181	188	H	0.795729	12.36821	-4.43984
153	H	9.660826	-0.72168	2.760381	189	C	1.086058	10.31616	-5.06185
154	H	-8.37128	0.25091	3.77813	190	H	2.479842	11.89499	-4.6
155	H	-10.2337	-0.70236	4.567963	191	H	0.077466	9.974948	-4.80247

156	H	-10.4986	-0.6711	2.821239	192	H	1.774526	9.479174	-4.88795
157	H	1.413602	7.743895	-0.2471	193	H	1.093619	10.53577	-6.13596
158	H	1.150778	9.823642	1.133903					

5. Photophysical Studies

Steady absorption spectra were recorded using a Varian Cary 50 spectrophotometer. Samples for UV-Vis absorption measurement were contained in a quartz cuvette with a path length of 1 cm and 1.5 mL cell volume. Stock solutions of [Cu(**Cn**)](PF_6) (n = 7 to 12), [Cu(**Ln**)$_2$](PF_6) (n = 4 to 6), [Cu(**C8-Ms**)](PF_6), [Cu(**C8-Ipps**)](PF_6), [Cu(**C8-Tipps**)](PF_6) and [Cu(**C8-Fmoc**)](PF_6) at 1 mM were prepared in MeCN. All the samples were mixed with a plastic disposable pipette prior to spectra collection.

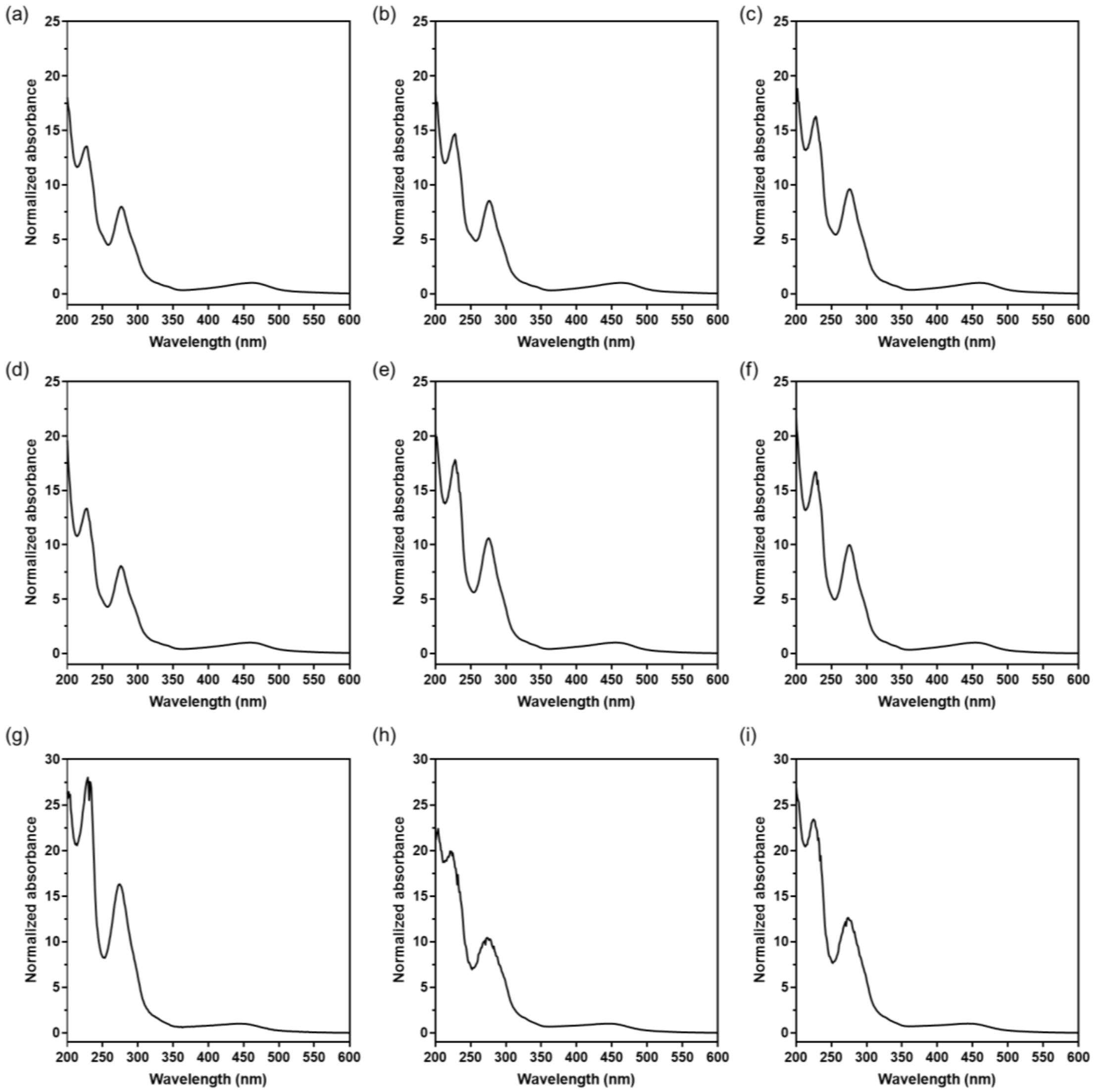

Figure S35. Normalized steady-state UV-Vis absorption spectra of a 30 μM solution of (a) [Cu(**C7**)]$^+$; (b) [Cu(**C8**)]$^+$; (c) [Cu(**C9**)]$^+$; (d) [Cu(**C10**)]$^+$; (e) [Cu(**C11**)]$^+$; (f) [Cu(**C12**)]$^+$; (g) [Cu(**L4**)$_2$]$^+$; (h) [Cu(**L5**)$_2$]$^+$; and (i) [Cu(**L6**)$_2$]$^+$ in MeCN.

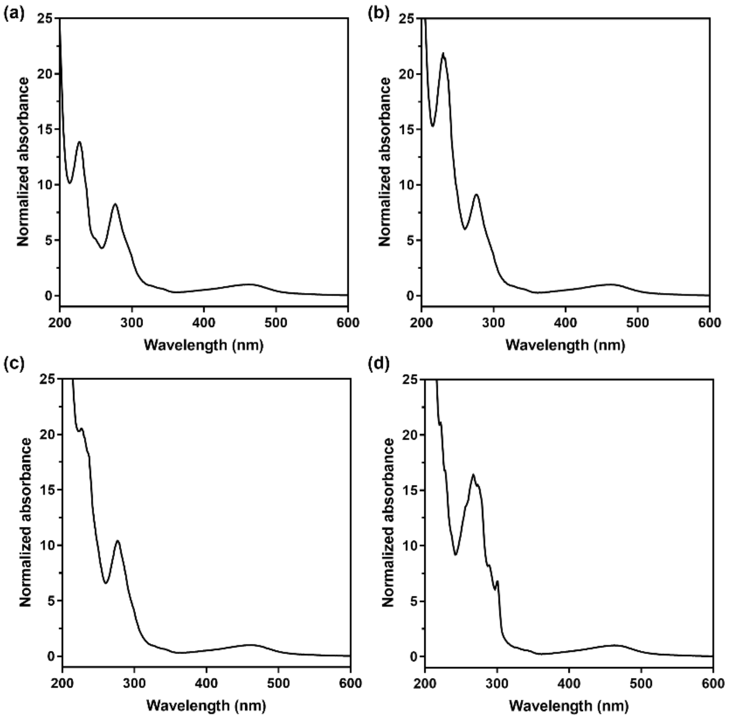

Figure S36. Normalized steady-state UV-Vis absorption spectra of a 30 μM solution of (a) [Cu(**C8-Ms**)]$^+$; (b) [Cu(**C8-Ipps**)]$^+$; (c) [Cu(**C8-Tipps**)]$^+$; and (d) [Cu(**C8-Fmoc**)]$^+$ in MeCN.

Transient absorption spectra were obtained according to the setups and methods described in the literature,[16,17] using a commercialized Helios pump–probe system (Ultrafast System) with the femtosecond laser beam from the regenerative amplified Ti:sapphire laser system (Spectra Physics, Spitfire Pro). The laser light (120 fs, 800 nm) was split into two beams with one used as the pump beam and the other as the probe beam. Wavelength of the pump beam was set as 400 nm (the third harmonic of the fundamental 800 nm), while the probe beam passed through a Ti:Sapphire crystal and generated a white-light continuum (430–750 nm). After photoexcitation of the sample by the pump light, the time-delayed probe beam (controlled by the optical delay rail with a maximum temporal delay at 3.3 ns) was passed through the photoexcited sample, and the TA signals were collected by the detector. A reference probe beam was also used to obtain a better signal-to-noise ratio. Samples was prepared at a concentration such that the absorbance at 400 nm is 0.3. The spectra were recorded in a 2 mm path-length quartz cuvette.

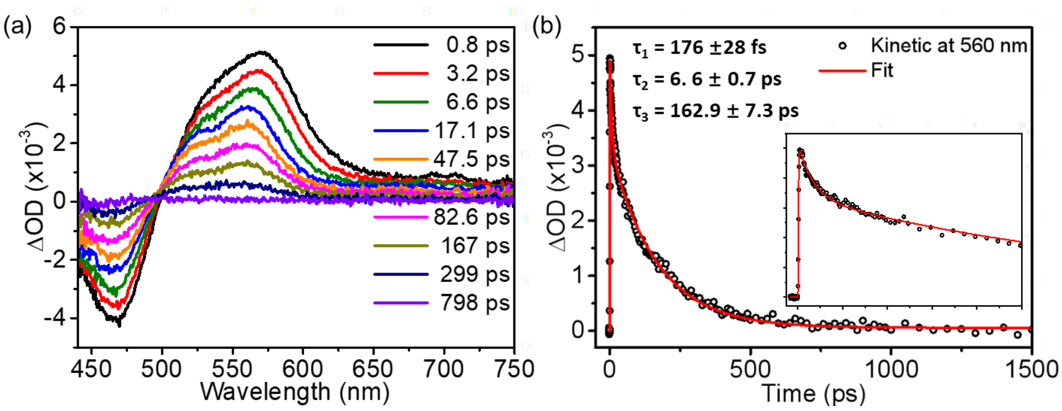

Figure S37. (a) Fs-TA spectra of [Cu(**C7**)]$^+$ in MeCN upon the excitation of 400 nm. (d) Kinetic recorded at 560 nm (inset shows the kinetics within the first 100 ps).

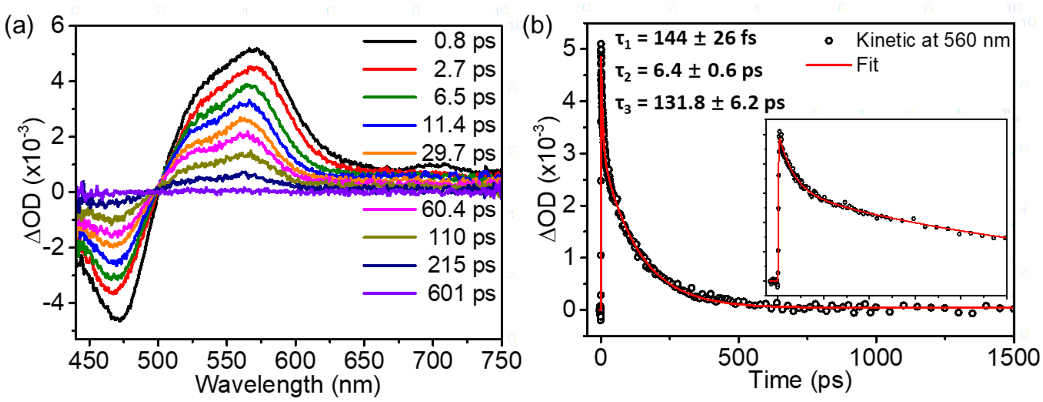

Figure S38. (a) Fs-TA spectra of [Cu(**C8**)]$^+$ in MeCN upon the excitation of 400 nm. (d) Kinetic recorded at 560 nm (inset shows the kinetics within the first 100 ps).

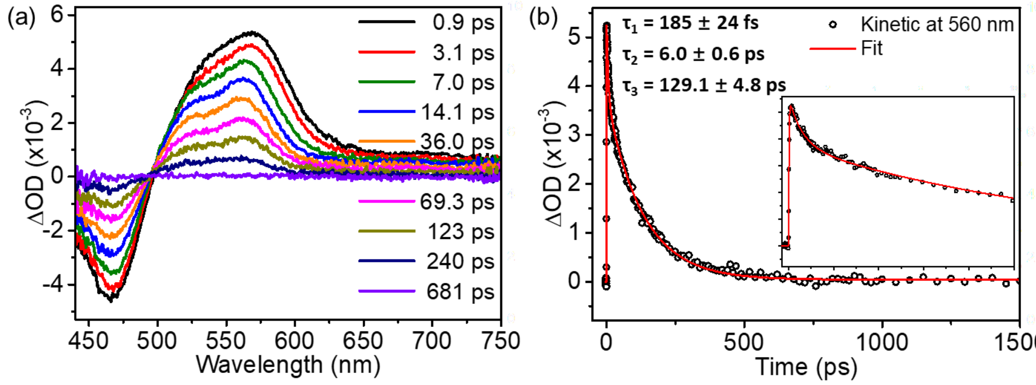

Figure S39. (a) Fs-TA spectra of [Cu(**C9**)]$^+$ in MeCN upon the excitation of 400 nm. (d) Kinetic recorded at 560 nm (inset shows the kinetics within the first 100 ps).

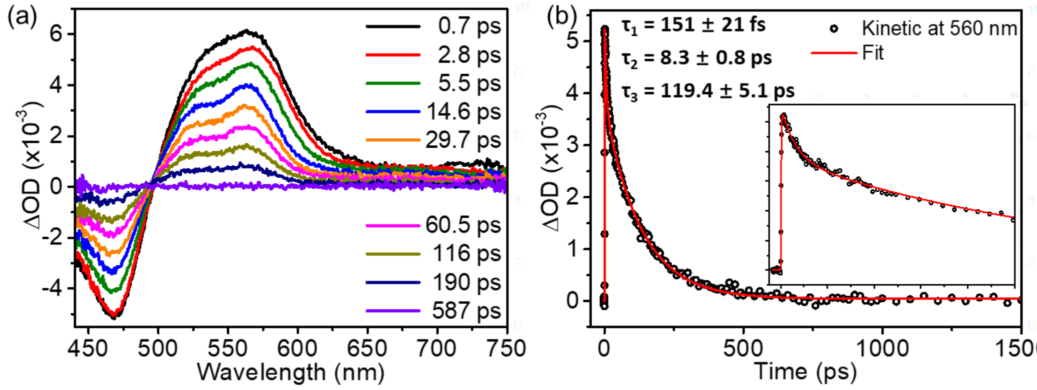

Figure S40. (a) Fs-TA spectra of [Cu(**C10**)]$^+$ in MeCN upon the excitation of 400 nm. (d) Kinetic recorded at 560 nm (inset shows the kinetics within the first 100 ps).

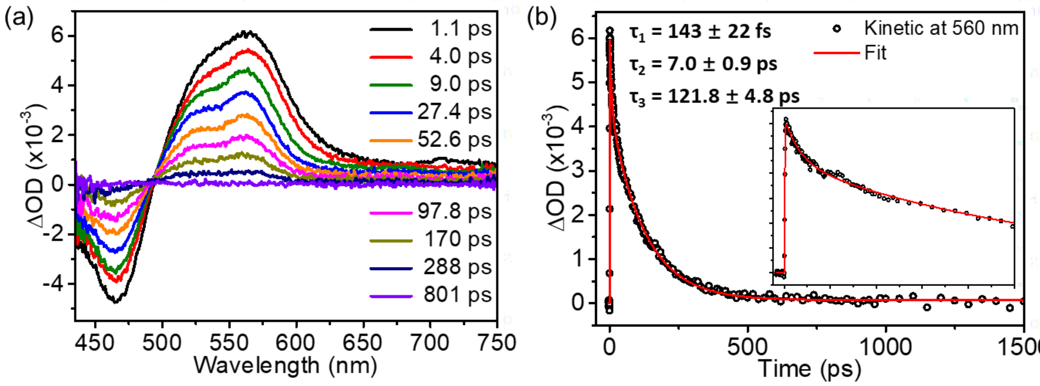

Figure S41. (a) Fs-TA spectra of [Cu(**C11**)]$^+$ in MeCN upon the excitation of 400 nm. (d) Kinetic recorded at 560 nm (inset shows the kinetics within the first 100 ps).

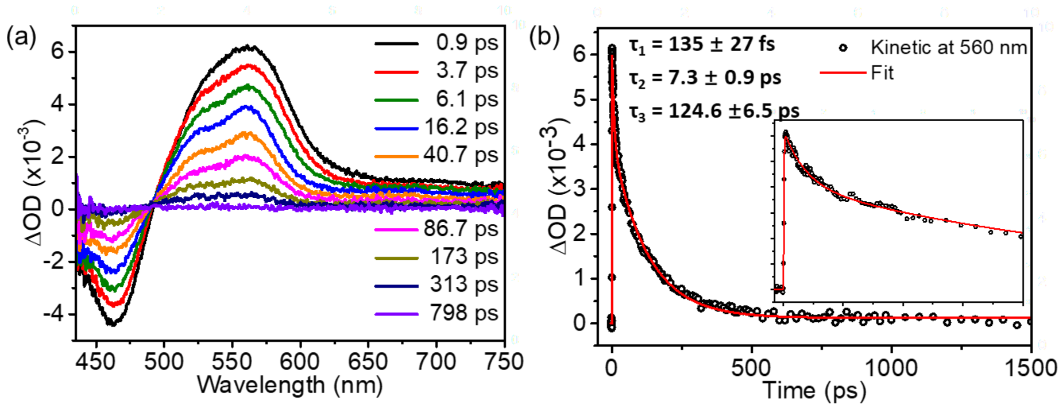

Figure S42. (a) Fs-TA spectra of [Cu(**C12**)]$^{+}$ in MeCN upon the excitation of 400 nm. (d) Kinetic recorded at 560 nm (inset shows the kinetics within the first 100 ps).

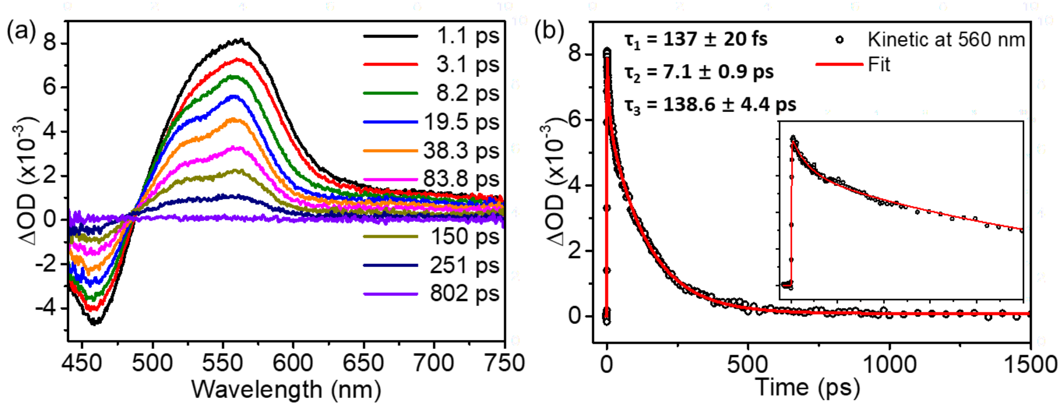

Figure S43. (a) Fs-TA spectra of [Cu(**L4**)$_2$]$^{+}$ in MeCN upon the excitation of 400 nm. (d) Kinetic recorded at 560 nm (inset shows the kinetics within the first 100 ps).

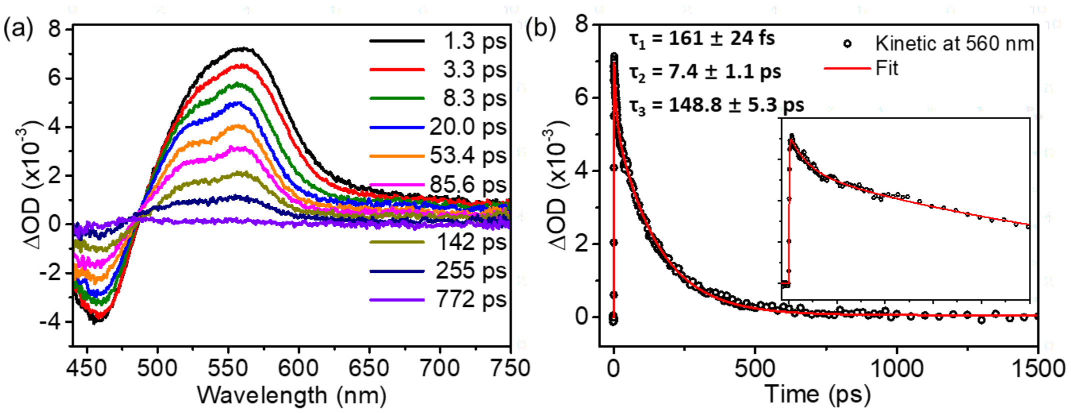

Figure S44. (a) Fs-TA spectra of [Cu(**L5**)$_2$]$^{+}$ in MeCN upon the excitation of 400 nm. (d) Kinetic recorded at 560 nm (inset shows the kinetics within the first 100 ps).

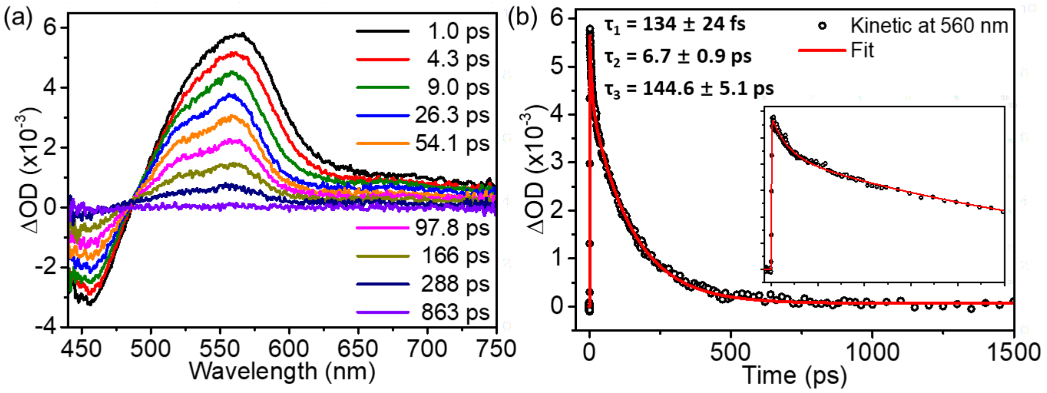

Figure S45. (a) Fs-TA spectra of $[Cu(\mathbf{L6})_2]^+$ in MeCN upon the excitation of 400 nm. (d) Kinetic recorded at 560 nm (inset shows the kinetics within the first 100 ps).

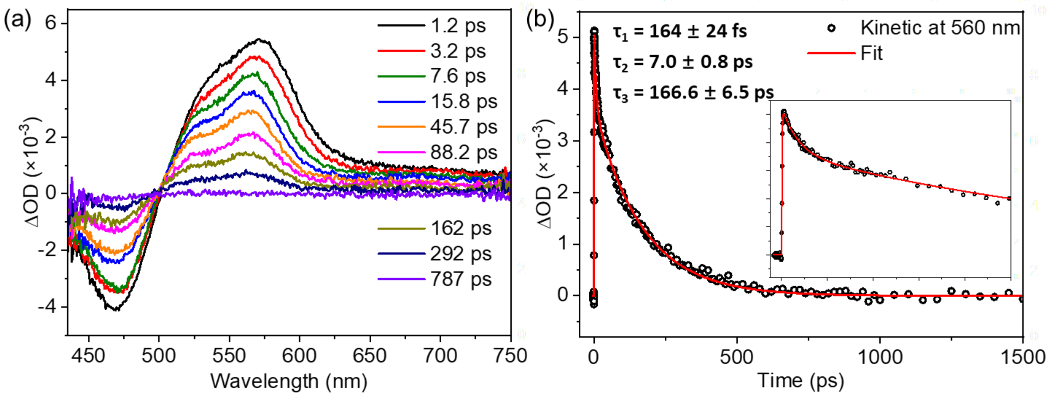

Figure S46. (a) Fs-TA spectra of $[Cu(\mathbf{C8})]^+$ in THF upon the excitation of 400 nm. (d) Kinetic recorded at 560 nm (inset shows the kinetics within the first 100 ps).

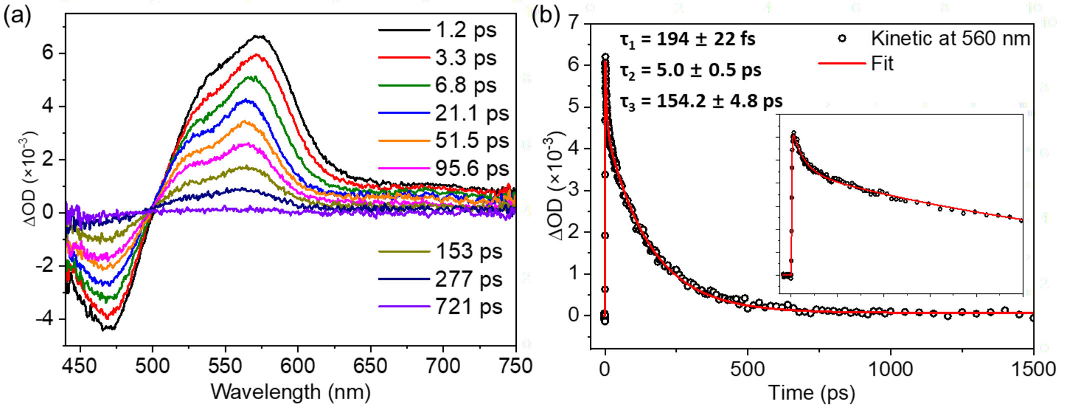

Figure S47. (a) Fs-TA spectra of $[Cu(\mathbf{C8})]^+$ in CH_2Cl_2 upon the excitation of 400 nm. (d) Kinetic recorded at 560 nm (inset shows the kinetics within the first 100 ps).

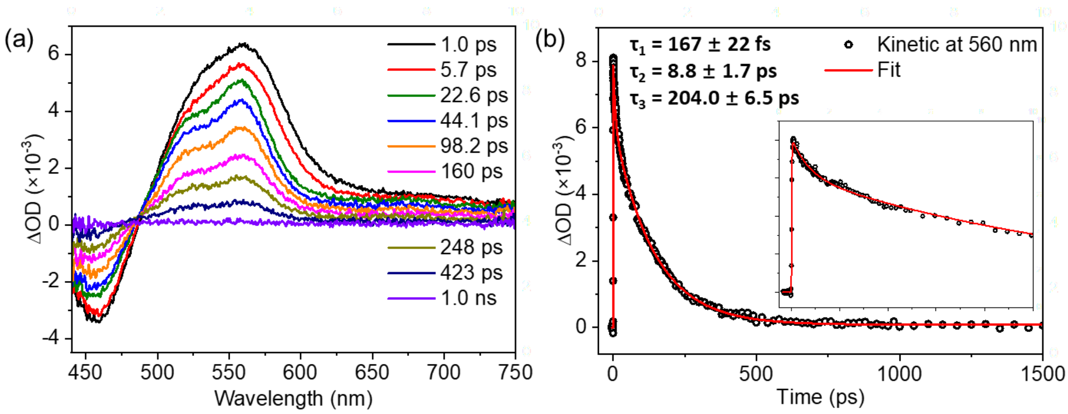

Figure S48. (a) Fs-TA spectra of [Cu(**L4**)$_2$]$^+$ in THF upon the excitation of 400 nm. (d) Kinetic recorded at 560 nm (inset shows the kinetics within the first 100 ps).

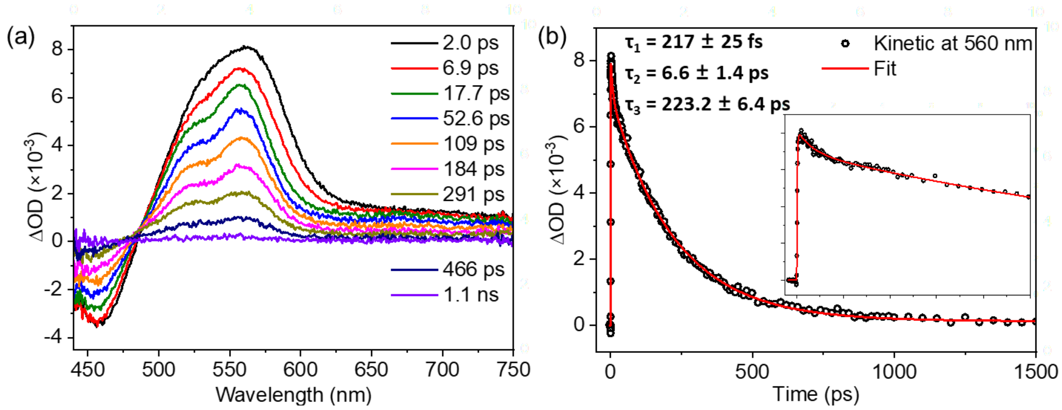

Figure S49. (a) Fs-TA spectra of [Cu(**L4**)$_2$]$^+$ in CH_2Cl_2 upon the excitation of 400 nm. (d) Kinetic recorded at 560 nm (inset shows the kinetics within the first 100 ps).

Table S22. A summary of the excited state dynamic data of the catenane and non-interlocked complexes.

	solvent	τ_1 / fs	τ_2 / ps	τ_3 / ps
[Cu(**C7**)]$^+$	MeCN	176 ± 28	6.6 ± 0.7	162.9 ± 7.3
[Cu(**C8**)]$^+$	MeCN	144 ± 26	6.4 ± 0.6	131.8 ± 6.2
	THF	164 ± 24	7.0 ± 0.8	166.6 ± 6.5
	CH_2Cl_2	194 ± 22	5.0 ± 0.5	154.2 ± 4.8
[Cu(**C9**)]$^+$	MeCN	185 ± 24	6.0 ± 0.6	129.1 ± 4.8
[Cu(**C10**)]$^+$	MeCN	151 ± 21	8.3 ± 0.8	119.4 ± 5.1
[Cu(**C11**)]$^+$	MeCN	143 ± 22	6.9 ± 0.9	121.8 ± 4.8
[Cu(**C12**)]$^+$	MeCN	135 ± 27	7.3 ± 0.9	124.6 ± 6.5
[Cu(**L4**)$_2$]$^+$	MeCN	137 ± 20	7.1 ± 0.9	138.6 ± 4.4
	THF	167 ± 22	8.8 ± 1.7	204.0 ± 6.5
	CH_2Cl_2	217 ± 25	6.6 ± 1.4	223.2 ± 6.4
[Cu(**L5**)$_2$]$^+$	MeCN	161 ± 24	7.4 ± 1.1	148.8 ± 5.3
[Cu(**L6**)$_2$]$^+$	MeCN	134 ± 24	6.7 ± 0.9	144.6 ± 5.1

6. *Electrochemical Studies*

Cyclic voltammograms of [Cu(**Cn**)]$^+$ (n = 7–12) in CH_3CN in the presence of 100 mM $TBAPF_6$ were collected using a CH Instruments 760E bipotentiostat with a custom-build glass 5 mL single compartment cell with three-electrode setup at 298 K. A glassy carbon (A = 0.07065 cm^2) was used as the working electrode, a platinum wire was used as the counter electrode, and saturated calomel electrode (sat' SCE) was used as the reference electrode. All electrochemical potentials were converted to the normal hydrogen electrode (NHE) according to published protocols.[18]

Prior to each experiment, the GC electrode was manually polished for 5 minutes using a polishing kit containing alumina suspension ranging from 3 to 0.5 µm (Allied Tech). The electrode was then thoroughly rinsed with deionized water and dried using a stream of N_2. All solutions were purged with N_2 for 20 minutes to remove any dissolved oxygen.

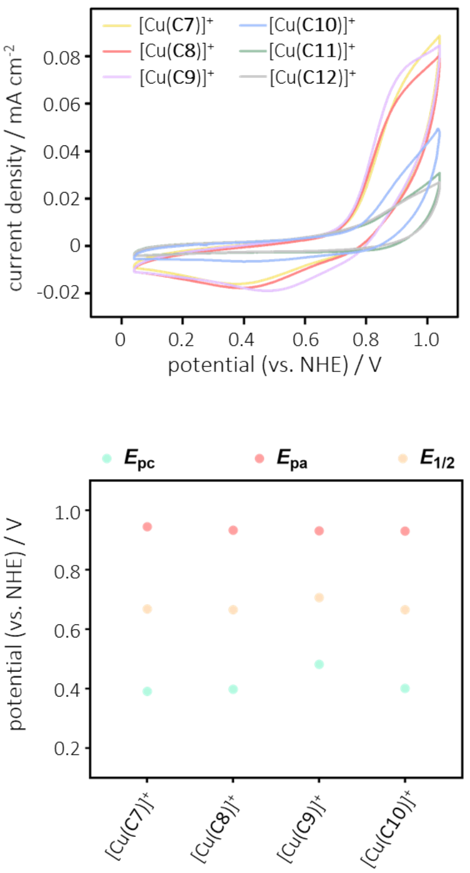

Figure S50. (a) Cyclic voltammograms of the Cu(I) catenane complexes in N_2-saturated with 100 mM $(Bu^n)_4PF_6$ in MeCN; and (b) E_{pc} (green), $E_{1/2}$ (orange), and E_{pa} (red) of [Cu(**Cn**)]$^+$ (n = 7–10).

7. Competitive Cyanide Exchange

Thermodynamic stabilities of Cu(I) catenane complexes

Thermodynamic stabilities of Cu(I) catenane complexes were studied by competitive cyanide exchange experiments. Stock solutions of $[Cu(\mathbf{Cn})]^+$ (n = 7–12), $[Cu(\mathbf{C8\text{-}Ms})]^+$, $[Cu(\mathbf{C8\text{-}Ipps})]^+$, $[Cu(\mathbf{C8\text{-}Tipps})]^+$, $[Cu(\mathbf{C8\text{-}Fmoc})]^+$ at 1 mM, and that of NaCN at 5.00 mM, 3.75 mM, 2.50 mM were prepared in 9:1 (v/v) $MeCN/H_2O$. For each measurement, a 1 mL solution of the Cu(I) [2]catenane at 50 μM was obtained by diluting the stock solution using the same solvent mixture, and 10 μL of the 5.00 mM, 3.75 mM or 2.50 mM NaCN stock solutions of NaCN, corresponding to 1, 0.75, and 0.5 equiv. respectively, was added using a Thermo Scientific™ pipette. Blank control reaction was obtained by adding 10 μL of Millipore water to replace the cyanide stock solution. Samples for absorption measurement were transferred to a quartz cuvette with a path length of 1 cm and a cell volume of 1.5 mL. UV-Vis spectra were collected after the reaction mixtures have been equilibrated at room temperature for 6 and 24 hours. No significant difference was observed in the spectra collected at 6 and 24 hours, suggesting that equilibrium has been reached.

Equilibrium concentrations of the Cu(I) catenane remained in the sample were calculated from the intensity of the Cu(I)-to-phenanthroline MLCT absorption at *ca.* 450 nm to obtain the apparent equilibrium constant K_{CN}. Stability constant β of the Cu(I) catenane complexes were calculated by Eq. S1 using a reported solubility product of CuCN ($\log K_{sp} = -20$ at 298 K).[19,20] Each measurement was repeated thrice, and β is reported as an averaged value from three measurements.

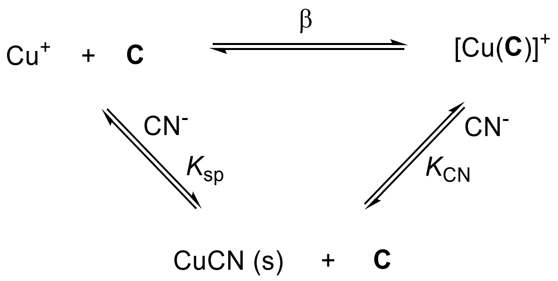

$$\beta = 1/K_{sp}K_{CN} \qquad \text{Eq. S1}$$

Enthalpy and entropy changes of Cu(I) catenane coordination

The stability constant β of the Cu(I) catenane complexes were obtained at 298 K, 313 K, 323 K, 333 K using an Agilent Cary 8454 spectrophotometer equipped with UNISOKI CoolSpeK cryostat, and solubility product of CuCN at the corresponding temperature was obtained from literature.[21] Enthalpy and entropy changes of the coordination of Cu^+ to the catenanes were obtained by van 't Hoff analysis.

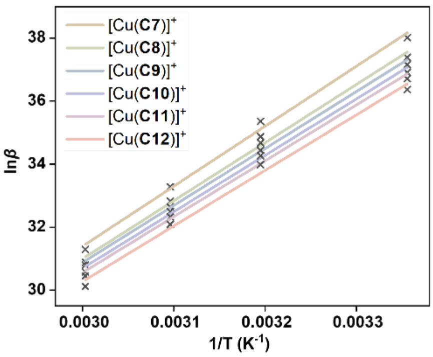

	ΔH (kJ/mol)	ΔS (J/mol/K)	linear-fitted relationships
[Cu(**C7**)]$^+$	-158.3	-213.9	y = 19041x – 25.7 (R^2 = 0.9932)
[Cu(**C8**)]$^+$	-153.2	-202.1	y = 18434x – 24.3 (R^2 = 0.9910)
[Cu(**C9**)]$^+$	-150.3	-194.1	y = 18074x – 23.3 (R^2 = 0.9919)
[Cu(**C10**)]$^+$	-148.9	-191.6	y = 17912x – 23.0 (R^2 = 0.9913)
[Cu(**C11**)]$^+$	-148.3	-188.2	y = 17729x – 22.6 (R^2 = 0.9918)
[Cu(**C12**)]$^+$	-146.5	-187.8	y = 17623x – 22.6 (R^2 = 0.9884)

Figure S51. The van 't Hoff plots and the obtained enthalpy and entropy changes.

Kinetic investigations of cyanide exchange

The competitively cyanide exchange was monitored using a fast-mixing technique using an Applied Photophysics SX20 stopped-flow spectrometer with UV-Vis PMT detector to obtain the kinetic parameters. The rate of ligand exchange was determined by monitoring the disappearance of the MLCT band at *ca.* 450 nm of a 50 μM solution of the Cu(I) complexes (9:1 (v/v) MeCN/H_2O) in the presence of 5, 10, 15, 25, 50 or 100 equiv. of NaCN. The observed demetallation rate constants (k_{obs}) were plotted as a function of the cyanide concentrations according to Eq. S2 to obtain the rate constant for the self-dissociation (k_{sol}) and cyanide-assisted demetallation (k_{CN}).[22]

$$k_{\text{obs}} = k_{\text{sol}} + k_{\text{CN}}[\text{CN}^-] \qquad \text{Eq. S2}$$

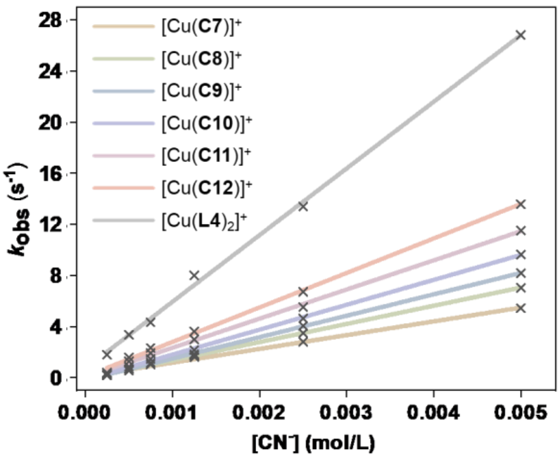

complex	linear-fitted relationships
$[Cu(\mathbf{C7})]^+$	$y = 1077x + 0.082$ ($R^2 = 0.9944$)
$[Cu(\mathbf{C8})]^+$	$y = 1424x - 0.079$ ($R^2 = 0.9997$)
$[Cu(\mathbf{C9})]^+$	$y = 1676x - 0.195$ ($R^2 = 0.9995$)
$[Cu(\mathbf{C10})]^+$	$y = 1950x - 0.160$ ($R^2 = 0.9989$)
$[Cu(\mathbf{C11})]^+$	$y = 2283x + 0.037$ ($R^2 = 0.9978$)
$[Cu(\mathbf{C12})]^+$	$y = 2700x + 0.078$ ($R^2 = 0.9972$)
$[Cu(\mathbf{L4})_2]^+$	$y = 5220x + 0.698$ ($R^2 = 0.9976$)

Figure S52. Kinetic data and the linear-fitting of cyanide-assisted demetallation of the Cu(I) complexes.

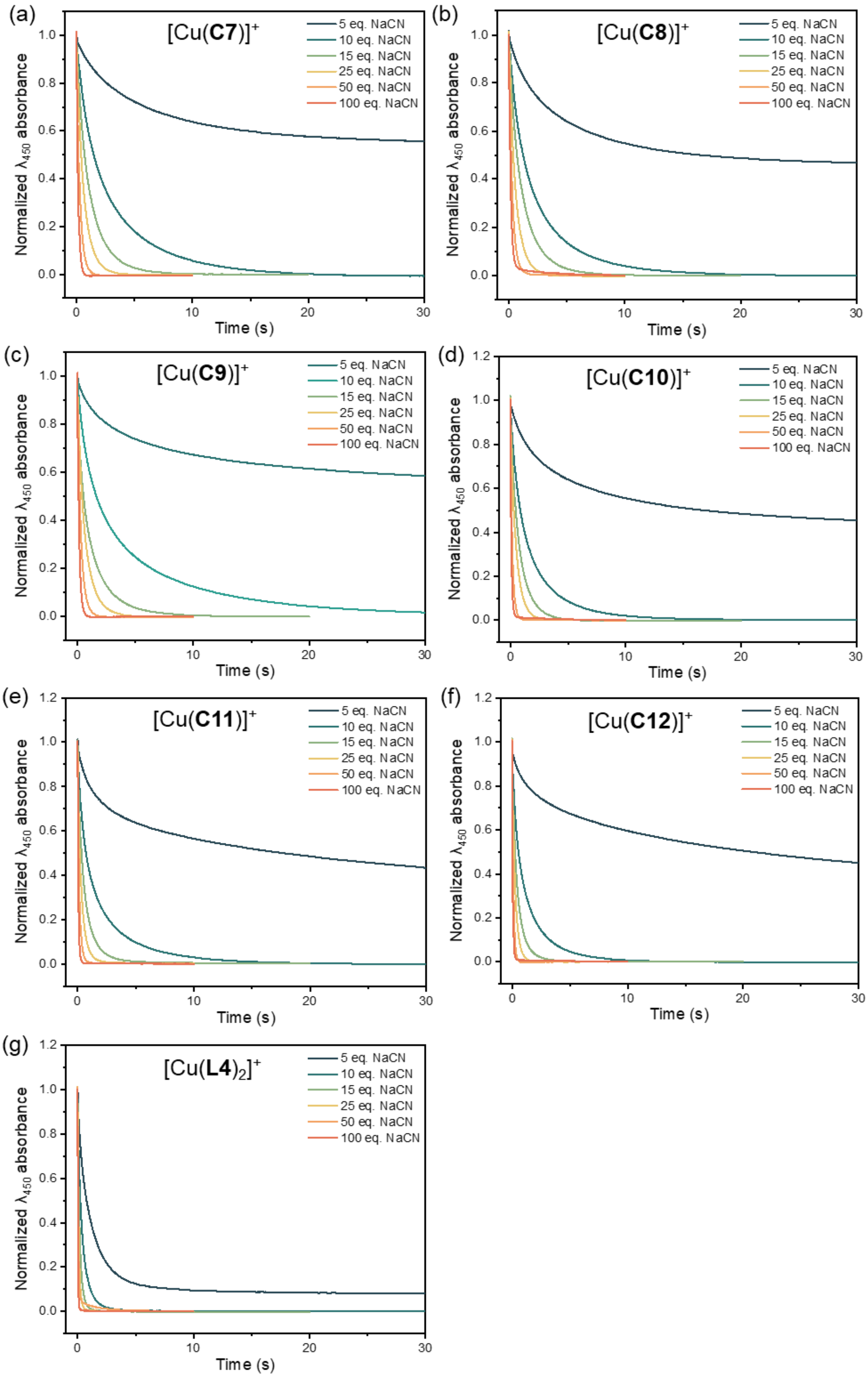

Figure S53. Demetallation kinetics of 50 μM of (a) [Cu(**C7**)]$^+$; (b) [Cu(**C8**)]$^+$; (c) [Cu(**C9**)]$^+$; (d) [Cu(**C10**)]$^+$; (e) [Cu(**C11**)]$^+$; (f) [Cu(**C12**)]$^+$; and (g) [Cu(**L4**)$_2$]$^+$ in 9:1 (v/v) MeCN/H_2O monitored by absorbance at 450 nm.

8. Catenane Scrambling

Scrambling of Cu^+ ion between catenanes were studied using ^1H NMR spectroscopy. Stock solutions of [Cu(**Cn**)]$^+$ (n = 7–12), [Cu(**C8-Ms**)]$^+$, [Cu(**C8-Ipps**)]$^+$, [Cu(**C8-Tipps**)]$^+$, [Cu(**C8-Fmoc**)]$^+$, as well as the metal-free [2]catenane ligands **Cn** (n = 8, 10, 12), **C8-Ms**, **C8-Tipps** at 4 mM were prepared in 1:1 (v/v) $CD_3CN/CDCl_3$. Samples of a 1:1 (mol/mol) mixture of [Cu(**C7**)]$^+$/**C10**, [Cu(**C8**)]$^+$/**C10**, [Cu(**C9**)]$^+$/**C12**, [Cu(**C10**)]$^+$/**C8**, [Cu(**C11**)]$^+$/**C8**, [Cu(**C12**)]$^+$/**C10**, [Cu(**C10**)]$^+$/**C8-Ms**, [Cu(**C10**)]$^+$/**C8-Tipps**, [Cu(**C8-Ms**)]$^+$/**C10**, [Cu(**C8-Ipps**)]$^+$/**C10**, [Cu(**C8-Tipps**)]$^+$/**C10** and [Cu(**C8-Fmoc**)]$^+$/**C10** were prepared by mixing 125 μL each of the Cu(I)-[2]catenane complex and the metal-free [2]catenane ligand stock solutions, followed by addition of 250 μL of 1:1 (v/v) $CD_3CN/CDCl_3$. Samples of a 1:2 (mol/mol) mixture were prepared by mixing 125 μL of the Cu(I)-[2]catenane complex stock solution, 250 μL of the metal-free [2]catenane ligand stock solution, and followed by addition of 125 μL of 1:1 (v/v) $CD_3CN/CDCl_3$. Changes in the concentration of the catenane complexes and free catenanes were monitored by ^1H NMR, and the first ^1H spectrum can be obtained within 2–5 min after the mixing. Each spectrum was obtained after 16 scans, and a spectrum was acquired every 1 min for the first 30 min, and then every 5 to 30 minutes for a total of 10 hours. Concentrations of the Cu(I) catenane complexes were calculated from the integration ratio of the phenyl (H_{Ar}) signals, and the data were plot against time. Non-linear fittings were performed using Origin 2022.

To extract the kinetic data, the time-dependent decrease of the concentration of the catenane complex was fitted to a kinetic model of guest exchange between two macrocyclic hosts as described by Biedermann.[23] In Biedermann's model, the rate of exchange of a guest (i.e. G) between two hosts (i.e. H1 and H2) was monitored, and the bound guest will have to first dissociate from the original host (e.g. H1) before binding to the other host (e.g. H2). For the much higher initial concentration of the free H2 and most H1 is bound as [H1•G] complex, the initial association rate of [H2•G] will be much faster than the initial association rate of [H1•G]. Concentration of [H1•G] was monitored over time, and kinetics of guest exchange can be described by Eq. S3:

$$C = Ae^{-kt} + B \qquad \text{Eq. S3}$$

A = total change in the concentration of [H1•G] after equilibrium

B = equilibrium concentration of [H1•G]

C = concentration of [H1•G] at time *t*

k = rate constant of the dissociation of G from [H1•G]

In our study, the 4-coordinate Cu(I) catenane complexes are coordinatively saturated, and the direct coordination of a free catenane to the 4-coordinate Cu^{+} in the other catenane will be highly improbable. Hence, at least one of the two phenanthrolines in the Cu(I) catenane complexes will have to first dissociate from the Cu^{+} to reduce its coordination number, before the metal can be exchanged to another catenane. As such, changes in the concentration of the Cu(I) catenane complexes in the scrambling experiment can also be described by Eq. S3, in which k is the rate constant for the process that the Cu^{+} ion in the catenane complex made available for exchange (i.e. k_{scrm}).

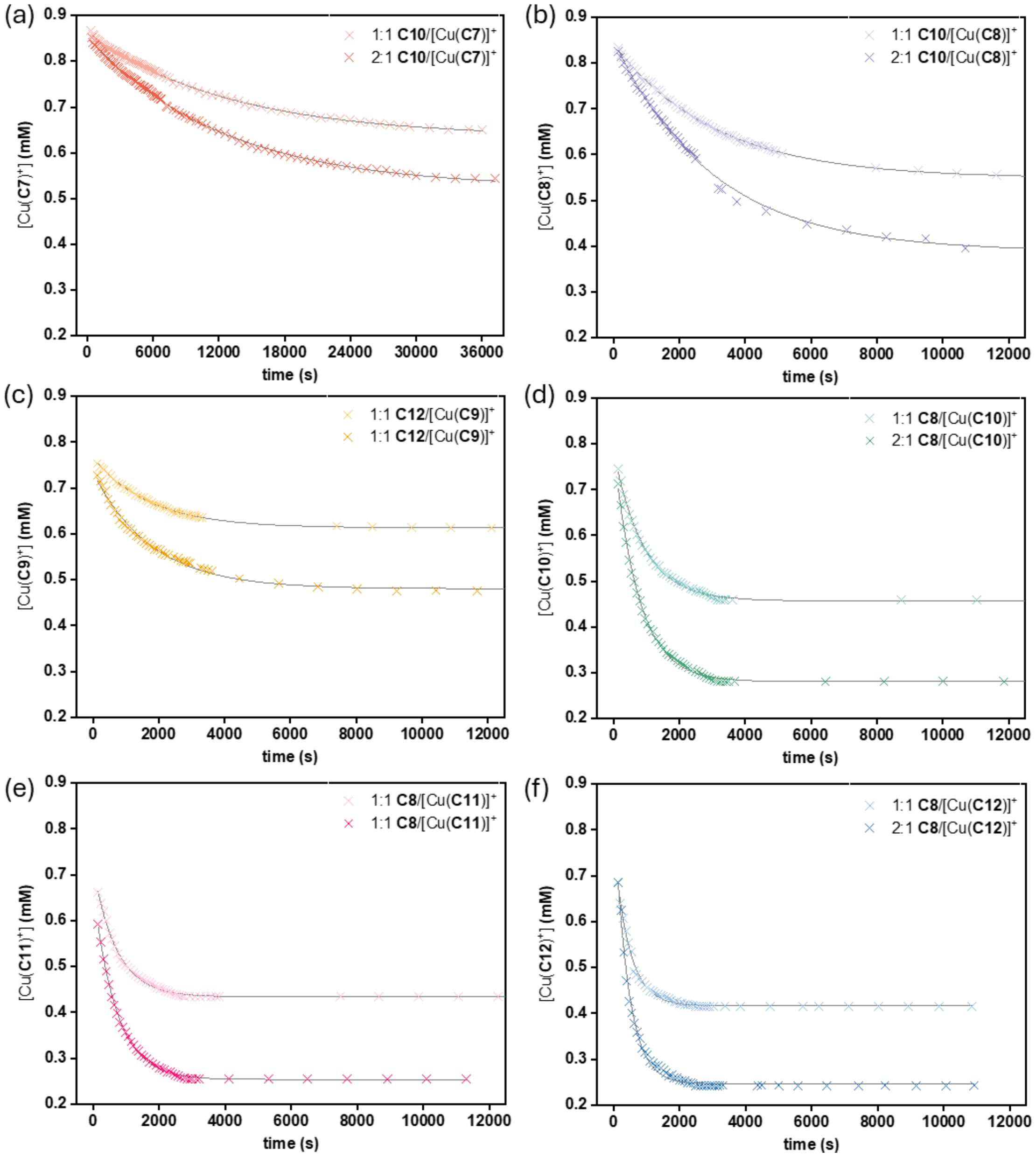

Figure S54. Time-dependent changes of the concentration and the non-linear curve fitting of the scrambling experiment with (a) [Cu(**C7**)]$^{+}$; (b) [Cu(**C8**)]$^{+}$; (c) [Cu(**C9**)]$^{+}$; (d) [Cu(**C10**)]$^{+}$; (e) [Cu(**C11**)]$^{+}$; and (f) [Cu(**C12**)]$^{+}$ as the starting Cu(I) catenane complex.

Table S23. Data of scrambling experiments.

initial conc. (mM)		equilibrium conc. (mM)				K_{ex}*	$y = Ae^{-kx} + B$			
[Cu(**C**)]+	**C′**	[Cu(**C**)]+	**C′**	[Cu(**C′**)]+	**C**		$k \times 10^{-3}$	A	B	R^2
[Cu(**C7**)]+	**C10**									
1	1	0.63	0.63	0.37	0.37	0.3	0.0778	0.228	0.635	0.9989
1	2	0.53	1.53	0.47	0.47	0.3	0.0825	0.332	0.523	0.9991
[Cu(**C8**)]+	**C10**									
1	1	0.55	0.55	0.45	0.45	0.7	0.325	0.294	0.548	0.9991
1	2	0.39	1.39	0.61	0.61	0.7	0.334	0.462	0.388	0.9971
[Cu(**C9**)]+	**C12**									
1	1	0.61	0.61	0.39	0.39	0.4	0.577	0.153	0.613	0.9991
1	2	0.48	1.48	0.52	0.52	0.4	0.562	0.255	0.480	0.9927
[Cu(**C10**)]+	**C8**									
1	1	0.46	0.46	0.54	0.54	1.4	1.11	0.329	0.457	0.9979
1	2	0.28	1.28	0.72	0.72	1.4	1.32	0.511	0.280	0.9982
[Cu(**C11**)]+	**C8**									
1	1	0.43	0.43	0.57	0.57	1.8	1.41	0.281	0.434	0.9965
1	2	0.26	1.26	0.74	0.74	1.7	1.43	0.412	0.253	0.9987
[Cu(**C12**)]+	**C8**									
1	1	0.42	0.42	0.58	0.58	1.9	2.16	0.350	0.417	0.9949
1	2	0.24	1.24	0.76	0.76	1.9	2.37	0.603	0.246	0.9931

*K_{ex} is the equilibrium constant of the catenane scrambling. For each scrambling experiment, the K_{ex} value is consistent with the ratio of the thermodynamic stability constants of the two involved Cu(I) [2]catenane complexes (i.e. $\beta_{C'}/\beta_{C}$).

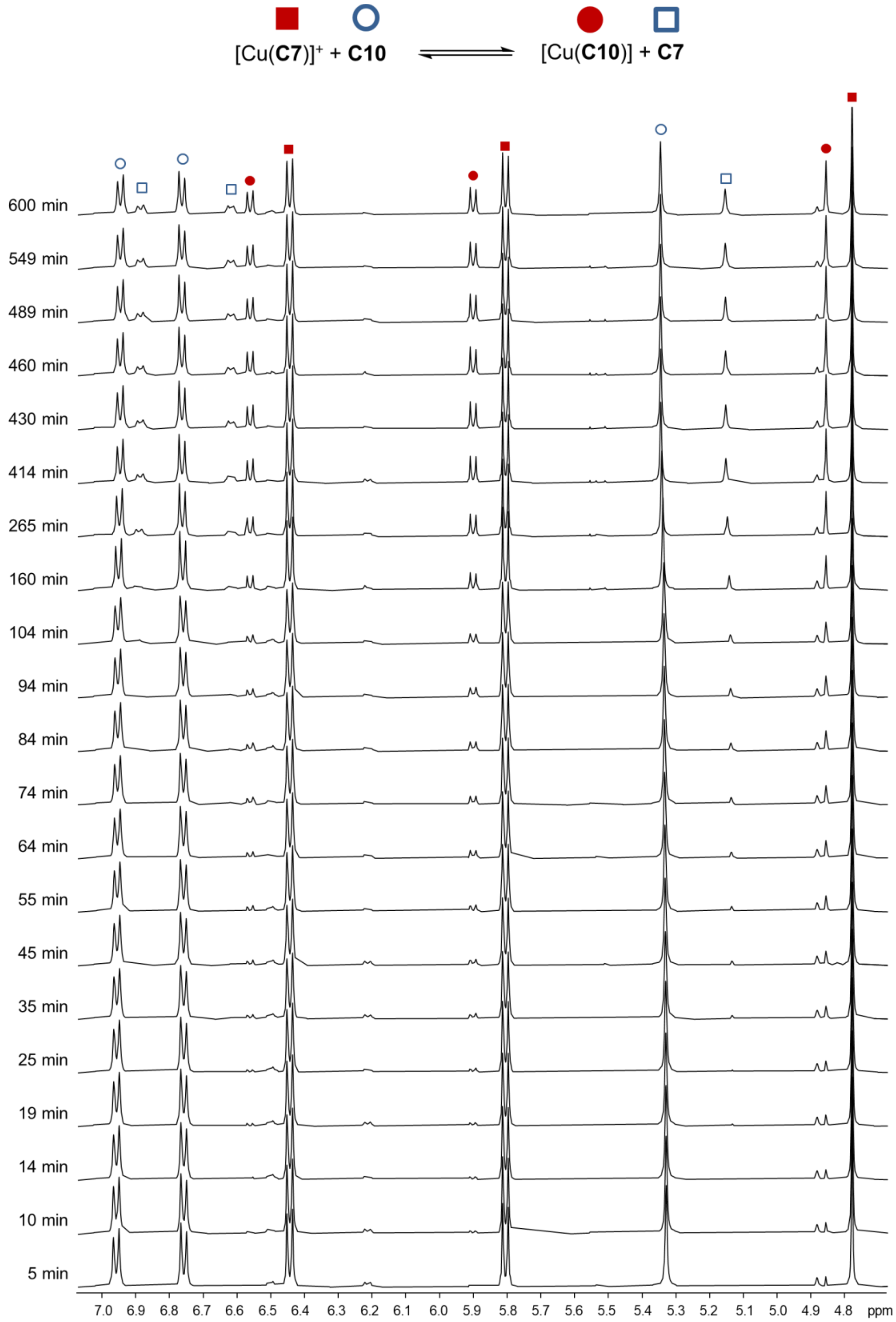

Figure S55. Partial ^{1}H NMR (500 MHz, v/v = 1:1 $CD_3CN/CDCl_3$, 298 K) time-dependent monitoring on ligand scrambling starting from 1:1 (mol/mol) [Cu(**C7**)]$^{+}$/**C10**.

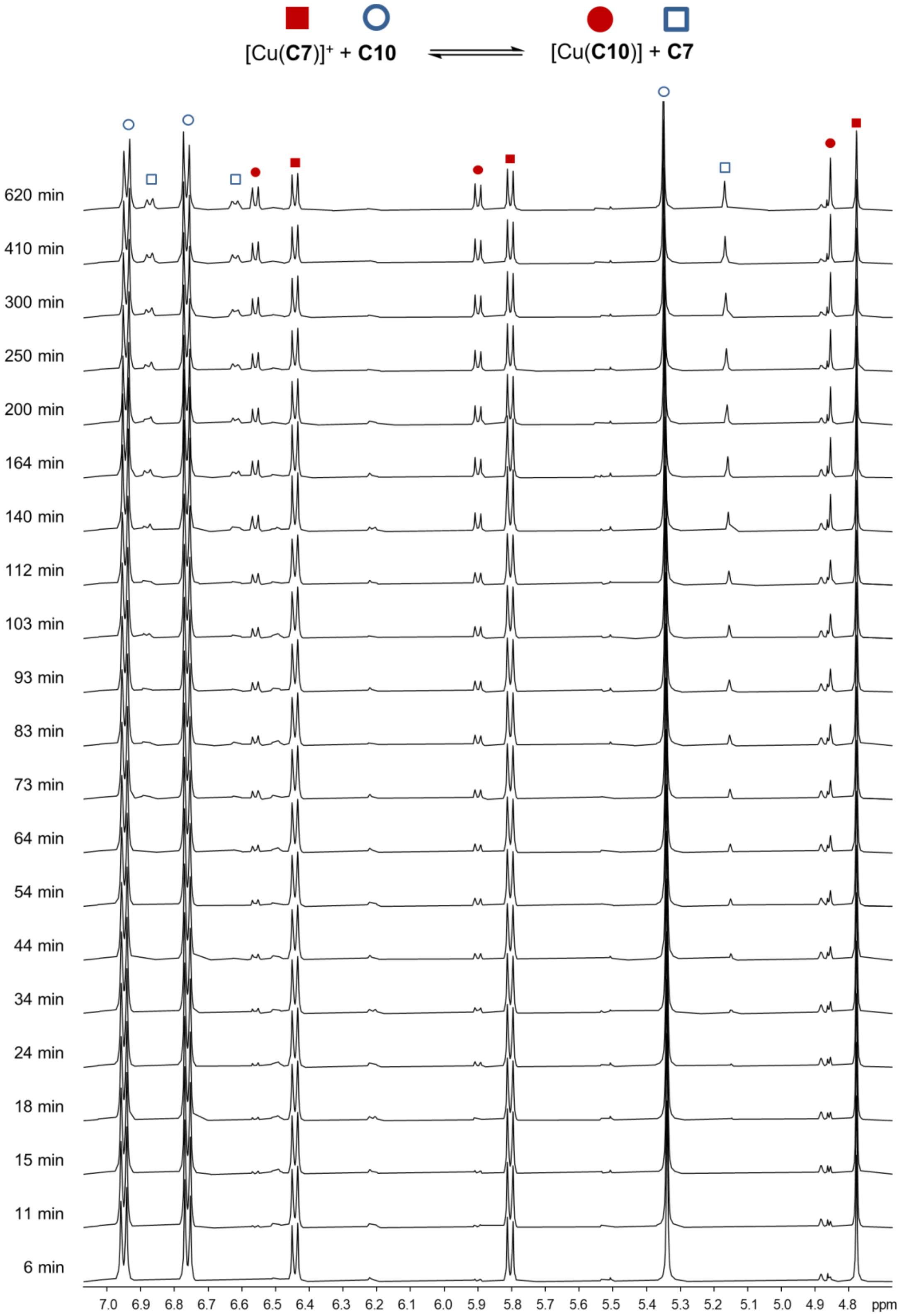

Figure S56. Partial ^{1}H NMR (500 MHz, v/v = 1:1 $CD_3CN/CDCl_3$, 298 K) time-dependent monitoring on ligand scrambling starting from 1:2 (mol/mol) [Cu(**C7**)]$^{+}$/**C10**.

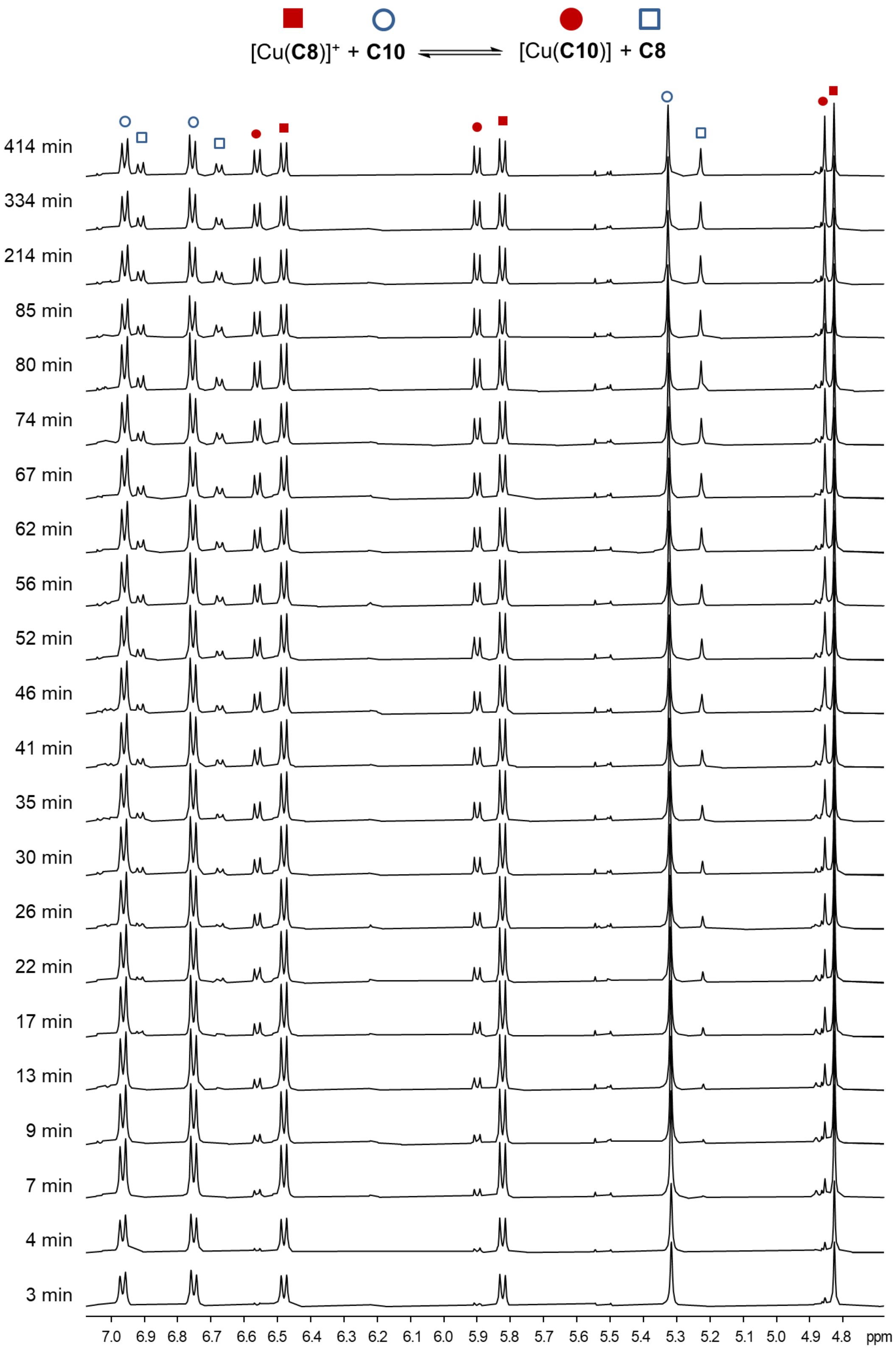

Figure S57. Partial ^{1}H NMR (500 MHz, v/v = 1:1 $CD_3CN/CDCl_3$, 298 K) time-dependent monitoring on ligand scrambling starting from 1:1 (mol/mol) [Cu(**C8**)]$^+$/**C10**.

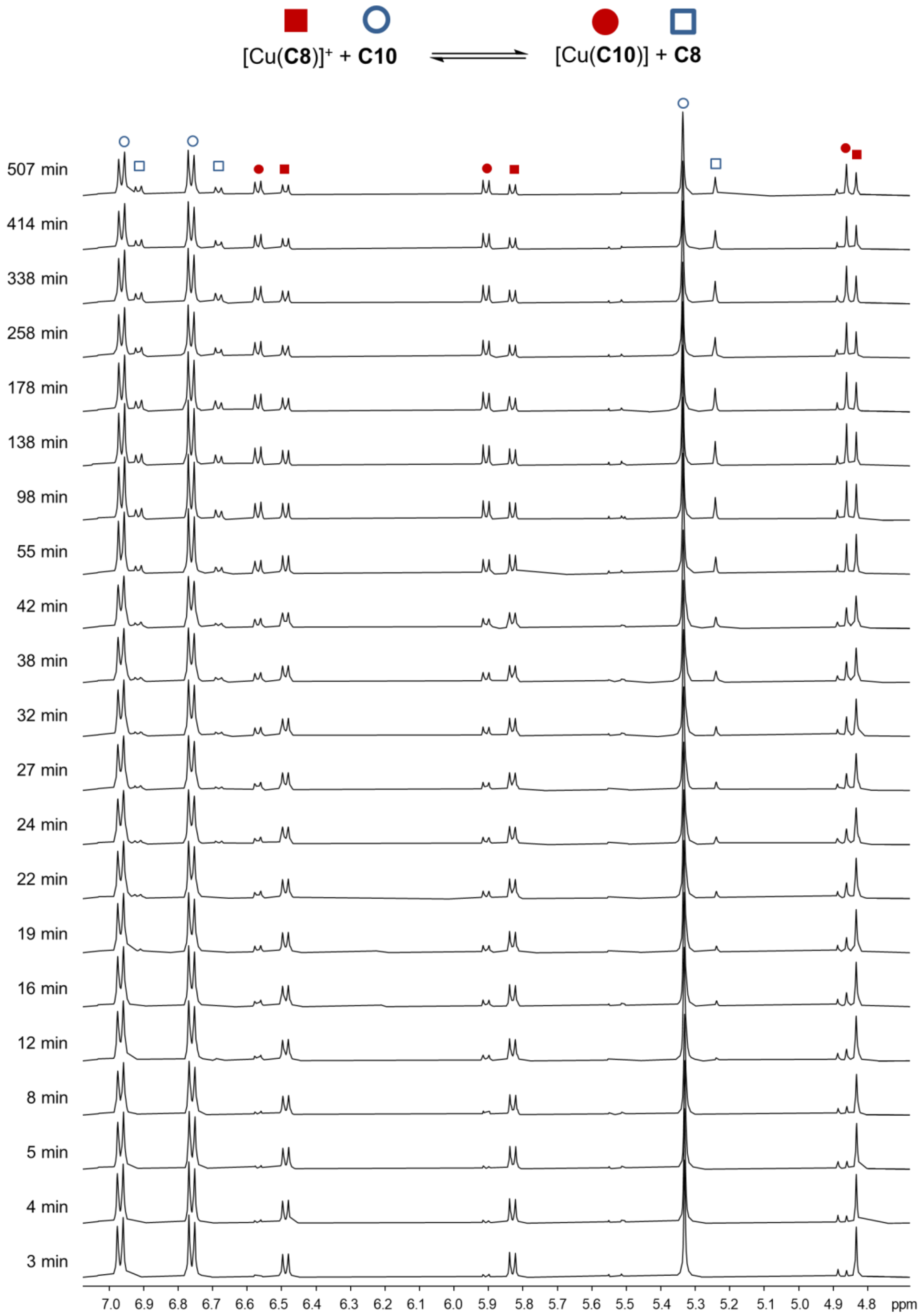

Figure S58. Partial ^{1}H NMR (500 MHz, v/v = 1:1 $CD_3CN/CDCl_3$, 298 K) time-dependent monitoring on ligand scrambling starting from 1:2 (mol/mol) [Cu(**C8**)]$^+$/**C10**.

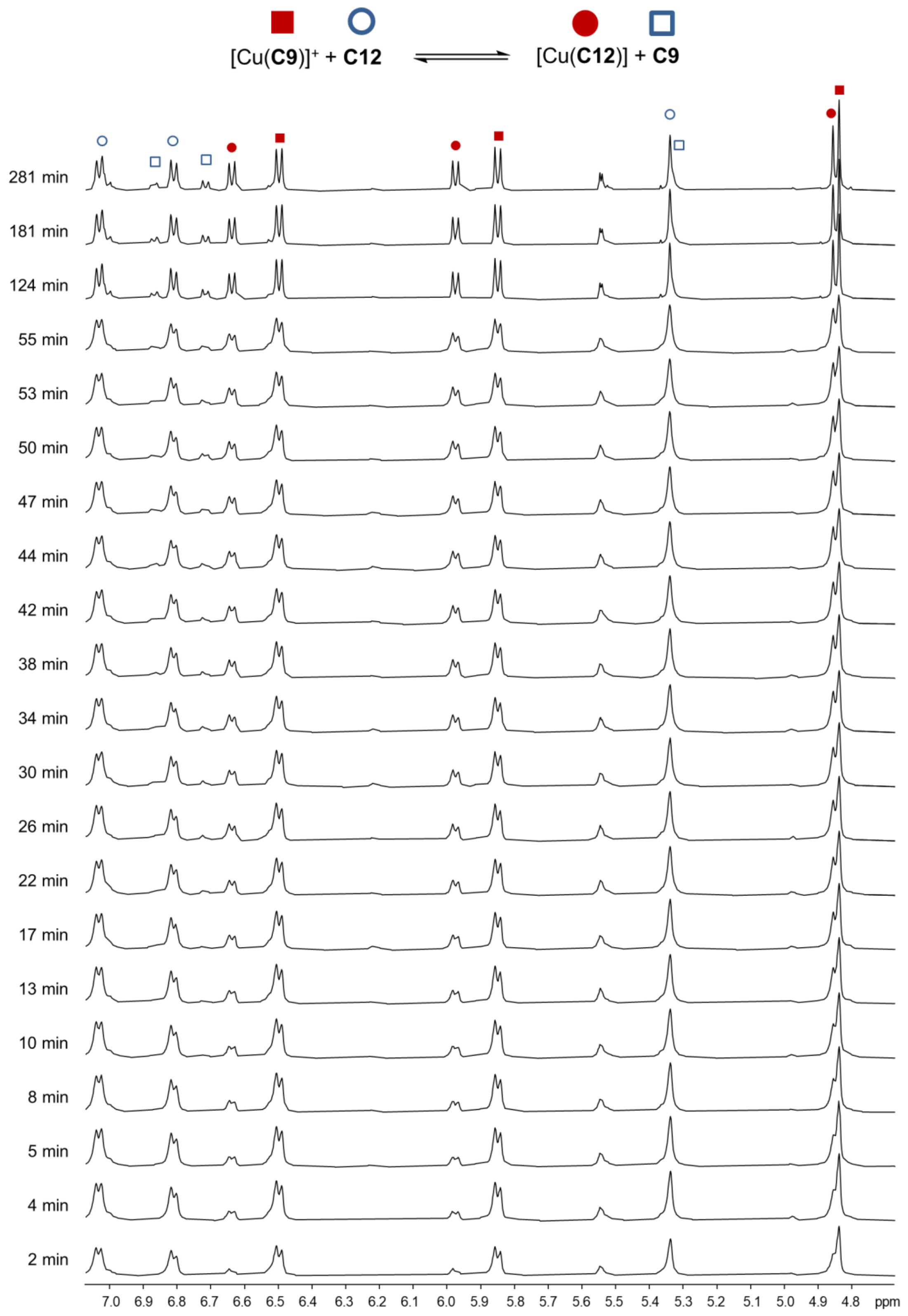

Figure S59. Partial ^{1}H NMR (500 MHz, v/v = 1:1 $CD_3CN/CDCl_3$, 298 K) time-dependent monitoring on ligand scrambling starting from 1:1 (mol/mol) $[Cu(\mathbf{C9})]^+$/**C12**.

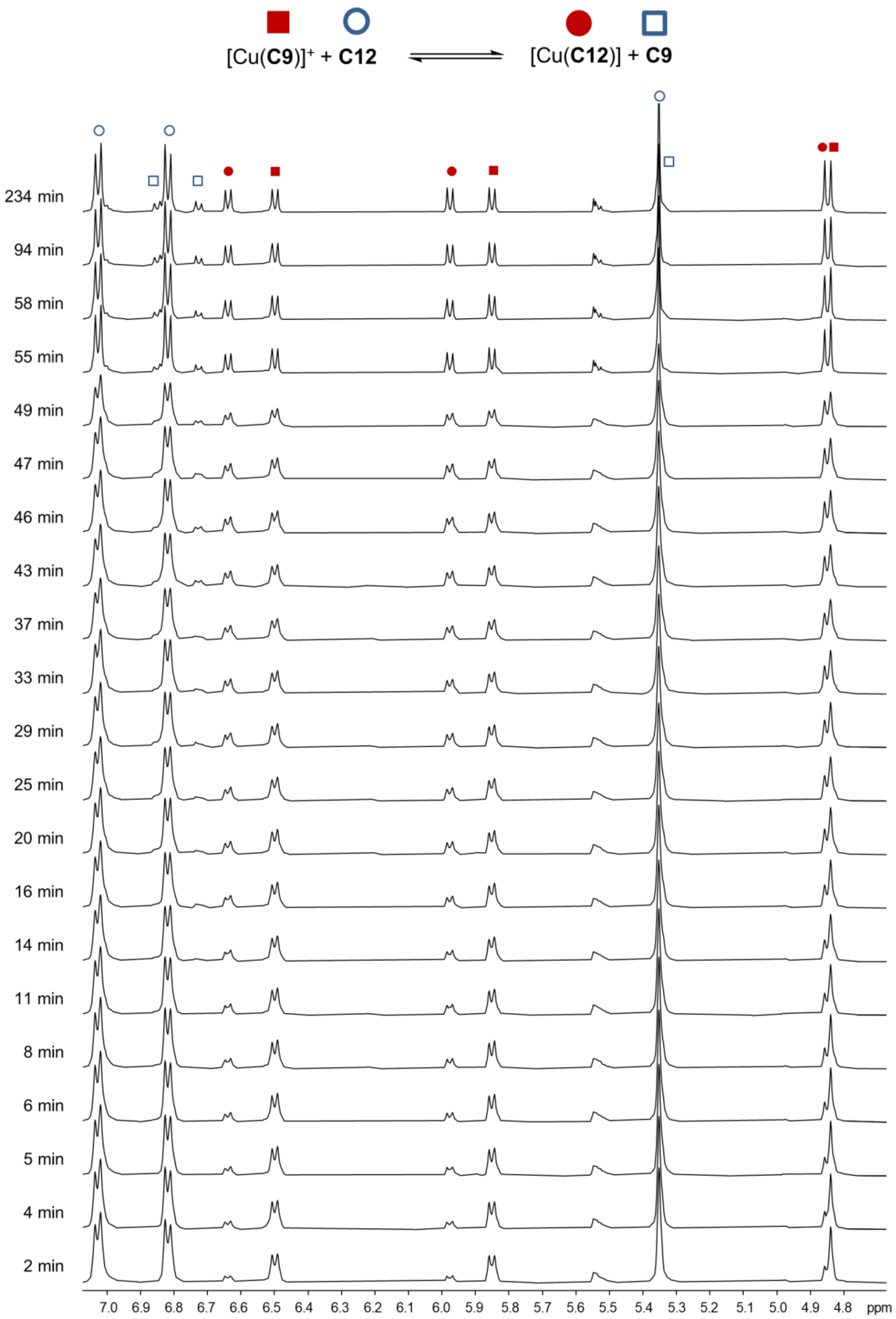

Figure S60. Partial ^{1}H NMR (500 MHz, v/v = 1:1 $CD_3CN/CDCl_3$, 298 K) time-dependent monitoring on ligand scrambling starting from 1:2 (mol/mol) [Cu(**C9**)]$^+$/**C12**.

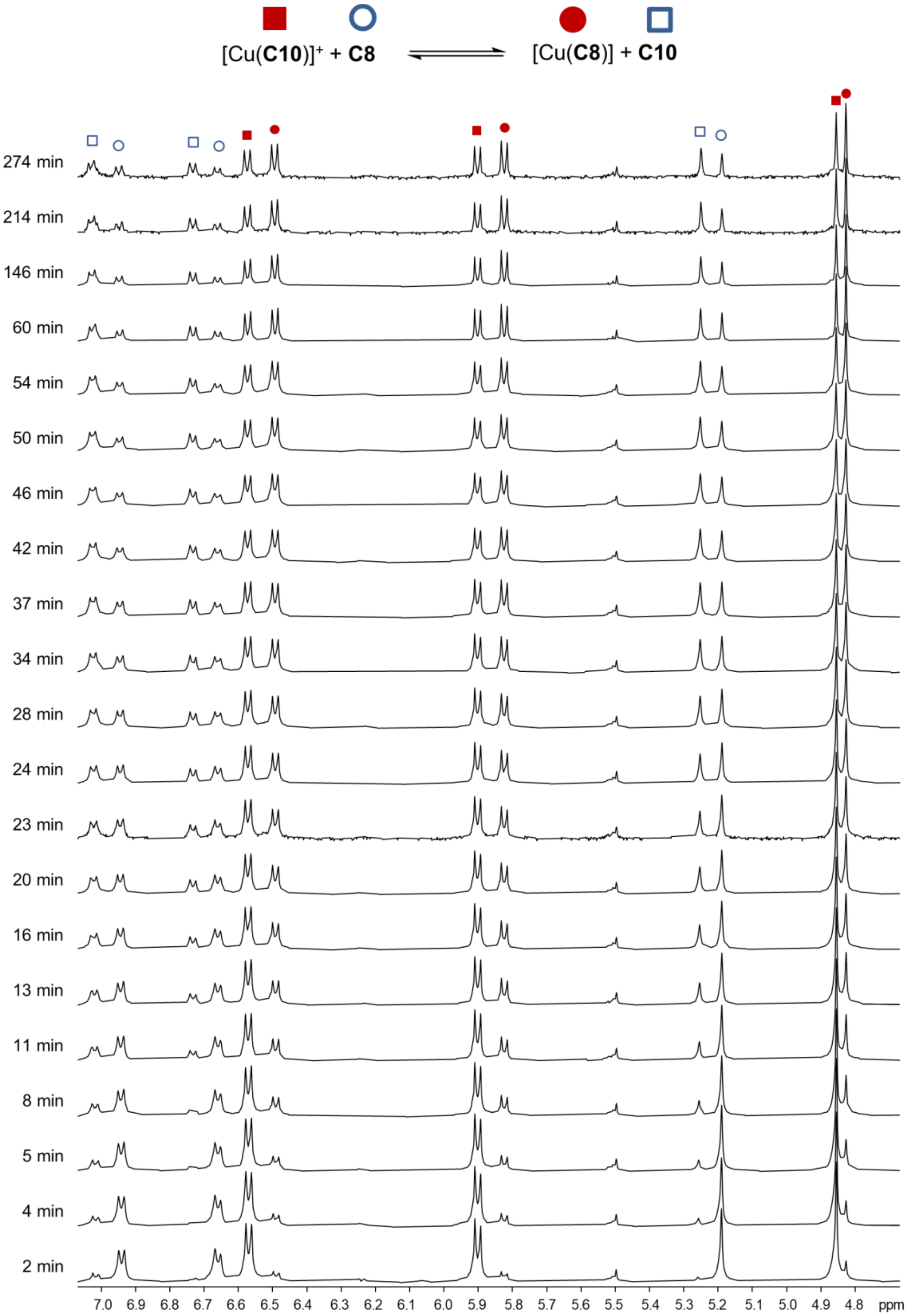

Figure S61. Partial ^1H NMR (500 MHz, v/v = 1:1 $CD_3CN/CDCl_3$, 298 K) time-dependent monitoring on ligand scrambling starting from 1:1 (mol/mol) [Cu(**C10**)]$^+$/**C8**.

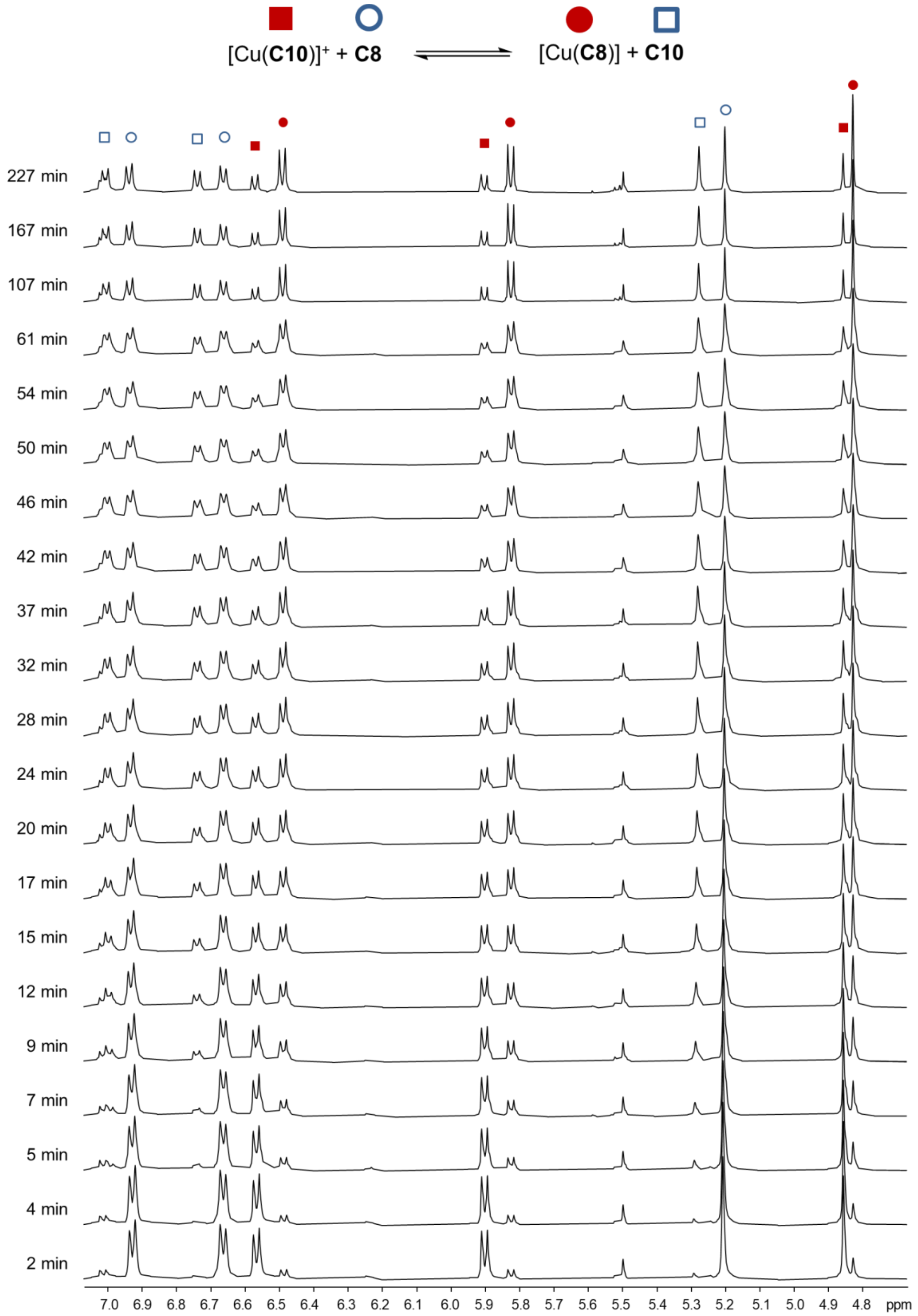

Figure S62. Partial ^{1}H NMR (500 MHz, v/v = 1:1 $CD_3CN/CDCl_3$, 298 K) time-dependent monitoring on ligand scrambling starting from 1:2 (mol/mol) $[Cu(\mathbf{C10})]^+$/**C8**.

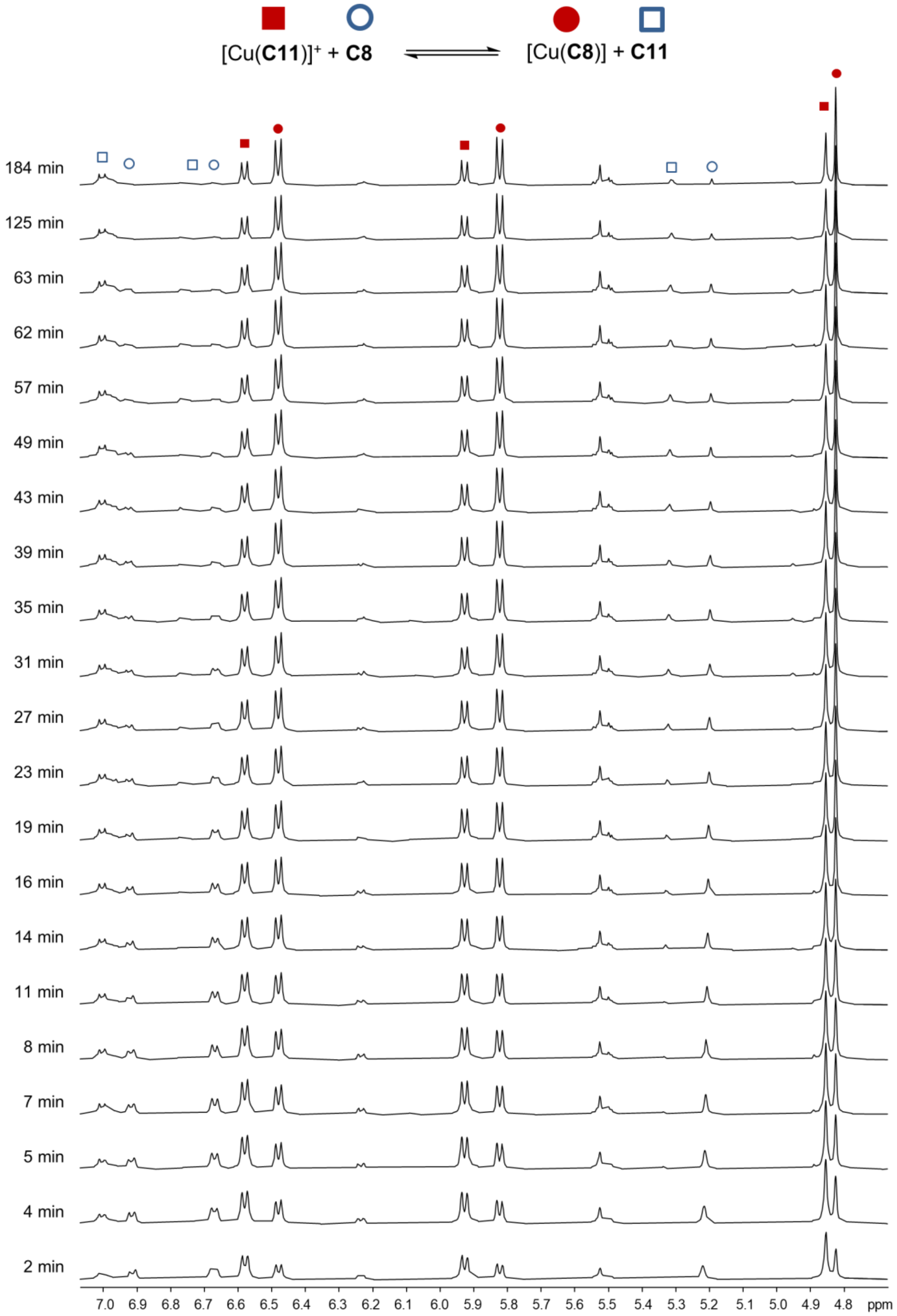

Figure S63. Partial ^{1}H NMR (500 MHz, v/v = 1:1 $CD_3CN/CDCl_3$, 298 K) time-dependent monitoring on ligand scrambling starting from 1:1 (mol/mol) [Cu(**C11**)]$^{+}$/**C8**.

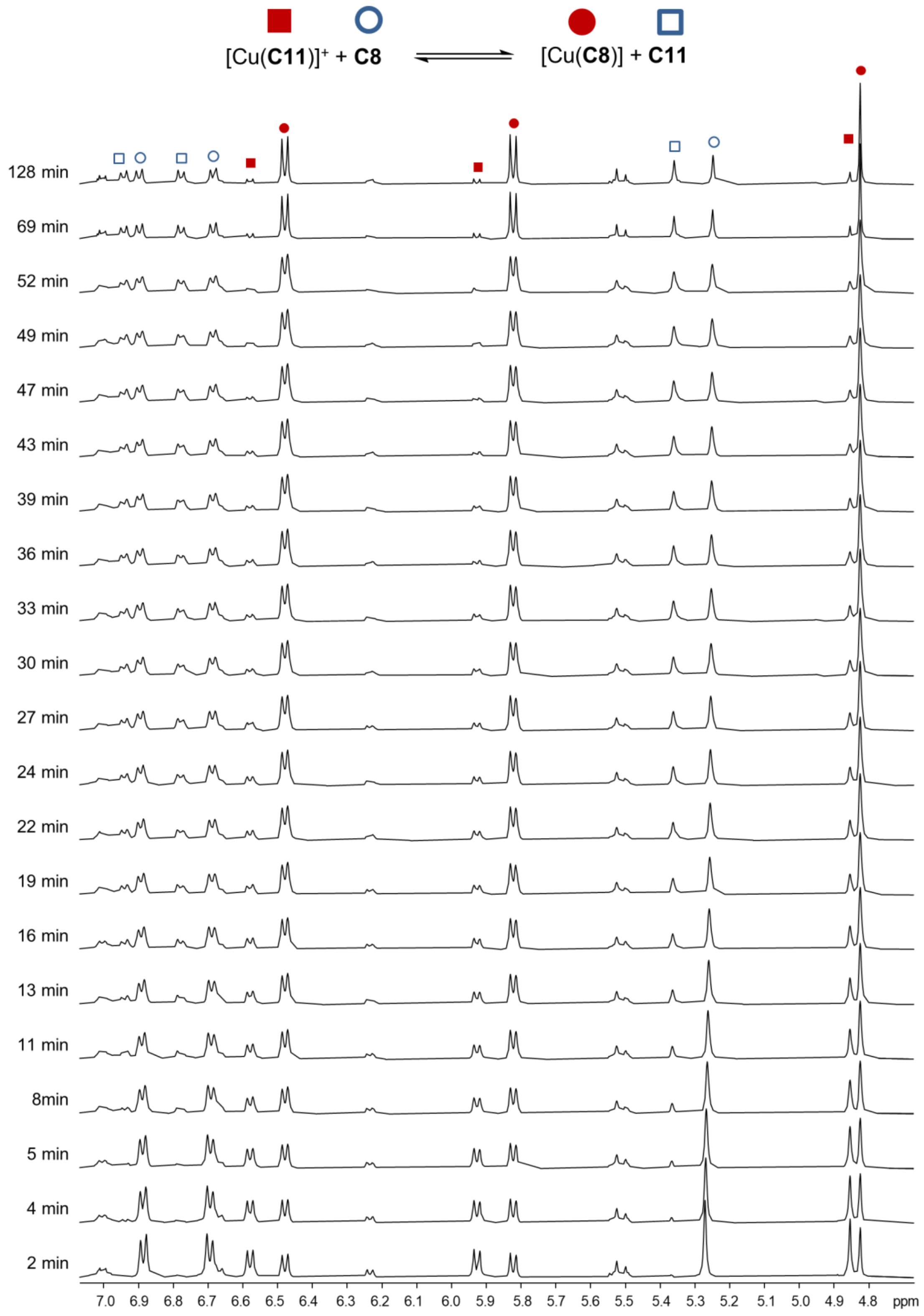

Figure S64. Partial ^{1}H NMR (500 MHz, v/v = 1:1 $CD_3CN/CDCl_3$, 298 K) time-dependent monitoring on ligand scrambling starting from 1:2 (mol/mol) [Cu(**C11**)]$^+$/**C8**.

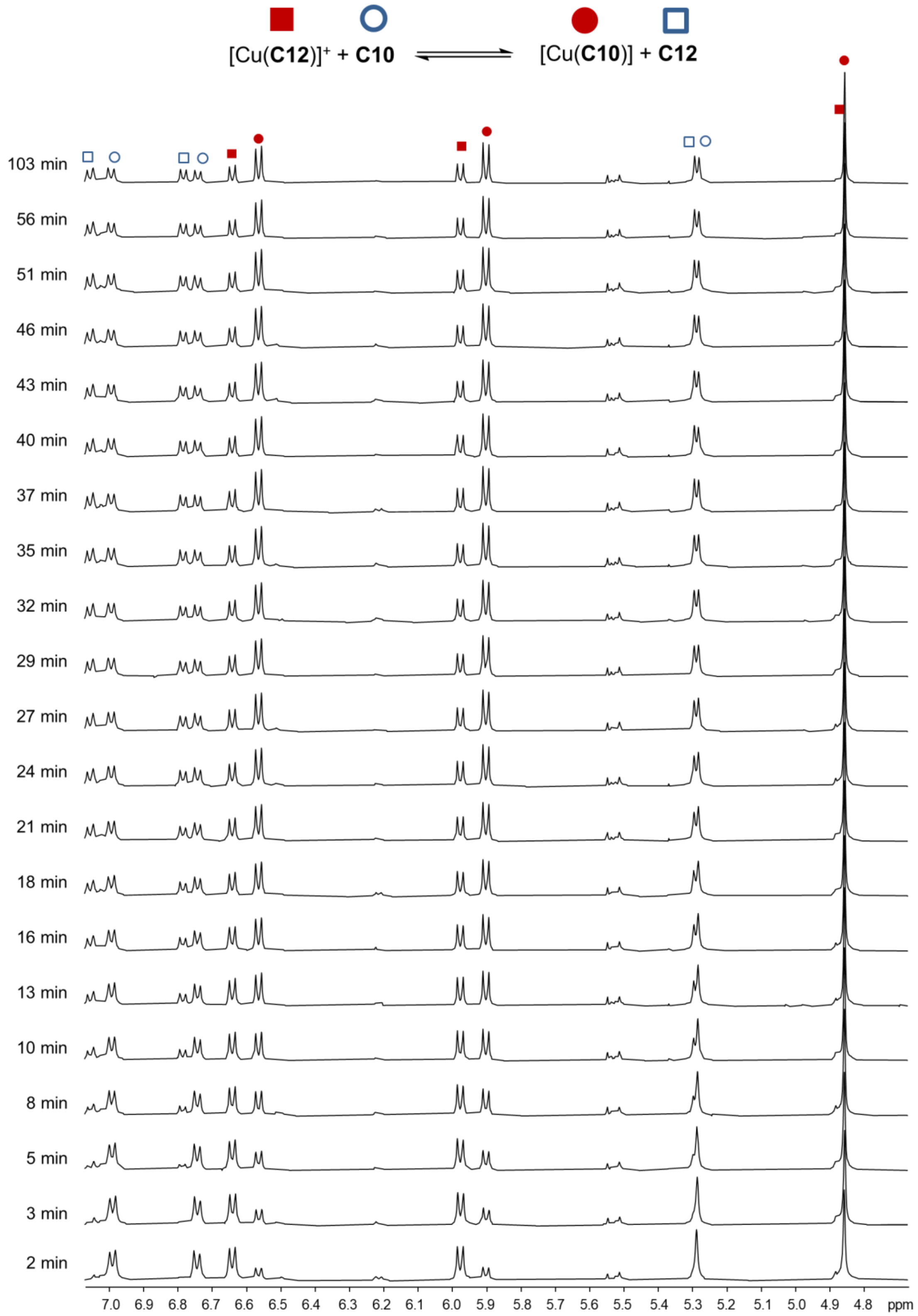

Figure S65. Partial ^{1}H NMR (500 MHz, v/v = 1:1 $CD_3CN/CDCl_3$, 298 K) time-dependent monitoring on ligand scrambling starting from 1:1 (mol/mol) [Cu(**C12**)]$^+$/**C10**.

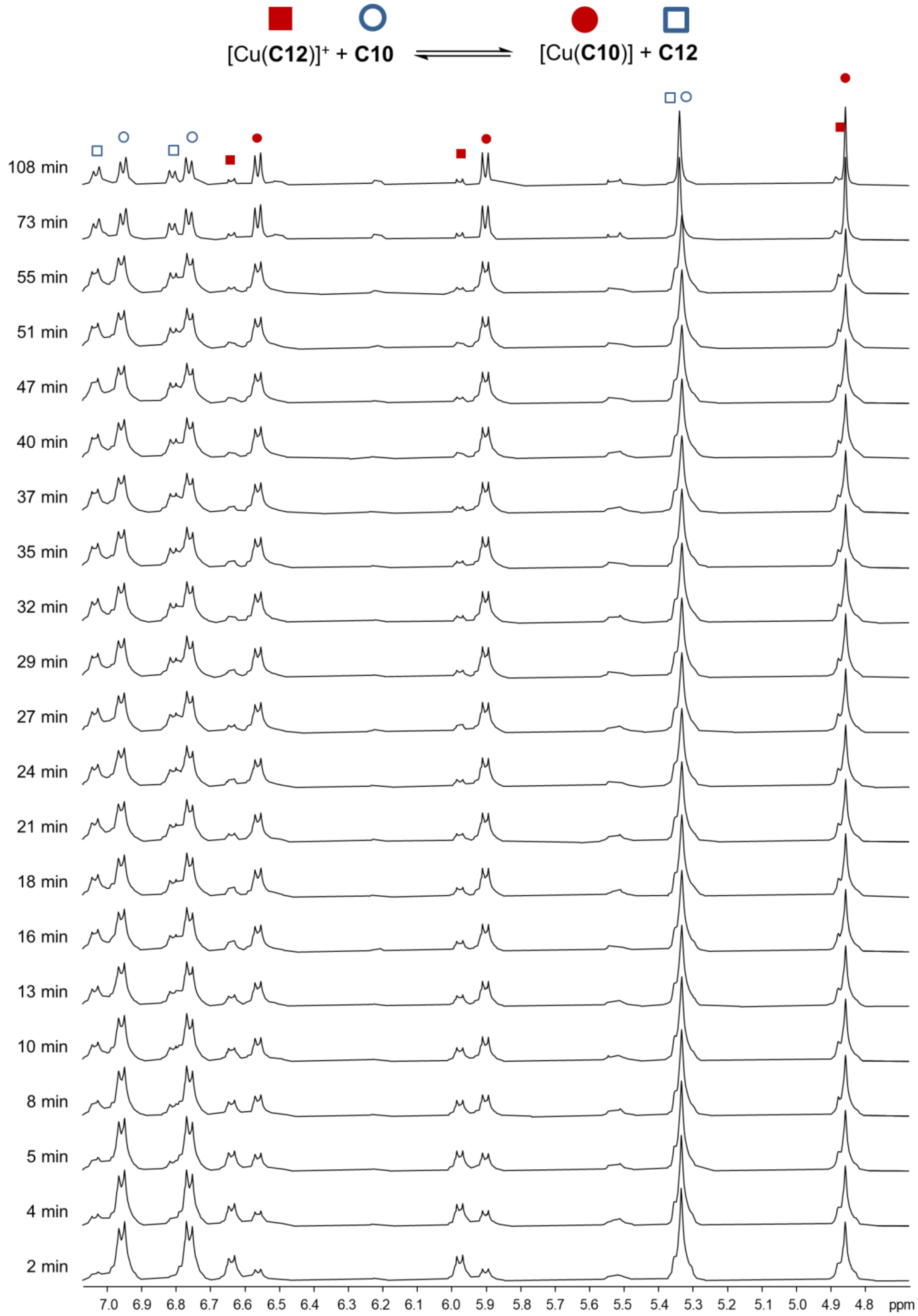

Figure S66. Partial ^{1}H NMR (500 MHz, v/v = 1:1 $CD_3CN/CDCl_3$, 298 K) time-dependent monitoring on ligand scrambling starting from 1:2 (mol/mol) [Cu(**C12**)]$^{+}$/**C10**.

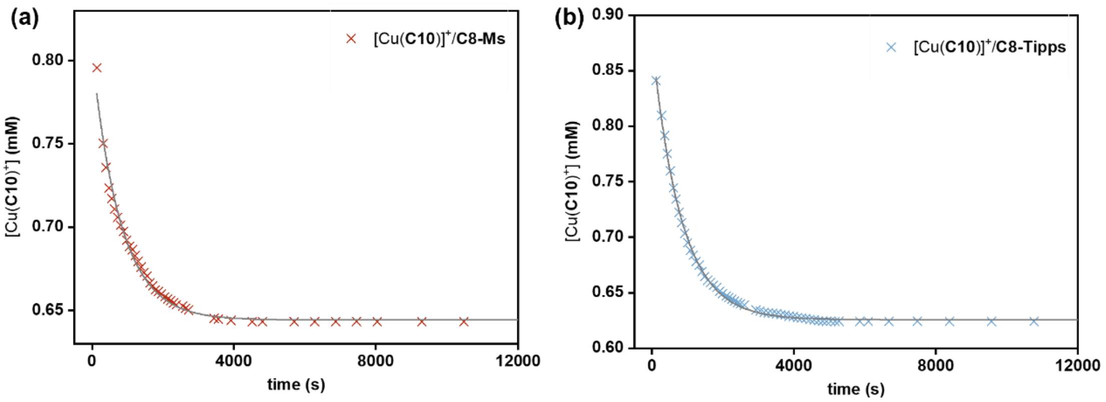

Figure S67. Time-dependent changes of the concentration and the non-linear curve fitting of the scrambling experiment with (a) [Cu(**C10**)]$^+$/**C8-Ms** (1 mM/1 mM); (b) [Cu(**C10**)]$^+$/**C8-Tipps** (1 mM/1 mM) as the starting Cu(I) catenane complex and metal-free [2]catenane ligand.

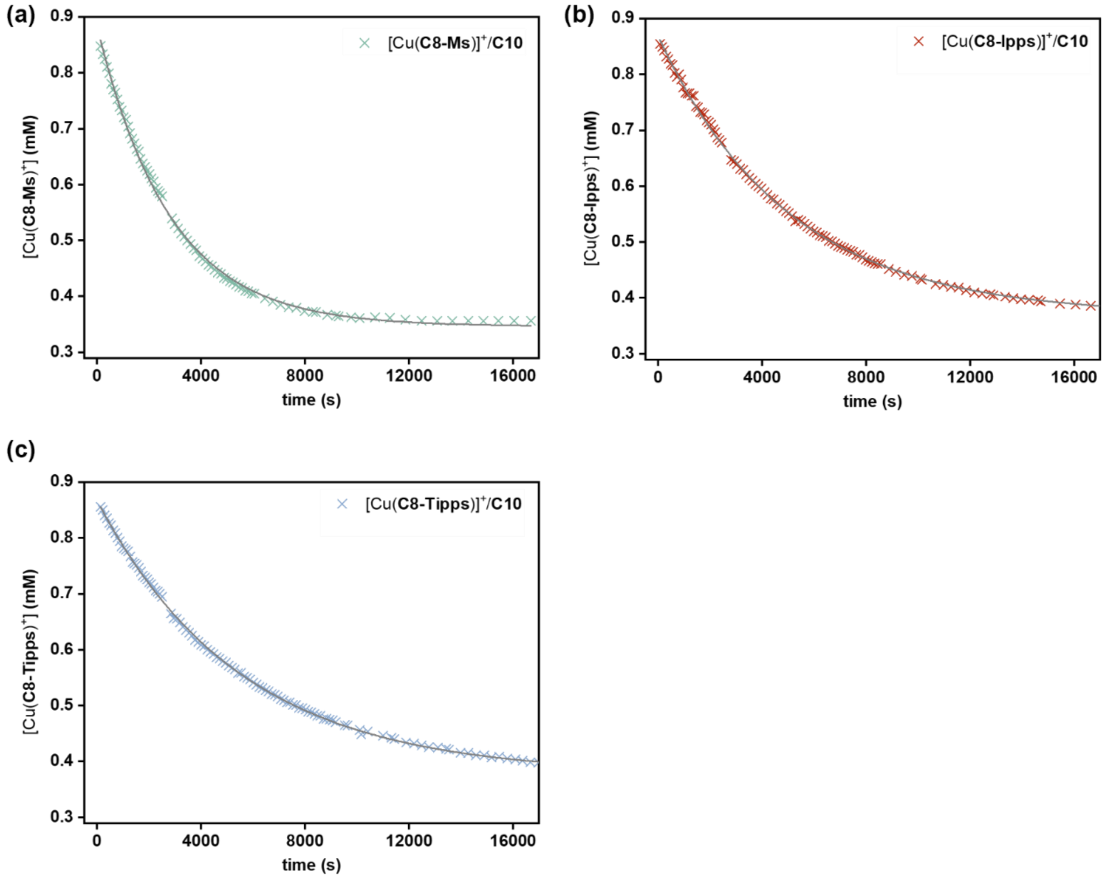

Figure S68. Time-dependent changes of the concentration and the non-linear curve fitting of the scrambling experiment with (a) [Cu(**C8-Ms**)]$^+$/**C10** (1 mM/1 mM); (b) [Cu(**C8-Ipps**)]$^+$/**C10** (1 mM/1 mM); (c) [Cu(**C8-Tipps**)]$^+$/**C10** (1 mM/1 mM) as the starting Cu(I) catenane complex and metal-free [2]catenane ligand.

Table S24. Data of scrambling experiments.

initial conc. (mM)		equilibrium conc. (mM)				K_{ex}*	$y = Ae^{-kx} + B$			
$[Cu(\mathbf{C})]^+$	**C′**	$[Cu(\mathbf{C})]^+$	**C′**	$[Cu(\mathbf{C'})]^+$	**C**		$k \times 10^{-3}$	A	B	R^2
[Cu(**C8-Ms**)]$^+$	**C10**									
1	1	0.36	0.36	0.64	0.64	3.3	0.356	0.536	0.346	0.9984
[Cu(**C8-Ipps**)]$^+$	**C10**									
1	1	0.37	0.37	0.63	0.63	2.8	0.200	0.498	0.369	0.9995
[Cu(**C8-Tipps**)]$^+$	**C10**									
1	1	0.38	0.38	0.62	0.62	2.7	0.182	0.490	0.377	0.9997
[Cu(**C10**)]$^+$	**C8-Ms**									
1	1	0.64	0.64	0.36	0.36	0.3	1.25	0.160	0.644	0.9887
[Cu(**C10**)]$^+$	**C8-Tipps**									
1	1	0.62	0.62	0.38	0.38	0.4	1.23	0.250	0.626	0.9985

*K_{ex} is the equilibrium constant of the catenane scrambling. For each scrambling experiment, the K_{ex} value is consistent with the ratio of the thermodynamic stability constants of the two involved Cu(I) [2]catenane complexes (i.e. $\beta_{C'}/\beta_{C}$).

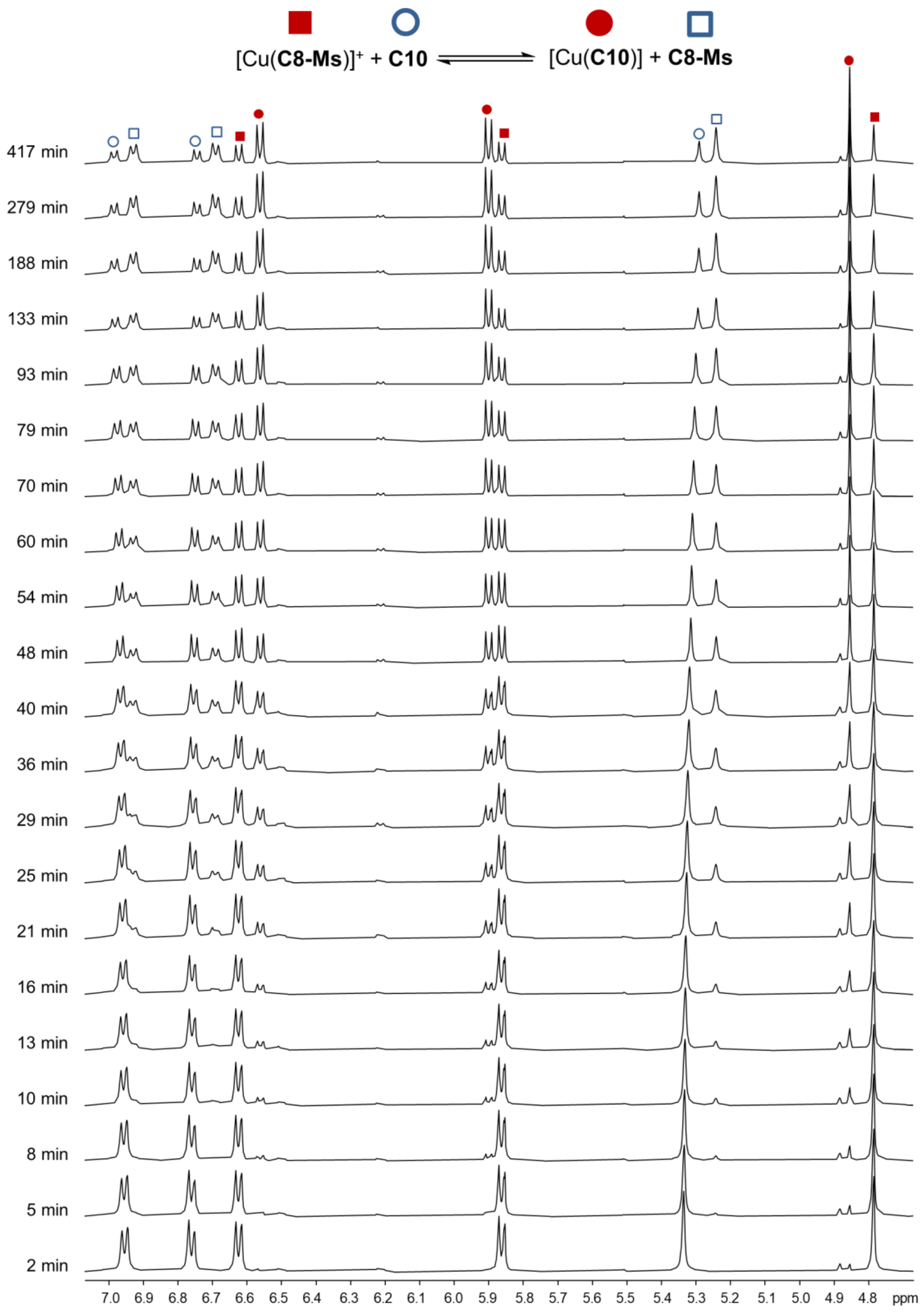

Figure S69. Partial ^{1}H NMR (500 MHz, v/v = 1:1 $CD_3CN/CDCl_3$, 298 K) time-dependent monitoring on ligand scrambling starting from 1:1 (mol/mol) [Cu(**C8-Ms**)]$^+$/**C10**.

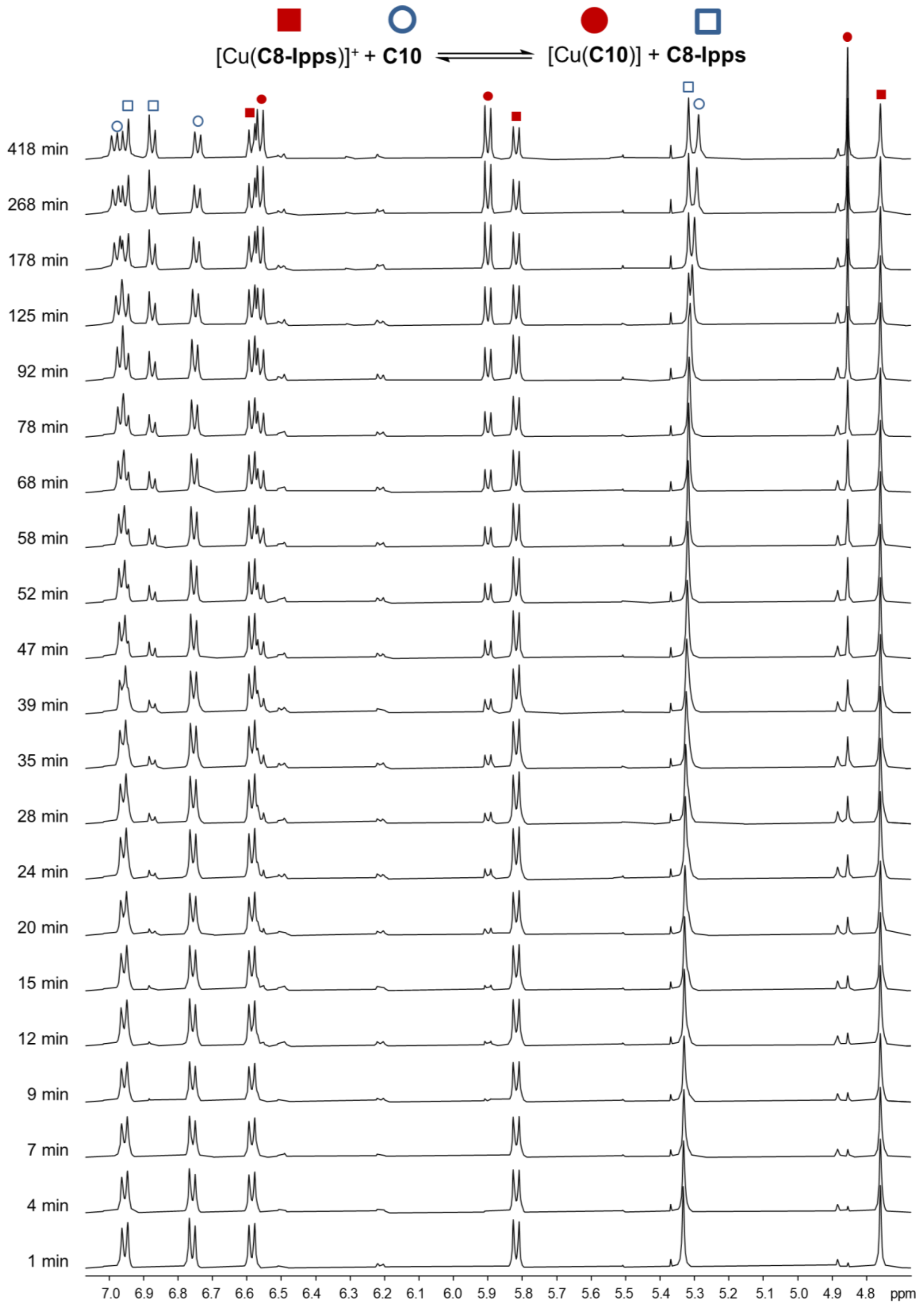

Figure S70. Partial ^{1}H NMR (500 MHz, v/v = 1:1 $CD_3CN/CDCl_3$, 298 K) time-dependent monitoring on ligand scrambling starting from 1:1 (mol/mol) [Cu(**C8-Ipps**)]$^{+}$/**C10**.

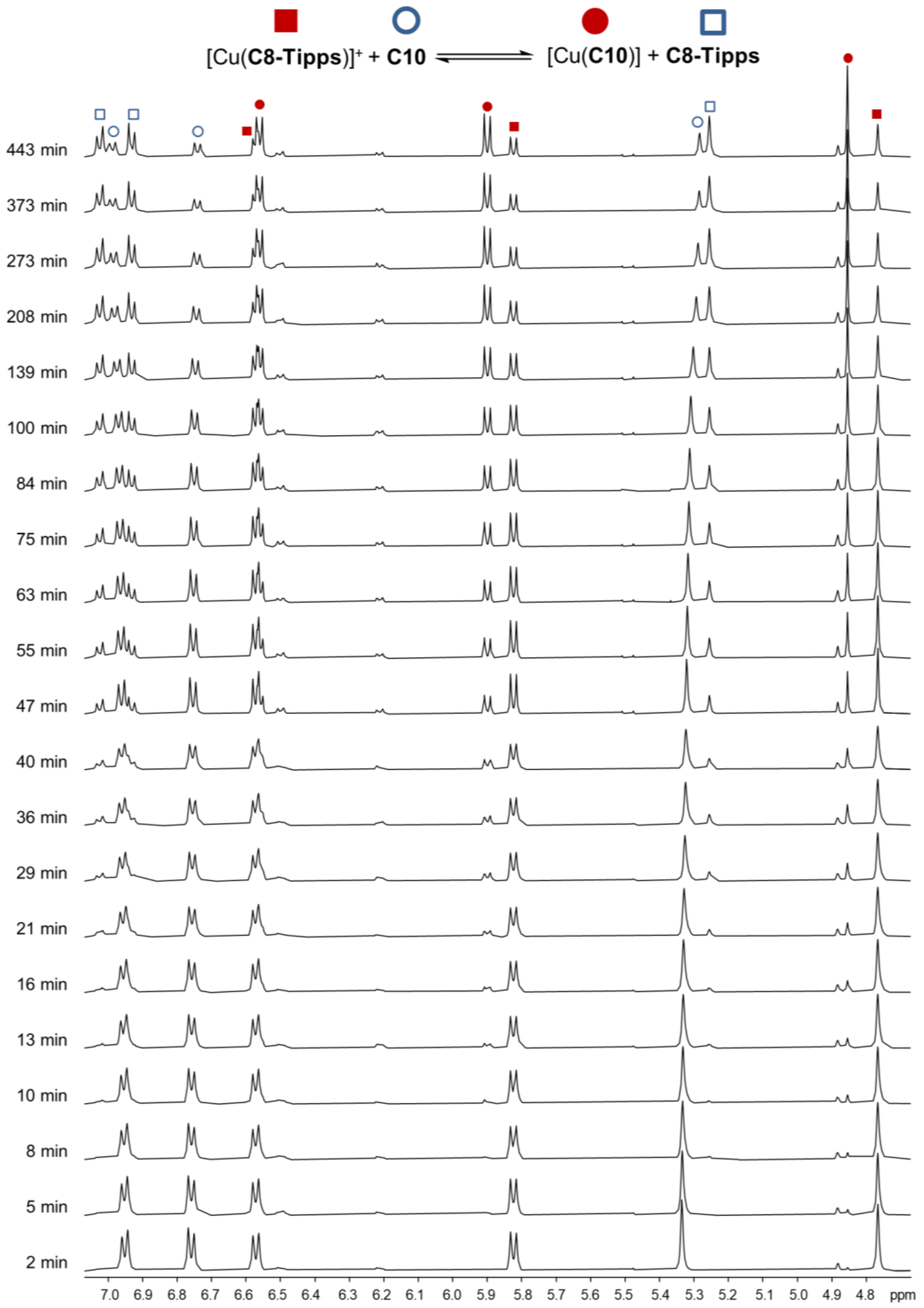

Figure S71. Partial ^{1}H NMR (500 MHz, v/v = 1:1 $CD_3CN/CDCl_3$, 298 K) time-dependent monitoring on ligand scrambling starting from 1:1 (mol/mol) [Cu(**C8-Tipps**)]$^+$/**C10**.

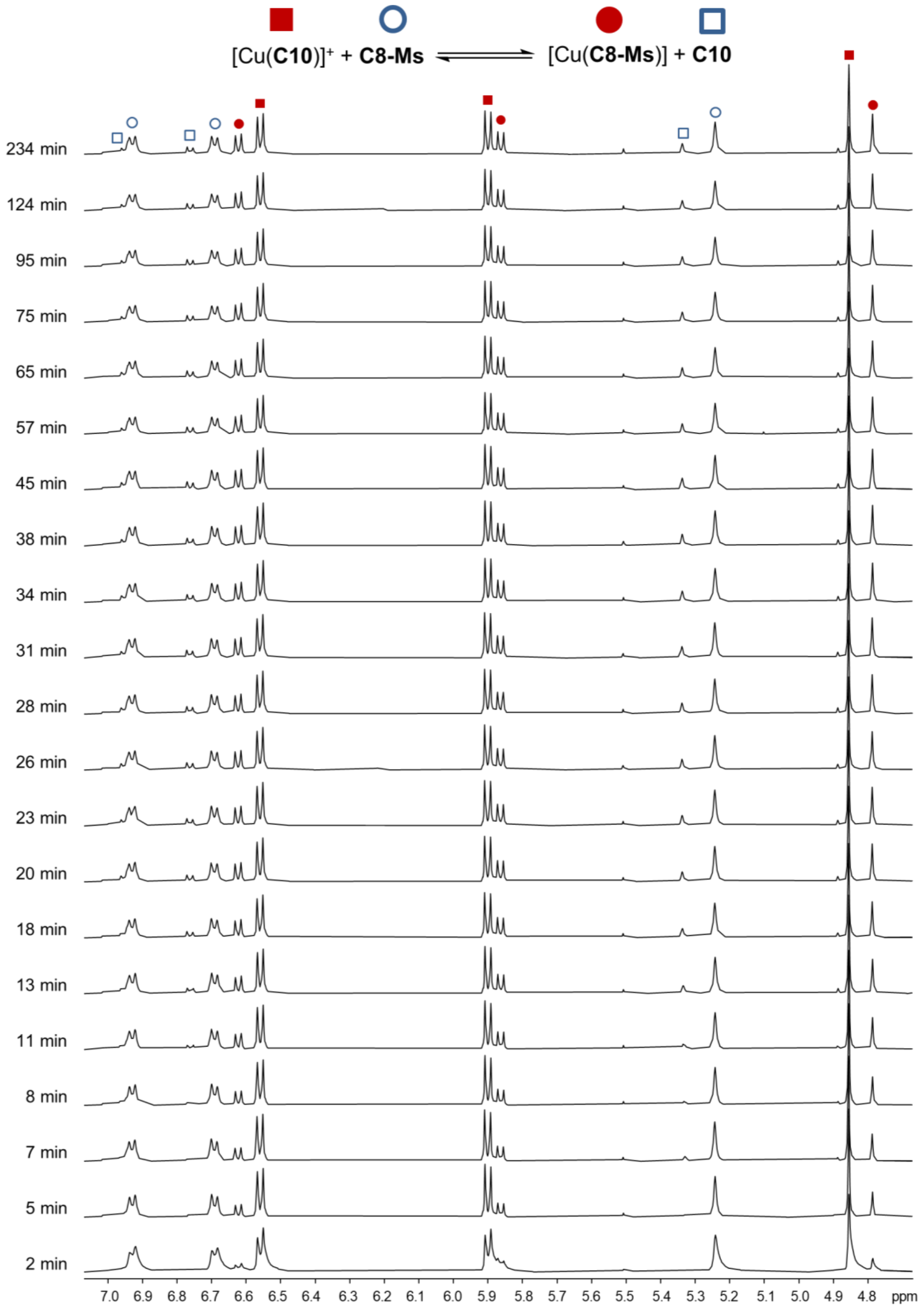

Figure S72. Partial ^{1}H NMR (500 MHz, v/v = 1:1 $CD_3CN/CDCl_3$, 298 K) time-dependent monitoring on ligand scrambling starting from 1:1 (mol/mol) [Cu(**C10**)]$^+$/**C8-Ms**.

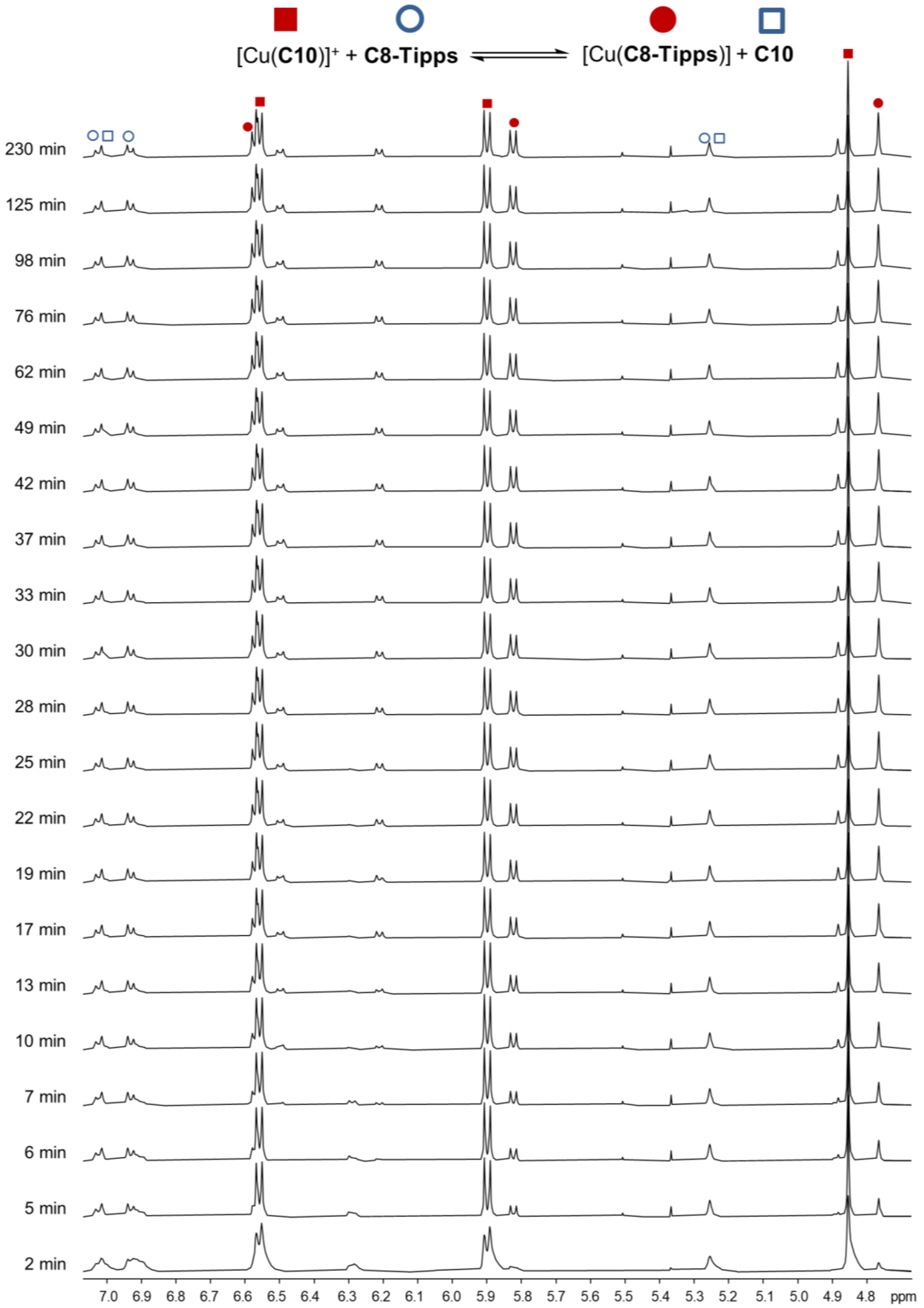

Figure S73. Partial ^{1}H NMR (500 MHz, v/v = 1:1 $CD_3CN/CDCl_3$, 298 K) time-dependent monitoring on ligand scrambling starting from 1:1 (mol/mol) [Cu(**C10**)]$^+$/**C8-Tipps**.

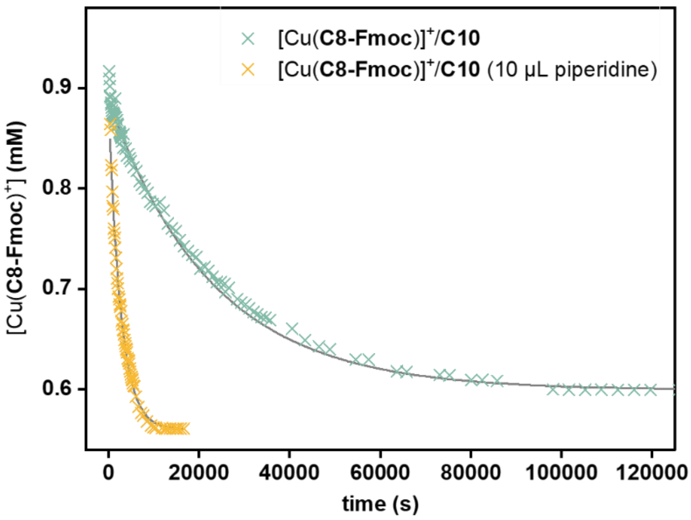

Figure S74. Time-dependent changes of the concentration and the non-linear curve fitting of the scrambling experiment with (a) [Cu(**C8-Fmoc**)]$^+$/**C10** (1 mM/1 mM) as the starting Cu(I) catenane complex and metal-free [2]catenane ligand; (b) [Cu(**C8-Fmoc**)]$^+$/**C10** (1 mM/1 mM) being treated in situ with 10 μL of piperidine at 60 °C for 30 min.

Table S25. Data of scrambling experiments.

initial conc. (mM)		equilibrium conc. (mM)				K_{ex}*	$y = Ae^{-kx} + B$			
[Cu(**C**)]$^+$	**C′**	[Cu(**C**)]$^+$	**C′**	[Cu(**C′**)]$^+$	**C**		$k \times 10^{-3}$	A	B	R^2
[Cu(**C8-Fmoc**)]$^+$	**C10**									
1	1	0.60	0.60	0.40	0.40	0.5	0.0416	0.289	0.599	0.9961
[Cu(**C8-Fmoc**)]$^+$ **	**C10**									
1	1	0.61	0.61	0.39	0.39	0.4	0.380	0.330	0.560	0.9944

*K_{ex} is the equilibrium constant of the catenane scrambling. For each scrambling experiment, the K_{ex} value is consistent with the ratio of the thermodynamic stability constants of the two involved Cu(I) [2]catenane complexes (i.e. $\beta_{C'}/\beta_C$). ** The solution of [Cu(**C8-Fmoc**)]$^+$ was heated at 60 °C for 30 minutes in the presence of 10 μL of piperidine then cooled down to room temperature before the in situ addition of **C10** for further scrambling experiments.

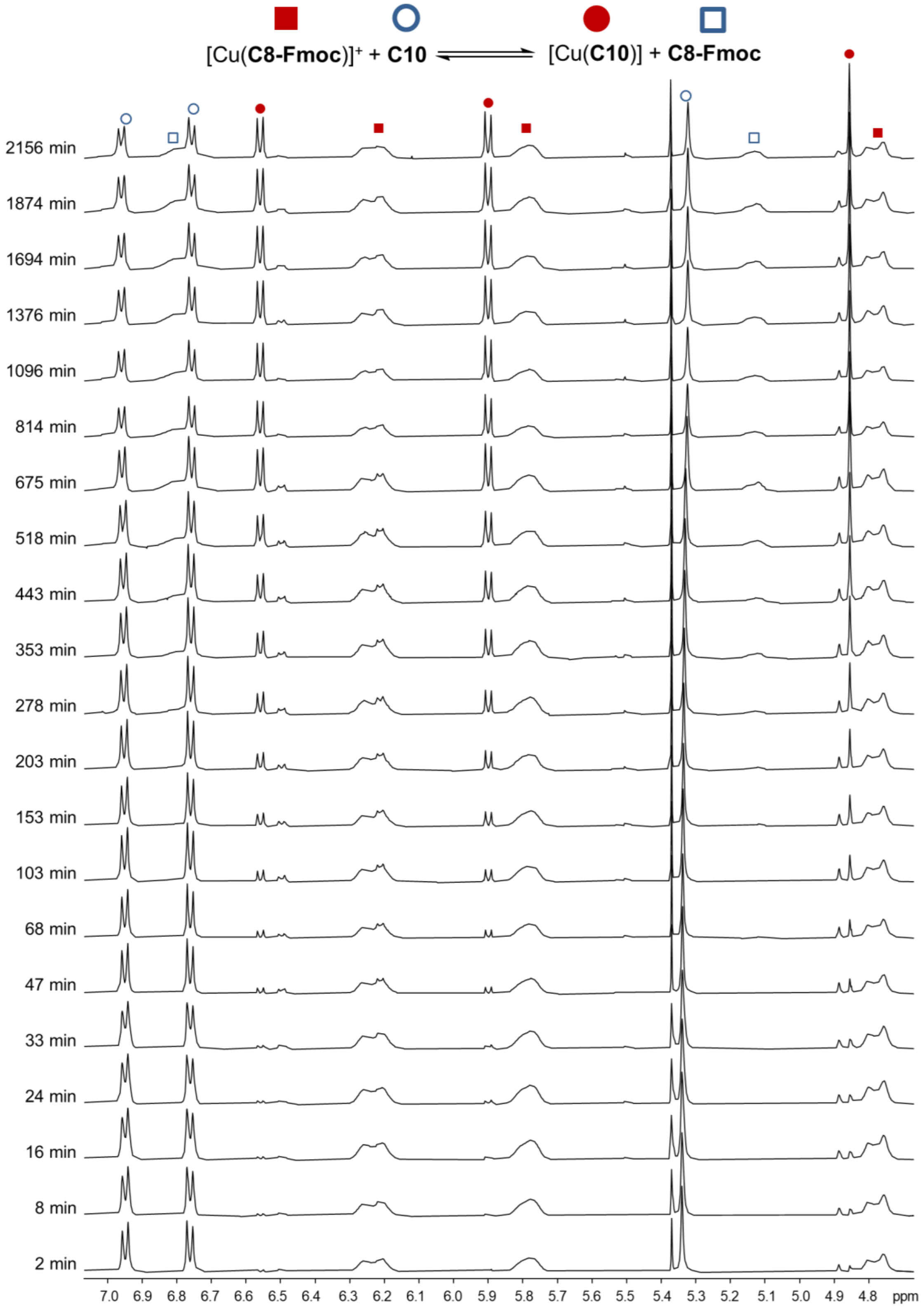

Figure S75. Partial ^{1}H NMR (500 MHz, v/v = 1:1 $CD_3CN/CDCl_3$, 298 K) time-dependent monitoring on ligand scrambling starting from 1:1 (mol/mol) [Cu(**C8-Fmoc**)]$^{+}$/**C10**.

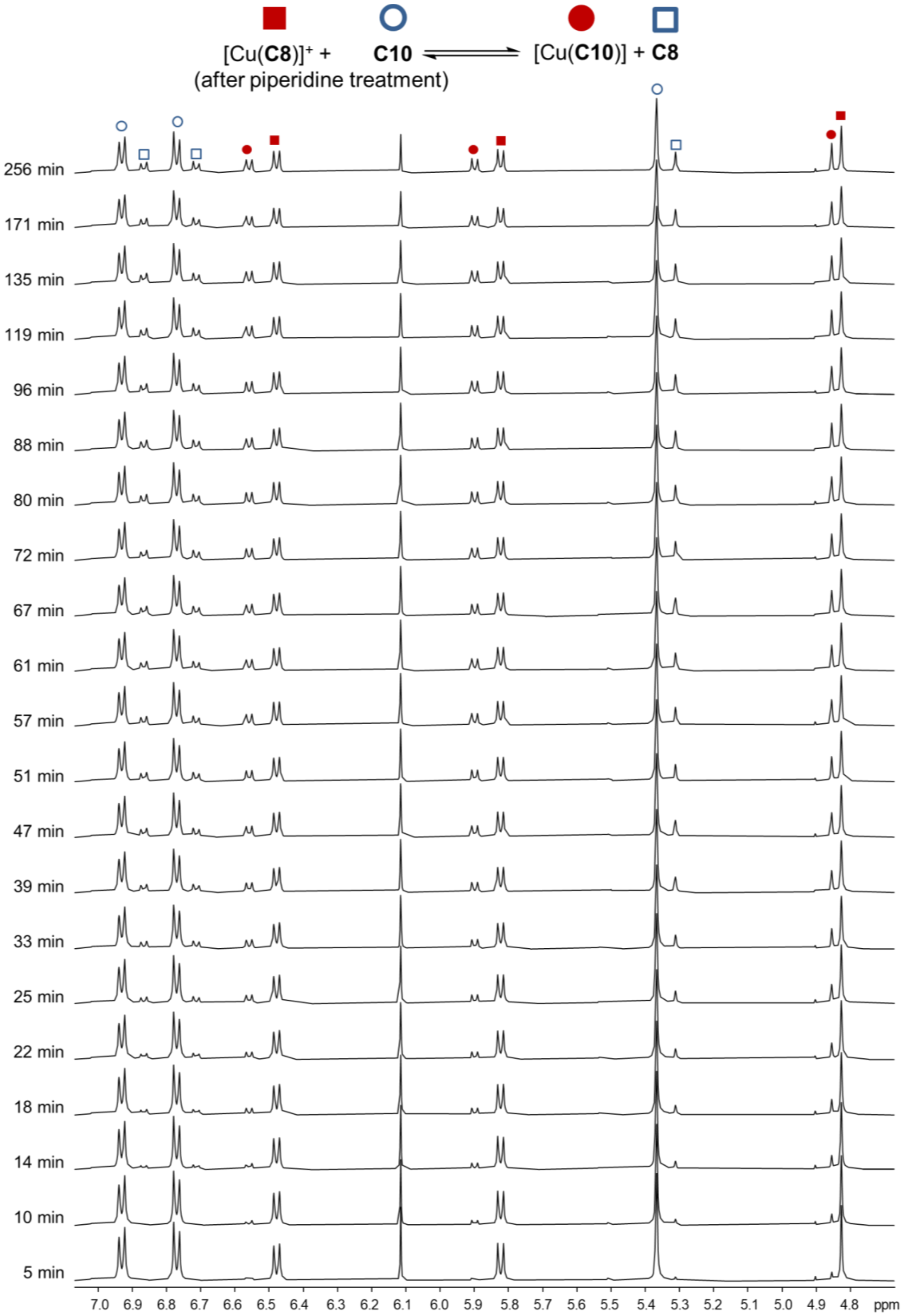

Figure S76. Partial ^{1}H NMR (500 MHz, v/v = 1:1 $CD_3CN/CDCl_3$, 298 K) time-dependent monitoring on ligand scrambling starting from 1:1 (mol/mol) [Cu(**C8-Fmoc**)]$^+$/**C10** being treated in situ with 10 µL of piperidine at 60 °C for 30 min.

9. Catalytic Studies

All catalysis reactions were conducted in 4 mL volume closed vials. For a typical reaction, phenylacetylene 1 (10.2 mg, 0.1 mmol), benzyl azide 2 (13.3 mg, 0.1 mmol), copper catalyst (2 μmol) in MeCN (0.5 mL) was stirred at 60 °C unless otherwise stated. After the reaction was completed, the reaction mixture was passed through a short pad of silica gel and the solvents were evaporated. For evaluating the recyclability of the catenane catalyst, after a 24-hour reaction, the solvents were removed and products were extracted with Et_2O (3 mL × 5). The obtained organic solutions were combined for determination of the yield of the click product (see below). The catenane catalyst [Cu(C**12**)](PF_6) remained in the residue was suspended in 3 mL Et_2O, and was isolated by centrifugation (3 min at 4,000 rpm). After drying under vacuum, the catalyst was used the next round of catalysis. The recovered [Cu(C**12**)](PF_6) after 5 rounds of catalysis was characterized by ^{1}H NMR, which showed no observable decomposition.

Yield of the cycloaddition product was determined by ^{1}H NMR using the triazole resonance at 7.66 ppm of 3 with 1,3,5-trimethoxybenzene as the internal standard. Thin layer chromatography (TLC) was performed on silica gel 60 F254 (Merck, Germany, Aluminum sheet) and column chromatography was carried out on silica gel 60F (Silicycle, Canada). NMR spectra were recorded on Bruker DPX spectrometers with working frequencies of 400 MHz or 500 MHz for ^{1}H, and 101 MHz or 126 MHz for ^{13}C, respectively. Chemical shifts are reported in ppm and referenced to solvent residues (For ^{1}H: $CDCl_3$: δ = 7.26 ppm; for ^{13}C: $CDCl_3$: δ = 77.16 ppm).

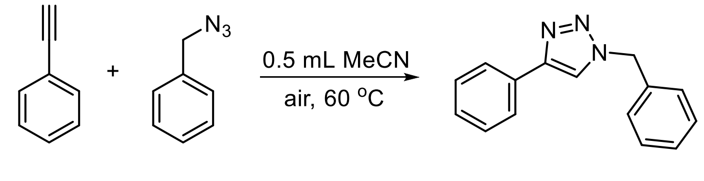

1 0.1 mmol **2** 0.1 mmol **3**

*Synthesis and characterization of **3**.* To a 4 mL volume vial was added phenylacetylene **1** (10.2 mg, 0.1 mmol), benzyl azide **2** (13.3 mg, 0.1 mmol), [Cu(**C12**)]PF_6 (2.9 mg, 0.002 mmol) and MeCN (0.5 mL). The reaction tube was sealed and heated to 60 °C for 24 h. The mixture was diluted with $CHCl_3$ (6 mL) and passed through a short pad of silica, the solvents were then evaporated. The resulting residue was purified by flash column chromatography using ethyl acetate/hexane (v/v = 1:4) as an eluent. Yield: 17.6 mg, 75%. ^{1}H NMR (500 MHz, $CDCl_3$, 298 K) δ 7.80 (d, *J* = 7.0 Hz, 2H), 7.66 (s, 1H), 7.44–7.34 (m, 5H), 7.34–7.27 (m, 3H), 5.57 (s, 2H). ^{13}C NMR (126 MHz, $CDCl_3$, 298 K) δ 148.3, 134.8, 130.7, 129.3, 128.9, 128.9, 128.3, 128.2, 125.8, 119.6, 54.3. HRMS (ESI+): m/z calcd. for $C_{15}H_{13}N_3$ $[M+H]^+$: 236.1182, found: 236.1183.

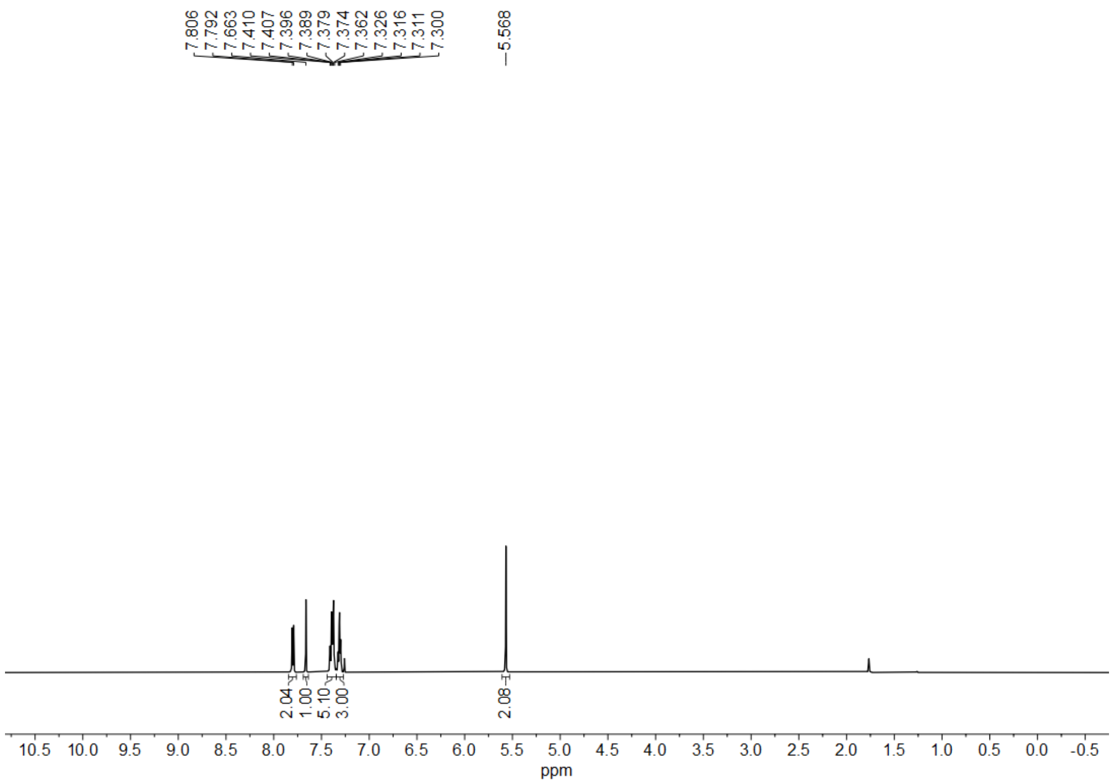

Figure S77. ^{1}H NMR (500 MHz, $CDCl_3$, 298 K) of **3**.

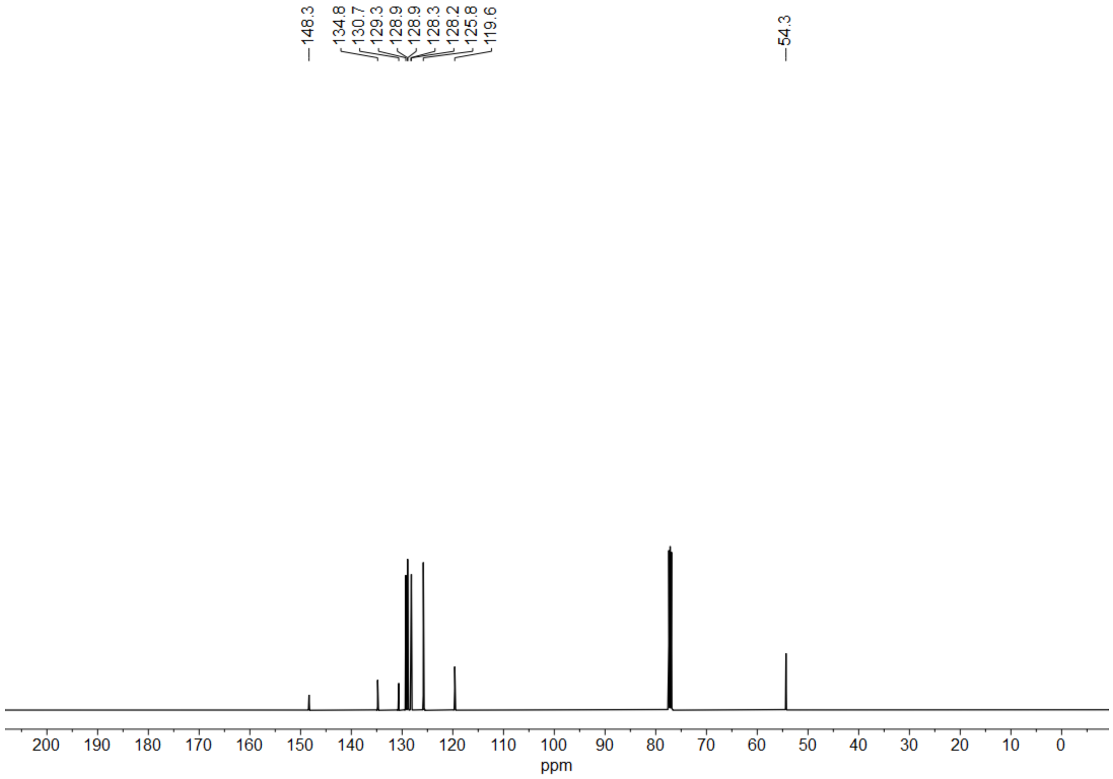

Figure S78. ^{13}C NMR (126 MHz, $CDCl_3$, 298 K) of **3**.

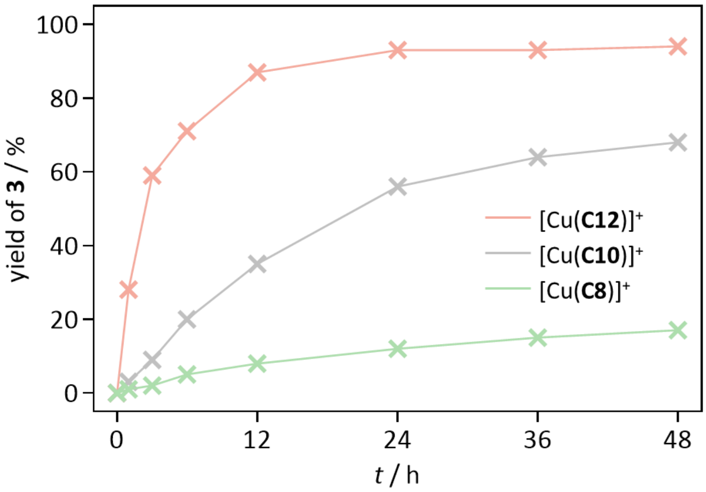

Figure S79. Comparison of the click activity of [Cu(**C8**)]$^+$, [Cu(**C10**)]$^+$, and [Cu(**C12**)]$^+$.

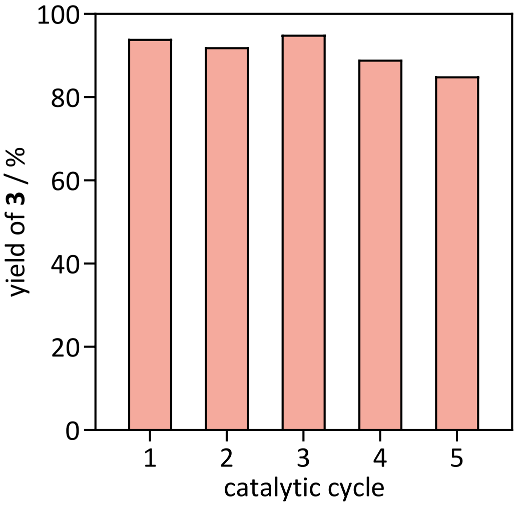

Figure S80. Yields of **3** in five catalytic cycles using [Cu(**C12**)](PF$_6$) as catalyst. A slight decrease in the yield of the click product in the 5th round of catalysis is attributed to a loss of the catenane catalyst during the extraction process.

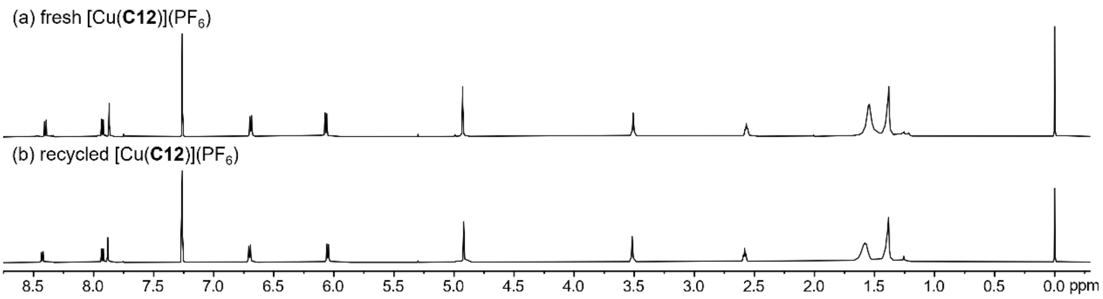

Figure S81. ^{1}H NMR spectra (500 MHz, $CDCl_3$, 298 K) of (a) fresh [Cu(**C12**)](PF$_6$) and (b) [Cu(**C12**)](PF$_6$) recycled after five rounds of catalysis.

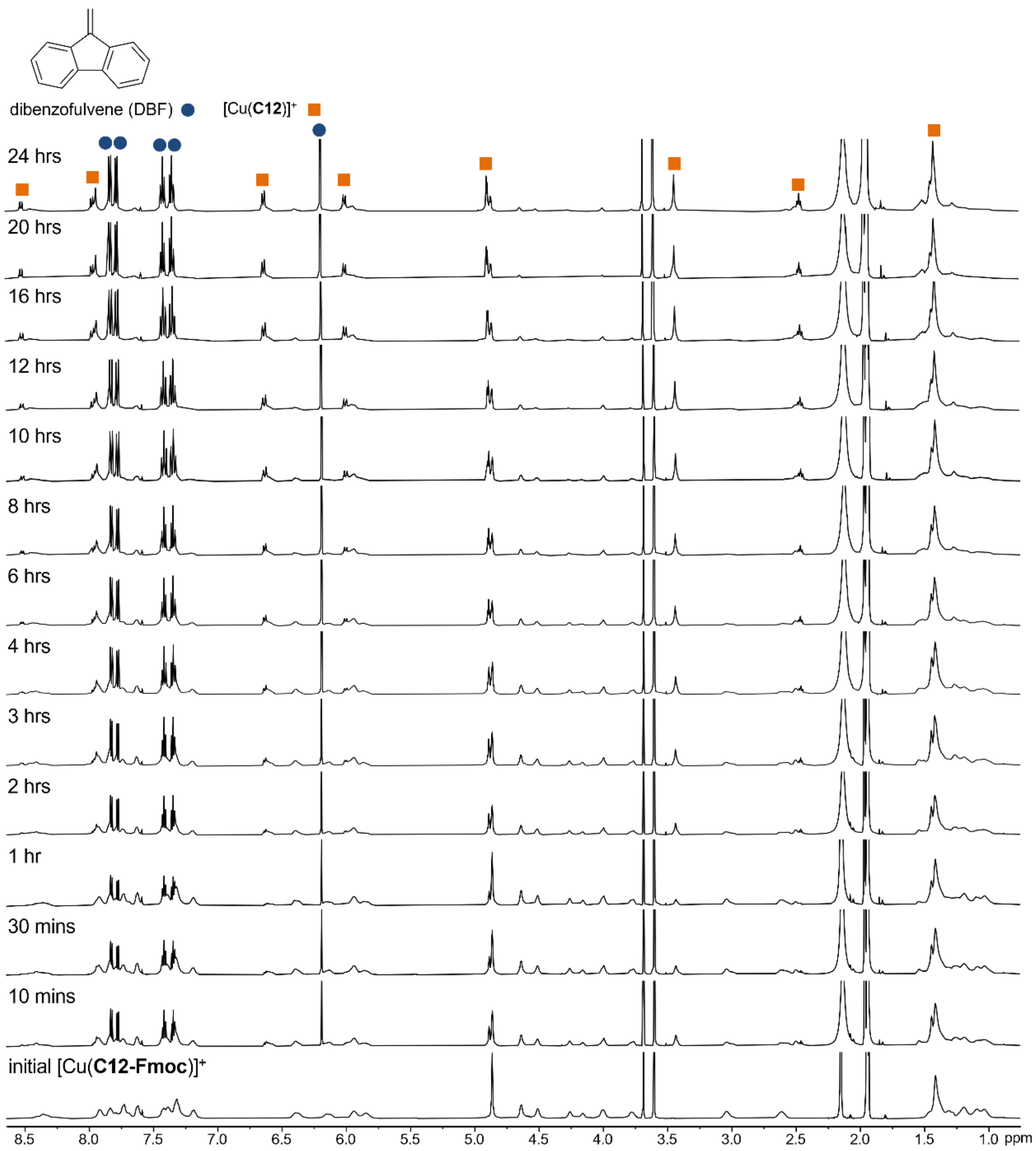

Figure S82. ^{1}H NMR (500 MHz, CD_3CN, 298 K) spectra of a 4 mM [Cu(**C12-Fmoc**)](PF_6) solution in the presence of 1% DBU obtained after heating at 60 °C for different time, showing the successful deprotection of the Fmoc groups.

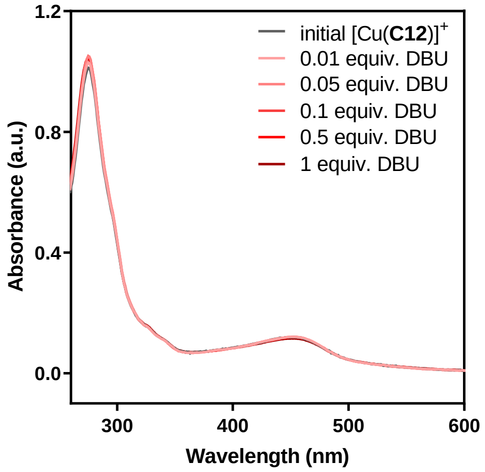

Figure S83. UV-Vis spectra of [Cu(**C12**)](PF_6) (30 μM, MeCN) in the presence and absence of DBU.

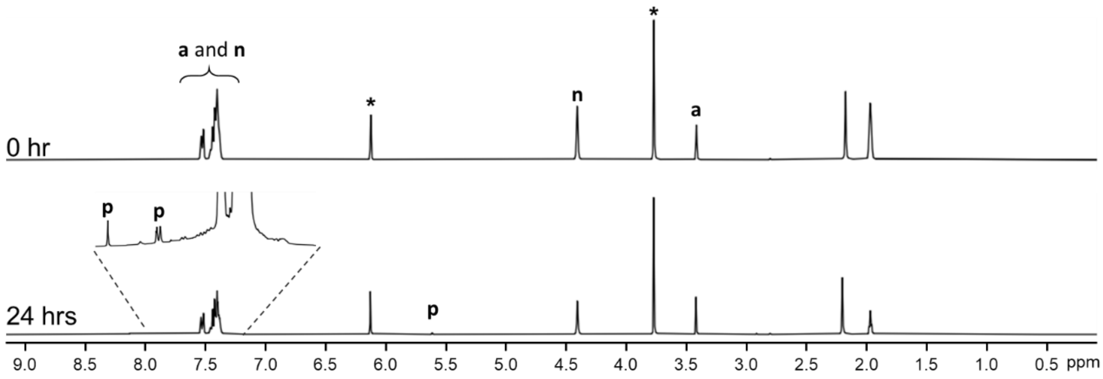

Figure S84. ^{1}H NMR spectra of the reaction mixture of 0.1 mmol each of phenylacetylene (labelled as **a**) and benzylazide (labelled as **n**) in the presence of 0.02 mol% DBU after heating at 60 °C for 24 hours. Only a small amount (<5%) of the triazole product (labelled as **p**) was found. Peaks labelled (*) are from 1,3,5-trimethoxybenzene (internal standard).

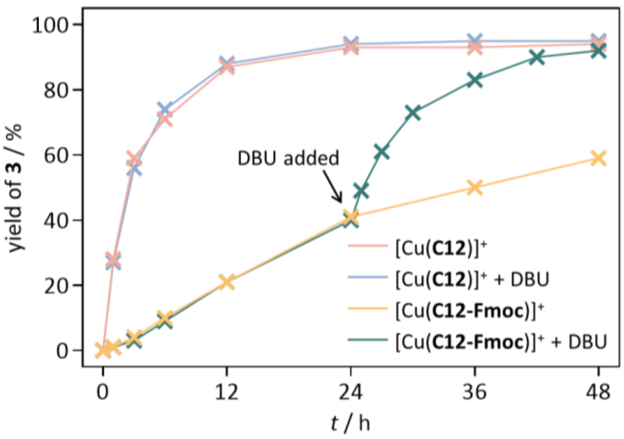

Figure S85. Comparison of the click activity of [Cu(**C12**)]$^+$ and [Cu(**C12-Fmoc**)]$^+$ in the presence and absence of 1% DBU.

10. References

[1] C.-C. Yee, A. W. H. Ng, H. Y. Au-Yeung, *Chem. Commun.* **2019**, *55*, 6169–6172.

[2] L. Zhu, J. Li, J. Yang, H. Y. Au-Yeung, *Chem. Sci.* **2020**, *11*, 13008–13014.

[3] M. Frisch, G. Trucks, H. Schlegel, G. Scuseria, M. Robb, J. Cheeseman, G. Scalmani, V. Barone, B. Mennucci, G. Petersson, **2009**.

[4] H. Ben El Ayouchia, L. Bahsis, H. Anane, L. R. Domingo, S.-E. Stiriba, *RSC Adv.* **2018**, *8*, 7670–7678.

[5] Y. Yang, M. N. Weaver, K. M. Merz, *J. Phys. Chem. A* **2009**, *113*, 9843–9851.

[6] Becke, A. D. Density‐functional thermochemistry. III. The role of exact exchange. *J. Chem. Phys.* **1993**, *98*, 5648–5652.

[7] C. Lee, W. Yang, R. G. Parr, *Phys. Rev. B* **1988**, *37*, 785–789.

[8] P. J. Stephens, F. J. Devlin, C. F. Chabalowski, M. J. Frisch, *J. Phys. Chem. A* **1994**, *98*, 11623–11627.

[9] J. Tomasi, B. Mennucci, R. Cammi, *Chem. Rev.* **2005**, *105*, 2999–3094.

[10] R. Bauernschmitt, R. Ahlrichs, *Chem. Phys. Lett.* **1996**, *256*, 454–464.

[11] M. E. Casida, C. Jamorski, K. C. Casida, D. R. Salahub, *J. Chem. Phys.* **1998**, *108*, 4439–4449.

[12] R. E. Stratmann, G. E. Scuseria, M. J. Frisch, *J. Chem. Phys.* **1998**, *109*, 8218–8224.

[13] T. Lu, F. Chen, *J. Comput. Chem.* **2012**, *33*, 580–592.

[14] R. L. Martin, *J. Chem. Phys.* **2003**, *118*, 4775–4777.

[15] M. D. Hanwell, D. E. Curtis, D. C. Lonie, T. Vandermeersch, E. Zurek, G. R. Hutchison, *J. Cheminform.* **2012**, *4*, 1–17.

[16] L. Du, M.-D. Li, Y. Zhang, J. Xue, X. Zhang, R. Zhu, S. C. Cheng, X. Li, D. L. Phillips, *J. Org. Chem.* **2015**, *80*, 7340–7350.

[17] M.-D. Li, J. Ma, T. Su, M. Liu, L. Yu, D. L. Phillips, *J. Phys. Chem. B* **2012**, *116*, 5882–5887.

[18] A. A. Ermoshkin, E. S. Nikolaeva, D. C. Neckers, A. V. Fedorov, *Macromolecules* **2008**, *41*, 9063–9066.

[19] C. Akilan, E. Königsberger, J. S. Solis, P. M. May, J. H. Kyle, G. Hefter, *Hydrometallurgy* **2016**, *164*, 202–207.

[20] R. M. Izatt, H. Dee Johnston, G. D. Watt, J. J. Christensen, *Inorg. Chem.* **1967**, *6*, 132–135.

[21] J. Lu, D. B. Dreisinger, W. C. Cooper, *Hydrometallurgy* **2002**, *66*, 23–36.

[22] A. M. Albrecht-Gary, Z. Saad, C. O. Dietrich-Buchecker, J. P. Sauvage, *J. Am. Chem. Soc.* **1985**, *107*, 3205–3209.

[23] A. Prabodh, S. Sinn, L. Grimm, Z. Miskolczy, M. Megyesi, L. Biczók, S. Bräse, F. Biedermann, *Chem. Commun.* **2020**, *56*, 12327–12330.
